# Microenvironmental and cell intrinsic factors governing human cDC2 differentiation and monocyte reprogramming

**DOI:** 10.3389/fimmu.2023.1216352

**Published:** 2023-07-19

**Authors:** Magdalena Lang, Corinna Krump, Anastasia Meshcheryakova, Carmen Tam-Amersdorfer, Elke Schwarzenberger, Christina Passegger, Sally Connolly, Diana Mechtcheriakova, Herbert Strobl

**Affiliations:** ^1^Division of Immunology, Otto Loewi Research Center, Medical University of Graz, Graz, Austria; ^2^Insitute of Pathophysiology and Allergy Research, Center for Pathophysiology, Infectiology and Immunology, Medical University of Vienna, Vienna, Austria

**Keywords:** dendritic cell, Langerhans cell, transcriptional reprogramming, lineage decision, epidermal signaling, inflammatory skin disease

## Abstract

cDC2s occur abundantly in peripheral tissues and arise from circulating blood cDC2s. However, the factors governing cDC2 differentiation in tissues, especially under inflammatory conditions, remained poorly defined. We here found that psoriatic cDC2s express the efferocytosis receptor Axl and exhibit a bone morphogenetic protein (BMP) and p38MAPK signaling signature. BMP7, strongly expressed within the lesional psoriatic epidermis, cooperates with canonical TGF-β1 signaling for inducing Axl^+^cDC2s from blood cDC2s *in vitro*. Moreover, downstream induced p38MAPK promotes Axl^+^cDC2s at the expense of Axl^+^CD207^+^ Langerhans cell differentiation from blood cDC2s. BMP7 supplementation allowed to model cDC2 generation and their further differentiation into LCs from CD34^+^ hematopoietic progenitor cells in defined serum-free medium. Additionally, p38MAPK promoted the generation of another cDC2 subset lacking Axl but expressing the non-classical NFkB transcription factor RelB *in vitro*. Such RelB^+^cDC2s occurred predominantly at dermal sites in the inflamed skin. Finally, we found that cDC2s can be induced to acquire high levels of the monocyte lineage identity factor kruppel-like-factor-4 (KLF4) along with monocyte-derived DC and macrophage phenotypic characteristics *in vitro.* In conclusion, inflammatory and psoriatic epidermal signals instruct blood cDC2s to acquire phenotypic characteristics of several tissue-resident cell subsets.

## 1 Introduction

Dendritic cells (DCs) arise from common DC progenitor cells (CDPs) and comprise at least two subsets of conventional DCs (cDC1 and cDC2) and plasmacytoid DCs (pDCs) (1, 2). While DCs are developmentally related to monocytes, both lineages arise from separate bone marrow progenitor cells (i.e. monocyte progenitor cells, vs CDPs) (3). Nevertheless, certain DC populations, such as epidermal Langerhans (LCs) and inflammation-associated monocyte-derived DCs (moDCs) were shown to originate from monocyte committed cells. These DC subsets are now classified as being monocyte-derived cells (4).

DCs and their immediate precursors can be detected as infrequent leukocyte subsets (<1% of mononuclear cells) in human peripheral blood (5–8). Among these, cDC2s can be identified as CD1c^+^ cells that comprise two major subsets, i.e. CD5^+^ (cDC2A) vs CD5^-^ (cDC2B) cell subsets, lacking or exhibiting certain monocyte-affiliated characteristics, respectively (e.g. low levels of monocyte affiliated CD14 and positivity for monocyte/macrophage transcription factor MafB by cDC2B) (1, 9–11). A third, less frequent subset of CD1c^+^ blood DCs co-segregates with CD5^+^CD1c^+^ cells and is marked by the efferocytosis receptor Axl and the leptin-binding protein Siglec-6 (termed “ASDCs”). ASDCs include another subset, lacking CD1c, but exhibiting certain pDC marker characteristics (10). These peripheral blood ASDCs remain poorly characterized functionally, and they reportedly include precursors of both, cDC2s and pDCs. Additionally, cDC3s have recently been delineated among human peripheral blood CD1c^+^ DCs, overlapping with above mentioned cDC2B, and sharing monocyte-affiliated characteristics (10, 12). The developmental heterogeneity of cDC2 lineage related cells (i.e. CD1c^+^ blood DCs) is still a matter of intense investigation.

In addition to blood, cDC2 lineage cells can also be found in anogenital tissues, inflamed tissues and in carcinomas (13–18). For example, the enlarged lesional psoriatic epidermis harbours a predominant population of epithelial DCs (eDC), known to outnumber epidermal-resident Langerhans cells (LCs). These cells express cDC2 markers CD1c and CLEC10A (16, 19) along with epithelial-associated genes such as TAM receptor *Axl* and *E-cadherin* mRNA (16). cDC2s in dermal/interstitial compartments differ phenotypically from epithelial cDC2s, indicating that local signals are critical in modifying cDC’s phenotypic characteristics (1). These tissue-associated signals currently remained poorly defined.

Human blood cDC2s can be induced to rapidly differentiate into CD207^+^ epidermal Langerhans cells (LCs), a cell type previously re-classified as being monocyte-derived (20–23). Such convergence in LC differentiation from developmentally distinct precursors (i.e. cDC2 vs monocytes) is supported by the observation of mixed cDC and monocyte origination of murine oral epithelial LCs *in vivo*. Oral LCs arising from either pathway showed a largely overlapping transcriptome, also shared by epidermal LCs, supporting a strong effect of epithelial signals in LC differentiation (24). This view is supported by previous studies that delineated two waves of progenitor cell to LC differentiation in the inflamed epidermis, with monocyte representing the first and non-monocyte precursors the second wave. Monocyte-derived LCs show a partial LC phenotype and these cells only transiently stayed in the epidermis, whereas precursors of the second wave led to long-lived LCs (25). However, monocytes can reconstitute at low efficiency the long-term LC network after immune injury (26). Together, these studies indicated that microenvironmental signals within the inflamed skin play key instructive functions in DC/LC subset specification of peripheral blood monocytes and cDCs.

Considering that both epithelial LCs in the steady-state and blood precursor-derived LCs in a model of psoriatic inflammation exerted tolerogenic characteristics (27, 28), local epidermal factors appear to be critical for the induction of LC/DC-mediated tolerance. Deciphering these epidermal instructive signals is therefore of considerable medical interest. Moreover, since normal and diseased epithelial cells of various tissues share certain characteristics, carcinoma immune evasion and epithelial DC tolerance might follow similar principles. For example, Gas6-Axl efferocytosis signaling, activated in epithelial cells, promote tolerogenic DC characteristics both in the epidermis and in carcinomas (15, 29).

Considering our lack of understanding of the local epithelial signals instructing DC differentiation during inflammation, we here asked: What are the microenvironmental cues driving epithelial-associated cDC2 differentiation? Moreover, what are the cDC2 intrinsic factors facilitating their differentiation in response to such instructive epithelial signals? We here demonstrated that cooperative lesional signals of the enlarged psoriatic epidermis induce human blood cDC2s to differentiate into epithelial cDC2-like cells. Moreover, blood cDC2 can also acquire characteristics of monocyte-derived DCs.

## 2 Results

### 2.1 Blood cDC2 cells lack KLF4 and show elevated Notch and TGF-β1 signaling relative to monocytes

**Figure 1:**
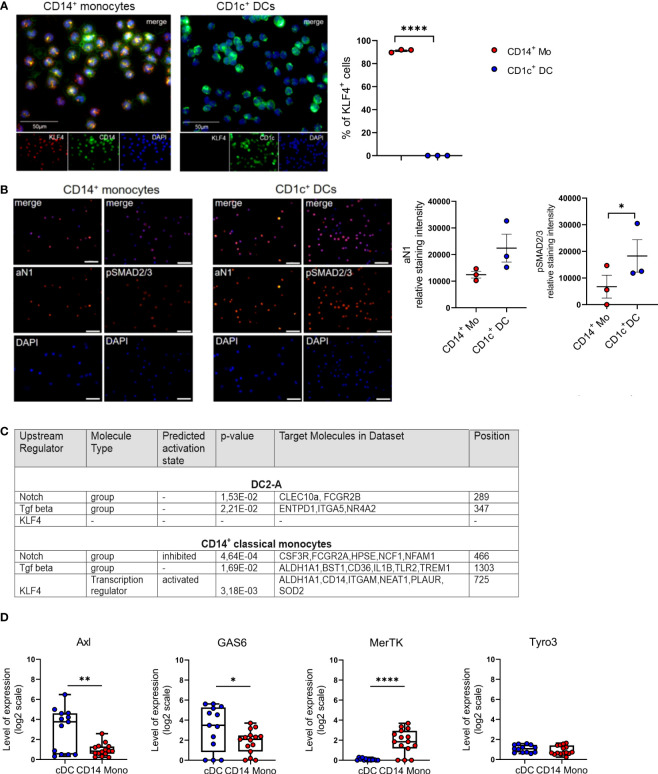
Blood cDC2s lack KLF4 and show an activated Notch-1 and p-SMAD2/3 profile. **(A)** Purified peripheral blood CD1c^+^ DCs and CD14^+^ monocytes were stained for CD1c or CD14 (green) and KLF4 (red) expression. Nuclei were counterstained with DAPI. Scale bar = 50 µm. Only KLF4^hi^ cells were considered for analysis. (n = 3, 2-tailed Student's *t*-test, ****p < 0.0001). **(B)** Immunofluorescence staining of CD1c^+^ DCs and CD14^+^ monocytes stained for intracellular p-SMAD2/3 and active Notch-1 (aN1). Nuclei were counterstained with DAPI. Graph depicts relative staining intensity of p-SMAD2/3 and aN1 across 3 independent experiments analyzed using ImageJ software (n=3, 2-tailed Student's *t*-test, *p < 0.05) **(C)** Identification of Notch group, TGF-β group and KLF4 as Upstream Regulators using the Ingenuity Pathway Analysis Software (IPA) based on DC2-A and classical CD14^+^ monocyte specific signature genes defined by Villani et al. (10). The name of the Upstream Regulator, the molecule type, the predicted activation state and the IPA-derived p-value are given in **Supplementary Table 4**. **(D)** Boxplots representing the GENEVESTIGATOR-based expression profile for the genes of interest of cDCs and CD14^+^ monocytes from four different studies of the publicly available microarray data sets (GSE118165: n=157; GSE115736: n=42; GSE75042: n=9; GSE107011: n=127) from the mRNA-Seq Gene Level Homo sapiens (ref. Ensembl 97, GRch38.p12) platform (mean± SEM, 2-tailed Student’s t-test *(*p* < 0.05), ** (*p* < 0.01) and ****(*p* < 0.0001).

The transcription factor KLF4 reconstitutes macrophage differentiation from PU.1^-/-^ fetal liver cells (30) and is essential for murine monocyte differentiation *in vivo* (31). In human immunohistology, KLF4 exceeds other monocyte-affiliated markers in monocyte lineage assignment (32) and induces monocyte markers CD14 and CD11b when transfected into myeloid cell lines (30). Moreover, KLF4 interferes with TGF-β1-mediated impairment of pro-inflammatory cytokine production by monocytic cells (33). We recently demonstrated that Notch represses KLF4 in human CD14^+^ monocytes, thereby rendering these cells capable of differentiating into LCs in response to TGF-β1/RUNX3 signaling. Mechanistically, KLF4 bound to the RUNX3 promoter in KLF4^hi^ monocyte-derived cells, likely interfering with RUNX3 transcription (34). Since in contrast to monocytes, CD1c^+^ blood cDC2 do not rely on Notch co-stimulation for differentiating into LCs *in vitro* (21), we assessed their KLF4 protein expression status. We purified CD1c^+^ blood cDC2s from mononuclear cell preparations by first depleting CD14^+^ monocytes and CD19^+^ B cells, followed by CD1c positive selection. While expectedly KLF4 is expressed by virtually all CD14^+^ monocytes, cDC2s lack KLF4 (**Figure 1A**). To address whether absence of KLF4 in cDC2 is associated with augmented TGF-β1/Notch signaling, we subsequently analyzed active Notch-1 (aN1) and canonical phospho-SMAD2/3 (p-SMAD2/3). Immunocytology analysis revealed that blood cDC2s exhibit elevated aN1 and p-SMAD2/3 levels relative to CD14^+^ monocytes (**Figure 1B**). Intracellular flow cytometry, albeit less sensitive, were supportive of these findings (**Supplementary Figure 1**). To validate these observations and gain mechanistic insights, we performed integrative data analysis of RNA seq datasets from blood cDC2s and CD14^+^ monocytes. We asked whether the increased activation of Notch and TGF-β signaling pathways in blood cDC2s can be verified based on their transcriptional profile. We therefore dissected the Upstream Regulators attributed to the blood cDC2s and CD14^+^ monocyte associated signature genes [based on (10)] using the Ingenuity Pathway Analysis platform (IPA). Among the significant Upstream Regulators, we identified TGF-β and Notch- attributed gene sets (defined by IPA and named therein as groups) for both cell types. Although the Notch and TGF-β group were similarly ranked in the blood cDC2s relative to CD14^+^ monocytes (Notch: p-value =1.53E-02, position 289/527 versus p-value =4.64E-04, position 466/2063; TGF- β: p-value = 2.21E-02, position 347/527 versus p-value =1.69E-02, position 1303/2063), the Notch group was predicted to be inhibited in CD14^+^ classical monocytes. Consistent with our immunostaining results (**Figure 1A**), we identified KLF4 only in CD14^+^ classical monocytes with an activated predicted state (p-value = 3.18E-03; position 725/2063) (**Figure 1C** and **Supplementary Table 4**). The analysis of known TGF-β regulated molecules in the monocyte/DC system also showed consistent results. Members of the TAM family of efferocytosis receptors are inversely controlled by TGF-β1 in cells of the mononuclear phagocyte system, with Axl and its ligand Gas6 induced, and MerTK repressed (29). Analyzing datasets from four different transcriptomic studies revealed that *Axl/Gas6* are indeed differentially expressed by peripheral blood cDCs versus classical CD14^+^ monocytes, with cDCs showing upregulated expression of *Axl* and ligand *Gas6*. In contrast, TAM receptor *MerTK* exhibited reduced expression levels in cDCs compared to CD14^+^ monocytes. Expectedly, *Tyro3* levels remained similar in both cell types (**Figure 1D**) (29). In conclusion, cDC2s lack detectable KLF4 protein, and show elevated constitutive Notch and TGF-β signaling relative to CD14^+^ monocytes.

### 2.2 Blood cDC2s can be induced to differentiate into KLF4^hi^ moDCs and macrophages

**Figure 2:**
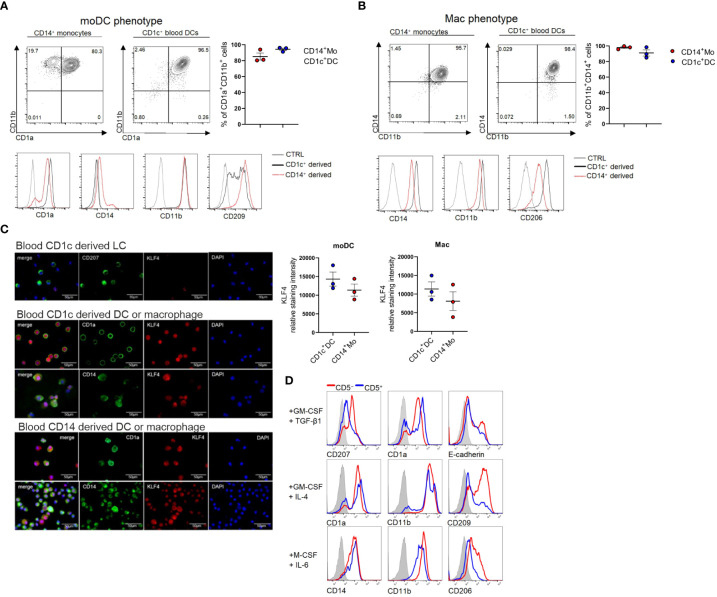
Blood cDC2s differentiate into macrophages, moDCs and LCs *in vitro*. **(A)** CD1c^+^ DCs and CD14^+^ monocytes were cultured under moDC promoting conditions GM-CSF + IL-4 or **(B)** macrophage promoting conditions (M-CSF + IL-6) and FACS analyzed for the respective lineage markers on day 5. Bar graphs represent percentages of CD11b^+^CD1a^+^ (moDC) and CD14^+^CD11b^+^ (mac) cells (n = 3, mean ± SEM). **(C)** Cytospins of CD1c^+^ DC-derived LCs (top), CD1c^+^ DC-derived moDCs and macrophages (middle) as well as CD14^+^ cell-derived moDC and macrophages (bottom) were stained for the indicated markers. Scale bar = 50 µm. Graph depicts the relative staining intensity of KLF4 analyzed using ImageJ software (n=3). **(D)** CD1c^+^ DCs were sorted according to their CD5 expression status. CD5^+^ and CD5^-^ cells were cultured with the indicated cytokines (n=1, 6 donors were pooled for sorting).

Since we previously observed that KLF4 is upregulated upon moDC and macrophage differentiation of CD14^+^ monocytes in response to GM-CSF/IL-4 and M-CSF/IL-6, respectively (34), we asked whether KLF4 can be induced in blood cDC2s in response to these stimuli. Indeed, we observed that cDC2 can be induced to acquire moDC (CD11b^+^CD1a^+^CD209^+^) (**Figure 2A**) and macrophage (CD14^+^CD11b^+^CD206^+^) (**Figure 2B**) phenotypic characteristics in response to GM-CSF/IL-4 and M-CSF/IL-6 stimulation, and this is accompanied by KLF4 induction, with KLF4 expression levels equalling or exceeding those observed in monocytes cultured in parallel under identical cytokine conditions (**Figure 2C**). Expectedly (34), CD1c^+^ cell-derived CD207^+^ LCs generated in response to GM-CSF plus TGF-β1 lacked any detectable KLF4 protein (**Figure 2C**). Moreover, pooled (n=6) sub-sorted fractions of blood cDC2s (i.e. CD5^+^ versus CD5^lo/-^ (9), exhibited similar potency to differentiate into macrophages and moDCs, with the limitation that CD5^+^ cells failed to gain high levels of CD209, a key marker for moDCs (35) that is selectively regulated in moDCs by activation signals (36) (exemplified in **Figure 2D**). Therefore, blood cDC2s can be induced to differentiate into KLF4^hi^ cells exhibiting phenotypic characteristics of moDCs and macrophages.

### 2.3 Psoriatic lesion-associated epithelial signals promote Axl^+^cDC2 differentiation, and essential role of canonical TGF-β1 signaling in Axl induction

**Figure 3:**
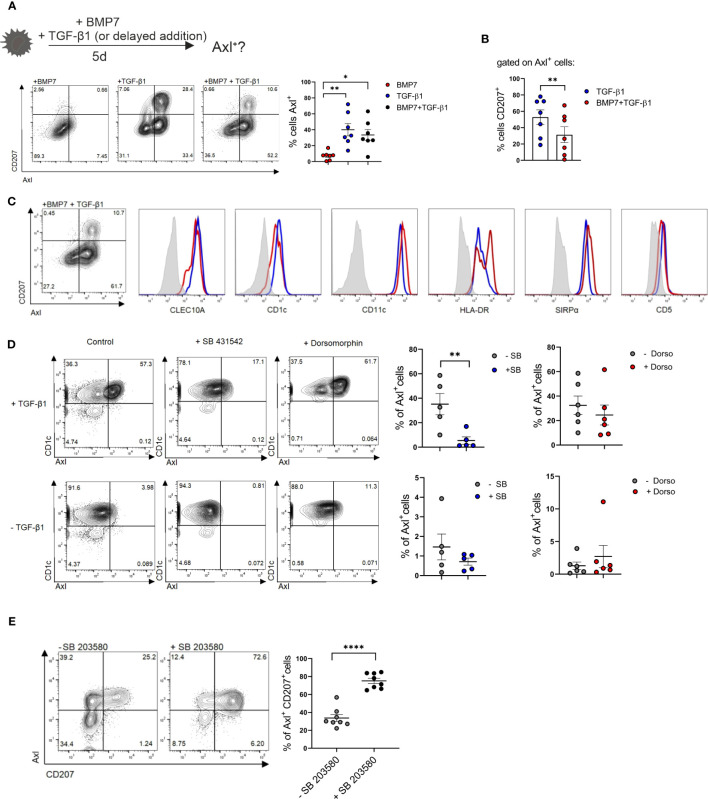
Axl and CD207 induction by blood cDC2s. **(A)** Schematic presentation of the experimental set-up of LC/Axl^+^DC differentiation culture from blood cDC2s. Blood cDC2s were stimulated with either BMP7, TGF-β1 or BMP7+TGF-β1 (2d) and analyzed for surface CD207 and Axl expression on day 5. Graph represents percentages of cells Axl^+^ in the respective cultures [n=7, mean± SEM, one-way ANOVA (*p <0.05; **p <0.01)]. **(B)** Graph shows percentages of CD207^+^ cells gated on Axl^+^ cells of TGF-β1 and BMP7+TGF-β1 differentiation cultures. [n=7, mean± SEM, paired 2-tailed Student’s t-test (**p <0.01)]. **(C)** Representative flow cytometry analysis of cDC2 marker genes of Axl^+^CD1a^+^CD207^+^ and Axl^+^CD1a^+^CD207^-^ cells in cytokine cocktail containing BMP7 plus TGF- β1 from CD1c^+^ DCs (n=3). **(D)** Blood cDC2s ± TGF-β1 were exposed to an ALK2/3/6 inhibitor (Dorsomorphin, n=6) or ALK2/5/7 inhibitor (SB 431542, n=5) and analyzed for Axl expression after 2 days. Graph represents percentages of Axl^+^ cells on day 2 [mean± SEM, paired 2-tailed Student’s t-test (**p, <0.01)]. **(E)** TGF-β1 stimulated blood cDC2s were exposed to the p38MAPK inhibitor SB 203580 and analyzed for CD207 vs Axl after 2 days. Graph depicts the quantification of Axl^+^CD207^+^cells in cultures supplemented or not with SB 203580 [n=8, mean± SEM, paired 2-tailed Student’s t-test (****p < 0.0001)].

The enlarged lesional psoriatic epidermis is populated by at least three representatives of the mononuclear phagocyte and DC system (16, 18, 19). In addition to two subsets of CD207^+^LCs (representing steady-state LCs and neo-appearing CD1c^+^LCs), also CD207^-^eDCs, outnumbering steady-state LCs, were described (16, 37). An overview of markers for classifying these DC subsets is shown in **Supplementary Table 1**. Their lineage relationship and the nature of the epithelial differentiation signals remained poorly defined. Since TGF-β1 promotes CD207^+^LC differentiation from blood monocytes (22) and cDC2s (21, 38) *in vitro*, we first quantified TGF-β1 and downstream p-SMAD2/3 in non-involved versus lesional epidermal sites. This revealed equivalent expression levels (**Supplementary Figures 2A, B**). This contrasted with the previously observed aberrant high BMP7 and downstream p-SMAD1/5/8 signal throughout the multilayered lesional epidermis (39, 40). Thus, both TGF-β1 and BMP7 signaling pathways are active in psoriatic epidermal cells. Since CD207^-^eDCs in the inflamed epidermis reportedly express *Axl* mRNA (16) and Axl is similarly expressed by LCs (29), we reasoned that Axl protein might represent a useful pan-DC marker in stratified epithelia/epidermis, potentially enabling to delineate epithelial DC subset differentiation. To address this question, we first studied Axl expression by blood cDC2 *in vitro*, followed by human tissue analysis, and using a differentiation model of human progenitor cells (see below). Purified CD1c^+^ blood DCs (i.e. termed cDC2) were cultured in the presence of GM-CSF for 5 days, and the effects of addition of BMP7 and/or TGF-β1 on cell differentiation was analyzed using flow cytometry. Initial experiments revealed that BMP7 shows little or no effect on Axl expression by cDC2s, when added to GM-CSF supplemented cultures (**Supplementary Figure 3B**). Conversely, Axl was induced to high levels by large percentages of blood cDC2s in response to TGF-β1 *in vitro*. In these differentiation cultures, Axl^+^ cells were sub-divided into two distinct cell subsets based on CD207 expression (**Figure 3A**). Pre-stimulation of cells with BMP7 for 3 days, followed by TGF-β1 for additional 48 h led to the preferential generation of Axl^+^CD207^-^cDC2 (**Figures 3A, B**). Both Axl^+^CD207^-^ and Axl^+^CD207^+^ cells generated from blood cDC2s in presence of TGF-β1 plus BMP7 exhibited cDC2 marker characteristics (CD1c^+^CLEC10A^+^CD11c^hi^SIRPα^+^CD5^+/-^) (**Figure 3C**). The CD207^+^ cells in TGF-β1 only cultures showed equivalent expression of cDC2-affiliated CD1c, CLEC10A and SIRPα, but less HLA-DR relative to Axl^+^cDC2s (**Supplementary Figure 4**). These phenotypic data of cDC2-derived CD207^+^ cells are confirmative to previous observations (21). Given the previous description of a minor subset of Axl^+^CD1c^+^ blood DCs (10), and of TGF-β synthesis by cDC2 (41), we next asked whether the constitutive Axl expression by Axl^+^CD1c^+^ blood DCs might similarly be regulated by TGF-β1. TGF-β1-induced Axl in blood cDC2s can be blocked by the addition of an inhibitor of canonical TGF-β1 signaling (ALK4/5/7 inhibitor SB 431542). The low constitutive Axl expression by blood cDC2s observed in most samples analyzed in the absence of TGF-β1 was also consistently reduced by the ALK4/5/7 inhibitor, indicating that canonical TGF-β1 signaling may maintain constitutive Axl expression by blood cDC2s. Given that TGF-β1 signals through BMPR1a/ALK3 to promote LC differentiation (42, 43), we added dorsomorphin, an inhibitor of BMP signaling, targeting type 1 BMP receptors ALK2/3/6 to parallel cultures. Basal and TGF-β1-induced Axl expression remained unaffected by dorsomorphin (**Figure 3D**). Moreover, since KLF4 interferes with TGF-β1 signaling in monocytes (33, 34), we asked whether short-term stimulation of cDC2 with GM-CSF/IL-4, i.e. factors promoting KLF4^hi^ moDCs (**Figures 2A, C**), might abrogate Axl induction. This was indeed the case (**Supplementary Figure 6**). In addition to strong BMP7 signaling, psoriatic lesional cells are marked by p38MAPK activation (44), and its activation in keratinocytes promotes psoriasis-like lesion formation in mice (45). Recently, the c-Jun/AP-1 signaling complex downstream of p38MAPK has been found to be highly activated in human psoriatic lesions. Particularly, cDC2s recruited to psoriatic inflammatory lesions show strong c-Jun activation, resulting in enhanced pro-inflammatory cytokine production (46). Therefore, we analyzed the role of p38MAPK activation on blood cDC2s undergoing TGF-β1-dependent Axl^+^ cell differentiation. Since p38MAPK signaling is known to be activated in GM-CSF stimulated cells *in vitro* [reviewed in (47)], we assessed the contribution of p38MAPK signaling in cDC2 differentiation, by adding a small molecule inhibitor. Short-term addition of the p38MAPK inhibitor SB 203580 diminished Axl^+^cDC2 in favour of CD207^+^ LC generation among Axl^+^ cells (**Figure 3E**). Together, these data revealed that two signals aberrantly induced in the lesional psoriatic epidermis (i.e. BMP7/BMPR1a and p38MAPK) promote Axl^+^cDC2 differentiation from blood cDC2. Moreover, diminishment of these lesional signals promotes CD207^+^ LCs at the expense of Axl^+^cDC2s.

### 2.4 Psoriatic lesion-associated eDCs exhibit a BMP7-driven gene program

**Figure 4:**
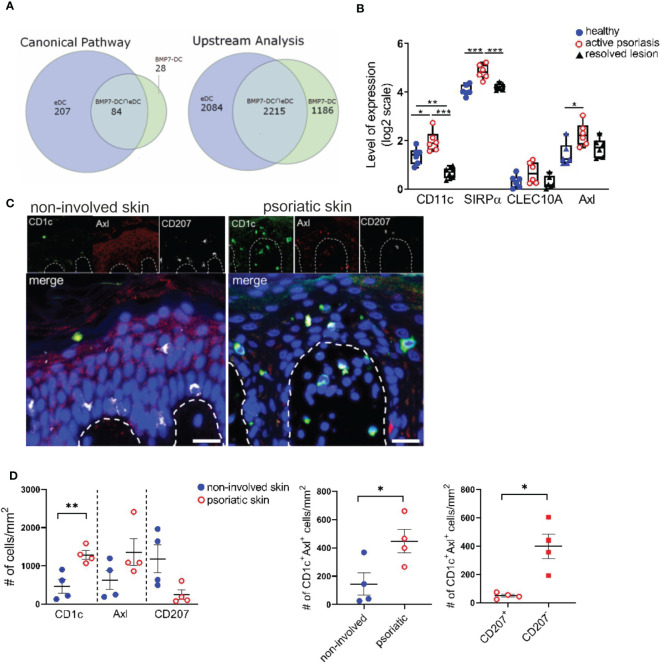
Identification of CD1c^+^Axl^+^ cells in the human psoriatic epidermis. **(A) **Venn diagrams illustrate the overlaps between Canonical Pathways and Upstream Regulators dissected by an IPA-based Core analysis on the basis of differentially expressed genes attributed to epidermal psoriatic DCs (eDCs), published by Cheng et al. (37) and CD34^+^ cell-derived CD1a^+^ BMP7-LC/DC. **(B)** Comparative analyses of mRNA expression levels of CD11c, SIRPα, CLEC10A and Axl between healthy, active psoriatic and resolved human epidermal skin. Expression values were extracted from the GENEVESTIGATOR platform (GSE103489 dataset). Box plots illustrate the differences in expression levels. Significant differences are indicated by asterisks *(*p* < 0.05), **(*p* < 0.01) and ***(*p* < 0.001) (2-tailed Student’s t-test). **(C)** Representative images of healthy and psoriatic epidermal skin section stained for CD1c (green), Axl (red), CD207 (white) and DAPI are shown upon automatic acquisition using the TissueFAXS platform. Scale bar = 20 µm. **(D)** Quantitative analysis of the Axl^+^CD1c^+^ immune cell populations based on a single cell recognition algorithm by TissueQuest software (TissueGnostics, Vienna, Austria). The epidermis was selected as region of interest. Graphs depict the cell density indicated as the numbers of positive cells per mm^2^ in healthy (n=4) and lesional (n = 4) skin biopsies for each indicated marker. Data are shown as mean ± SEM and were analyzed using 2-tailed Student’s t-test; significant differences are indicated by asterisks *(*p* < 0.05) and **(*p* < 0.01).

Above experiments revealed that anti-Axl stainings can be used to delineate Axl^+^cDC2 and to study their differentiation into LCs. BMP7 and p38MAPK promoted Axl^+^cDC2 at the expense of LCs. Previous studies showed p38MAPK activation in response to BMPR1A signaling in other cell types (48), opening the possibility that p38MAPK is activated downstream of BMP7 signaling in cDC2 differentiation. We next asked whether above-mentioned *in vivo* occurring psoriatic CD207^-^eDCs (37) exhibit molecular resemblance with activated (TLR2-stimulated) BMP7-driven LC/DC-like cells generated in cultures of CD34^+^ progenitor cells (40). As also shown for neo-appearing lesional epithelial DCs and LC-like cells, these latter cells expressed CD1a, lacked Birbeck granules, and exhibited a CD207^lo^CD1c^+^CD206^+^ marker profile (40). Data analysis via the Ingenuity Pathway Analysis platform indeed revealed that both cell fractions exhibit pronounced commonalities, as 75% of Canonical Pathways and 65% of Upstream Regulators described on the basis of BMP7-associated genes overlapped with the eDCs found in the psoriatic patients (**Figure 4A**). Among the common induced pathways, we identified IL-17A and ERK/MAPK signaling, both known to be fundamentally involved in atopic dermatitis and psoriasis (44, 49, 50). Subsequent IPA-based analysis identified TGF-β, BMP7, NOTCH1, p38MAPK/RelB and CDH1/CTNNB1 as common significant Upstream Regulators (**Figure 4A** and **Supplementary Tables 5–8**). Therefore, lesional CD207^-^eDCs indeed exhibit close resemblance with BMP7 driven CD207^lo^ LC/cDC2-like cells.

### 2.5 Identification of a spectrum of Axl^+^CD1c^+^ and CD207^+^LCs in the lesional psoriatic skin

In subsequent analyses we aimed to identify Axl^+^cDC2 within the epidermis of psoriatic cutaneous lesions. GENEVESTIGATOR- based comprehensive analysis of a publicly available transcriptomic dataset comprising healthy, psoriatic and resolved epidermal tissue (51) showed up-regulation of Axl, CD11c and SIRPα in the diseased skin sections, markers expressed by Axl^+^cDC2 (**Figure 4B**). We next performed quantitative immunofluorescence analysis of healthy and psoriatic human skin biopsies for CD207, CD1c and Axl using triple stainings. Being aware that epidermal keratinocytes of human healthy skin biopsies reportedly express Axl (29) and potentially gain low levels of CD1c under inflammatory conditions, only CD1c^hi^ cells were considered for the analysis. Only CD207^hi^ cells were considered as epidermal LCs. Transcriptomic analysis of single-cell RNAseq datasets (**Supplementary Figure 7A**) and total RNAseq datasets (**Supplementary Figure 7B**) of healthy skin sections revealed (i) higher Axl expression in DCs compared to epidermal keratinocytes and (ii) lack of CD1c in healthy keratinocytes. Indeed, computerized microscopy and the follow-up quantitative analysis revealed the presence of a spectrum of epidermal DC subsets in psoriatic human skin sections (**Figure 4C**). In the increased stratum spinosum, we localized two distinct cell types marked as CD207^+^CD1c^+^Axl^+^ (classical steady-state LC morphology) and CD207^low/-^CD1c^+^Axl^+^. Within the papillae, we found CD1c^+^Axl^+^CD207^+/-^ cells, which might translocate from the epidermal to the dermal region. In comparison, in healthy skin biopsies, we only occasionally found Axl^+^CD1c^+^CD207^-^ cells, and the majority of the star-like shaped CD207^hi^CD1c^-^Axl^+^ LCs resided in the epidermal stratum spinosum. Next, we performed statistical evaluation by comparative analysis using the densities of marker-positive cells as variables. Healthy (non-involved) and psoriatic skin samples were compared for CD1c, Axl and CD207. The CD1c^+^Axl^+^ double positive cell population was further assessed for co-expression of LC marker CD207. Overall, we found that the psoriatic skin harbours CD1c^+^Axl^+^ cells, the majority thereof lacking or only expressing low levels of LC-marker CD207 (**Figure 4D**). These observations are consistent with previously reported CD207^-^ eDCs exhibiting *Axl* mRNA expression and outnumbering LCs in the lesional psoriatic epidermis (16).

### 2.6 Non-canonical NFkB transcription factor RelB is induced in a subset of TGF-β1 stimulated cDC2s *in vitro*, and occurrence of RelB^+^ DCs in psoriatic lesions

**Figure 5:**
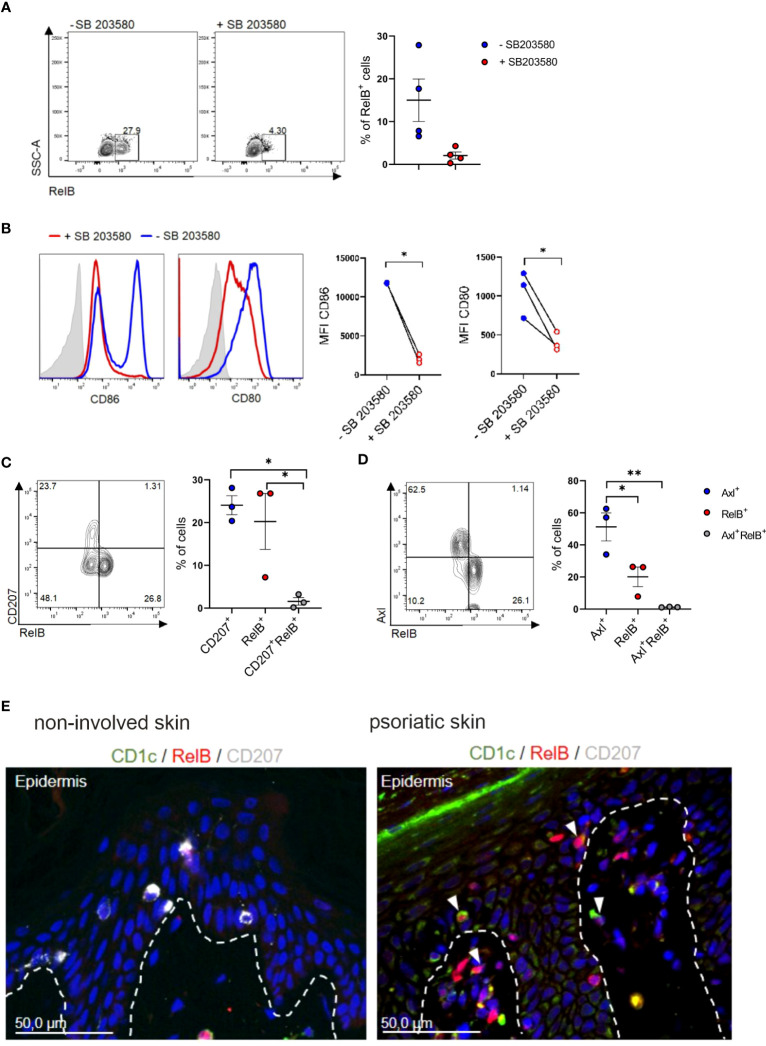
Identification of RelB^+^ cDC2s *in vitro* and *in vivo*. **(A) **TGF-β1 stimulated blood cDC2s were cultured in presence or absence of SB 203580 for 2 days and analyzed for intracellular RelB (n=4) and **(B)** surface expression of CD80 and CD86 [n=3, mean± SEM, paired 2-tailed Student’s t-test (*p<0.05)]. **(C)** Day 2 FACS analysis of TGF-β1 stimulated blood cDC2s for RelB vs CD207 or **(D)** RelB vs Axl [n=3, mean± SEM, one-way ANOVA (*p <0.05; **p <0.01)]. **(E)** Sections of non-involved and psoriatic human skin (n=4) analyzed for the expression of RelB (red), and CD207 (white) and CD1c (green). DAPI was used to visualize nuclei. The arrows indicate examples of cells double-positive for CD1c and RelB. Scale bar = 50 µm.

The non-classical NF-kB family member RelB was both implicated in p38MAPK signaling in DCs and in murine cDC2 differentiation. Specifically, RelB is induced downstream of p38MAPK signaling in CD1a^+^DCs/LCs in cultures of human progenitor cells (52), and is essential for myeloid-related DC differentiation in mouse and human [murine splenic CD4^+^ESAM^+^cDC2 (53) and CD11b^+^CD8α^-^DC (54); human monocyte-derived interstitial/dermal CD11b^+^DC (55)]. Conversely RelB is dispensable for LC differentiation, and epidermal LCs lack RelB (54, 55). Its positive effects on human interstitial/dermal-type DC differentiation was mediated via the promotion of monocyte intermediates pre-generated in cultures of human CD34^+^ hematopoietic progenitor cells (55). Our *in vitro* blood cDC2 differentiation cultures revealed that RelB is expressed by a portion of GM-CSF/TGF-β1 stimulated cDC2s in a p38MAPK dependent manner (**Figure 5A**), with p38MAPK inhibition resulting in diminished CD80 and CD86 expression (**Figure 5B**). RelB^+^ cells emerging in cultures of blood cDC2 in response to GM-CSF/TGF-β1 lacked both Axl and CD207 (**Figures 5C, D**). RelB stainings of the inflamed skin have to our knowledge not been reported previously. Immunohistology revealed numerous RelB^+^CD1c^+^ cells in the lesional psoriatic skin, but not in healthy control (**Figure 5E**), and these cells predominantly occurred in the dermal compartment (**Figure 5E**). In conclusion, RelB can be induced in blood cDC2s and these cells lack Axl and CD207. RelB^+^CD1c^+^ cells may therefore represent a distinct cDC2 differentiation pathway in the inflamed skin.

### 2.7 Axl^+^cDC2 to LC differentiation in cultures of human CD34^+^ hematopoietic progenitor cells

**Figure 6:**
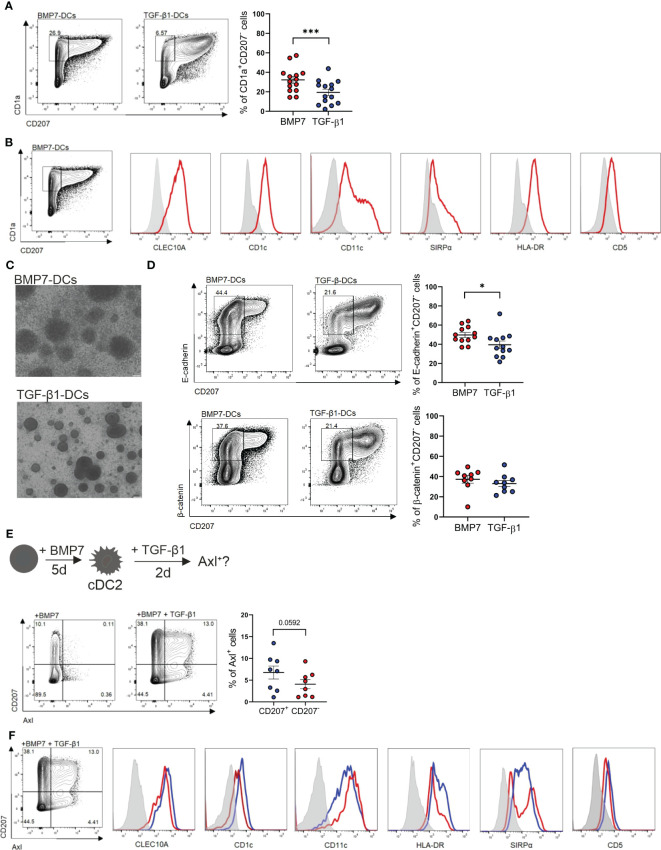
BMP7 promotes a cDC2 associated phenotype in CD34^+^ progenitor cells, which is repressed by TGF-β1/ALK5 signaling. **(A)** Human cord blood CD34^+^ progenitor cells cultured for 7 days in a basal DC differentiation cytokine mix (GM-CSF, TNFα, SCF, FLT3-L) in presence of BMP7 or TGF-β1. Representative FACS plot shows surface expression of CD1a versus CD207. The bar depicts the percentages (± SEM) of gated CD1a^+^CD207^-^ cells. The dots represent the number of replicates [n=14, mean± SEM, paired 2-tailed Student’s t-test (***p < 0.001)]. **(B)** CD34^+^ progenitor cells cultured in the presence of GM-CSF, TNFα, SCF, FLT3-L and BMP7 for 7 days. Representative flow cytometry histograms of gated CD1a^+^CD207^-^ cells for the indicated markers (n=3). **(C)** Cell cluster morphology of BMP7 and TGF-β1-DC cultures on day 7. Scale bar = 100 µm. **(D)** CD34^+^ progenitor cells were cultured as in **(A)** in presence of BMP7 or TGF-β1 for 7 days. Representative flow cytometry plot depicts pre-gated CD1a^+^CD207^-^ gated cells, analyzed for surface CD207 versus E-cadherin (n= 12) and CD207 versus β-catenin (n=9). Bars represent cells positive for the indicated markers [mean± SEM, paired 2-tailed Student’s t-test (*p <0.05)]. **(E)** Schematic representation of the experimental set-up of BMP7-cDC2-like differentiation cultures of CD34^+^ progenitor cells *in vitro*. CD34^+^ cells were differentiated into BMP7-cDC2-like cells for 5 days, followed by TGF-β1 stimulation for 2 days. Representative FACS plot shows CD207 versus Axl expression on day 7 [n=8, paired 2-tailed Student’s t-test (*p <0.05)]. **(F)** Representative flow cytometry analysis of cDC2 associated marker genes of Axl^+^CD1a^+^CD207^+^ and Axl^+^CD1a^+^CD207^-^ cells generated in the basal DC mix (GM-CSF, TNFα, SCF, FLT3-L) containing BMP7 plus TGF-β1 from CD34^+^ cells (n=3).

To further delineate the lineage relationship and signal requirement of above described epithelial Axl^+^DC/LC subsets, we utilized differentiation cultures of CD34^+^ hematopoietic progenitor/stem cells. cDC2-like cells can be generated *in vitro* in association with cDC1 and pDCs (56). However, co-generation of cDC2-like cells together with LCs has to our knowledge not been reported. TGF-β1 induces LC differentiation when added to GM-CSF plus FLT3-L and TNFα supplemented cultures of CD34^+^ HPCs. Adding BMP7 instead of TGF-β1 to these cultures also led to LC differentiation (43). These BMP7-LC generation cultures showed higher cellularity and more numerous large homotypic LC-type clusters than TGF-β1-LC generation cultures (43). mRNA profiling and subsequent FACS analysis revealed among others expression of the cDC2 markers CD1c and CLEC10A (12, 57) by CD207^+^ cells from BMP7-LC but not from TGF-β1-LC generation cultures (40). Above pathway analysis confirmed that psoriatic eDCs closely resemble BMP7-LCs (**Figure 4A**). Conversely LC-like cells generated in TGF-β1-LC cultures phenotypically resembled steady-state LCs (CD1c^-^CD207^hi^). Interestingly, CD1a^+^ cells lacking CD207 accumulated in BMP7-LC generation cultures, and were much less frequent in TGF-β1-LC generation cultures (**Figure 6A**) (40, 43). These cells remained unclassified. Flow cytometry analysis revealed that they exhibit all analyzed cDC2 marker characteristics (CLEC10A^+^CD1c^+^SIRPα^+^CD11c^+^HLA-DR^+^CD5^lo^; **Figure 6B**) (11, 12). We previously observed that CD1a^+^CD207^+^ BMP7-LCs exceed TGF-β1-LCs in their capacity to induce regulatory T cell generation from naïve allogeneic CD4^+^ T cells (39). A similar effect was seen when analyzing flow sorted CD1a^+^CD207^-^ cells [i.e. CD1a^+^CD207^-^ cells from BMP7-LC exceeding those from TGF-β1-LC cultures in regulatory T cell (Treg) induction (**Supplementary Figure 8**)]. Therefore, BMP7 in combination with additional cytokines (GM-CSF, FLT3-L and TNFα) co-induces cDC2- and LC-like cell generation from CD34^+^ HPCs, and these cells are potent inducers of Tregs, corroborating an anti-inflammatory effect of BMPR1a in murine CD11c^+^ DCs (39, 42). Considering their regulatory function and the presence of numerous large cell clusters in BMP7 cultures (**Figure 6C**), morphologically resembling previously described E-cadherin-dependent LC clusters in TGF-β1-LC generation cultures (58), we further asked whether CD1a^+^CD207^-^cDC2-like cells express E-cadherin and its intracellular binding partner β-catenin. We found high percentages of E-cadherin among CD207^-^ cells generated in BMP7 cultures, and these cells were more frequently observed in BMP7- than in TGF-β1-supplemented cultures. Moreover, BMP7 supplemented cultures showed in 6 of 9 independent experiments higher percentages of β-catenin compared to TGF-β-supplemented cells (**Figure 6D**). We further asked whether the *in vitro* generated cDC2-like cells may show TGF-β1-inducible Axl expression, as observed for blood cDC2s (**Figure 3A**). In these experiments (in **Figure 6E**) we first cultured progenitor cells in the presence of BMP7 (plus basic cytokines GM-CSF, TNFα, FLT3L and SCF), followed by TGF-β1 addition for additional 48 h. Indeed, a portion of day 5 generated cDC2-like cells in the presence of BMP7 acquired Axl in response to short term (48 h) TGF-β1 stimulation. Specifically, we observed the induction of two cell populations, i.e. CD207^+^Axl^+^ and CD207^-^Axl^+^ (**Figure 6E**), both exhibiting cDC2 marker characteristics (CLEC10A^+^CD1c^+^SIRPα^+^CD5^+/-^CD11c^+^HLA-DR^+^; **Figure 6F**). In subsequent experiments, we asked whether CD207^-^cDC2-like cells may differentiate into CD207^+^LCs, and whether canonical TGF-β1 signaling might repress cDC2 in favour of LC characteristics. Adding TGF-β1 to BMP7-cDC2 cultures indeed induced a phenotypic shift of CD1a^+^CD207^-^ to CD1a^hi^CD207^+^ cells (**Supplementary Figure 9A**) and slightly repressed cDC2 markers (CD1c, CLEC10A and SIRPα; **Supplementary Figure 9B**). Since TGF-β1 can signal through both ALK5 and ALK3/BMPR1a, we performed an inverse experiment in which we inhibited canonical ALK5 signaling in TGF-β1-supplemented LC generation cultures. Adding an ALK5 inhibitor promoted the expression of all three cDC2 markers analyzed (**Supplementary Figure 9C**), supporting that BMPR1a/ALK3 promotes, whereas canonical TGF-β1-ALK5 represses cDC2 marker characteristics. However, these data do not prove that TGF-β1 signaling inhibits cDC2 markers by CD207^+^CD1a^hi^ LCs.

### 2.8 Identification of a subset of blood cDC2s co-expressing Axl, CD5 and E-cadherin

**Figure 7:**
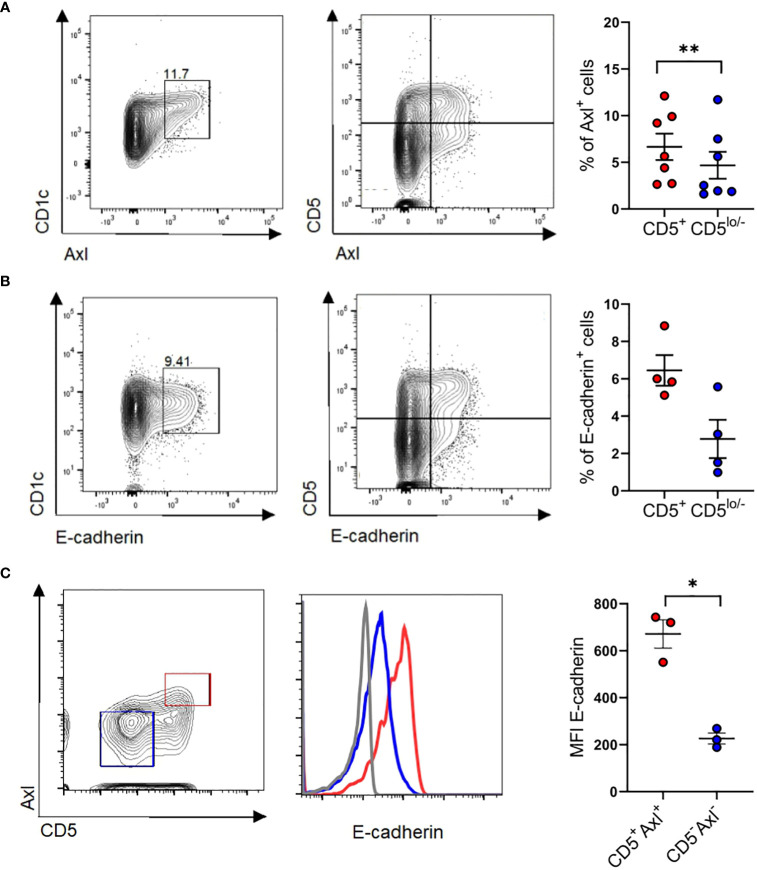
Identification of CD5^+^E-cadherin^+^ blood cDC2. **(A) **Purified blood cDC2s were analyzed for surface expression of Axl versus CD5 (n=7) and **(B)** E-cadherin vs CD5 (n=4). Graphs depict percentages of Axl^+^ and E-cadherin^+^ cells of CD5^+^ and CD5^low/-^ CD1c subpopulations (paired 2-tailed Student’s t-test (**p < 0.01). **(C)** Isolated blood cDC2 were gated for Axl^+^CD5^+^ and Axl^-^CD5^-^ subpopulations and analyzed for surface expression of E-cadherin (n=3, mean± SEM, paired 2-tailed Student’s t-test (*p <0.05). The isotype control staining is shown in **Supplementary Figure 10**.

Axl is constitutively expressed by a subset of peripheral blood CD1c^+^ cDC2s. Given that above *in vitro* generated cDC2-like cells co-express E-cadherin and Axl (**Figures 6D, E**), we asked whether E-cadherin is similarly expressed by Axl^+^ blood cDC2s. We confirmed that purified CD1c^+^ DCs express cDC2 associated markers CLEC10A, CD1c, SIRPα with a subset being Axl^+^ (10, 11) (**Figure 7A**). E-cadherin can indeed be detected on a subset of blood cDC2s and these cells were enriched among the CD5^+^ cell subset (9) (**Figure 7B**), and overlapped with Axl^+^ cells (**Figure 7C**).

## 3 Discussion

We here demonstrated that peripheral blood cDC2s lack the monocyte identity transcription factor KLF4, consistent with their non-monocyte origination. However, they can be induced to acquire high levels of KLF4 along with macrophage and moDC phenotypic characteristics *in vitro.* Moreover, a subset of cDC2 acquired RelB, previously shown to be essential for human monocyte and interstitial/dermal DC development (55). These RelB^+^CD1c^+^ cells predominantly occurred in the dermal compartment of cutaneous psoriatic lesions. In addition, we delineated a second, predominantly epidermal cDC2 differentiation pathway, leading to the development of LCs, another cell type classified as being monocyte-derived. We demonstrated that BMP7, strongly induced during inflammation, promotes the generation of Axl^+^cDC2s from blood cDC2s, and that these cells can further differentiate into LCs. These BMP7-driven epithelial cDC2s possessed pronounced Treg inducing capability, and we demonstrated that their differentiation can be modelled using cultures of CD34^+^ hematopoietic progenitor cells. In conclusion, cDC2s can gain characteristics of three cell types previously classified as being monocyte-derived, i.e. KLF4^hi^CD14^+^CD11b^+^ macrophages, as well as moDCs/dermal DCs and LCs (1). Sub-sorting of cDC2 into CD5^+^CD14^-^ and CD5^-^CD14^dim^ subsets confirmed that both are capable of differentiating into monocyte-derived cells. Therefore, despite blood cDC2 and monocytes arise via separate pathways from hematopoietic stem cells, they can “transdifferentiate” into monocyte-derived cells in response to inflammatory signals.

Our notion that blood cDC2s can be induced to differentiate into monocyte-derived cells (i.e. CD1a^+^CD11b^+^CD209^+^ moDCs and CD14^+^CD11b^+^ macrophages) is supported by transcription factor analysis. KLF4 is a master transcription factor of monocytopoiesis and proved to represent a reliable lineage marker for human monocytes in clinical haematology (30–32). High levels of KLF4 protein expression observed in moDCs and macrophages generated from blood cDC2s therefore support their monocyte-derived cell identity. Additionally, we showed that blood cDC2s can differentiate into RelB^+^ cells *in vitro*. Our previous demonstration that RelB promotes the generation of CD11b^+^CD14^+^ monocyte intermediates of intestinal/dermal DC from human CD34^+^ hematopoietic progenitor cells (55) is consistent with the here observed monocyte reprogramming of cDC2s. Corroborating these findings, CD1c^+^RelB^+^ cells predominantly occurred in the dermis of cutaneous psoriatic lesions and was rarely detected in non-lesional skin. Moreover, *in vitro* generated blood cDC2-derived RelB^+^ cells were distinct from co-generated Axl^+^CD207^-^ and Axl^+^CD207^+^ cells. This finding is consistent with our previous demonstration that RelB fails to promote LC differentiation (55). Similar to RelB, KLF4 promoted CD11b^+^CD14^+^ cell differentiation (30), and we previously reported that retroviral inducible ectopic KLF4 in monocyte progenitor cells interferes with TGF-β1/RUNX3-dependent LC differentiation of monocytopoietic cells. Consistently, short term pre-stimulation of blood cDC2s under moDC differentiation conditions (accompanied by KLF4 induction) led to a loss in LC differentiation potential. Additionally, dermal DCs but not LCs expressed detectable KLF4 protein (34). In conclusion, transcriptional regulators RelB and KLF4 promote monocyte differentiation from human hematopoietic progenitor cells and both may cooperatively induce moDCs/dermal DCs from blood cDC2 in response to inflammatory signals.

A key finding of our study was that anti-Axl stainings enable to delineate a predominantly epidermal pathway of cDC2 to LC differentiation. Using two independent *in vitro* differentiation culture models (i.e. initiated by blood cDC2 or CD34^+^ hematopoietic progenitor cells) we showed that BMP7 plus TGF-β1 provide critical co-signals for Axl^+^cDC2 differentiation. Specifically, canonical TGF-β1-SMAD-2/3 signaling induces Axl by cDC2s. Conversely, BMP7/BMPR1a signaling promotes the accumulation of cDC2, and these cells further differentiated into Axl^+^LCs in response to diminished BMPR1a or p38MAPK signaling. *In vivo* data are consistent with this model. First, the lesional enlarged psoriatic epidermis is marked by high levels of BMP7 throughout all keratinocyte layers, and here presented Ingenuity Pathway Analysis showed that lesional epithelial DCs, eDCs (16), exhibit a BMP7/BMPR1a-p38MAPK signature. Conversely, BMP7 is restricted to the basal/germinal keratinocyte layer in the healthy skin, and we previously showed that topical treatment of psoriatic lesions results in reduced BMP7 expression similar to healthy epidermis (39). Second, TGF-β1-SMAD2/3 signaling is constitutively activated at equivalent levels in the normal and psoriatic lesional epidermis. Consistently, we demonstrate here that canonical TGF-β1 signaling is required for Axl induction by cDC2 and that eDCs showed TGF-β1 pathway activation. Therefore, both TGF-β1 and BMP7 seem to cooperate in Axl^+^cDC2 differentiation and accumulation, and Axl^+^cDC2 to LC differentiation may be triggered by the loss of BMP7 expression marking the resolution phase of psoriatic lesions.

We here described a new differentiation culture model for the *in vitro* generation of cDC2-like cells from human CD34^+^ hematopoietic progenitor cells. Previous cell culture systems generated cDC2s along with cDC1 and plasmacytoid DCs, but not with LCs as reported in our study (reviewed in: (59). Notably, unlike previously pusblished protocols, our optimized cDC2-like cell generation system is devoid of serum/plasma and stroma cells and is of particular advantage for cell therapy-oriented studies. Moreover, we demonstrated that cDC2s expressed E-cadherin and form large cell clusters allowing for cell enrichment using a simple 1 x g cluster sedimentation step as previously described for LC-like cells (60). Additionally, we described specific culture conditions for cDC2 generation and for their further differentiation into LCs.

We show that the *in vitro* generated BMP7-BMPR1a-driven cDC2s possess potent capacity to induce FoxP3^+^ regulatory T cells. These data are reminiscent of our recent study showing that the deletion of BMPR1a in murine CD11c^+^ cells leads to defective resolution of allergic and psoriatic skin inflammation (42). Murine psoriatic cutaneous lesions are marked by similar strong epidermal BMP7 expression as observed for human lesions (40), supporting this notion. Interestingly, regulatory Axl^+^cDCs were also described in murine and human carcinomas (15), and BMP7 densities by carcinoma cells positively correlate with disease severity and progression in patients access several carcinoma entities (61–66). Therefore, the here observed BMP7-driven Axl^+^cDC2 accumulation in the enlarged psoriatic skin might be of relevance for DC-mediated carcinoma immune evasion. In both epithelial tissues, Axl might be induced by canonical TGF-β1 signaling. Consequently, our previous demonstration that Axl mediates TGF-β1-dependent enhanced efferocytosis by macrophages (29) might be of relevance to tolerogenic DC programming in carcinomas (15). Additionally, we here described that Axl^+^cDC2s express E-cadherin and β-catenin, two interacting molecules previously linked to DC-mediated tolerance (67, 68), and previous studies demonstrated that BMP7 promotes PDL2 expression by DCs (39, 69).

We here detected E-cadherin expression on a subset of blood cDC2s and showed that these cells overlap with previously described Axl^+^CD5^+^ ASDCs. Moreover, the weak constitutive Axl expression by blood ASDC was inhibited by abrogation of canonical TGF-β1 signaling. This is in line with the previously reported constitutive high TGF-β expression by blood cDC2 (41), and with the here described absence of KLF4, also known for its inhibition of TGF-β1 signaling (30, 34, 70). Our short-term cultures revealed that Axl and E-cadherin can be neo-induced by a large portion of blood cDC2, indicating that most cDC2s possess the capacity to differentiate into Axl^+^cDC2s upon immigration into epithelial/epidermal tissues. It will be interesting to quantify E-cadherin^+^Axl^+^CD5^+^ cDC2s in the blood of psoriasis and carcinoma patients. Subsets of blood cDC2s might be derived from E-cadherin^+^Axl^+^ tissue-resident cDC2s in diseased epithelial tissues; alternatively, they might represent precursors of tissue-associated cDC2s. Consistent with the latter possibility, murine oral mucosal LCs (unlike epidermal LCs) are replenished from blood cDCs in the steady-state (24). CD5^+^cDC2 accumulate in inflamed psoriatic lesions (71) and their diminished frequency in draining lymph nodes of cancer patients correlated with poor prognosis and response to checkpoint inhibitor therapy (72). E-cadherin versus CD5 versus Axl expression may lead to the identification of new functional subsets of cDC2. E-cadherin/β-catenin expression in cDC2 might be invovled in their migration and function. Single cell analyses can be performed on DCs migrating from psoriatic skin (73) and carcinomas (72), facilitating detailed cDC2 subset analysis.

Cultured cDC2 do not show signs of proliferation, and we restricted culture periods of *ex vivo* isolated cDC2s to a maximum of 5 days. However, despite microscopic inspection of viability, a limitation of our study is that we can not exclude the possibility of cell death of portions or subsets of cDC2s under the various culture conditions. The CD207^+^ cells generated from cDC2s in the presence of GM-CSF and TGF-β1 retained cDC2 markers CD1c, CLEC10A and SIRPα. These cells are phenotypically similar to CD207^+^ cells neo-appearing in the lesional psoriatic epidermis (16, 40). Whether these day 5 generated cells may lose cDC2 markers upon prolonged TGF-β1 stimulation remains to be analyzed. Our data from the CD34^+^ cell-derived DC2 generation model indicate that canonical TGF-β1 signaling may repress cDC2 marker characteristics by cultured cells. However, additional experiments are needed to analyse whether cDC2 can differentiate into steady-state-like LCs or recently characterized LC2s (19). The majority of Axl^+^CD1c^+^ cells expressed CD207 at a low level. Given that we also detected co-localization of Axl and CD1c in the dermis and pre-dominantly at the dermal-epidermal border, it is interesting to investigate whether these cells originate from the dermis or epidermis.

**Figure 8:**
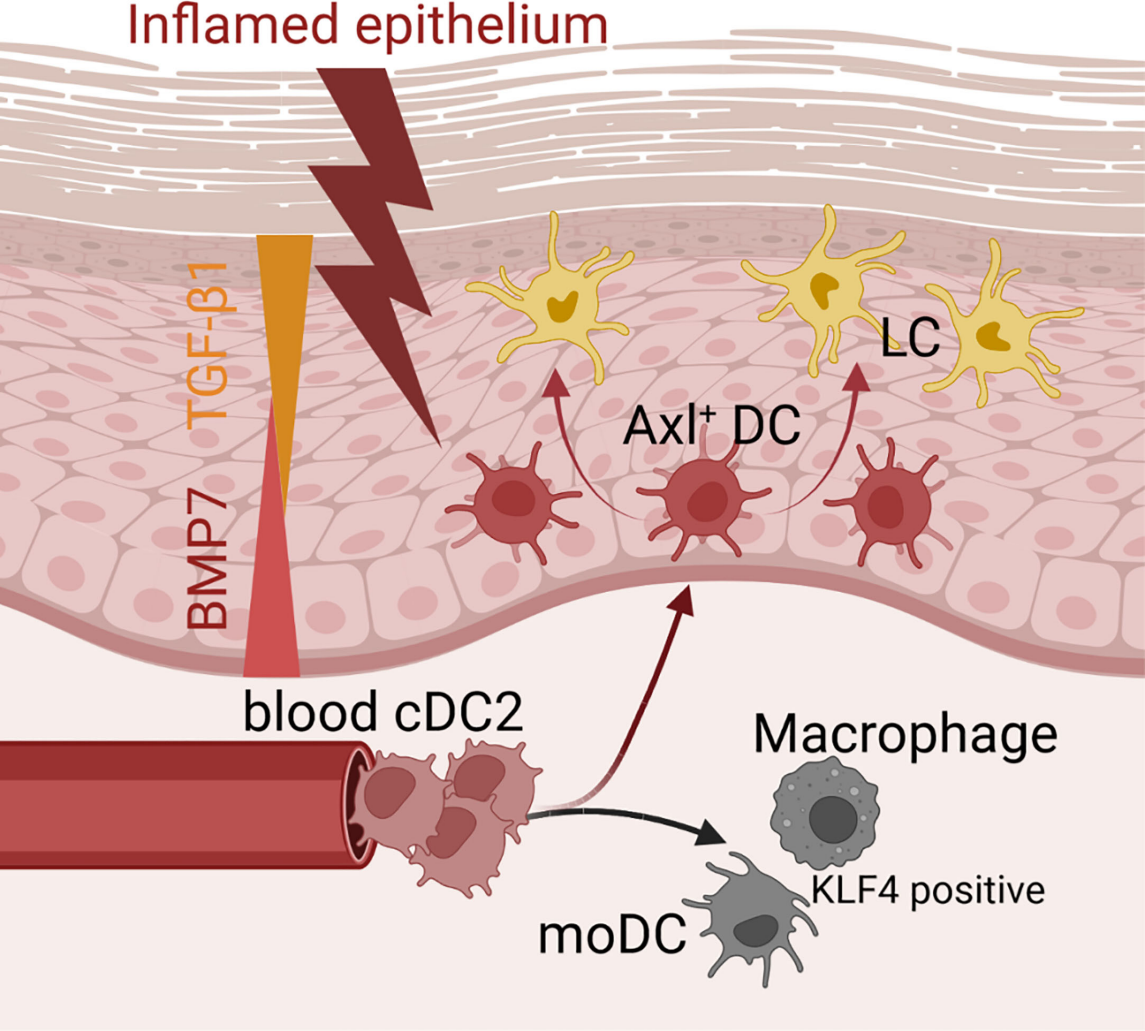
Microenvironmental signals directing the cDC2 phenotype. Schematic representation of how microenvironmental signals modulate phenotypic characteristics of cDC2. While epithelial signals such as TGF-β ligands lead to the promotion of Axl^+^cDC2 and LCs, pro-inflammatory stimuli convert cDC2 to gain moDC or macrophage characteristics.

In conclusion, our data revealed that inflammatory signals induce human cDC2s to differentiate into several cell subsets found in the lesion psoriatic skin, and they support a model whereby canonical pathways of hematopoiesis can be altered during inflammation (**Figure 8**). The here delineated roles of canonical TGF-β1 versus BMP receptor signaling in Axl^+^cDC2 accumulation versus further differentiation into LCs, may be of relevance for future immunotherapy-oriented studies in psoriasis and cancer. Additionally, we demonstrated here serum-free cytokine dependent culture conditions for the directed generation of cDC2s from human hematopoietic progenitor cells, which may facilitate their use in cell therapy.

## 4 Materials and methods

### 4.1 Human cell isolation and *in vitro* differentiation model

Umbilical cord blood was obtained from healthy donors during full-term delivery within the ethical approval (EK 26-520 ex 13/14) obtained from the Medical University of Graz. The isolation of CD34^+^ hematopoietic progenitor cells was performed by magnetic sorting with the EasySep human CD34 positive selection kit (Stem Cell Technologies) according to manufacturer’s instructions. Isolated CD34^+^ cells were pre-expanded in serum-free X-VIVO15 (Lonza) medium, in the presence of 50 ng/mL SCF, 50 ng/mL FLT3-L and 50 ng/ml TPO for 3-4 days. For subsequent LC differentiation, 5x10^4^ pre-expanded CD34^+^ cells were cultured in 24-well tissue culture plates in serum-free CellGro GMP DC medium (CellGenix, Freiburg, Germany) supplemented with 100 ng/mL GM-CSF, 2.5 ng/mL TNFα, 50 ng/mL FLT3-L, 20 ng/mL SCF, and 1 ng/mL TGF-β1 or 200 ng/mL BMP7 for 7 days. On day 3, fresh BMP7 or TGF-β1 was added. Monocytes were isolated using the CD14 MicroBeads (Miltenyi). For CD1c^+^ DC culture, PBMCs of two donors were pooled and cells were isolated using the human CD1c (BDCA-1)^+^ Dendritic Cell Isolation Kit (Miltenyi) according to manufacturer’s protocol. The purity of all isolated fractions was analyzed by flow cytometry. For DC generation, purified cells were stimulated with 100 ng/mL GM-CSF and 35 ng/mL IL-4. Macrophage differentiation was induced by the addition of 100 ng/mL M-CSF and 2 ng/mL IL-6, LCs were generated from purified CD1c^+^ blood DCs in response to 100 ng/mL GM-CSF and 1 ng/mL TGF-β1 or 200 ng/mL BMP7. FACS analysis was performed after 5-6 days. These cell culture experiments were performed in RPMI 1640 media supplemented with 10% FBS, 2.5 mmol/L GlutaMAX (Invitrogen, Grand Island, NY) and 125 U/ml penicillin/streptomycin (PAA, Pasching, Austria). Cytokines and reagents are listed in **Supplementary Table 2**. Inhibitors for BMP (dorsomorphin), classical TGF-β1 (SB431542), and p38MAK (SB203580) signaling were titrated to secure viability of cultured cells (the chosen concentrations are indicated in **Supplementary Figure 5**).

### 4.2 Mixed leukocyte reaction

The mixed leukocyte reaction assay was conducted as previously described (39). Day 7 generated CD1a^+^CD207^-^ cells of CD34^+^ cell-derived TGF-β1 and BMP7-LC cultures were enriched by magnetic sorting. 2.7x10^4^ sorted cells were co-cultured with 1x10^5^ purified allogeneic CD4^+^CD45RA^+^ naive T cells in triplicates. The assay was performed using RPMI 1640 media supplemented with 10% FBS, 2.5 mmol/L GlutaMAX (Invitrogen, Grand Island, NY) and 125 U/ml penicillin/streptomycin (PAA, Pasching, Austria). The cells were analyzed on day 5 by flow cytometry.

### 4.3 Flow cytometry

The viability of the cells was critically assessed by microscopy. Only samples in which the cells looked healthy were used for further analysis. For flow cytometry analysis, cells were stained and analyzed as previously described (39, 40). In brief, cells were harvested and washed with PBS. Prior to antibody staining, Fc receptors were blocked. For intracellular Treg and β-catenin stainings the FoxP3 staining buffer set (ThermoFisher, Waltham, Mass) was used. Intracellular RelB was analyzed using the FIX&PERM kit (Nordic MUbio, Susteren, the Netherlands) according to the manufacturer’s instructions. All samples were recorded using the LSRFortessa (BD Biosciences) and analyzed with the FlowJo software. All antibodies used are listed in **Supplementary Table 3**.

### 4.4 Human tissue samples

Paraffin-embedded biopsies were provided by the department of Dermatology, Medical University of Graz. The study was approved by the Ethical Committee of the State of Carinthia, Austria (Dithranol study ClinicalTrials.gov no. NCT02752672; approval number A23/15). Informed consent was provided to all patients in accordance with the Declaration of Helsinki.

### 4.5 Immunofluorescent staining and tissue image cytometry

For cell staining a polyclonal rabbit antiactivated Notch-1 (Abcam, Cambridge, United Kingdom) antibody, a polyclonal rabbit anti-KLF4 (Sigma-Aldrich, Cat#HPA002926) antibody and a p-SMAD2/3 (Cell Signaling Technology, Cat#8828) antibody were used.

Tissues were stained using pAb anti-BMP7 (LSBio, Cat# LS-B4567-50), pAb anti-pSMAD1/5/8 (Cell Signaling Technology, Cat# 9516), anti-TGF-β1 (Novus, Cat# NBP2-22114), anti-pSMAD3 (Abcam, Cat# ab118825) and anti-RelB (Cell Signaling Technology, Cat# 10544). Goat anti-Rabbit-Cy3 (Jackson ImmunoResearch Labs, Cat# 111-165-144) and horse anti-Mouse-Dy488 (Vector Laboratories, Cat# DI-2488) were used as secondary antibodies. Nuclei were counterstained with DAPI (Molecular Probes). Pictures were taken by the Leica DM4000B microscope and ZEISS LSM700 confocal microscope (Carl Zeiss Microscopy, White Plains, NY) and further processed using Adobe Photoshop CS5.

For analyzing CD1c, Axl and CD207 co-localisation in human skin sections, TissueFAXS platform (TissueGnostics, Vienna, Austria) was used for acquisition and quantitative analysis. Paraffin-embedded 4 µm sections of healthy (n=4) and active psoriatic (n=4) tissue sections were used for stainings. For the detection of CD1c, a monoclonal mouse antibody (Abcam, Cat# ab156708) was used followed by a donkey anti-mouse AF488-conjugated antibody (Jackson ImmunoResearch Labs, Cat# 715-545-150). Axl was detected by a rabbit antibody (Cell Signaling Technology, Cat# 4977), followed by a donkey anti-rabbit Cy3-conjugated antibody (Jackson ImmunoResearch Labs, Cat# 711-165-152). A rat antibody was used to detect CD207 (Eurobio Scientific, Cat#DDX0362B), followed by a donkey anti-rat AF647-labelled secondary antibody (Jackson ImmunoResearch Labs, Cat# 712-605-153). IgG rabbit (Calbiochem, Cat#N101), IgG mouse (Millipore Corporation, Temecula, Ca, USA, #N103) and IgG2a rat (Thermo Fisher Scientific, Cat# 14-4321-81) were used for isotype control. Nuclei counterstaining was performed using DAPI. All tissue slides were mounted using Dako Fluorescence Mounting Medium (Agilent, Inc, Santa Clara, California) and stored at -20°C until acquisition. Whole skin sections were recorded in the DAPI (nuclei), TRITC (Axl), GFP (CD1c) and Cy5 (CD207) channels using the 20x objective by automated tissue FAXS system. Quantitative analysis was performed using the TissueQuest software (version 7.1.1.123) (TissueGnostics, Vienna, Austria). The epidermis was selected as region of interest (ROI) and considered for quantitative analysis. Areas of parakeratosis, staining artefacts and high auto-fluorescence regions were excluded from the analysis. Patient-specific thresholds on scatter plots for detection of marker-positive cells were determined by three independent and experienced observers. Additionally, isotype staining of healthy and psoriatic skin biopsies were used as controls. Using TissueQuest software, for each patient the density values of marker-positive cells were exported and statistically analyzed using GraphPad Prism 5.0.0.286.

### 4.6 Compendium-wide analysis of transcriptomic datasets using GENEVESTIGATOR

GENEVESTIGATOR is a platform which comprises a compendium of publicly available and curated microarray, mRNASeq, and single-cell Seq datasets. The power of GENEVESTIGATOR alone and in combination with tissue image cytometry by TissueFAXS was previously shown by us (74–77). Four different datasets (GSE118165: n_total_=157; GSE115736: n_total_=42; GSE75042: n_total_=9; GSE107011: n_total_=127) of the mRNA Seq Gene Level Homo sapiens (Ref: Ensembl 97, GRCh38.12) platform were analyzed. From the multitude of cell types, datasets attributed to cDCs and CD14 monocytes (n=28) were filtered. Expression values (log2) of selected genes of interest were extracted for each sample and used for follow-up statistical analysis. Statistical significance was determined, and graphical visualisation was achieved using GraphPad Prism 5.0.0.286.

Expression values (log2) of human healthy and psoriatic epidermis (GSE103489) (**Figure 4B**) and healthy human skin [total RNA-seq Gene Level Homo sapiens GSE115898/10x scRNA-Seq Gene Level Homo sapiens (GSE147424)] (**Supplementary Figures 7A, B**) for the genes of interest were exported from GENEVESTGATOR and analyzed using GraphPad Prism 5.0.0.286.

### 4.7 Pathway analysis using the ingenuity pathway analysis tool

The IPA platform was used to assign differentially expressed genes to common biologically relevant Canonical Pathways and Upstream Regulators. The analysis was based on genes attributed to DCs, found in the psoriatic epidermis (37) and BMP7-LC, specified above. Gene names and the corresponding fold changes (FC) of the DC profile and differentially expressed genes in BMP7-LCs versus TGF-β1-LCs (FC > 1.5) were used for IPA-based Core analysis. Only statistically significant (p-value < 0.05) Canonical Pathways and Upstream Regulators were exported. The ranking is based on the p-value. For identifying overlaps between two datasets VENNY2.1.0 was used. Graphical visualisation was performed by use of the R package “ggplot”.

### 4.8 Statistical analysis

Statistical analysis was performed using GraphPad Prism Version 8.3.0 and Version 5.0.0.286. For the comparison of two groups Student’s two-tailed t-test was used, for the analysis of multiple groups a one-way ANOVA was performed. *p* values < 0.05 were considered as significant. All data are represented as means ± SEM.

## Data availability statement

The original contributions presented in the study are included in the article/**Supplementary Material**. Further inquiries can be directed to the corresponding author.

## Ethics statement

This study used human skin and human cord blood. Human skin: approved by Ethical Committee of the State of Carinthia. Cord blood: Ethical approval obtained from the Medical University of Graz. The patients/participants provided their written informed consent to participate in this study.

## Author contributions

ML and HS designed the concept of the study and wrote the manuscript; ML, CK, ES, CP, DM, AM, and SC contributed to data acquisition and analysis; CT-A performed histology staining; DM and AM supported in tissue image cytometry and in transcriptomics data as well as pathway analysis. All authors contributed to the article and approved the submitted version.
