# Supplementary material for: Microenvironmental and cell intrinsic factors governing human cDC2 differentiation and monocyte reprogramming
Source: Front Immunol. 2023 Jul 19;14:1216352. doi: 10.3389/fimmu.2023.1216352 (PMC10395083; doi:10.3389/fimmu.2023.1216352)
Supplement: Supplementary file 1 [file DataSheet_1.pdf]

Supplementary Material

TABLE OF CONTENTS

Supplementary Figures S1-S10

Supplementary Tables S1-S8

SUPPLEMENTARY DATA

Supplementary Figures

Supplementary Figure 1

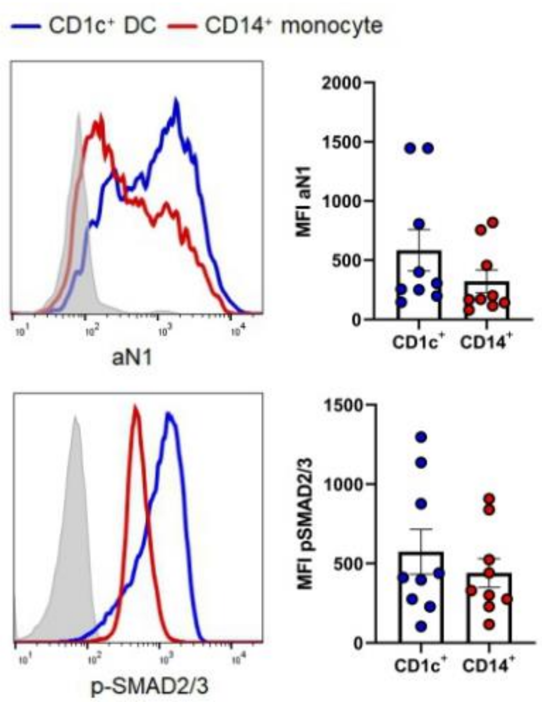

Fig.S1. Flow cytometric profile of p-SMAD2/3 and aN1.

Flow cytometric staining of CD1c$^+$ DCs and CD14$^+$ monocytes for intracellular p-SMAD2/3 and active Notch-1 (aN1). Graph depicts analysis of p-SMAD2/3 and aN1 of CD1c$^+$ DCs and CD14$^+$ monocytes (n=9, 2-tailed Student *t* test)

Supplementary Figure 2

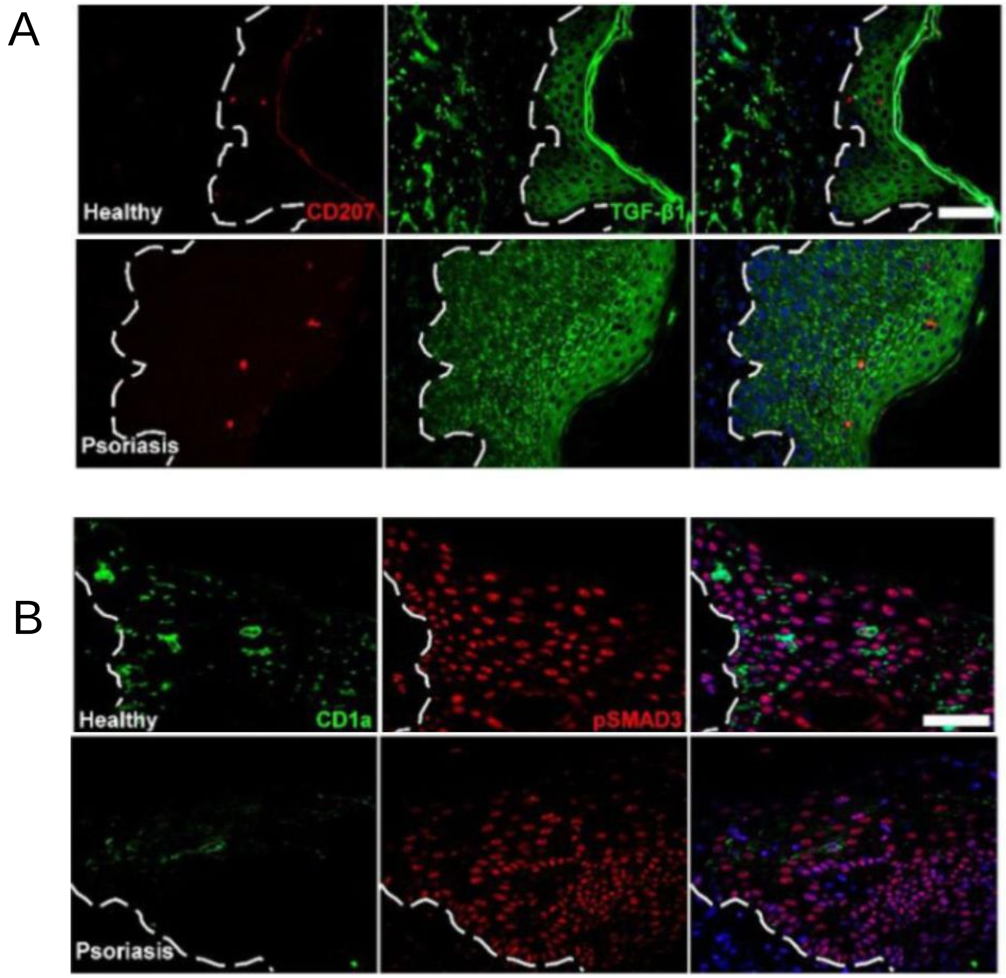

Fig.S2. Human healthy and psoriatic epidermal skin equally express TGF-β1 and pSMAD3. Representative immunofluorescence staining of human healthy vs lesional psoriatic skin for **(A)** CD207 and TGF-β1 and **(B)** CD1a and pSMAD3

Supplementary Figure 3

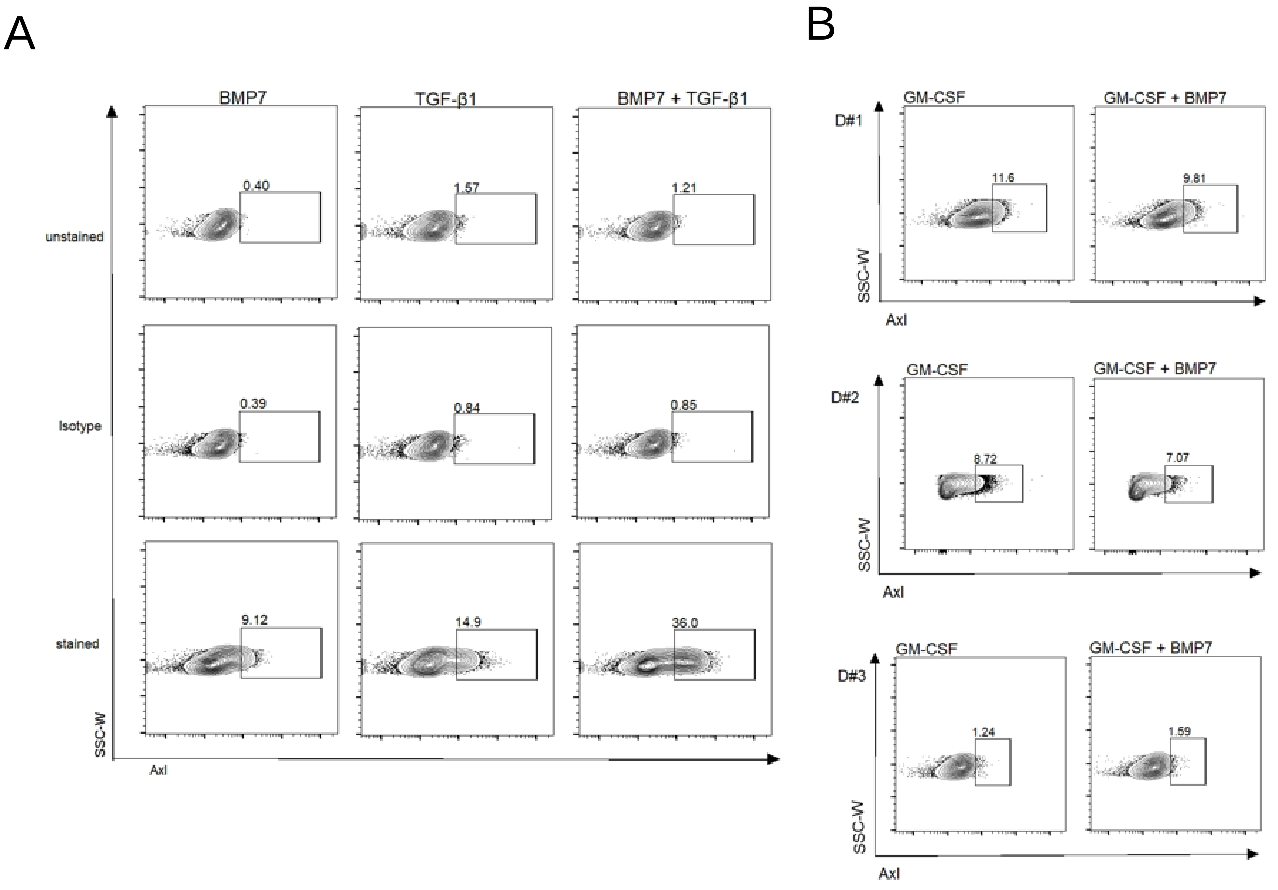

Fig.S3. Gating strategy for Axl expression. (A) Blood cDC2s were cultured in presence of TGF-β ligands (BMP7, TGF-β1, BMP7+TGF-β1) and analyzed on day 5. Representative graph shows unstained and isotype control staining of surface Axl expression for all conditions. **(B)** Day 5 GM-CSF or GM-CSF+BMP7 stimulated blood cDC2s were analyzed for Axl expression across 3 independent experiments.

Supplementary Figure 4

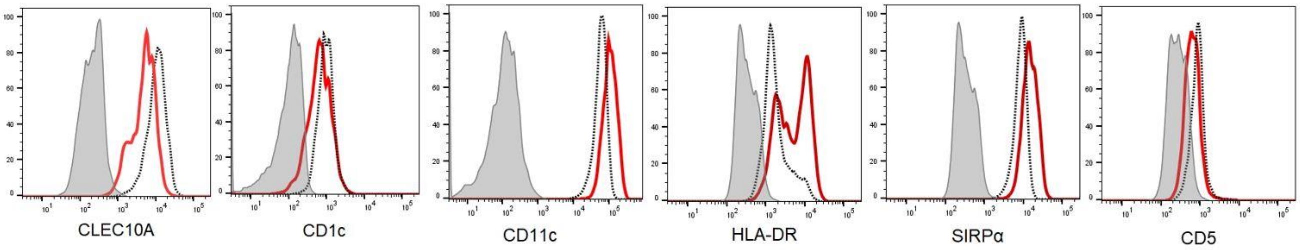

Fig.S4. Expression of cDC2 markers by Axl$^+$DCs an LCs. Phenotypic analysis of Axl$^+$ DCs compared with blood cDC2 derived TGF-β1-LCs. Red line, Axl$^+$CD207$^-$ cells derived from BMP7+TGF-β1 cultures; dotted black line, Axl$^+$CD207$^+$ cells of TGF-β1 culture (n=3).

Supplementary Figure 5

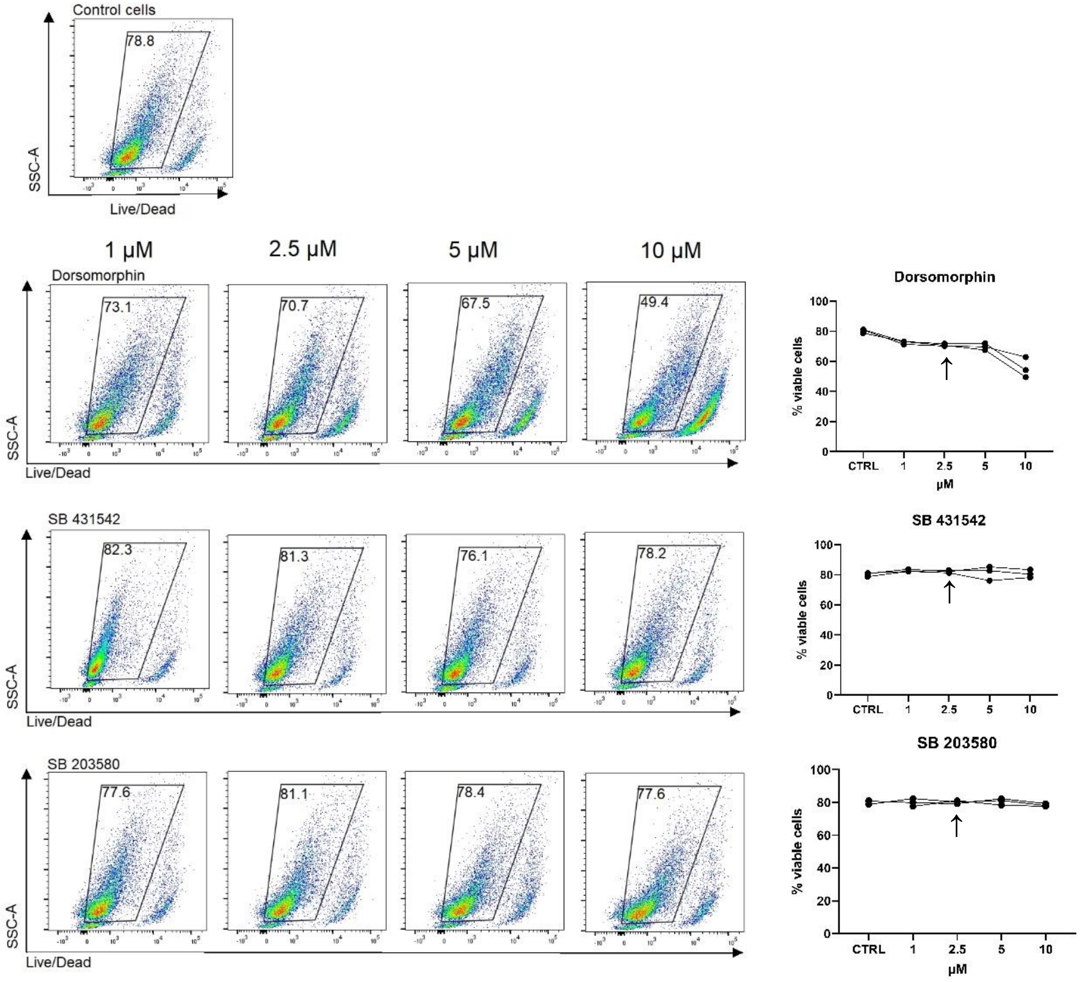

Fig.S5. Inhibitor cytotoxicity assessment on primary cells. Human primary cells were treated with Dorsomorphin, SB431542 or SB203580 in different concentrations ranging from 1-10 µM. Viability of 3 independent donors was measured after 3 days of stimulation. A concentration of 2.5 µM of all inhibitors was used.

Supplementary Figure 6

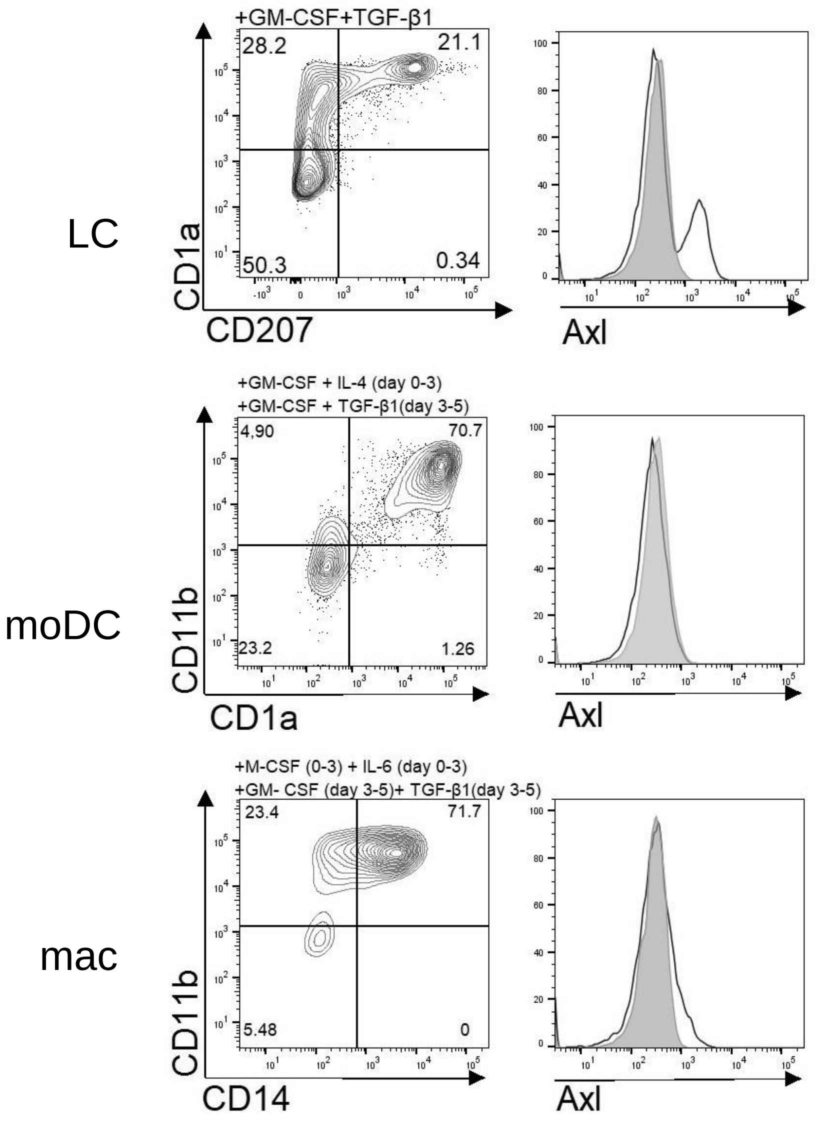

Fig.S6. cDC2 lose their potential to gain Axl expression after stimulation under moDC/mac conditions.
CD1c^{+} DCs were cultured under moDC (GM-CSF/IL-4) or macrophage (M-CSF/IL-6) differentiation condition. On day 3, cells were sub-cultured in presence of GM-CSF/TGF-β1 for 2 days and Axl expression was analyzed on day 5. CD1c^{+} DCs stimulated under LC promoting conditions (GM-CSF/TGF-β1) were used as control (n=2).

Supplementary Figure 7

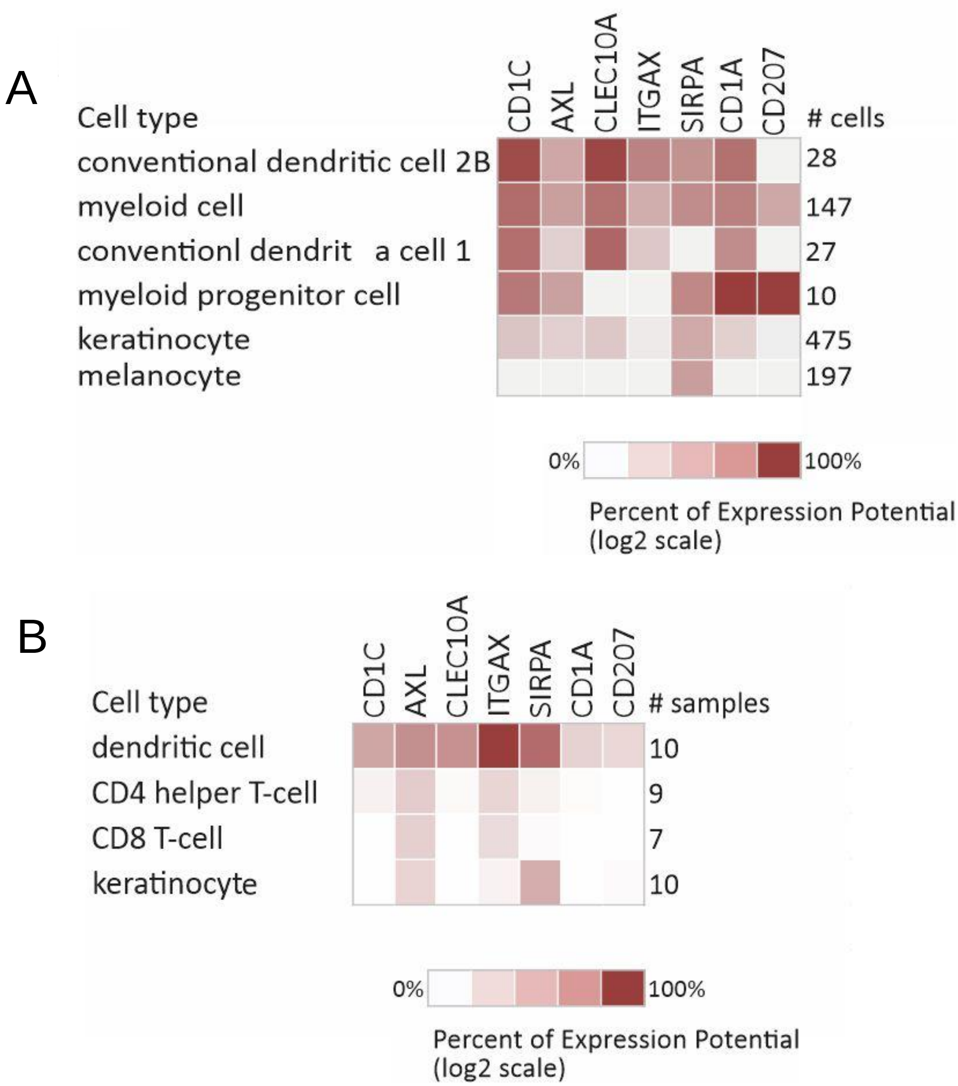

Fig.S7. Upregulation of cDC2 markers and Axl in the human psoriatic skin. GENEVESTIGATOR-based compendium-wide analysis of the expression pattern across all epidermal cell types of the healthy human skin using **(A)** single-cell RNAseq datasets and **(B)** total RNAseq datasets.

Supplementary Figure 8

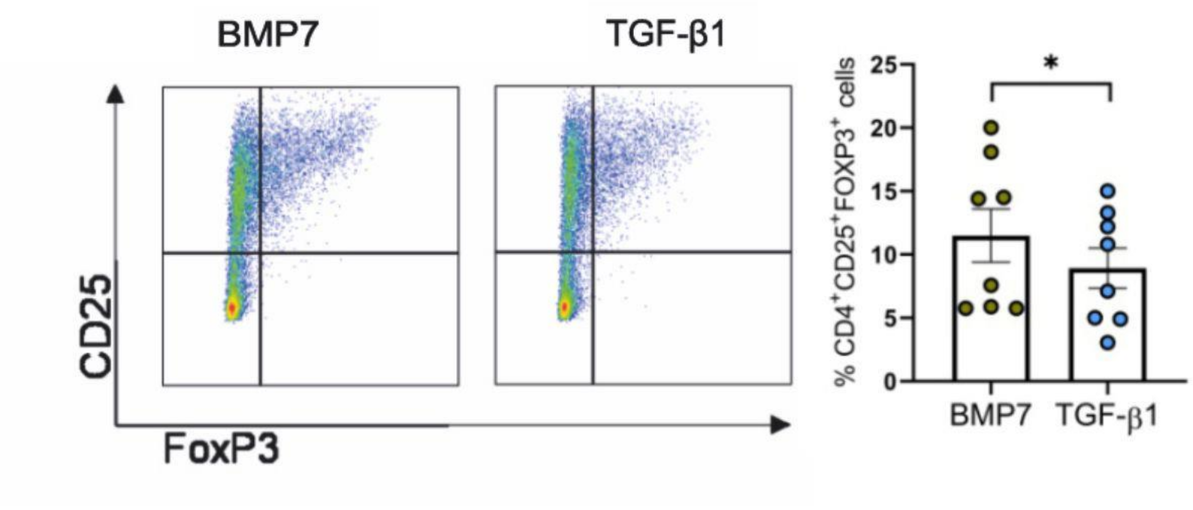

Fig.S8. BMP7-DCs exceed TGF-β1-DCs in their Treg cell stimulatory capacity.
MACS sorted BMP7-DCs or TGF-β1-DCs were co-cultured with naïve $CD4^+CD45RA^+$ T cells and FACS analyzed after 5 days. Representative flow cytometry plots and graph depict the frequencies of $CD4^+CD25^+FoxP3^+$ cells (n= 8 ± SEM, 2-tailed Student t test, *$p < 0.05$).

Supplementary Figure 9

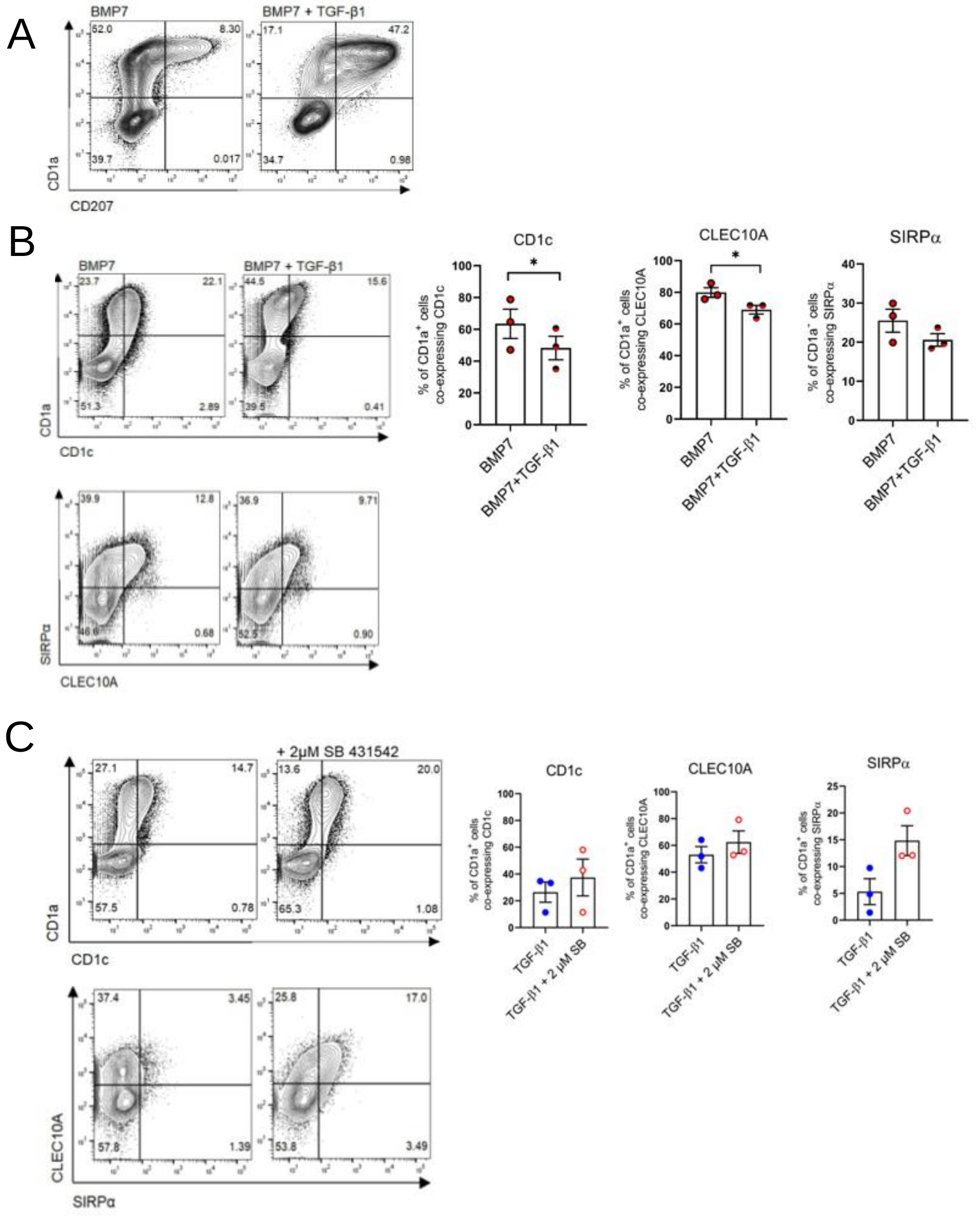

Fig.S9. BMP induced cDC2-like signature genes are diminished by TGF-β1 signaling. (A) Pre-expanded CD34+ progenitor cells were differentiated in LC lineage conditions in presence of BMP7 for 7 days. TGF-β1 was added to parallel cultures on day 5 (BMP7+TGF-β1) and analyzed on day 7. **(B)** Representative FACS plots show changes in cDC2 marker expression of cells generated in the BMP7 versus BMP7+TGF-β1 on day 7. Bars represent the percentages of CD1a+ gated cells co-expressing CD1c, SIRPα and CLEC10A (n = 3 ± SEM). **(C)** CD34+ progenitor cells were induced to

differentiate into LCs in the presence of basic cytokine cocktail plus TGF-β1. 2 μM ALK4/5/7 inhibitor (SB 431542) was added to parallel cultures and cells are analyzed at day 7. Bars indicate the changes in percentage of cells expressing cDC2 markers among gated $CD1a^+$ cells (n= 3 ± SEM, 2-tailed Student's t-test, *$p < 0.05$).

Supplementary Figure 10

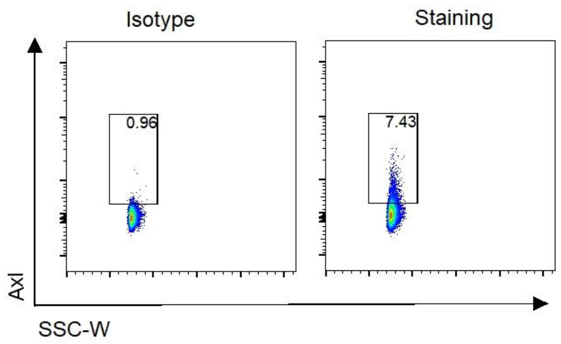

Fig.S10. Isotype control for anti-Axl antibody on blood cDC2s.
Representative flow cytometric gating strategy according to isotype control for identifying $Axl^{+}CD1c^{+}$ cells.

Supplementary Tables

Table S1. Surface marker based classification of dendritic cell subsets.

Table S2. List of Cytokines and Reagents used for generation of the data.

Table S3. List of antibodies used for generation of the data.

Table S4 . The discriminative gene set-based Upstream Regulators for CD14+ monocytes and DC2-A identified by IPA. The order is based on the IPA-based p-value. List of all significant upstream Regulators are shown ($p < 0.05$)

Table S5. The gene signature-linked Canonical Pathways of eDCs (Cheng et al) identified by IPA. All significant pathways calculated by the IPA-based p values are shown ($p < 0.05$)

Table S6. The gene signature-linked Upstream Regulators of eDCs (Cheng et al) identified by IPA. All significant pathways calculated by the IPA-based p values are shown ($p < 0.05$)

Table S7. Common canonical pathways expressed by eDCs (Cheng et al) and BMP7-DCs identified by VENNY. Only significant pathways were used for analysis ($p < 0.05$)

Table S8. Common Upstream Regulators expressed by eDCs (Cheng et al) and BMP7-DCs identified by VENNY. Only significant Upstream Regulators were used for analysis ($p < 0.05$)

Table S1. Surface marker-based classification of dendritic cell subsets

SURFACE MARKERS	EPIDERMAL DC[1,2]	LC	PSORIATIC LC[1,3,4]	BLOOD CDC2
**CD1A**	+	+	+	-
**CD1C**	+	+	++	+
**CD207**	-/ss+[2]	+	+	-
**AXL**	mRNA+[1]/ not analyzed	+	not analyzed	-/ ss+[5]
**REFERENCES**	(1,2,4,6)	(6–9)	(1,3,4)	(5,6,8,9)

ss=subset

1. Martini E, Wikén M, Cheuk S, Gallais Sérézal I, Baharom F, Ståhle M, et al. Dynamic Changes in Resident and Infiltrating Epidermal Dendritic Cells in Active and Resolved Psoriasis. J Invest Dermatol. 2017;137(4):865–73.

2. Bertram KM, Botting RA, Baharlou H, Rhodes JW, Rana H, Graham JD, et al. Identification of HIV transmitting CD11c+ human epidermal dendritic cells. Nat Commun [Internet]. 2019;10(1). Available from: http://dx.doi.org/10.1038/s41467-019-10697-w

3. Borek I, Köffel R, Feichtinger J, Spies M, Glitzner-Zeis E, Hochgerner M, et al. BMP7 aberrantly induced in the psoriatic epidermis instructs inflammation-associated Langerhans cells. J Allergy Clin Immunol. 2020;145(4):1194-1207.e11.

4. Liu X, Zhu R, Luo Y, Wang S, Zhao Y, Qiu Z, et al. Distinct human Langerhans cell subsets orchestrate reciprocal functions and require different developmental regulation. Immunity [Internet]. 2021;54(10):2305-2320.e11. Available from: https://doi.org/10.1016/j.immuni.2021.08.012

5. Villani AC, Satija R, Reynolds G, Sarkizova S, Shekhar K, Fletcher J, et al. Single-cell RNA-seq reveals new types of human blood dendritic cells, monocytes, and progenitors. Science (80-). 2017;356(6335).

6. Rhodes JW, Tong O, Harman AN, Turville SG. Human dendritic cell subsets, ontogeny, and impact on HIV infection. Front Immunol. 2019;10(MAY).

7. Bauer T, Zagórska A, Jurkin J, Yasmin N, Köffel R, Richter S, et al. Identification of Axl as a downstream effector of TGF-β1 during Langerhans cell differentiation and epidermal homeostasisBauer, T., Zagórska, A., Jurkin, J., Yasmin, N., Köffel, R., Richter, S., … Strobl, H. (2012). Identification of Axl as a downstream e. J Exp Med. 2012;209(11):2033–47.

8. Collin M, Bigley V. Human dendritic cell subsets: an update. Immunology. 2018;154(1):3–20.

9. Milne P, Bigley V, Gunawan M, Haniffa M, Collin M. CD1c+ blood dendritic cells have Langerhans cell potential. Blood [Internet]. 2015;125(3):470–3. Available from: http://dx.doi.org/10.1182/blood-2014-08-593582

Table S 2 Cytokines and Reagents

Cytokines and Reagents	Company
Human tumor necrosis factor-a (TNFα)	Peprotech (London, UK)
Human thrombopoietin (TPO)	Peprotech
Human stem cell factor (SCF)	Peprotech
Human FMS-related receptor tyrosine kinase 3 ligand (FLT3-L)	Peprotech
Human GM-CSF	Peprotech
Human M-CSF	Peprotech
Human IL-4	Peprotech
Human IL-6	Peprotech
SB 203580	Sigma-Aldrich
SB 431542	TOCRIS (Bristol, UK)
Dorsomorphin (DM)	TOCRIS
Human TGF-β1	R&D Systems (Minneapolis, Minn)
Human BMP7	Immunotools (Friesoythe, Germany)
Cell activation cocktail (without Brefeldin A)	Biolegend
Brefeldin A	Biolegend

Table S 3 Antibodies used for flow cytometry

Antibody	Fluorophore	Clone	Source
Antihuman CD11b	PE-Cy7	ICRF44	BioLegend
Antihuman CD11b	PE	ICRF44	BioLegend
Antihuman CD207	PE	DCGM4	Beckman Coulter
Antihuman CD324 (E-Cadherin)	AF647	67A4	BD Biosciences
Antihuman CD324 (E-cadherin)	PE-Cy7	67A4	BioLegend
Antihuman FoxP3	APC	236A/E7	Thermo Fisher Scientific
Antihuman CD1a	BV421	HI149	BD Biosciences
Antihuman CD1a	BUV395	HI149	BD Biosciences
Antihuman CD1a	APC	HI149	BioLegend
Antihuman HLA-DR	FITC	G46-6	BD Biosciences
Antihuman CD25	BV421	2A3	BD Biosciences
Antihuman CD4	PE	RPA-T4	BD Biosciences
Antihuman CD172a (SIRPα)	FITC	15-414	BioLegend
Antihuman CD5	BV605	UCHT2	BD Biosciences
Antihuman CD1c	BV421	F10/21A3	BD Biosciences
Antihuman CD1c	BB515	F10/21A3	BD Biosciences
Antihuman CD14	PE	M5E2	BioLegend
Antihuman CD14	AF700	M5E2	BD Biosciences
Antihuman Axl	APC	FAB154A	R and D Systems
Antihuman CD206 (MMR)	APC	15-2	BioLegend
Antihuman CD209	APC	eB-h209	Thermo Fisher Scientific
Antihuman CD11c	AF700	Bu15	BioLegend
Antihuman CLEC10A	APC	H037G3	BioLegend
Antihuman CLEC10A	PE	H037G3	BioLegend

Table S4. The discriminative gene set-based Upstream Regulators for CD14+ monocytes and DC2-A identified by IPA.
The order is based on the IPA-based p-value. List of all significant upstream Regulaotrs are shown (p < 0.05)

**DC2-A**			
**Upstream Regulator**	**Molecule Type**	**Predicted Activation State**	**p-value of overlap**
IL13	cytokine		1,62E-06
IFNG	cytokine	Activated	1,69E-06
Ige	complex		3,57E-06
IgG	complex		3,57E-06
ADCY	group		3,75E-06
lipopolysaccharide	chemical drug	Activated	3,78E-06
bee venom	chemical - endogenous non-mammalian		7,20E-06
dexamethasone	chemical drug		1,16E-05
tetradecanoylphorbol acetate	chemical drug	Activated	1,35E-05
SPI1	transcription regulator		3,72E-05
CSF2	cytokine	Activated	5,10E-05
H89	chemical drug		5,60E-05
Pde	group		5,67E-05
TYROBP	transmembrane receptor		6,78E-05
PDGF BB	complex	Activated	7,60E-05
prostaglandin E2	chemical - endogenous mammalian	Activated	9,86E-05
lipoteichoic acid	chemical - endogenous non-mammalian		1,02E-04
TREM1	transmembrane receptor	Activated	1,16E-04
IL4	cytokine	Activated	1,23E-04
AGT	growth factor		1,35E-04
TGFA	growth factor		1,62E-04
TCF3	transcription regulator		1,72E-04
IL33	cytokine		1,86E-04
IL13RA2	transmembrane receptor		2,13E-04
cholesterol	chemical - endogenous mammalian		2,45E-04
IL2	cytokine	Activated	2,69E-04
IL10	cytokine		3,02E-04
IgG1	complex		3,60E-04
KLF6	transcription regulator	Activated	3,92E-04
TOX	transcription regulator		4,29E-04
resiquimod	chemical drug		4,86E-04
Alpha catenin	group		5,03E-04
estrogen	chemical drug		5,05E-04
PSMB11	peptidase		5,15E-04
glucocorticoid	chemical drug		5,94E-04
Interferon alpha	group		6,18E-04
ELF1	transcription regulator		6,29E-04
NPC2	transporter		6,29E-04
U0126	chemical drug		6,75E-04
mir-132	microRNA		7,19E-04

IDR-1002	chemical reagent		7,19E-04
TNFSF13B	cytokine		8,99E-04
SU6656	chemical toxicant		9,18E-04
KN 93	chemical drug		9,18E-04
IL3	cytokine		9,40E-04
ERBB2	kinase		1,09E-03
10E,12Z-octadecadienoic acid	chemical - endogenous mammalian		1,10E-03
Immunoglobulin	complex		1,11E-03
CLEC10A	other		1,14E-03
IMMT	other		1,14E-03
camptothecin	chemical drug		1,16E-03
ASAH1	enzyme		1,20E-03
histone deacetylase	complex		1,20E-03
H-7	chemical - kinase inhibitor		1,26E-03
tobramycin	chemical drug		1,28E-03
morinda officinalis oligosaccharide extract	chemical drug		1,28E-03
indoprofen	chemical drug		1,28E-03
tandospirone	chemical drug		1,28E-03
WTIP	transcription regulator		1,28E-03
TCFL5	transcription regulator		1,28E-03
stearidonic acid	chemical - endogenous mammalian		1,28E-03
TIMELESS	other		1,28E-03
ZNF140	transcription regulator		1,28E-03
triazolam	chemical drug		1,28E-03
prostanoid	chemical drug		1,28E-03
P2y Receptor	group		1,28E-03
FAS	transmembrane receptor		1,29E-03
Salmonella enterica serotype abortus equi lipopolysaccharide	chemical toxicant		1,29E-03
EGF	growth factor		1,30E-03
chloropromazine	chemical drug		1,32E-03
IL21	cytokine		1,48E-03
dopamine	chemical - endogenous mammalian		1,50E-03
lipid A	chemical toxicant		1,51E-03
TGFBR2	kinase		1,52E-03
CpG oligonucleotide	chemical drug		1,57E-03
RAG1	enzyme		1,65E-03
isoprenaline	chemical drug		1,65E-03
progesterone	chemical - endogenous mammalian		1,67E-03
Akt	group		1,73E-03
TLR4	transmembrane receptor		1,76E-03
PTGS2	enzyme		1,89E-03
ionomycin	chemical reagent		2,00E-03
Am 580	chemical reagent		2,17E-03
BCR (complex)	complex		2,24E-03

EDN1	cytokine		2,31E-03
BIRC5	other		2,34E-03
SOX4	transcription regulator		2,37E-03
L-methionine	chemical - endogenous mammalian		2,42E-03
forskolin	chemical toxicant	Activated	2,44E-03
bicuculline	chemical - endogenous non-mammalian		2,50E-03
NR3C1	ligand-dependent nuclear receptor		2,52E-03
7beta-hydroxycholesterol	chemical - endogenous mammalian		2,56E-03
prostaglandin E3	chemical - endogenous mammalian		2,56E-03
4-nitrobenzoic acid	chemical toxicant		2,56E-03
A2M-AS1	other		2,56E-03
CYRIA	other		2,56E-03
Pde3	group		2,56E-03
CDK10	kinase		2,56E-03
STX6	transporter		2,56E-03
GEMIN2	other		2,56E-03
capmatinib	chemical drug		2,56E-03
TMPRSS4	peptidase		2,56E-03
Bhlhe41	transcription regulator		2,56E-03
SNRPA	other		2,56E-03
gliclazide	chemical drug		2,56E-03
urushiol	chemical - endogenous non-mammalian		2,56E-03
1,1-bis(3'-indolyl)-1-(4-chlorophenyl)methane	chemical reagent		2,56E-03
telmisartan	chemical drug		2,59E-03
TLR7	transmembrane receptor		2,67E-03
palbociclib	chemical drug		2,68E-03
IRF6	transcription regulator		2,68E-03
dalfampridine	chemical drug		2,76E-03
clozapine	chemical drug		2,76E-03
Pkc(s)	group		2,78E-03
butyric acid	chemical - endogenous mammalian		2,84E-03
TAL1	transcription regulator		2,92E-03
CITED2	transcription regulator		2,92E-03
IL1B	cytokine	Activated	2,97E-03
TNF	cytokine	Activated	3,06E-03
BSCL2	other		3,13E-03
GSTO1	enzyme		3,13E-03
ATP-gamma-S	chemical reagent		3,32E-03
Go 6976	chemical drug		3,32E-03
Histone h3	group		3,37E-03
rosiglitazone	chemical drug		3,40E-03
KCNE3	ion channel		3,42E-03
BCL6	transcription regulator		3,50E-03
AHR	ligand-dependent nuclear receptor		3,57E-03

USF2	transcription regulator		3,62E-03
Igm	complex		3,83E-03
D-4476	chemical drug		3,84E-03
CSNK1E	kinase		3,84E-03
EMP2	other		3,84E-03
recombinant interferon gamma	biologic drug		3,84E-03
RBMX	other		3,84E-03
fosfomycin	chemical drug		3,84E-03
CRTC1-MAML2	fusion gene/product		3,84E-03
HGF	growth factor		3,89E-03
KRAS	enzyme		4,01E-03
corticosteroid	chemical drug		4,15E-03
gemcitabine	chemical drug		4,15E-03
25-hydroxycholesterol	chemical reagent		4,15E-03
dinoprost	chemical - endogenous mammalian		4,36E-03
PLCG2	enzyme		4,36E-03
WT1	transcription regulator		4,48E-03
PRNP	other		4,82E-03
cyclic AMP	chemical - endogenous mammalian		4,92E-03
epinephrine	chemical - endogenous mammalian		5,05E-03
platelet activating factor-C16	chemical - endogenous mammalian		5,11E-03
adalimumab	biologic drug		5,11E-03
nebivolol	chemical drug		5,11E-03
ENPP7	enzyme		5,11E-03
CYRIB	other		5,11E-03
CUEDC2	other		5,11E-03
TBR1	transcription regulator		5,11E-03
RAB11A	enzyme		5,11E-03
CHRM4	G-protein coupled receptor		5,11E-03
VSNL1	other		5,11E-03
GZMB	peptidase		5,11E-03
WTAP	other		5,11E-03
NSC719239	chemical drug		5,11E-03
LDL	complex		5,17E-03
USF1	transcription regulator		5,29E-03
5-O-mycolyl-beta-araf-(1->2)-5-O-mycolyl-alpha-araf-(1->1')-glycerol	chemical - endogenous non-mammalian		5,29E-03
LGALS1	other		5,41E-03
THRB	ligand-dependent nuclear receptor		5,55E-03
AREG	growth factor		5,66E-03
RNA polymerase II	complex		5,71E-03
haloperidol	chemical drug		5,79E-03
EGFR	kinase		5,86E-03
CREB1	transcription regulator		5,97E-03
PRDM1	transcription regulator		6,11E-03

GATA1	transcription regulator		6,22E-03
MYD88	other		6,34E-03
PD98059	chemical - kinase inhibitor		6,34E-03
(+)-fluprostenol	chemical drug		6,39E-03
estradiol 17beta-cypionate	chemical drug		6,39E-03
FOXN4	transcription regulator		6,39E-03
MARCHF3	other		6,39E-03
INSL5	other		6,39E-03
farletuzumab	biologic drug		6,39E-03
TRA2B	other		6,39E-03
CLDN2	other		6,39E-03
Collagen type V	complex		6,39E-03
PI3K (complex)	complex		6,46E-03
NFKB1	transcription regulator		6,46E-03
NR4A2	ligand-dependent nuclear receptor		6,57E-03
trinitrobenzenesulfonic acid	chemical reagent		6,57E-03
DYSF	other		6,70E-03
VEGFA	growth factor		6,88E-03
miR-146a-5p (and other miRNAs w/seed GAGAACU)	mature microRNA		6,97E-03
IL17A	cytokine		7,32E-03
Pdgf (complex)	complex		7,39E-03
GW9662	chemical reagent		7,53E-03
ADORA2	group		7,66E-03
BICC1	other		7,66E-03
AMER1	other		7,66E-03
PDZK1IP1	other		7,66E-03
DR1	transcription regulator		7,66E-03
GMFG	growth factor		7,66E-03
POLRMT	enzyme		7,66E-03
VTX-2337	chemical drug		7,66E-03
ZYX	other		7,66E-03
GABA receptor	complex		7,66E-03
paclitaxel	chemical drug		7,85E-03
erlotinib	chemical drug		7,97E-03
trichostatin A	chemical drug		8,02E-03
CXCL8	cytokine		8,41E-03
mifepristone	chemical drug		8,53E-03
N-[N-(3,5-difluorophenacetyl-L-Ala)]-S-phenylglycine t-butyl ester	chemical - protease inhibitor		8,56E-03
SMARCA4	transcription regulator		8,65E-03
methylprednisolone	chemical drug		8,70E-03
GnRH analog	biologic drug		8,74E-03
Collagen type III	complex		8,93E-03
AGR2	other		8,93E-03
XPA	other		8,93E-03

NPAS2	transcription regulator		8,93E-03
PITPNA	transporter		8,93E-03
Eda	other		8,93E-03
fluphenazine	chemical drug		8,93E-03
diazepam	chemical drug		8,93E-03
dihydrotestosterone	chemical - endogenous mammalian		9,27E-03
T-5224	chemical reagent		9,49E-03
Lh	complex		9,93E-03
BTK	kinase		1,01E-02
N6-benzoyl-cAMP	chemical reagent		1,02E-02
SPIC	transcription regulator		1,02E-02
GAL3ST1	enzyme		1,02E-02
APCS	other		1,02E-02
Ifi202b	other		1,02E-02
PLP2	transporter		1,02E-02
ZNF91	transcription regulator		1,02E-02
naproxen	chemical drug		1,02E-02
raclopride	chemical drug		1,02E-02
cinnamon powder	chemical reagent		1,02E-02
IL1	group		1,02E-02
YAP1	transcription regulator		1,04E-02
bucladesine	chemical toxicant		1,11E-02
APOE	transporter		1,11E-02
TGFB1	growth factor		1,13E-02
dopamine receptor	group		1,15E-02
bazedoxifene	chemical drug		1,15E-02
quisinostat	chemical drug		1,15E-02
SPTAN1	other		1,15E-02
TRG	other		1,15E-02
PPM1A	phosphatase		1,15E-02
MC1R	G-protein coupled receptor		1,15E-02
EDA	cytokine		1,15E-02
adapalene	chemical drug		1,15E-02
poly rI:rC-RNA	biologic drug		1,17E-02
miR-34a-5p (and other miRNAs w/seed GGCAGUG)	mature microRNA		1,25E-02
HTT	transcription regulator		1,26E-02
13,14-dihydro-15-ketoprostaglandin D2	chemical - endogenous mammalian		1,27E-02
voltage-gated calcium channel	complex		1,27E-02
Arntl-Clock	complex		1,27E-02
TLR2/3/4/9	group		1,27E-02
ITGA4	transmembrane receptor		1,27E-02
MIR320	group		1,27E-02
mir-217	microRNA		1,27E-02
IL9R	transmembrane receptor		1,27E-02

CAMK2A	kinase		1,27E-02
GPAT4	enzyme		1,27E-02
butylhydroxybutylnitrosamine	chemical toxicant		1,27E-02
clomipramine	chemical drug		1,27E-02
calcium chloride	chemical drug		1,27E-02
alpha-naphthoflavone	chemical reagent		1,27E-02
STAT3	transcription regulator		1,35E-02
ADORA2A	G-protein coupled receptor		1,36E-02
semaxinib	chemical drug		1,36E-02
AR	ligand-dependent nuclear receptor		1,37E-02
ADAM10	peptidase		1,38E-02
azathioprine	chemical drug		1,40E-02
MGAT1	enzyme		1,40E-02
TAF5	transcription regulator		1,40E-02
ST3-Hel2A-2	chemical reagent		1,40E-02
Ncoa6	transcription regulator		1,40E-02
delphinidin	chemical - endogenous non-mammalian		1,40E-02
CD40LG	cytokine		1,41E-02
VHL	transcription regulator		1,42E-02
Pka	complex		1,44E-02
FSH	complex		1,51E-02
NR3C2	ligand-dependent nuclear receptor		1,51E-02
Rp-8-Br-cAMPS	chemical - kinase inhibitor		1,53E-02
chlorcyclizine	chemical drug		1,53E-02
USP9X	peptidase		1,53E-02
HLF	transcription regulator		1,53E-02
baclofen	chemical drug		1,53E-02
genistein	chemical drug		1,53E-02
Notch	group		1,53E-02
Histone h4	group		1,55E-02
GFI1	transcription regulator		1,61E-02
GNAQ	enzyme		1,63E-02
STAT1	transcription regulator		1,65E-02
Pdgfr	group		1,65E-02
perhexiline	chemical drug		1,65E-02
particulate matter	chemical reagent		1,65E-02
mir-127	microRNA		1,65E-02
THBS2	other		1,65E-02
CRY2	enzyme		1,65E-02
PLA2G2E	enzyme		1,65E-02
CRY1	enzyme		1,65E-02
oxonic acid	chemical drug		1,65E-02
astressin 2B	biologic drug		1,65E-02
JAK2	kinase		1,67E-02

NFAT5	transcription regulator		1,69E-02
ZBTB10	transcription regulator		1,74E-02
CREM	transcription regulator		1,78E-02
dazucorilant	chemical drug		1,78E-02
Actin	group		1,78E-02
DNAJC3	other		1,78E-02
MGAT3	enzyme		1,78E-02
LAMC1	other		1,78E-02
pyruvic acid	chemical - endogenous mammalian		1,78E-02
palmitoleic acid	chemical - endogenous mammalian		1,78E-02
prostaglandin	chemical drug		1,78E-02
clarithromycin	chemical drug		1,90E-02
TAP1	transporter		1,90E-02
PER2	transcription regulator		1,90E-02
KHDRBS1	transcription regulator		1,90E-02
MAS1	G-protein coupled receptor		1,90E-02
ST6GAL1	enzyme		1,90E-02
SP2	transcription regulator		1,90E-02
CRTC1	transcription regulator		1,90E-02
EDNRA	transmembrane receptor		1,90E-02
finerenone	chemical drug		1,90E-02
fevipiprant	chemical drug		1,90E-02
zalcitabine	chemical drug		1,90E-02
ARNT	transcription regulator		1,90E-02
IGF2	growth factor		1,99E-02
CEBPA	transcription regulator		2,01E-02
S100A9	other		2,01E-02
NREP	other		2,03E-02
IGFBP1	other		2,03E-02
cystamine	chemical drug		2,03E-02
fluticasone propionate	chemical drug		2,06E-02
APC	enzyme		2,10E-02
PTH	other		2,15E-02
Pam3-Cys-Ser-Lys4	chemical reagent		2,15E-02
adenine	chemical - endogenous mammalian		2,16E-02
3-deoxy-2-octulosonic acid(2)-lipid A	chemical - endogenous non-mammalian		2,16E-02
triclosan	chemical drug		2,16E-02
TOR1A	enzyme		2,16E-02
USP1	peptidase		2,16E-02
premarin	chemical drug		2,16E-02
nimesulide	chemical drug		2,16E-02
norepinephrine	chemical - endogenous mammalian		2,17E-02
Tgf beta	group		2,21E-02
FOXP3	transcription regulator		2,24E-02

S100A8	other		2,26E-02
HOXA9	transcription regulator		2,26E-02
docosahexaenoic acid	chemical drug		2,26E-02
aldosterone	chemical - endogenous mammalian		2,26E-02
Pdgf Ab	complex		2,28E-02
PHB2	transcription regulator		2,28E-02
mir-451	microRNA		2,28E-02
HOXB9	transcription regulator		2,28E-02
CD70	cytokine		2,28E-02
EREG	growth factor		2,28E-02
PTGFR	G-protein coupled receptor		2,28E-02
HDC	enzyme		2,28E-02
NCR2	transmembrane receptor		2,28E-02
CH-223191	chemical reagent		2,28E-02
nimodipine	chemical drug		2,28E-02
thyroid hormone	chemical - endogenous mammalian		2,33E-02
4-hydroxytamoxifen	chemical drug		2,33E-02
kainic acid	chemical toxicant		2,38E-02
CBX7	other		2,41E-02
PROM1	other		2,41E-02
CRHR1	G-protein coupled receptor		2,41E-02
SLC29A1	transporter		2,41E-02
ITGB4	transmembrane receptor		2,41E-02
DBP	transcription regulator		2,41E-02
thiazolidinedione	chemical drug		2,41E-02
MR-409	chemical reagent		2,41E-02
leuprolide	biologic drug		2,41E-02
gefitinib	chemical drug		2,43E-02
kawain	chemical - endogenous non-mammalian		2,53E-02
mir-192	microRNA		2,53E-02
HOXD3	transcription regulator		2,53E-02
STAU1	transporter		2,53E-02
HNRNPA1	other		2,53E-02
YAP/TAZ	group		2,53E-02
antimycin A	chemical - endogenous non-mammalian		2,53E-02
prazosin	chemical drug		2,53E-02
thioridazine	chemical drug		2,53E-02
MAP2K1	kinase		2,62E-02
Iga	complex		2,66E-02
SRA1	transcription regulator		2,66E-02
EDN3	other		2,66E-02
miR-31-5p (and other miRNAs w/seed GGCAAGA)	mature microRNA		2,66E-02
RND3	enzyme		2,66E-02
bisphenol A	chemical - endogenous mammalian		2,67E-02

FGF2	growth factor		2,67E-02
tetrachlorodibenzodioxin	chemical toxicant		2,70E-02
RB1	transcription regulator		2,76E-02
INHBA	growth factor		2,77E-02
TRIM2	enzyme		2,78E-02
GPNMB	enzyme		2,78E-02
KLF10	transcription regulator		2,78E-02
ITGA2	transmembrane receptor		2,78E-02
lapatinib	chemical drug		2,78E-02
fumonisin B1	chemical toxicant		2,78E-02
dichloroacetic acid	chemical drug		2,78E-02
farnesol	chemical reagent		2,78E-02
NUPR1	transcription regulator		2,78E-02
ARNT2	transcription regulator		2,85E-02
PDGF-DD	complex		2,91E-02
ORMDL3	other		2,91E-02
FAM3B	cytokine		2,91E-02
MED12	transcription regulator		2,91E-02
SLC2A4	transporter		2,91E-02
BMP15	growth factor		2,91E-02
miR-19b-3p (and other miRNAs w/seed GUGCAAA)	mature microRNA		2,91E-02
MGAT5	enzyme		2,91E-02
CRHR2	G-protein coupled receptor		2,91E-02
CD44	other		2,95E-02
GATA6	transcription regulator		3,00E-02
FOXF1	transcription regulator		3,03E-02
Mt2	other		3,03E-02
THZ2	chemical drug		3,03E-02
THZ1	chemical drug		3,03E-02
estradiol-17beta-benzoate	chemical reagent		3,05E-02
SP3	transcription regulator		3,10E-02
deferoxamine	chemical drug		3,13E-02
CCL3	cytokine		3,15E-02
mir-7	microRNA		3,15E-02
UBE3A	enzyme		3,15E-02
ITGA1	other		3,15E-02
amitriptyline	chemical drug		3,15E-02
LY294002	chemical drug		3,20E-02
PDGF-AA	complex		3,28E-02
RNF20	enzyme		3,28E-02
CD9	other		3,28E-02
CLEC4E	other		3,28E-02
AZGP1	transporter		3,28E-02
IOX2	chemical reagent		3,28E-02

HIF1A	transcription regulator		3,32E-02
STAT4	transcription regulator		3,35E-02
risperidone	chemical drug		3,40E-02
CD300LF	other		3,40E-02
CTSS	peptidase		3,40E-02
mir-144	microRNA		3,40E-02
nilvadipine	chemical drug		3,40E-02
ECSIT	transcription regulator		3,40E-02
Mt1	other		3,40E-02
cyanocobalamin	chemical - endogenous mammalian		3,40E-02
INSR	kinase		3,41E-02
APP	other		3,44E-02
CG	complex		3,51E-02
IPMK	kinase		3,53E-02
ACTL6A	other		3,53E-02
MAP2K2	kinase		3,53E-02
PRKAR2B	kinase		3,53E-02
TFPI2	other		3,53E-02
Smad2/3-Smad4	complex		3,65E-02
LUCAT1	other		3,65E-02
7(R)-maresin 1	chemical - endogenous mammalian		3,65E-02
imipramine	chemical drug		3,65E-02
SFRP4	transmembrane receptor		3,77E-02
DDIT4	other		3,77E-02
VAV3	cytokine		3,77E-02
IRAK3	kinase		3,77E-02
NUP98-HOXA9	fusion gene/product		3,77E-02
HISTONE	group		3,90E-02
MAC	complex		3,90E-02
mir-31	microRNA		3,90E-02
VTN	other		3,90E-02
VAV2	transcription regulator		3,90E-02
urethane	chemical toxicant		3,90E-02
fluvoxamine	chemical drug		3,90E-02
NOSTRIN	transcription regulator		4,02E-02
PTH1R	G-protein coupled receptor		4,02E-02
SRSF1	other		4,02E-02
ADIPOR1	transmembrane receptor		4,02E-02
KNG1	other		4,02E-02
LTB4R	G-protein coupled receptor		4,02E-02
ceramide	chemical - endogenous mammalian		4,02E-02
ERK	group		4,05E-02
ADIPOQ	other		4,14E-02
Fgf	group		4,14E-02

HLX	transcription regulator		4,14E-02
PHF12	transcription regulator		4,14E-02
ARHGAP31	other		4,14E-02
EPAS1	transcription regulator		4,17E-02
indomethacin	chemical drug		4,23E-02
ZBTB7A	transcription regulator		4,27E-02
deoxycorticosterone acetate/potassium chloride/sodium chloride	chemical reagent		4,27E-02
bryostatin 1	chemical drug		4,27E-02
cicaprost	chemical drug		4,27E-02
CLOCK	transcription regulator		4,32E-02
CEBPB	transcription regulator		4,35E-02
interferon alfacon-1	biologic drug		4,39E-02
resatorvid	chemical drug		4,39E-02
PRKCZ	kinase		4,39E-02
sodium orthovanadate	chemical reagent		4,39E-02
E. coli B5 lipopolysaccharide	chemical - endogenous non-mammalian		4,44E-02
CSF1	cytokine		4,47E-02
CpG ODN 2216	chemical reagent		4,51E-02
TNFSF14	cytokine		4,51E-02
miR-450a-5p (and other miRNAs w/seed UUUGCGA)	mature microRNA		4,51E-02
miR-92a-3p (and other miRNAs w/seed AUUGCAC)	mature microRNA		4,51E-02
NCSTN	peptidase		4,51E-02
UPF1	enzyme		4,51E-02
AIM2	other		4,51E-02
amiodarone	chemical drug		4,51E-02
phorbol 12,13-dibutyrate	chemical - endogenous non-mammalian		4,51E-02
PPARD	ligand-dependent nuclear receptor		4,62E-02
crizotinib	chemical drug		4,63E-02
IKZF2	transcription regulator		4,63E-02
GNB2	enzyme		4,63E-02
GTF2B	transcription regulator		4,63E-02
PRKAR1A	kinase		4,63E-02
ACE2	peptidase		4,63E-02
rolipram	chemical drug		4,63E-02
exenatide	biologic drug		4,63E-02
CCAR2	peptidase		4,76E-02
daporinad	chemical drug		4,76E-02
LHCGR	G-protein coupled receptor		4,76E-02
eprenetapopt	chemical drug		4,76E-02
POU4F2	transcription regulator		4,76E-02
ethylene glycol tetraacetic acid	chemical reagent		4,76E-02
calcitriol	chemical drug		4,85E-02
F2	peptidase		4,87E-02
ITCH	enzyme		4,88E-02

GNB1	enzyme		4,88E-02
RAD21	transcription regulator		4,88E-02
palmitic acid	chemical - endogenous mammalian		4,94E-02

Table S5. The gene signature-linked Canonical Pathways of eDCs (Cheng et al.) identified by IPA.
All significant pathways calculated by the IPA-based p values are shown ($p < 0.05$)

**Canonical Pathway (eDC Cheng et al.)**	**p-value of overlap**
Antigen Presentation Pathway	2,51E-18
MSP-RON Signaling In Macrophages Pathway	2,00E-17
Th1 Pathway	6,31E-15
Phagosome Maturation	1,58E-14
Integrin Signaling	2,00E-14
B Cell Development	2,51E-14
Neuroinflammation Signaling Pathway	1,58E-13
fMLP Signaling in Neutrophils	3,16E-13
Regulation of Actin-based Motility by Rho	3,16E-13
Glucocorticoid Receptor Signaling	5,01E-13
Th1 and Th2 Activation Pathway	7,94E-13
RAC Signaling	7,94E-13
IL-4 Signaling	7,94E-13
Th2 Pathway	6,31E-12
Actin Nucleation by ARP-WASP Complex	1,00E-11
Germ Cell-Sertoli Cell Junction Signaling	5,01E-11
Glioma Invasiveness Signaling	5,01E-11
Production of Nitric Oxide and Reactive Oxygen Species in Macrophages	5,01E-11
Actin Cytoskeleton Signaling	1,41E-10
Signaling by Rho Family GTPases	1,48E-10
Remodeling of Epithelial Adherens Junctions	2,40E-10
IL-8 Signaling	2,82E-10
Virus Entry via Endocytic Pathways	6,31E-10
ILK Signaling	7,59E-10
PD-1, PD-L1 cancer immunotherapy pathway	8,32E-10
Axonal Guidance Signaling	1,23E-09
Fcγ Receptor-mediated Phagocytosis in Macrophages and Monocytes	1,55E-09
Estrogen Receptor Signaling	2,14E-09
Clathrin-mediated Endocytosis Signaling	2,63E-09
RHOGDI Signaling	2,69E-09
Hepatic Fibrosis Signaling Pathway	4,27E-09
Ephrin Receptor Signaling	6,03E-09
HER-2 Signaling in Breast Cancer	6,92E-09
Crosstalk between Dendritic Cells and Natural Killer Cells	1,00E-08
HMGB1 Signaling	1,10E-08
Molecular Mechanisms of Cancer	1,48E-08
Leukocyte Extravasation Signaling	1,62E-08

Epithelial Adherens Junction Signaling	2,88E-08
PEDF Signaling	3,72E-08
LPS-stimulated MAPK Signaling	4,27E-08
Acute Phase Response Signaling	5,01E-08
Apoptosis Signaling	5,13E-08
CLEAR Signaling Pathway	6,61E-08
IL-10 Signaling	6,76E-08
Synaptogenesis Signaling Pathway	9,12E-08
Natural Killer Cell Signaling	1,45E-07
PI3K/AKT Signaling	1,45E-07
Dendritic Cell Maturation	1,51E-07
Thrombin Signaling	1,70E-07
Tumor Microenvironment Pathway	1,82E-07
Role of Tissue Factor in Cancer	1,91E-07
Reelin Signaling in Neurons	2,45E-07
Cholecystokinin/Gastrin-mediated Signaling	2,57E-07
NGF Signaling	2,82E-07
PI3K Signaling in B Lymphocytes	3,55E-07
TNFR1 Signaling	3,55E-07
CXCR4 Signaling	4,79E-07
Gαq Signaling	5,25E-07
Colorectal Cancer Metastasis Signaling	5,89E-07
IL-6 Signaling	6,03E-07
Acute Myeloid Leukemia Signaling	7,76E-07
CD28 Signaling in T Helper Cells	1,02E-06
Macropinocytosis Signaling	1,12E-06
Sertoli Cell-Sertoli Cell Junction Signaling	1,23E-06
Xenobiotic Metabolism Signaling	1,62E-06
Semaphorin Signaling in Neurons	1,66E-06
mTOR Signaling	1,78E-06
Granulocyte Adhesion and Diapedesis	2,00E-06
JAK/STAT Signaling	2,29E-06
Renin-Angiotensin Signaling	2,29E-06
GNRH Signaling	2,45E-06
LXR/RXR Activation	2,51E-06
RHOA Signaling	2,75E-06
PPARα/RXRα Activation	2,95E-06
PTEN Signaling	3,63E-06
TWEAK Signaling	3,89E-06
PPAR Signaling	3,98E-06
Erythropoietin Signaling Pathway	4,57E-06

Atherosclerosis Signaling	4,90E-06
HGF Signaling	5,25E-06
Ferroptosis Signaling Pathway	5,25E-06
Oxidative Phosphorylation	5,62E-06
P2Y Purigenic Receptor Signaling Pathway	5,62E-06
Role of JAK1, JAK2 and TYK2 in Interferon Signaling	5,75E-06
Cardiac Hypertrophy Signaling	6,31E-06
HIF1α Signaling	6,46E-06
IL-12 Signaling and Production in Macrophages	7,08E-06
Prostate Cancer Signaling	7,24E-06
PAK Signaling	1,02E-05
TREM1 Signaling	1,05E-05
Angiopoietin Signaling	1,05E-05
Phagosome Formation	1,07E-05
NF-κB Activation by Viruses	1,15E-05
Hepatic Fibrosis / Hepatic Stellate Cell Activation	1,29E-05
Glioblastoma Multiforme Signaling	1,51E-05
Pancreatic Adenocarcinoma Signaling	1,91E-05
Type I Diabetes Mellitus Signaling	2,19E-05
Tight Junction Signaling	2,34E-05
Sirtuin Signaling Pathway	2,69E-05
Paxillin Signaling	2,69E-05
Gα12/13 Signaling	3,16E-05
CCR3 Signaling in Eosinophils	3,63E-05
STAT3 Pathway	3,63E-05
Agranulocyte Adhesion and Diapedesis	3,89E-05
Role of MAPK Signaling in Promoting the Pathogenesis of Influenza	4,07E-05
RANK Signaling in Osteoclasts	4,07E-05
Insulin Secretion Signaling Pathway	4,27E-05
Ephrin B Signaling	4,68E-05
Amyotrophic Lateral Sclerosis Signaling	5,01E-05
MSP-RON Signaling In Cancer Cells Pathway	5,01E-05
CDC42 Signaling	5,62E-05
Xenobiotic Metabolism General Signaling Pathway	6,17E-05
Wound Healing Signaling Pathway	6,17E-05
Death Receptor Signaling	6,17E-05
Mitochondrial Dysfunction	7,08E-05
CD27 Signaling in Lymphocytes	7,41E-05
Pulmonary Healing Signaling Pathway	7,41E-05
Role of NFAT in Regulation of the Immune Response	8,32E-05
PKCθ Signaling in T Lymphocytes	9,33E-05

Semaphorin Neuronal Repulsive Signaling Pathway	1,00E-04
Renal Cell Carcinoma Signaling	1,00E-04
14-3-3-mediated Signaling	1,10E-04
Osteoarthritis Pathway	1,12E-04
Role of Macrophages, Fibroblasts and Endothelial Cells in Rheumatoid Arthritis	1,12E-04
NRF2-mediated Oxidative Stress Response	1,15E-04
Systemic Lupus Erythematosus In T Cell Signaling Pathway	1,23E-04
Role of Pattern Recognition Receptors in Recognition of Bacteria and Viruses	1,35E-04
BAG2 Signaling Pathway	1,41E-04
Chronic Myeloid Leukemia Signaling	1,45E-04
T Cell Receptor Signaling	1,58E-04
Cardiac Hypertrophy Signaling (Enhanced)	1,62E-04
Activation of IRF by Cytosolic Pattern Recognition Receptors	1,70E-04
Induction of Apoptosis by HIV1	1,70E-04
IL-23 Signaling Pathway	1,74E-04
BMP signaling pathway	1,82E-04
Role of IL-17F in Allergic Inflammatory Airway Diseases	1,95E-04
iNOS Signaling	1,95E-04
Ephrin A Signaling	1,95E-04
Opioid Signaling Pathway	1,95E-04
HOTAIR Regulatory Pathway	1,95E-04
Regulation Of The Epithelial Mesenchymal Transition By Growth Factors Pathway	2,09E-04
Oxytocin Signaling Pathway	2,14E-04
Regulation of Cellular Mechanics by Calpain Protease	2,14E-04
Iron homeostasis signaling pathway	2,29E-04
TNFR2 Signaling	2,57E-04
Apelin Endothelial Signaling Pathway	2,57E-04
Agrin Interactions at Neuromuscular Junction	2,75E-04
GM-CSF Signaling	2,75E-04
Melanoma Signaling	2,75E-04
MYC Mediated Apoptosis Signaling	2,75E-04
Gap Junction Signaling	2,82E-04
Adrenomedullin signaling pathway	2,95E-04
Pulmonary Fibrosis Idiopathic Signaling Pathway	3,31E-04
Sphingosine-1-phosphate Signaling	3,47E-04
Coronavirus Pathogenesis Pathway	3,47E-04
IL-9 Signaling	3,98E-04
MIF-mediated Glucocorticoid Regulation	4,57E-04
GDNF Family Ligand-Receptor Interactions	4,57E-04
Type II Diabetes Mellitus Signaling	4,90E-04
Endocannabinoid Developing Neuron Pathway	5,25E-04

VDR/RXR Activation	5,37E-04
Relaxin Signaling	5,50E-04
Role of IL-17A in Arthritis	5,62E-04
IL-3 Signaling	5,75E-04
Sumoylation Pathway	5,75E-04
G Beta Gamma Signaling	5,75E-04
Docosahexaenoic Acid (DHA) Signaling	5,89E-04
IL-17A Signaling in Fibroblasts	5,89E-04
IGF-1 Signaling	6,17E-04
Cancer Drug Resistance By Drug Efflux	6,17E-04
Aryl Hydrocarbon Receptor Signaling	6,61E-04
Estrogen-Dependent Breast Cancer Signaling	6,61E-04
BEX2 Signaling Pathway	6,61E-04
EIF2 Signaling	8,32E-04
Role of PKR in Interferon Induction and Antiviral Response	8,51E-04
Antioxidant Action of Vitamin C	1,00E-03
Xenobiotic Metabolism AHR Signaling Pathway	1,02E-03
ICOS-ICOSL Signaling in T Helper Cells	1,02E-03
ID1 Signaling Pathway	1,10E-03
MIF Regulation of Innate Immunity	1,15E-03
Role of PI3K/AKT Signaling in the Pathogenesis of Influenza	1,23E-03
Ceramide Signaling	1,26E-03
Bladder Cancer Signaling	1,26E-03
RAR Activation	1,29E-03
CD40 Signaling	1,35E-03
Fc Epsilon RI Signaling	1,41E-03
Apelin Pancreas Signaling Pathway	1,41E-03
Glutathione Redox Reactions I	1,55E-03
Role of JAK1 and JAK3 in γc Cytokine Signaling	1,55E-03
ERBB Signaling	1,62E-03
Altered T Cell and B Cell Signaling in Rheumatoid Arthritis	1,74E-03
Small Cell Lung Cancer Signaling	1,82E-03
IL-1 Signaling	1,82E-03
Phenylalanine Degradation IV (Mammalian, via Side Chain)	1,86E-03
Glioma Signaling	1,91E-03
Heme Degradation	1,91E-03
Autophagy	1,95E-03
FAT10 Cancer Signaling Pathway	2,04E-03
Huntington's Disease Signaling	2,09E-03
ERK5 Signaling	2,24E-03
Caveolar-mediated Endocytosis Signaling	2,40E-03

Hypoxia Signaling in the Cardiovascular System	2,57E-03
Hepatic Cholestasis	2,63E-03
Synaptic Long Term Potentiation	2,69E-03
Antiproliferative Role of Somatostatin Receptor 2	2,75E-03
p70S6K Signaling	2,82E-03
Airway Inflammation in Asthma	2,88E-03
Toll-like Receptor Signaling	2,95E-03
Regulation of the Epithelial-Mesenchymal Transition Pathway	3,02E-03
4-1BB Signaling in T Lymphocytes	3,24E-03
Chemokine Signaling	3,31E-03
Telomerase Signaling	3,39E-03
α-Adrenergic Signaling	3,55E-03
T Cell Exhaustion Signaling Pathway	3,72E-03
Circadian Rhythm Signaling	3,89E-03
Interferon Signaling	3,98E-03
Polyamine Regulation in Colon Cancer	3,98E-03
VEGF Family Ligand-Receptor Interactions	4,27E-03
Hereditary Breast Cancer Signaling	4,47E-03
Endometrial Cancer Signaling	4,57E-03
GADD45 Signaling	4,57E-03
Prolactin Signaling	4,68E-03
Role of Hypercytokinemia/hyperchemokinemia in the Pathogenesis of Influenza	4,68E-03
IL-13 Signaling Pathway	5,25E-03
Inhibition of Matrix Metalloproteases	5,25E-03
Inflammasome pathway	5,37E-03
Regulation of eIF4 and p70S6K Signaling	5,50E-03
Thrombopoietin Signaling	5,62E-03
ERK/MAPK Signaling	6,17E-03
Apelin Adipocyte Signaling Pathway	6,17E-03
Stearate Biosynthesis I (Animals)	6,46E-03
OX40 Signaling Pathway	6,92E-03
April Mediated Signaling	6,92E-03
Putrescine Degradation III	7,08E-03
IL-17 Signaling	7,24E-03
IL-17A Signaling in Airway Cells	7,41E-03
B Cell Activating Factor Signaling	7,59E-03
Oncostatin M Signaling	7,59E-03
Graft-versus-Host Disease Signaling	7,76E-03
ERBB4 Signaling	7,76E-03
Role of Osteoblasts, Osteoclasts and Chondrocytes in Rheumatoid Arthritis	7,94E-03
Necroptosis Signaling Pathway	7,94E-03

TGF-β Signaling	8,13E-03
Protein Kinase A Signaling	8,13E-03
Ovarian Cancer Signaling	8,32E-03
Senescence Pathway	8,51E-03
Tumoricidal Function of Hepatic Natural Killer Cells	8,91E-03
VEGF Signaling	9,33E-03
Role of RIG1-like Receptors in Antiviral Innate Immunity	9,55E-03
G-Protein Coupled Receptor Signaling	9,55E-03
TEC Kinase Signaling	9,77E-03
Oxytocin In Brain Signaling Pathway	1,00E-02
nNOS Signaling in Neurons	1,02E-02
Pathogenesis of Multiple Sclerosis	1,07E-02
Salvage Pathways of Pyrimidine Deoxyribonucleotides	1,07E-02
IL-17A Signaling in Gastric Cells	1,12E-02
Protein Ubiquitination Pathway	1,20E-02
Androgen Signaling	1,20E-02
Breast Cancer Regulation by Stathmin1	1,26E-02
T Helper Cell Differentiation	1,26E-02
Role of MAPK Signaling in Inhibiting the Pathogenesis of Influenza	1,29E-02
UVC-Induced MAPK Signaling	1,35E-02
Neurotrophin/TRK Signaling	1,35E-02
Gαi Signaling	1,41E-02
Insulin Receptor Signaling	1,41E-02
Thyroid Cancer Signaling	1,45E-02
FAK Signaling	1,48E-02
Role of p14/p19ARF in Tumor Suppression	1,51E-02
FLT3 Signaling in Hematopoietic Progenitor Cells	1,66E-02
LPS/IL-1 Mediated Inhibition of RXR Function	1,78E-02
CSDE1 Signaling Pathway	1,86E-02
FcγRIIB Signaling in B Lymphocytes	1,91E-02
Autoimmune Thyroid Disease Signaling	1,91E-02
CNTF Signaling	1,95E-02
Role of NFAT in Cardiac Hypertrophy	2,04E-02
Inhibition of Angiogenesis by TSP1	2,34E-02
Phospholipase C Signaling	2,34E-02
Endothelin-1 Signaling	2,40E-02
IL-2 Signaling	2,45E-02
NUR77 Signaling in T Lymphocytes	2,51E-02
Acyl-CoA Hydrolysis	2,57E-02
Pyroptosis Signaling Pathway	2,69E-02
Non-Small Cell Lung Cancer Signaling	2,82E-02

GP6 Signaling Pathway	2,82E-02
Complement System	2,88E-02
ERB2-ERBB3 Signaling	3,02E-02
Allograft Rejection Signaling	3,16E-02
WNT/Ca+ pathway	3,16E-02
Melanocyte Development and Pigmentation Signaling	3,31E-02
UVA-Induced MAPK Signaling	3,31E-02
SPINK1 General Cancer Pathway	3,63E-02
Neuropathic Pain Signaling In Dorsal Horn Neurons	3,63E-02
Mechanisms of Viral Exit from Host Cells	3,80E-02
Melatonin Signaling	4,17E-02
Calcium-induced T Lymphocyte Apoptosis	4,17E-02
Coronavirus Replication Pathway	4,79E-02
Leptin Signaling in Obesity	4,90E-02

Table S6. The gene signature-linked Upstream Regulators of eDCs (Cheng et al) identified by IPA.
All significant pathways calculated by the IPA-based p values are shown ($p < 0.05$)

**Upstream Regulator (eDC Cheng et al.)**	**Molecule Type**	**p-value of overlap**
TP53	transcription regulator	2,19E-83
TGFB1	growth factor	1,09E-68
beta-estradiol	chemical - endogenous mammalian	2,31E-64
dexamethasone	chemical drug	1,55E-62
lipopolysaccharide	chemical drug	3,05E-60
MYC	transcription regulator	1,50E-59
TNF	cytokine	5,92E-55
KRAS	enzyme	3,71E-43
IFNG	cytokine	3,33E-42
PD98059	chemical - kinase inhibitor	4,48E-41
IgG	complex	9,88E-41
sirolimus	chemical drug	1,67E-40
NFKBIA	transcription regulator	2,52E-40
5-fluorouracil	chemical drug	1,64E-39
CDKN1A	kinase	4,50E-39
IL2	cytokine	1,34E-36
HGF	growth factor	1,46E-36
CD3	complex	1,83E-36
ERBB2	kinase	2,21E-36
IL1B	cytokine	2,22E-36
CDKN2A	transcription regulator	9,20E-36
aflatoxin B1	chemical - endogenous non-mammalian	9,42E-36
ESR1	ligand-dependent nuclear receptor	1,05E-35
HRAS	enzyme	1,23E-35
APP	other	1,26E-35
camptothecin	chemical drug	5,35E-35
E2F1	transcription regulator	8,53E-35
Immunoglobulin	complex	1,42E-34
IL4	cytokine	3,13E-34
E2F4	transcription regulator	4,73E-34
tretinoin	chemical - endogenous mammalian	5,43E-34
CEBPB	transcription regulator	1,72E-33
PDGF BB	complex	2,08E-33
COPS5	transcription regulator	4,65E-33
CD40LG	cytokine	2,26E-32
CTNNB1	transcription regulator	3,10E-32
medroxyprogesterone acetate	chemical drug	6,43E-32
doxorubicin	chemical drug	7,76E-32
CSF2	cytokine	8,67E-32
hydrogen peroxide	chemical - endogenous mammalian	1,49E-31

2-(4-amino-1-isopropyl-1H-pyrazolo[3,4-d]pyrimidin-3-yl)-1H-indol-5-ol	chemical reagent	2,42E-31
TCR	complex	6,14E-31
TP73	transcription regulator	1,21E-30
CD 437	chemical drug	3,58E-30
methylselenic acid	chemical reagent	6,59E-30
LY294002	chemical drug	9,02E-30
ESR2	ligand-dependent nuclear receptor	1,53E-29
calcitriol	chemical drug	4,21E-29
FOXO3	transcription regulator	4,40E-29
ZBTB17	transcription regulator	3,59E-28
Interferon alpha	group	3,83E-28
dihydrotestosterone	chemical - endogenous mammalian	1,17E-27
GLI1	transcription regulator	1,40E-27
EGF	growth factor	1,55E-27
MAPT	other	2,19E-27
tetradecanoylphorbol acetate	chemical drug	3,14E-27
fulvestrant	chemical drug	3,33E-27
IL3	cytokine	5,32E-27
ST1926	chemical drug	8,82E-27
MYCN	transcription regulator	8,95E-27
butyric acid	chemical - endogenous mammalian	1,19E-26
OSM	cytokine	1,31E-26
filgrastim	biologic drug	1,66E-26
TP63	transcription regulator	1,98E-26
8-bromo-cAMP	chemical reagent	2,69E-26
PRL	cytokine	3,93E-26
PGR	ligand-dependent nuclear receptor	6,37E-26
Vegf	group	6,39E-26
STAT3	transcription regulator	6,56E-26
RB1	transcription regulator	7,58E-26
JUN	transcription regulator	2,14E-25
YAP1	transcription regulator	2,26E-25
YY1	transcription regulator	3,43E-25
l-asparaginase	biologic drug	3,88E-25
methylprednisolone	chemical drug	5,39E-25
forskolin	chemical toxicant	8,97E-25
FOS	transcription regulator	1,35E-24
progesterone	chemical - endogenous mammalian	1,45E-24
U0126	chemical drug	1,79E-24
EGFR	kinase	2,37E-24
imatinib	chemical drug	5,93E-24
IL13	cytokine	9,39E-24
IL33	cytokine	1,16E-23
trichostatin A	chemical drug	1,34E-23

arsenic trioxide	chemical drug	2,08E-23
KLF6	transcription regulator	6,24E-23
IL10	cytokine	6,79E-23
Eldr	other	6,79E-23
cisplatin	chemical drug	2,66E-22
NR3C1	ligand-dependent nuclear receptor	4,40E-22
cyclosporin A	biologic drug	4,48E-22
PTEN	phosphatase	4,82E-22
CG	complex	9,13E-22
IL6	cytokine	1,41E-21
NFkB (complex)	complex	1,82E-21
cycloheximide	chemical reagent	2,24E-21
AGN194204	chemical drug	3,41E-21
IL15	cytokine	4,56E-21
TNFSF11	cytokine	6,66E-21
APOE	transporter	6,84E-21
AKT1	kinase	7,55E-21
TCF3	transcription regulator	8,94E-21
SB203580	chemical drug	9,72E-21
CREB1	transcription regulator	1,40E-20
poly rI:rC-RNA	biologic drug	1,55E-20
CASR	G-protein coupled receptor	1,67E-20
Ige	complex	2,09E-20
TCF4	transcription regulator	3,99E-20
LARP1	translation regulator	4,45E-20
IKBKB	kinase	4,81E-20
SMARCA4	transcription regulator	3,25E-19
paclitaxel	chemical drug	3,44E-19
RAF1	kinase	3,56E-19
geldanamycin	chemical drug	4,42E-19
CD44	other	9,18E-19
D-glucose	chemical - endogenous mammalian	9,53E-19
4-hydroxytamoxifen	chemical drug	9,55E-19
fluticasone propionate	chemical drug	1,01E-18
prostaglandin E2	chemical - endogenous mammalian	1,05E-18
dextran sulfate	chemical drug	1,15E-18
BCR (complex)	complex	1,18E-18
HIF1A	transcription regulator	1,70E-18
PSEN1	peptidase	1,85E-18
SPI1	transcription regulator	2,16E-18
ERK1/2	group	3,07E-18
LDL	complex	3,32E-18
Lh	complex	4,80E-18
CD28	transmembrane receptor	5,28E-18

E2F3	transcription regulator	5,28E-18
thapsigargin	chemical toxicant	7,51E-18
FOXM1	transcription regulator	1,34E-17
decitabine	chemical drug	1,65E-17
F2	peptidase	1,66E-17
mir-21	microRNA	1,88E-17
SLC15A4	transporter	2,12E-17
torin1	chemical reagent	2,32E-17
nitrofurantoin	chemical drug	2,32E-17
IGF1	growth factor	2,80E-17
etoposide	chemical drug	3,35E-17
P38 MAPK	group	3,52E-17
FOXO1	transcription regulator	5,04E-17
GPER1	G-protein coupled receptor	6,51E-17
VEGFA	growth factor	6,78E-17
DYSF	other	7,97E-17
ERK	group	8,26E-17
CCND1	transcription regulator	1,03E-16
mifepristone	chemical drug	1,12E-16
FAS	transmembrane receptor	1,20E-16
HSF1	transcription regulator	1,20E-16
EPO	cytokine	1,48E-16
IL27	cytokine	1,78E-16
BCL6	transcription regulator	1,83E-16
MAPK1	kinase	2,31E-16
PI3K (complex)	complex	2,68E-16
NFKB1	transcription regulator	2,68E-16
TFRC	transporter	2,95E-16
tetrachlorodibenzodioxin	chemical toxicant	3,07E-16
gefitinib	chemical drug	3,31E-16
CDK19	kinase	3,58E-16
Z-LLL-CHO	chemical - protease inhibitor	4,31E-16
RELA	transcription regulator	4,58E-16
NUPR1	transcription regulator	5,82E-16
genistein	chemical drug	5,86E-16
CKAP2L	other	6,02E-16
KAT2A	enzyme	6,75E-16
TBX2	transcription regulator	6,79E-16
PC-SPES	chemical drug	8,66E-16
lactacystin	chemical - protease inhibitor	1,16E-15
CSF1	cytokine	1,29E-15
CHUK	kinase	1,30E-15
LDLR	transporter	1,38E-15
FSH	complex	1,38E-15

GRN	growth factor	1,46E-15
CD40	transmembrane receptor	1,52E-15
VDR	transcription regulator	1,56E-15
diethylstilbestrol	chemical drug	2,15E-15
troglitazone	chemical drug	2,42E-15
EIF4E	translation regulator	2,93E-15
metribolone	chemical reagent	3,46E-15
BRCA1	transcription regulator	4,06E-15
AR	ligand-dependent nuclear receptor	4,07E-15
AHR	ligand-dependent nuclear receptor	4,61E-15
INSR	kinase	4,89E-15
RABL6	other	5,10E-15
TAZ	enzyme	5,86E-15
TAS4464	chemical drug	6,13E-15
Salmonella enterica serotype abortus equi lipopolysaccharide	chemical toxicant	6,56E-15
IGF1R	transmembrane receptor	7,00E-15
MTOR	kinase	7,41E-15
NRG1	growth factor	8,01E-15
bee venom	chemical - endogenous non-mammalian	8,66E-15
resiquimod	chemical drug	9,81E-15
AREG	growth factor	1,14E-14
let-7	microRNA	1,21E-14
tanespimycin	chemical drug	1,31E-14
vorinostat	chemical drug	1,49E-14
NR1H3	ligand-dependent nuclear receptor	1,50E-14
MAP2K1/2	group	1,99E-14
Insulin	group	2,05E-14
leukotriene D4	chemical - endogenous mammalian	2,08E-14
bexarotene	chemical drug	2,12E-14
NRAS	enzyme	2,29E-14
IL1A	cytokine	2,46E-14
palbociclib	chemical drug	2,58E-14
ANGPT2	growth factor	3,41E-14
IL5	cytokine	3,47E-14
gentamicin	chemical drug	3,58E-14
ionomycin	chemical reagent	4,03E-14
inosine	chemical - endogenous mammalian	4,51E-14
mitomycin C	chemical drug	4,62E-14
actinomycin D	biologic drug	4,68E-14
curcumin	chemical drug	4,80E-14
ID2	transcription regulator	4,98E-14
cardiotoxin	chemical - other	5,36E-14
26s Proteasome	complex	5,56E-14
romidepsin	biologic drug	5,67E-14

KDM5B	transcription regulator	6,08E-14
CALCA	other	6,68E-14
nitric oxide	chemical - endogenous mammalian	8,09E-14
HNF4A	transcription regulator	8,38E-14
RASSF1	other	9,73E-14
epigallocatechin-gallate	chemical drug	1,02E-13
FGF2	growth factor	1,04E-13
EGR1	transcription regulator	1,12E-13
HOXA9	transcription regulator	1,29E-13
topotecan	chemical drug	1,34E-13
CCL5	cytokine	1,51E-13
vancomycin	biologic drug	1,65E-13
MITF	transcription regulator	1,72E-13
bortezomib	chemical drug	1,72E-13
ELAVL1	other	1,86E-13
PRKCD	kinase	2,04E-13
CEBPA	transcription regulator	2,30E-13
STAT1	transcription regulator	2,47E-13
KDM1A	enzyme	2,60E-13
SP600125	chemical drug	2,66E-13
AGT	growth factor	2,76E-13
PPARA	ligand-dependent nuclear receptor	3,29E-13
GNAQ	enzyme	3,39E-13
triamcinolone acetonide	chemical drug	3,72E-13
wortmannin	chemical drug	5,30E-13
VHL	transcription regulator	5,42E-13
IRF4	transcription regulator	5,61E-13
Igm	complex	5,70E-13
WT1	transcription regulator	6,07E-13
OGA	enzyme	6,23E-13
MAP2K1	kinase	6,87E-13
budesonide	chemical drug	6,91E-13
ETV6-RUNX1	fusion gene/product	7,04E-13
MET	kinase	7,66E-13
H2AZ1	other	7,73E-13
5-azacytidine	chemical drug	8,14E-13
TERT	enzyme	8,36E-13
Akt	group	8,76E-13
NGF	growth factor	9,94E-13
MYOD1	transcription regulator	1,19E-12
IRAK4	kinase	1,55E-12
salmonella minnesota R595 lipopolysaccharides	chemical - endogenous non-mammalian	1,61E-12
PAX3-FOXO1	fusion gene/product	1,77E-12
TFAP2A	transcription regulator	1,78E-12

HTT	transcription regulator	2,00E-12
deferoxamine	chemical drug	2,01E-12
rosiglitazone	chemical drug	2,04E-12
tacrolimus	chemical drug	2,10E-12
concanavalin a	chemical drug	2,90E-12
PTGER2	G-protein coupled receptor	2,99E-12
RICTOR	other	3,25E-12
SP1	transcription regulator	3,85E-12
SIRT1	transcription regulator	4,46E-12
PPARG	ligand-dependent nuclear receptor	4,54E-12
NFE2L2	transcription regulator	4,62E-12
MHC II	group	4,95E-12
BDNF	growth factor	5,04E-12
black raspberry extract	chemical drug	5,17E-12
DUSP1	phosphatase	5,58E-12
CSF3	cytokine	5,82E-12
GNB1	enzyme	5,99E-12
HAVCR1	other	7,04E-12
IFNA2	cytokine	7,44E-12
GnRH analog	biologic drug	7,69E-12
RNA polymerase II	complex	7,77E-12
IFI16	transcription regulator	8,40E-12
EPHA2	kinase	8,44E-12
TLR4	transmembrane receptor	8,76E-12
Jnk	group	8,84E-12
Ap1	complex	1,12E-11
Tgf beta	group	1,17E-11
ADRB	group	1,18E-11
HDAC1	transcription regulator	1,21E-11
IL17A	cytokine	1,21E-11
RRP1B	transcription regulator	1,21E-11
Mek	group	1,27E-11
tributyrin	chemical drug	1,62E-11
TSC2	other	1,68E-11
EIF2AK2	kinase	1,69E-11
TNFSF10	cytokine	1,72E-11
n-nitrosomethylbenzylamine	chemical toxicant	1,72E-11
raloxifene	chemical drug	2,06E-11
pirinixic acid	chemical toxicant	2,07E-11
ATF3	transcription regulator	2,16E-11
PTP4A1	phosphatase	2,45E-11
BTK	kinase	2,45E-11
BMS-690514	chemical drug	2,48E-11
GNA15	enzyme	2,67E-11

SMARCB1	transcription regulator	2,67E-11
TGFA	growth factor	2,75E-11
acyline	biologic drug	2,85E-11
GnRH-A	chemical reagent	2,95E-11
FOXP3	transcription regulator	2,97E-11
trans-hydroxytamoxifen	chemical drug	2,97E-11
CREM	transcription regulator	3,06E-11
mibolerone	chemical drug	3,38E-11
IL1	group	3,53E-11
cuprizone	chemical toxicant	3,71E-11
SNAI1	transcription regulator	3,75E-11
valproic acid	chemical drug	3,75E-11
panobinostat	chemical drug	4,07E-11
L-methionine	chemical - endogenous mammalian	4,07E-11
REL	transcription regulator	4,49E-11
NPM1	transcription regulator	4,59E-11
BHLHE40	transcription regulator	4,64E-11
tamoxifen	chemical drug	4,64E-11
PRKCE	kinase	4,75E-11
mycophenolic acid	chemical drug	5,13E-11
TNFSF13B	cytokine	5,46E-11
IGF2	growth factor	5,54E-11
ID3	transcription regulator	5,58E-11
5-N-ethylcarboxamido adenosine	chemical reagent	5,69E-11
ATM	kinase	5,85E-11
miR-124-3p (and other miRNAs w/seed AAGGCAC)	mature microRNA	5,86E-11
EDN1	cytokine	6,17E-11
carbon tetrachloride	chemical toxicant	7,13E-11
quercetin	chemical drug	8,95E-11
nicotine	chemical drug	8,95E-11
kainic acid	chemical toxicant	8,98E-11
2-bromoethylamine	chemical reagent	9,41E-11
indomethacin	chemical drug	9,89E-11
N-acetyl-L-cysteine	chemical drug	9,89E-11
INS	other	1,01E-10
bisindolylmaleimide I	chemical drug	1,32E-10
KITLG	growth factor	1,33E-10
TWIST1	transcription regulator	1,48E-10
tunicamycin	chemical - endogenous non-mammalian	1,53E-10
ERG	transcription regulator	1,60E-10
MAX	transcription regulator	1,64E-10
okadaic acid	chemical toxicant	1,64E-10
FLCN	other	1,74E-10
GABA	chemical - endogenous mammalian	1,77E-10

ZBTB16	transcription regulator	1,78E-10
IPMK	kinase	1,81E-10
JUNB	transcription regulator	1,86E-10
SMAD7	transcription regulator	1,86E-10
PGF	growth factor	1,91E-10
TGFBR2	kinase	1,95E-10
CST5	other	2,03E-10
IL21	cytokine	2,05E-10
FBXW7	enzyme	2,13E-10
FUS	transcription regulator	2,13E-10
ERBB3	kinase	2,14E-10
uranyl nitrate	chemical toxicant	2,39E-10
GNB2	enzyme	2,53E-10
tyrphostin AG490	chemical drug	2,62E-10
palmitic acid	chemical - endogenous mammalian	2,68E-10
E. coli B5 lipopolysaccharide	chemical - endogenous non-mammalian	2,71E-10
simvastatin	chemical drug	2,85E-10
ILF3	transcription regulator	2,93E-10
SNCA	enzyme	2,94E-10
MAPK7	kinase	2,97E-10
H89	chemical drug	3,24E-10
vinorelbine	chemical drug	3,63E-10
RARA	ligand-dependent nuclear receptor	3,64E-10
RC3H1	enzyme	3,70E-10
miR-17-5p (and other miRNAs w/seed AAAGUGC)	mature microRNA	3,86E-10
Tcf7	transcription regulator	3,88E-10
glutamine	chemical - endogenous mammalian	3,89E-10
GAST	other	3,95E-10
BCL3	transcription regulator	3,97E-10
IRF8	transcription regulator	4,14E-10
ACOX1	enzyme	4,14E-10
POU5F1	transcription regulator	4,22E-10
TLR3	transmembrane receptor	4,74E-10
allopurinol	chemical drug	4,74E-10
RGS2	enzyme	4,74E-10
CDH1	other	5,24E-10
EML4-ALK	fusion gene/product	5,24E-10
estrogen	chemical drug	5,79E-10
NKX2-3	transcription regulator	5,92E-10
NR3C2	ligand-dependent nuclear receptor	6,16E-10
STAT6	transcription regulator	6,21E-10
tosedostat	chemical drug	6,48E-10
NFYA	transcription regulator	6,91E-10
discodermolide	chemical drug	7,03E-10

SP110	transcription regulator	7,54E-10
sorafenib	chemical drug	7,83E-10
MYBL2	transcription regulator	7,91E-10
NOTCH1	transcription regulator	8,15E-10
ADCYAP1	other	8,34E-10
NFATC2	transcription regulator	8,66E-10
prexasertib	chemical drug	8,80E-10
PTGS2	enzyme	9,03E-10
COL18A1	other	9,07E-10
Creb	group	9,47E-10
RBL2	other	1,01E-09
Histone h3	group	1,09E-09
potassium chloride	chemical drug	1,14E-09
diclofenac	chemical drug	1,19E-09
cerivastatin	chemical drug	1,25E-09
Irgm1	other	1,42E-09
puromycin aminonucleoside	chemical reagent	1,43E-09
ARNT	transcription regulator	1,46E-09
ATF4	transcription regulator	1,55E-09
calcimycin	chemical reagent	1,55E-09
5-O-mycolyl-beta-araf-(1->2)-5-O-mycolyl-alpha-araf-(1->1')-glycerol	chemical - endogenous non-mammalian	1,63E-09
interferon beta-1a	biologic drug	1,63E-09
peptidoglycan	chemical - endogenous non-mammalian	1,80E-09
F7	peptidase	1,81E-09
QKI	other	1,88E-09
PPARGC1A	transcription regulator	2,03E-09
PELP1	other	2,04E-09
semaxinib	chemical drug	2,04E-09
triamterene	chemical drug	2,10E-09
mir-15	microRNA	2,13E-09
PPP1R13L	transcription regulator	2,14E-09
LONP1	peptidase	2,21E-09
hyaluronic acid	chemical - endogenous mammalian	2,40E-09
testosterone	chemical - endogenous mammalian	2,42E-09
ACTL6A	other	2,43E-09
CNTF	cytokine	2,52E-09
FN1	enzyme	2,52E-09
hydrocortisone	chemical - endogenous mammalian	2,64E-09
KLF4	transcription regulator	2,72E-09
bleomycin	biologic drug	2,73E-09
fenamic acid	chemical reagent	2,86E-09
estrogen receptor	group	2,88E-09
phytohemagglutinin	chemical drug	2,92E-09
cyclophosphamide	chemical drug	2,97E-09

ibrutinib	chemical drug	3,01E-09
bicuculline	chemical - endogenous non-mammalian	3,19E-09
TAL1	transcription regulator	3,43E-09
PIK3R1	kinase	3,61E-09
FOSL1	transcription regulator	3,61E-09
AMPK	complex	3,67E-09
SRC	kinase	3,73E-09
PLK1	kinase	3,74E-09
eicosapentenoic acid	chemical drug	3,77E-09
NTRK2	kinase	3,83E-09
resveratrol	chemical drug	3,91E-09
ADRA1D	G-protein coupled receptor	4,16E-09
S100A6	transporter	4,38E-09
TCL1A	transcription regulator	4,67E-09
niacinamide	chemical - endogenous mammalian	4,67E-09
lomustine	chemical drug	4,99E-09
fluoride	chemical - endogenous mammalian	5,01E-09
FOXC1	transcription regulator	5,07E-09
PLG	peptidase	5,21E-09
PML	transcription regulator	5,28E-09
MDM2	transcription regulator	5,40E-09
arsenite	chemical toxicant	5,42E-09
staurosporine	chemical drug	5,56E-09
PAX3	transcription regulator	5,72E-09
Growth hormone	group	5,86E-09
TGM2	enzyme	6,02E-09
Pkc(s)	group	6,03E-09
docosahexaenoic acid	chemical drug	6,11E-09
PAF1	other	6,12E-09
Hdac	group	6,25E-09
BNIP3L	other	6,27E-09
bucladesine	chemical toxicant	6,46E-09
Rb	group	6,52E-09
MMP9	peptidase	6,70E-09
NLRP3	other	6,86E-09
KLF5	transcription regulator	6,86E-09
entinostat	chemical drug	6,87E-09
NFYB	transcription regulator	6,89E-09
STAT5A	transcription regulator	7,07E-09
lovastatin	chemical drug	7,24E-09
SREBF1	transcription regulator	7,44E-09
ARVib-7	chemical reagent	7,62E-09
ARVib-31	chemical reagent	7,62E-09
TREM1	transmembrane receptor	7,74E-09

telapristone acetate	chemical drug	7,81E-09
MAPK9	kinase	7,93E-09
raltitrexed	chemical drug	8,18E-09
IRF1	transcription regulator	8,99E-09
PTH	other	9,32E-09
cytokine	group	1,03E-08
mir-8	microRNA	1,04E-08
IL12 (complex)	complex	1,06E-08
PTPRJ	phosphatase	1,07E-08
dopamine	chemical - endogenous mammalian	1,09E-08
ASPSCR1-TFE3	fusion gene/product	1,09E-08
RUNX1	transcription regulator	1,13E-08
IKBKG	kinase	1,16E-08
15-deoxy-delta-12,14 -PGJ 2	chemical - endogenous mammalian	1,16E-08
XBP1	transcription regulator	1,16E-08
1-methyl-4-phenyl-1,2,3,6-tetrahydropyridine	chemical toxicant	1,17E-08
SOCS1	other	1,39E-08
CCN1	other	1,40E-08
NS-398	chemical reagent	1,46E-08
fluocinolone acetonide	chemical drug	1,50E-08
DDIT3	transcription regulator	1,50E-08
NORAD	other	1,55E-08
RET	kinase	1,56E-08
oxaliplatin	chemical drug	1,62E-08
ECSIT	transcription regulator	1,65E-08
STK11	kinase	1,69E-08
Pdgf (complex)	complex	1,72E-08
HNF1A-AS1	other	1,73E-08
diethylnitrosamine	chemical toxicant	1,75E-08
CD24	other	1,81E-08
TMPRSS2-ERG	fusion gene/product	1,81E-08
ADRA1A	G-protein coupled receptor	1,82E-08
miR-155-5p (miRNAs w/seed UAAUGCU)	mature microRNA	1,84E-08
thioacetamide	chemical toxicant	1,88E-08
MYD88	other	2,03E-08
MAP2K4	kinase	2,07E-08
methyl methanesulfonate	chemical toxicant	2,07E-08
SPP1	cytokine	2,10E-08
VCAN	other	2,17E-08
ATF6	transcription regulator	2,19E-08
gentamicin C	chemical drug	2,19E-08
EIF2AK3	kinase	2,20E-08
caffeic acid phenethyl ester	chemical drug	2,23E-08
CLDN7	other	2,25E-08

NPC1	transporter	2,27E-08
SELP	transmembrane receptor	2,37E-08
ADGRE2	other	2,37E-08
Tnf (family)	group	2,46E-08
ammonium	chemical - endogenous mammalian	2,46E-08
TPH1	enzyme	2,54E-08
BCOR	transcription regulator	2,56E-08
SMAD3	transcription regulator	2,73E-08
RBL1	transcription regulator	2,76E-08
TFAP2C	transcription regulator	2,76E-08
CXCL12	cytokine	2,84E-08
IL9	cytokine	2,84E-08
anisomycin	chemical - endogenous non-mammalian	2,84E-08
prednisolone	chemical drug	2,85E-08
CDK1	kinase	2,86E-08
entolimod	biologic drug	2,86E-08
sulindac	chemical drug	2,95E-08
TREX1	enzyme	3,08E-08
EP300	transcription regulator	3,10E-08
Pam3-Cys-Ser-Lys4	chemical reagent	3,11E-08
Ifn gamma	complex	3,14E-08
RAS	group	3,18E-08
Ca2+	chemical - endogenous mammalian	3,18E-08
JQ1	chemical reagent	3,31E-08
mir-1	microRNA	3,37E-08
deoxycholate	chemical - endogenous mammalian	3,58E-08
BCR-ABL1	fusion gene/product	3,64E-08
TCF	group	3,64E-08
O6-benzylguanine	chemical drug	3,82E-08
S-nitroso-N-acetyl-DL-penicillamine	chemical reagent	3,87E-08
dactolisib	chemical drug	3,93E-08
IL7	cytokine	3,97E-08
PLX5622	chemical drug	3,97E-08
homocysteine	chemical - endogenous mammalian	4,14E-08
PP2/AG1879 tyrosine kinase inhibitor	chemical drug	4,14E-08
Histone h4	group	4,69E-08
MRTFB	transcription regulator	4,75E-08
SOD1	enzyme	4,94E-08
MAPK3	kinase	5,02E-08
diphenyleneiodonium	chemical reagent	5,21E-08
dalfampridine	chemical drug	5,22E-08
LYN	kinase	5,25E-08
glucocorticoid	chemical drug	5,51E-08
SHH	peptidase	5,75E-08

lysophosphatidic acid	chemical - other	6,03E-08
IL11RA	transmembrane receptor	6,13E-08
glutamyl-Se-methylselenocysteine	chemical - endogenous mammalian	6,44E-08
LIN9	other	7,01E-08
ZFP36	transcription regulator	7,16E-08
desmopressin	biologic drug	7,16E-08
STAT5B	transcription regulator	7,89E-08
VCP	enzyme	7,94E-08
benzo(a)pyrene	chemical toxicant	8,08E-08
ethionine	chemical toxicant	8,20E-08
cigarette smoke	chemical toxicant	8,34E-08
G protein alphai	group	8,36E-08
IL22	cytokine	8,44E-08
trabectedin	chemical drug	8,54E-08
methylnitrosourea	chemical toxicant	8,54E-08
TIMP3	other	8,55E-08
E. coli B4 lipopolysaccharide	chemical toxicant	8,67E-08
SRF	transcription regulator	8,74E-08
WNT3A	cytokine	8,74E-08
PRDM1	transcription regulator	8,95E-08
methotrexate	chemical drug	8,99E-08
ciprofloxacin	chemical drug	9,05E-08
IL32	cytokine	9,22E-08
seocalcitol	chemical drug	9,33E-08
CD38	enzyme	9,49E-08
IRF3	transcription regulator	9,77E-08
CDK4	kinase	9,96E-08
aspirin	chemical drug	1,00E-07
spermine	chemical - endogenous mammalian	1,03E-07
temozolomide	chemical drug	1,04E-07
ELK1	transcription regulator	1,07E-07
GATA1	transcription regulator	1,11E-07
ELOVL3	enzyme	1,17E-07
SNAI2	transcription regulator	1,17E-07
IRS1	enzyme	1,18E-07
RAC2	enzyme	1,18E-07
napabucasin	chemical drug	1,23E-07
PTPN6	phosphatase	1,24E-07
PTX3	other	1,27E-07
stearic acid	chemical - endogenous mammalian	1,27E-07
ST3-Hel2A-2	chemical reagent	1,31E-07
2-deoxyglucose	chemical drug	1,31E-07
KLF2	transcription regulator	1,34E-07
MXI1	transcription regulator	1,35E-07

EPAS1	transcription regulator	1,44E-07
ZFTA-RELA	fusion gene/product	1,47E-07
aldesleukin	biologic drug	1,47E-07
TRAF2	enzyme	1,53E-07
bromodeoxyuridine	chemical drug	1,62E-07
nitroprusside	chemical drug	1,62E-07
MAPKAPK2	kinase	1,66E-07
ethanol	chemical - endogenous mammalian	1,67E-07
6-hydroxydopamine	chemical toxicant	1,68E-07
CpG oligonucleotide	chemical drug	1,78E-07
acetaminophen	chemical drug	1,83E-07
IFN Beta	group	1,93E-07
GNA14	enzyme	1,95E-07
PLAU	peptidase	1,95E-07
clopidogrel	chemical drug	1,95E-07
SOX2	transcription regulator	1,98E-07
Fc gamma receptor	group	2,00E-07
PRKAG3	kinase	2,02E-07
PRKAA1	kinase	2,05E-07
S100A8	other	2,05E-07
IFNB1	cytokine	2,09E-07
Pka	complex	2,10E-07
infliximab	biologic drug	2,12E-07
imiquimod	chemical drug	2,18E-07
BAX	transporter	2,20E-07
RIPK1	kinase	2,20E-07
MFAP5	other	2,20E-07
phenylbutazone	chemical drug	2,21E-07
rottlerin	chemical drug	2,21E-07
BRD4	kinase	2,21E-07
HOXA10	transcription regulator	2,35E-07
NAE1	enzyme	2,40E-07
SMC3	other	2,40E-07
IKZF1	transcription regulator	2,43E-07
triptolide	chemical drug	2,47E-07
Cdc42	enzyme	2,49E-07
1,2-dithiol-3-thione	chemical reagent	2,50E-07
KDM3B	enzyme	2,50E-07
GFI1	transcription regulator	2,59E-07
STAT4	transcription regulator	2,59E-07
fenofibrate	chemical drug	2,63E-07
SATB1	transcription regulator	2,71E-07
EWSR1-FLI1	fusion gene/product	2,79E-07
CP-55940	chemical reagent	2,87E-07

KRT14	other	2,87E-07
PAK2	kinase	2,87E-07
TNFRSF8	transmembrane receptor	2,92E-07
TAF6	transcription regulator	2,93E-07
PPARD	ligand-dependent nuclear receptor	2,94E-07
oblimersen	biologic drug	2,96E-07
fluoromethyl 2,2-difluoro-1-(trifluoromethyl)vinyl ether	chemical toxicant	3,07E-07
HLX	transcription regulator	3,07E-07
TAB1	enzyme	3,07E-07
mono-(2-ethylhexyl)phthalate	chemical toxicant	3,12E-07
Map3k7	kinase	3,19E-07
sulindac sulfide	chemical drug	3,19E-07
Sb202190	chemical drug	3,21E-07
TCF7L2	transcription regulator	3,46E-07
PRNP	other	3,50E-07
ELANE	peptidase	3,52E-07
MKNK1	kinase	3,59E-07
TSC1	other	3,76E-07
MAPK14	kinase	3,80E-07
FASLG	cytokine	3,88E-07
zVAD-FMK	chemical - protease inhibitor	3,88E-07
PDX1	transcription regulator	3,93E-07
mir-223	microRNA	3,95E-07
PD184352	chemical drug	4,02E-07
HMGA1	transcription regulator	4,04E-07
E. coli lipopolysaccharide	chemical - endogenous non-mammalian	4,04E-07
GAPDH	enzyme	4,17E-07
lenalidomide	chemical drug	4,27E-07
PIN1	enzyme	4,38E-07
F2R	G-protein coupled receptor	4,53E-07
E2f	group	4,55E-07
PRKCA	kinase	4,55E-07
Raf	group	4,56E-07
EFNA1	other	4,56E-07
trans-cinnamaldehyde	chemical drug	4,66E-07
MAP3K8	kinase	4,72E-07
androgen	chemical drug	4,77E-07
IL1RN	cytokine	4,90E-07
baicalein	chemical drug	4,91E-07
Fcer1	complex	4,99E-07
TLR2	transmembrane receptor	5,09E-07
epothilone B	chemical drug	5,11E-07
anacardic acid	chemical - endogenous non-mammalian	5,11E-07
ZFP91	transcription regulator	5,11E-07

spermidine	chemical - endogenous mammalian	5,11E-07
CX3CL1	cytokine	5,17E-07
hexachlorobenzene	chemical toxicant	5,25E-07
ITGB2	transmembrane receptor	5,26E-07
SB 216763	chemical drug	5,26E-07
miR-16-5p (and other miRNAs w/seed AGCAGCA)	mature microRNA	5,27E-07
NFKB2	transcription regulator	5,36E-07
atorvastatin	chemical drug	5,62E-07
sodium arsenite	chemical drug	5,82E-07
bromobenzene	chemical toxicant	5,82E-07
LEP	growth factor	5,90E-07
laminaran	chemical drug	5,98E-07
NOS2	enzyme	5,99E-07
CCL2	cytokine	6,26E-07
INSIG1	other	6,26E-07
RBPJ	transcription regulator	6,33E-07
pembrolizumab	biologic drug	6,79E-07
CDK2	kinase	6,79E-07
GDF2	growth factor	6,81E-07
RHOA	enzyme	6,81E-07
CLEC11A	growth factor	6,91E-07
carrageenan	chemical drug	7,07E-07
APC	enzyme	7,19E-07
IL18	cytokine	7,43E-07
PI3K (family)	group	7,47E-07
SB-431542	chemical reagent	7,90E-07
arachidonic acid	chemical - endogenous mammalian	7,92E-07
ETV5	transcription regulator	8,25E-07
clofibrate	chemical drug	8,25E-07
10E,12Z-octadecadienoic acid	chemical - endogenous mammalian	8,41E-07
isobutylmethylxanthine	chemical toxicant	8,50E-07
CHEK1	kinase	8,84E-07
ADAP1	other	8,89E-07
PCLAF	other	8,89E-07
cyclic AMP	chemical - endogenous mammalian	9,22E-07
TEAD4	transcription regulator	9,49E-07
Sos	group	9,50E-07
STING1	other	9,70E-07
PIK3CA	kinase	9,99E-07
Y 27632	chemical drug	9,99E-07
captopril	chemical drug	9,99E-07
fenretinide	chemical drug	9,99E-07
MDK	growth factor	1,00E-06
DDX3X	enzyme	1,00E-06

PXDN	enzyme	1,01E-06
PPP2R5C	other	1,01E-06
NRP1	transmembrane receptor	1,01E-06
LGALS1	other	1,03E-06
POU2AF1	transcription regulator	1,03E-06
T-5224	chemical reagent	1,06E-06
mir-154	microRNA	1,08E-06
tazemetostat	chemical drug	1,11E-06
ARID1A	transcription regulator	1,12E-06
HNF1B	transcription regulator	1,14E-06
ROCK2	kinase	1,17E-06
HSP90B1	other	1,17E-06
MRTFA	transcription regulator	1,18E-06
KDM3A	transcription regulator	1,18E-06
SAA1	transporter	1,18E-06
BAG1	other	1,18E-06
phenacetin	chemical drug	1,18E-06
DETA-NONOate	chemical reagent	1,25E-06
ADRA1B	G-protein coupled receptor	1,26E-06
MUC1	other	1,26E-06
CD244	transmembrane receptor	1,28E-06
JAK2	kinase	1,28E-06
melatonin	chemical - endogenous mammalian	1,28E-06
BMP4	growth factor	1,30E-06
CEBPD	transcription regulator	1,35E-06
ZC3H12A	enzyme	1,43E-06
alvocidib	chemical drug	1,44E-06
MLXIPL	transcription regulator	1,48E-06
NFAT5	transcription regulator	1,48E-06
TFEB	transcription regulator	1,51E-06
cadmium chloride	chemical toxicant	1,51E-06
cis-urocanic acid	chemical drug	1,51E-06
farnesol	chemical reagent	1,51E-06
SCD	enzyme	1,51E-06
CSF	group	1,51E-06
HIC1	transcription regulator	1,51E-06
CREBBP	transcription regulator	1,52E-06
FADD	other	1,54E-06
SELPLG	other	1,63E-06
everolimus	chemical drug	1,63E-06
alefacept	biologic drug	1,64E-06
CDK9	kinase	1,64E-06
NOX4	enzyme	1,64E-06
carboplatin	chemical drug	1,64E-06

APEX1	enzyme	1,64E-06
ritonavir	chemical drug	1,69E-06
Z36	chemical reagent	1,70E-06
hydroxyurea	chemical drug	1,71E-06
trovafloxacin	chemical drug	1,71E-06
KLF3	transcription regulator	1,71E-06
MAPK8	kinase	1,79E-06
cholesterol	chemical - endogenous mammalian	1,79E-06
BTNL2	transmembrane receptor	1,83E-06
E2F2	transcription regulator	1,83E-06
25-hydroxycholesterol	chemical reagent	1,83E-06
celecoxib	chemical drug	1,85E-06
isoproterenol	chemical drug	1,86E-06
MAP3K14	kinase	1,88E-06
PLC	group	1,89E-06
KLK4	peptidase	1,89E-06
CYB561A3	enzyme	1,89E-06
APOC1	transporter	1,89E-06
superoxide	chemical - endogenous mammalian	1,89E-06
silicon dioxide	chemical drug	1,89E-06
Ptprd	phosphatase	1,94E-06
L2HGDH	enzyme	1,94E-06
N-(3-(aminomethyl)benzyl)acetamidine	chemical reagent	2,03E-06
LMO2	transcription regulator	2,04E-06
uric acid	chemical - endogenous mammalian	2,05E-06
RAC1	enzyme	2,05E-06
PRKAA	group	2,13E-06
BIRC5	other	2,13E-06
STAT5a/b	group	2,16E-06
ZNF106	other	2,23E-06
2-amino-5-phosphonovaleric acid	chemical - other	2,26E-06
ETS1	transcription regulator	2,35E-06
imipramine blue	chemical drug	2,40E-06
SUPT20H	other	2,49E-06
reactive oxygen species	chemical toxicant	2,52E-06
amino acids	chemical - endogenous mammalian	2,61E-06
cobalt chloride	chemical reagent	2,62E-06
caffeine	chemical drug	2,70E-06
CASP8	peptidase	2,76E-06
MAP2K7	kinase	2,76E-06
NKX3-1	transcription regulator	2,76E-06
CD5	transmembrane receptor	2,76E-06
EFNA2	kinase	2,76E-06
dinoprost	chemical - endogenous mammalian	2,81E-06

TGFBR1	kinase	2,83E-06
CARM1	transcription regulator	2,84E-06
piceatannol	chemical - endogenous non-mammalian	2,84E-06
GSK3B	kinase	2,93E-06
EFNA5	kinase	2,96E-06
miR-483-3p (miRNAs w/seed CACUCCU)	mature microRNA	3,01E-06
HDAC3	transcription regulator	3,13E-06
Sn50 peptide	chemical toxicant	3,14E-06
SP2509	chemical reagent	3,15E-06
CHRM3	G-protein coupled receptor	3,29E-06
TRAF7	enzyme	3,29E-06
metronidazole	chemical drug	3,31E-06
4-nitroquinoline-1-oxide	chemical toxicant	3,35E-06
miR-125b-5p (and other miRNAs w/seed CCCUGAG)	mature microRNA	3,45E-06
AZ-1	chemical - protease inhibitor	3,45E-06
RELB	transcription regulator	3,45E-06
oleic acid	chemical - endogenous mammalian	3,45E-06
miR-24-3p (and other miRNAs w/seed GGCUCAG)	mature microRNA	3,46E-06
ZBTB7A	transcription regulator	3,46E-06
FHIT	enzyme	3,46E-06
SND1	enzyme	3,46E-06
GNRH1	other	3,55E-06
benzene	chemical toxicant	3,55E-06
S100A9	other	3,65E-06
SYK	kinase	3,69E-06
roscovitine	chemical drug	3,87E-06
PRKDC	kinase	3,92E-06
chrysotile asbestos	chemical toxicant	3,92E-06
LIF	cytokine	3,94E-06
zerumbone	chemical - endogenous non-mammalian	3,99E-06
PTAFR	G-protein coupled receptor	3,99E-06
DRAP1	transcription regulator	3,99E-06
L-glutamic acid	chemical - endogenous mammalian	3,99E-06
stallimycin	biologic drug	4,03E-06
eflornithine	chemical drug	4,03E-06
hydroxypropyl-beta-cyclodextrin	chemical drug	4,17E-06
NSUN3	enzyme	4,29E-06
LDB1	transcription regulator	4,39E-06
ATF2	transcription regulator	4,77E-06
colistin	biologic drug	4,80E-06
CITED2	transcription regulator	4,87E-06
DICER1	enzyme	4,90E-06
EZR	other	4,91E-06
mir-214	microRNA	4,91E-06

BCYRN1	other	4,91E-06
ITGAM	transmembrane receptor	4,91E-06
NDRG1	kinase	4,95E-06
methylmercury	chemical toxicant	4,95E-06
enalapril	biologic drug	5,02E-06
histamine	chemical - endogenous mammalian	5,02E-06
SPHK1	kinase	5,03E-06
17-alpha-ethinylestradiol	chemical drug	5,07E-06
PP1	chemical drug	5,35E-06
mitoxantrone	chemical drug	5,35E-06
LGALS3	other	5,65E-06
PKNOX2	transcription regulator	5,69E-06
BID	other	5,69E-06
TP53COR1	other	5,69E-06
S-(2,3-bispalmitoyloxypropyl)-cysteine-GDPKHPKSF	chemical reagent	5,69E-06
METTL3	enzyme	5,78E-06
ATP-gamma-S	chemical reagent	6,18E-06
BMI1	transcription regulator	6,18E-06
SDCBP	enzyme	6,20E-06
Nfat (family)	group	6,23E-06
MAP3K1	kinase	6,49E-06
RBM20	other	6,63E-06
kanamycin A	chemical drug	6,63E-06
BMP2	growth factor	6,66E-06
LEPR	transmembrane receptor	6,68E-06
TLR9	transmembrane receptor	6,73E-06
FOLR1	transporter	6,78E-06
EHF	transcription regulator	6,78E-06
daidzein	chemical drug	6,78E-06
IL12 (family)	group	6,79E-06
PLAT	peptidase	6,86E-06
EWSR1	other	6,86E-06
SOX11	transcription regulator	6,95E-06
mir-155	microRNA	6,95E-06
NFkB (family)	group	7,03E-06
MASTL	kinase	7,03E-06
H2AX	transcription regulator	7,03E-06
PIK3CD	kinase	7,03E-06
hexamethylene bisacetamide	chemical reagent	7,03E-06
N-formyl-Met-Leu-Phe	chemical reagent	7,03E-06
EFNA4	kinase	7,22E-06
PDPK1	kinase	7,22E-06
ABL1	kinase	7,22E-06
GAS2L3	other	7,25E-06

mevastatin	chemical drug	7,25E-06
ALKBH5	enzyme	7,25E-06
beraprost	chemical drug	7,38E-06
clorgyline	chemical drug	7,38E-06
SR 144528	chemical reagent	7,38E-06
1-((2-chlorophenyl)diphenylmethyl)-1H-pyrazole	chemical reagent	7,38E-06
cytarabine	chemical drug	7,67E-06
INHBA	growth factor	7,74E-06
gemcitabine	chemical drug	7,76E-06
GATA3	transcription regulator	8,19E-06
SAFB	other	8,30E-06
TRPV4	ion channel	8,35E-06
CFLAR	other	8,35E-06
SYVN1	transporter	9,08E-06
PTGES	enzyme	9,11E-06
EZH2	transcription regulator	9,16E-06
nilotinib	chemical drug	9,17E-06
GH1	growth factor	9,28E-06
SIN3B	transcription regulator	9,30E-06
idarubicin	chemical drug	9,30E-06
MDL 73811	chemical reagent	9,30E-06
PALMD	other	9,40E-06
arotinoid acid	chemical toxicant	9,40E-06
ozone	chemical toxicant	9,46E-06
HOXD10	transcription regulator	9,47E-06
calphostin C	chemical drug	9,47E-06
EFNA3	kinase	9,65E-06
CLOCK	transcription regulator	9,82E-06
promegestone	chemical drug	1,03E-05
TICAM1	other	1,04E-05
CAT	enzyme	1,06E-05
RPS15	other	1,06E-05
9,10-dimethyl-1,2-benzanthracene	chemical toxicant	1,06E-05
Alpha catenin	group	1,07E-05
berberine	chemical drug	1,07E-05
FGF1	growth factor	1,08E-05
dimethyl sulfoxide	chemical drug	1,08E-05
FBXO32	enzyme	1,10E-05
PLCG2	enzyme	1,15E-05
PDCD1	transmembrane receptor	1,16E-05
CTLA4	transmembrane receptor	1,16E-05
TREM2	transmembrane receptor	1,16E-05
SCH 58261	chemical reagent	1,19E-05
collagenase	group	1,19E-05

AS1842856	chemical reagent	1,19E-05
bendamustine	chemical drug	1,20E-05
SMAD1/5	group	1,20E-05
miR-183-5p (miRNAs w/seed AUGGCAC)	mature microRNA	1,20E-05
MED15	transcription regulator	1,20E-05
SP3	transcription regulator	1,21E-05
methapyrilene	chemical drug	1,21E-05
LCN2	transporter	1,23E-05
TNC	other	1,28E-05
leukotriene C4	chemical - endogenous mammalian	1,28E-05
pCPT-cAMP	chemical - kinase inhibitor	1,28E-05
AKT inhibitor VIII	chemical reagent	1,29E-05
EIF2AK4	kinase	1,29E-05
lisinopril	biologic drug	1,29E-05
5-fluoro-2-hydroxycinnamaldehyde	chemical reagent	1,32E-05
5-fluoro-2-benzoyloxycinnamaldehyde	chemical reagent	1,32E-05
SERCA	group	1,32E-05
vanillyl-N-nonylamide	chemical drug	1,32E-05
mir-488	microRNA	1,32E-05
EEF2K	kinase	1,32E-05
6,7-dinitroquinoxaline-2,3-dione	chemical reagent	1,32E-05
Pln	other	1,33E-05
Notch	group	1,34E-05
sulforafan	chemical drug	1,34E-05
BMP7	growth factor	1,34E-05
CXCL8	cytokine	1,39E-05
HSPB1	other	1,39E-05
EPCAM	other	1,39E-05
SIN3A	transcription regulator	1,41E-05
PLN	transporter	1,41E-05
CDK4/6	group	1,43E-05
silibinin	chemical drug	1,43E-05
GW3965	chemical reagent	1,43E-05
PTGER4	G-protein coupled receptor	1,45E-05
CAV1	transmembrane receptor	1,45E-05
DOT1L	phosphatase	1,47E-05
V-PYRRO/NO	chemical reagent	1,47E-05
selenomethylselenocysteine	chemical - endogenous mammalian	1,47E-05
E2F8	transcription regulator	1,49E-05
CHRM1	G-protein coupled receptor	1,49E-05
TLE1	transcription regulator	1,49E-05
DAP3	other	1,49E-05
RPL22	translation regulator	1,49E-05
HSPA1A/HSPA1B	enzyme	1,49E-05

F2RL1	G-protein coupled receptor	1,56E-05
FOXL2	transcription regulator	1,58E-05
apigenin	chemical - endogenous non-mammalian	1,58E-05
docetaxel	chemical drug	1,62E-05
Cdk	group	1,66E-05
vitamin K3	chemical drug	1,66E-05
hymecromone	chemical drug	1,66E-05
PLAUR	transmembrane receptor	1,67E-05
GPX1	enzyme	1,67E-05
FLT3	kinase	1,67E-05
NME1	kinase	1,67E-05
ouabain	chemical drug	1,67E-05
folic acid	chemical - endogenous mammalian	1,68E-05
trichloroethylene	chemical toxicant	1,68E-05
EBI3	cytokine	1,68E-05
di(2-ethylhexyl) phthalate	chemical toxicant	1,68E-05
metformin	chemical drug	1,70E-05
etanercept	biologic drug	1,74E-05
NSUN6	enzyme	1,74E-05
CAMK4	kinase	1,74E-05
JAG2	growth factor	1,74E-05
12-(3-adamantan-1-yl-ureido) dodecanoic acid	chemical reagent	1,74E-05
NANOG	transcription regulator	1,75E-05
miR-1-3p (and other miRNAs w/seed GGAAUGU)	mature microRNA	1,75E-05
MAFB	transcription regulator	1,88E-05
diphtheria toxin	chemical - endogenous non-mammalian	1,90E-05
ABCB4	transporter	1,94E-05
EIF4EBP1	translation regulator	1,94E-05
MAP2K5	kinase	1,94E-05
IFIH1	enzyme	1,94E-05
ERN1	kinase	1,95E-05
halofuginone	chemical drug	1,95E-05
Gsk3	group	2,01E-05
JAG1	growth factor	2,11E-05
THPO	cytokine	2,13E-05
CRBN	enzyme	2,15E-05
C5	cytokine	2,17E-05
benzyl isothiocyanate	chemical - endogenous non-mammalian	2,17E-05
Ro31-8220	chemical drug	2,17E-05
plicamycin	chemical drug	2,18E-05
IRF5	transcription regulator	2,23E-05
zidovudine	chemical drug	2,25E-05
Pkg	group	2,25E-05
4-O-carboxymethylascochlorin	chemical reagent	2,25E-05

CETP	enzyme	2,25E-05
evodiamine	chemical - endogenous non-mammalian	2,25E-05
ilomastat	chemical drug	2,25E-05
MAOA	enzyme	2,29E-05
bryostatin 1	chemical drug	2,29E-05
vismodegib	chemical drug	2,31E-05
mir-148	microRNA	2,31E-05
TAF4	transcription regulator	2,39E-05
MXD1	transcription regulator	2,41E-05
JUND	transcription regulator	2,41E-05
GSKJ4	chemical reagent	2,41E-05
BRD2	kinase	2,46E-05
CGA	other	2,48E-05
GLI2	transcription regulator	2,52E-05
paraquat	chemical toxicant	2,52E-05
RNF31	enzyme	2,55E-05
JAK1	kinase	2,55E-05
CRH	cytokine	2,55E-05
SIRT3	enzyme	2,62E-05
CIP2A	other	2,62E-05
TSH	complex	2,62E-05
RPTOR	other	2,63E-05
IL6R	transmembrane receptor	2,64E-05
cytochalasin B	chemical toxicant	2,80E-05
ZEB1	transcription regulator	2,80E-05
Igkv1-117	other	2,82E-05
acetic acid	chemical - endogenous mammalian	2,82E-05
tiron	chemical reagent	2,82E-05
MAP3K12	kinase	2,88E-05
EPOR	transmembrane receptor	2,88E-05
pevonedistat	chemical drug	2,88E-05
GABARAPL2	other	2,88E-05
hemozoin	chemical - endogenous non-mammalian	2,88E-05
erlotinib	chemical drug	2,89E-05
let-7a-5p (and other miRNAs w/seed GAGGUAG)	mature microRNA	2,95E-05
MED1	transcription regulator	2,97E-05
losartan potassium	chemical drug	2,97E-05
RNASEH2A	enzyme	3,05E-05
TSC22D3	transcription regulator	3,09E-05
1-palmitoyl-2-(5-oxovaleroyl)-sn-glycero-3-phosphorylcholine	chemical reagent	3,11E-05
IL36A	cytokine	3,11E-05
NPSR1	G-protein coupled receptor	3,13E-05
CHFR	enzyme	3,13E-05
ITGAX	transmembrane receptor	3,13E-05

adenosine	chemical - endogenous mammalian	3,21E-05
NOTCH3	transcription regulator	3,21E-05
STUB1	enzyme	3,21E-05
vitamin D	chemical drug	3,21E-05
L-triiodothyronine	chemical - endogenous mammalian	3,35E-05
pyrrolidine dithiocarbamate	chemical reagent	3,36E-05
IL36B	cytokine	3,37E-05
TINCR	other	3,37E-05
NTRK1	kinase	3,37E-05
Mt2	other	3,37E-05
SOCS2	other	3,37E-05
cannabidiol	chemical drug	3,37E-05
NUMB	other	3,47E-05
ACTN4	transcription regulator	3,47E-05
DPP-23	chemical reagent	3,47E-05
SOX4	transcription regulator	3,62E-05
PRKN	enzyme	3,66E-05
phorbol esters	chemical - other	3,66E-05
IL11	cytokine	3,68E-05
SMAD4	transcription regulator	3,73E-05
Ifnar	group	3,81E-05
miR-291a-3p (and other miRNAs w/seed AAGUGCU)	mature microRNA	3,81E-05
ICAM1	transmembrane receptor	3,86E-05
FCGR2A	transmembrane receptor	3,86E-05
F3	transmembrane receptor	3,87E-05
FOXO4	transcription regulator	3,87E-05
KIT	transmembrane receptor	3,87E-05
NCOR2	transcription regulator	3,95E-05
CIITA	transcription regulator	3,98E-05
ATF1	transcription regulator	3,98E-05
CCR5	G-protein coupled receptor	3,98E-05
chloropromazine	chemical drug	3,98E-05
WNT5A	cytokine	4,00E-05
GABARAPL1	other	4,03E-05
EIF2B5	translation regulator	4,03E-05
C1QBP	transcription regulator	4,03E-05
ferulic acid	chemical - endogenous non-mammalian	4,03E-05
bardoxolone methyl	chemical drug	4,03E-05
N(G)-monomethyl-D-arginine	chemical - endogenous mammalian	4,03E-05
NR4A1	ligand-dependent nuclear receptor	4,04E-05
TNFSF14	cytokine	4,18E-05
TRIB3	kinase	4,18E-05
MYL2	other	4,18E-05
andrographolide	chemical drug	4,18E-05

vinblastine	chemical drug	4,18E-05
PCM1	other	4,32E-05
RBX1	enzyme	4,32E-05
CKS1B	kinase	4,32E-05
4-coumaric acid	chemical - endogenous mammalian	4,32E-05
2-mercaptoethanol	chemical - endogenous mammalian	4,32E-05
NEIL2	enzyme	4,39E-05
miR-199a-5p (and other miRNAs w/seed CCAGUGU)	mature microRNA	4,39E-05
cephaloridine	chemical drug	4,43E-05
baicalin	chemical - endogenous non-mammalian	4,53E-05
ITGA5	transmembrane receptor	4,53E-05
TWIST2	transcription regulator	4,53E-05
miR-21-5p (and other miRNAs w/seed AGCUUAU)	mature microRNA	4,67E-05
ITGB3	transmembrane receptor	4,67E-05
platelet activating factor	chemical - endogenous mammalian	4,80E-05
VEGFB	growth factor	4,80E-05
CLU	other	4,80E-05
mir-24	microRNA	4,80E-05
amphotericin B	chemical drug	4,90E-05
nystatin	chemical drug	4,90E-05
TRRAP	transcription regulator	4,90E-05
dexmedetomidine	chemical drug	4,90E-05
geranylgeranylacetone	chemical drug	4,90E-05
CALC	group	4,90E-05
carfilzomib	biologic drug	4,91E-05
POLR2M	other	4,91E-05
E2F5	transcription regulator	4,91E-05
CCL3	cytokine	4,91E-05
kaempferol	chemical toxicant	4,91E-05
UCN-01	chemical drug	4,91E-05
levodopa	chemical - endogenous mammalian	5,00E-05
TGFB2	growth factor	5,09E-05
CD2	transmembrane receptor	5,14E-05
CSF1R	kinase	5,14E-05
D-galactosamine	chemical - endogenous mammalian	5,14E-05
VIP	other	5,16E-05
BCL2	transporter	5,16E-05
LEF1	transcription regulator	5,19E-05
COP1	enzyme	5,23E-05
cocaine	chemical drug	5,39E-05
AURKB	kinase	5,41E-05
anakinra	biologic drug	5,55E-05
GABARAP	transporter	5,55E-05
glutathione	chemical - endogenous mammalian	5,61E-05

TEAD1	transcription regulator	5,62E-05
LIN28A	other	5,71E-05
BAPTA-AM	chemical reagent	5,71E-05
HUWE1	transcription regulator	5,81E-05
ganetespib	chemical drug	5,81E-05
DAB2IP	other	5,81E-05
AFP	transporter	5,81E-05
norepinephrine	chemical - endogenous mammalian	5,89E-05
N-nitro-L-arginine methyl ester	chemical drug	5,95E-05
GNA12	enzyme	6,20E-05
SFTPA1	transporter	6,20E-05
IFNAR1	transmembrane receptor	6,28E-05
UQCC3	other	6,48E-05
DDX58	enzyme	6,48E-05
IFNL1	cytokine	6,60E-05
ITK	kinase	6,60E-05
SHC1	other	6,72E-05
GDNF	growth factor	6,72E-05
gemfibrozil	chemical drug	6,72E-05
tert-butyl-hydroquinone	chemical reagent	6,81E-05
CD9	other	7,01E-05
ADAMTS12	peptidase	7,01E-05
CXCR4	G-protein coupled receptor	7,11E-05
rotenone	chemical toxicant	7,11E-05
8-chlorophenylthio-adenosine 3',5'-cyclic monophosphate	chemical reagent	7,14E-05
TRPS1	transcription regulator	7,14E-05
MEN1	transcription regulator	7,14E-05
IRGM	enzyme	7,14E-05
H2AB3 (includes others)	other	7,14E-05
LRP1	transmembrane receptor	7,28E-05
CCL11	cytokine	7,28E-05
IFNA1/IFNA13	cytokine	7,28E-05
CAB39L	kinase	7,28E-05
daporinad	chemical drug	7,28E-05
RASSF5	other	7,28E-05
GJA1	transporter	7,28E-05
KRT17	other	7,28E-05
SN-38	chemical drug	7,28E-05
PTPN11	phosphatase	7,33E-05
grape seed extract	chemical drug	7,53E-05
ROR1	kinase	7,53E-05
SRSF1	other	7,53E-05
5-hydroxytryptamine	chemical - endogenous mammalian	7,56E-05
SBDS	other	7,72E-05

cinnamaldehyde	chemical toxicant	7,72E-05
ShK-223	chemical reagent	7,92E-05
Ngf	group	8,01E-05
ALKBH1	enzyme	8,15E-05
KLF17	transcription regulator	8,15E-05
H1-2	other	8,15E-05
MAPK10	kinase	8,15E-05
asciminib	chemical drug	8,15E-05
RHOB	enzyme	8,15E-05
L-685,458	chemical - protease inhibitor	8,15E-05
DT-061	chemical reagent	8,16E-05
ABT-737	chemical drug	8,16E-05
SMARCE1	transcription regulator	8,16E-05
PD 153035	chemical drug	8,16E-05
SMARCA5	transcription regulator	8,21E-05
lipid A	chemical toxicant	8,38E-05
estriol	chemical - endogenous mammalian	8,38E-05
conjugated linoleic acid	chemical drug	8,38E-05
SKIV2L	enzyme	8,38E-05
A2M	transporter	8,38E-05
WWTR1	transcription regulator	8,60E-05
N-Ac-Leu-Leu-norleucinal	chemical - protease inhibitor	8,64E-05
UCP1	transporter	8,69E-05
WNT1	cytokine	8,74E-05
advanced glycation end-products	chemical - endogenous mammalian	8,88E-05
3-methyladenine	chemical toxicant	8,89E-05
ZAP70	kinase	8,89E-05
TAC1	other	9,01E-05
lithium chloride	chemical drug	9,28E-05
IRF2BP1	transcription regulator	9,46E-05
ITCH	enzyme	9,46E-05
FGFR3	kinase	9,46E-05
MEOX2	transcription regulator	9,46E-05
TRAF6	enzyme	9,52E-05
PRKAA2	kinase	9,82E-05
NFAT (complex)	complex	9,82E-05
Mt1	other	9,82E-05
irinotecan	chemical drug	9,82E-05
C1QA	other	1,01E-04
CNOT7	transcription regulator	1,01E-04
BACH2	transcription regulator	1,03E-04
ERBB4	kinase	1,05E-04
SREBF2	transcription regulator	1,05E-04
methamphetamine	chemical drug	1,05E-04

STAT2	transcription regulator	1,08E-04
IRF7	transcription regulator	1,08E-04
GCS-100	chemical drug	1,08E-04
dinaciclib	chemical drug	1,08E-04
FANCA	other	1,08E-04
NFE2L3	transcription regulator	1,08E-04
MTBP	other	1,08E-04
FEM1A	transcription regulator	1,08E-04
EIF2AK1	kinase	1,08E-04
PPM1B	phosphatase	1,08E-04
DIABLO	other	1,08E-04
RHOC	enzyme	1,08E-04
COTI-2	chemical drug	1,08E-04
aplidine	biologic drug	1,08E-04
L-histidine	chemical - endogenous mammalian	1,09E-04
NELFB	other	1,09E-04
CCAT1	other	1,09E-04
ERRFI1	other	1,09E-04
NUMBL	other	1,09E-04
DGKA	kinase	1,09E-04
putrescine	chemical - endogenous mammalian	1,09E-04
cucurbitacin B	chemical - endogenous non-mammalian	1,09E-04
midostaurin	chemical drug	1,09E-04
ADAM10	peptidase	1,10E-04
molybdenum disulfide	chemical reagent	1,10E-04
fatty acid	chemical - endogenous mammalian	1,11E-04
ZBTB10	transcription regulator	1,17E-04
HMOX1	enzyme	1,18E-04
PF4	cytokine	1,19E-04
TNFRSF1A	transmembrane receptor	1,19E-04
TBK1	kinase	1,20E-04
phenethyl isothiocyanate	chemical drug	1,20E-04
IgG1	complex	1,20E-04
EIF4G2	translation regulator	1,20E-04
ADRB3	G-protein coupled receptor	1,20E-04
IND S1	chemical - kinase inhibitor	1,20E-04
RPS6KA5	kinase	1,20E-04
3-aminobenzamide	chemical toxicant	1,20E-04
edaravone	chemical drug	1,20E-04
W7	chemical reagent	1,20E-04
BAPTA	chemical reagent	1,20E-04
biochanin A	chemical toxicant	1,20E-04
propylthiouracil	chemical drug	1,21E-04
SAMSN1	other	1,22E-04

CGAS	enzyme	1,22E-04
TLR7/8	group	1,22E-04
tetraethylammonium	chemical drug	1,22E-04
NFATC3	transcription regulator	1,23E-04
doxifluridine	chemical drug	1,30E-04
ADP	chemical - endogenous mammalian	1,30E-04
Foxo	group	1,30E-04
Sod	group	1,30E-04
E2F7	transcription regulator	1,30E-04
FANCD2	other	1,30E-04
MMP8	peptidase	1,30E-04
C5	other	1,30E-04
actinonin	chemical reagent	1,30E-04
TFE3	transcription regulator	1,31E-04
tanshinone II	chemical - endogenous non-mammalian	1,32E-04
C3AR1	G-protein coupled receptor	1,32E-04
vincristine	chemical drug	1,32E-04
Il3	cytokine	1,33E-04
CISH	other	1,33E-04
bardoxolone	chemical drug	1,33E-04
carbamazepine	chemical drug	1,35E-04
calcipotriene	chemical drug	1,35E-04
PLA2R1	transmembrane receptor	1,35E-04
IND S7	chemical - kinase inhibitor	1,35E-04
ESRRA	transcription regulator	1,36E-04
Mapk	group	1,37E-04
FLT1	kinase	1,39E-04
CDX2	transcription regulator	1,42E-04
DOCK8	other	1,46E-04
PDGFB	growth factor	1,46E-04
FANCC	other	1,46E-04
ANXA7	ion channel	1,46E-04
N-acetylmuramyl-L-alanyl-D-isoglutamine	chemical - endogenous non-mammalian	1,46E-04
BUD23	enzyme	1,47E-04
TYMS	enzyme	1,47E-04
ARHGEF25	other	1,47E-04
floxuridine	chemical drug	1,47E-04
mir-25	microRNA	1,55E-04
ARRB1	transcription regulator	1,55E-04
DACH1	transcription regulator	1,55E-04
HNRNPU	transporter	1,55E-04
ATG7	enzyme	1,56E-04
UCHL1	peptidase	1,56E-04
AGER	transmembrane receptor	1,56E-04

TYROBP	transmembrane receptor	1,57E-04
ATP	chemical - endogenous mammalian	1,59E-04
HDAC2	transcription regulator	1,61E-04
TNFAIP3	enzyme	1,64E-04
DCN	other	1,64E-04
salicylic acid	chemical drug	1,64E-04
Fus	transcription regulator	1,65E-04
n-3 fatty acids	chemical drug	1,65E-04
NADPH oxidase	complex	1,72E-04
HMGXB4	other	1,72E-04
FAM3B	cytokine	1,72E-04
IL2RA	transmembrane receptor	1,72E-04
ELK4	transcription regulator	1,72E-04
TWNK	enzyme	1,72E-04
TSLP	cytokine	1,73E-04
mir-133	microRNA	1,73E-04
leucine	chemical - endogenous mammalian	1,74E-04
IL17RA	transmembrane receptor	1,74E-04
CDC42	enzyme	1,74E-04
green tea polyphenol	chemical drug	1,74E-04
SU6656	chemical toxicant	1,74E-04
CNR1	G-protein coupled receptor	1,76E-04
4-phenylbutyric acid	chemical - endogenous mammalian	1,77E-04
CDH11	other	1,83E-04
BCAP31	transporter	1,83E-04
PYCARD	transcription regulator	1,83E-04
NFKBIZ	transcription regulator	1,83E-04
MEL S3	chemical - kinase inhibitor	1,83E-04
nitroarginine	chemical reagent	1,83E-04
N-cor	group	1,83E-04
HMGB1	transcription regulator	1,87E-04
TLE3	other	1,87E-04
HDAC5	transcription regulator	1,87E-04
OSMR	transmembrane receptor	1,89E-04
KCNN4	ion channel	1,89E-04
CSF2RA	transmembrane receptor	1,89E-04
hydroquinone	chemical - endogenous mammalian	1,89E-04
bempedoic acid	chemical drug	1,89E-04
EP400	other	1,96E-04
carbamylcholine	chemical drug	1,96E-04
propofol	chemical drug	1,96E-04
S-nitrosoglutathione	chemical toxicant	1,96E-04
CSF2RB	transmembrane receptor	1,98E-04
Angiotensin II receptor type 1	group	1,98E-04

LYL1	transcription regulator	1,98E-04
CD70	cytokine	1,98E-04
indirubin	chemical drug	1,98E-04
nimodipine	chemical drug	1,98E-04
neuroprotectin D1	chemical - endogenous mammalian	1,98E-04
IGFBP2	other	1,99E-04
IL24	cytokine	2,03E-04
TFAP4	transcription regulator	2,03E-04
MTA1	transcription regulator	2,03E-04
TXN	enzyme	2,03E-04
PSEN2	peptidase	2,09E-04
ciprofibrate	chemical drug	2,14E-04
trinitrobenzenesulfonic acid	chemical reagent	2,15E-04
TGFB3	growth factor	2,15E-04
pioglitazone	chemical drug	2,24E-04
PPP2CA	phosphatase	2,25E-04
DDX5	enzyme	2,25E-04
emodin	chemical drug	2,25E-04
8-bromoguanosine 3',5'-cyclic monophosphate	chemical reagent	2,25E-04
miR-92a-3p (and other miRNAs w/seed AUUGCAC)	mature microRNA	2,25E-04
NCSTN	peptidase	2,25E-04
amiodarone	chemical drug	2,25E-04
phorbol 12,13-dibutyrate	chemical - endogenous non-mammalian	2,25E-04
Pde	group	2,28E-04
cytidylyl-3'-5'-guanosine	chemical reagent	2,28E-04
tryptase	group	2,28E-04
TRG	other	2,28E-04
HINFP	transcription regulator	2,28E-04
NELFA	other	2,28E-04
PTMA	other	2,28E-04
MC1R	G-protein coupled receptor	2,28E-04
YBX3	transcription regulator	2,28E-04
RTKN	other	2,28E-04
TKT	enzyme	2,28E-04
NELFE	other	2,28E-04
GNRH2	other	2,28E-04
asbestos	chemical toxicant	2,28E-04
PD 168393	chemical drug	2,28E-04
1,1-bis(3'-indolyl)-1-(4-trifluoromethyl-phenyl)methane	chemical reagent	2,28E-04
BRAF	kinase	2,37E-04
LIPE	enzyme	2,38E-04
dexamethasone phosphate	chemical drug	2,41E-04
ADCY	group	2,41E-04
Ikb	group	2,41E-04

UBA1	enzyme	2,41E-04
GDF15	growth factor	2,41E-04
IL37	cytokine	2,41E-04
THZ2	chemical drug	2,41E-04
MMP2	peptidase	2,41E-04
HFE	transmembrane receptor	2,42E-04
LLGL2	other	2,44E-04
CASP3	peptidase	2,44E-04
miR-7a-5p (and other miRNAs w/seed GGAAGAC)	mature microRNA	2,44E-04
arsenic	chemical toxicant	2,44E-04
IDH2	enzyme	2,46E-04
NFYC	transcription regulator	2,46E-04
MAP3K7	kinase	2,49E-04
NLRX1	other	2,49E-04
MYOC	other	2,50E-04
ADORA2A	G-protein coupled receptor	2,60E-04
sunitinib	chemical drug	2,67E-04
BCL2L1	other	2,67E-04
NRG2	growth factor	2,67E-04
GSTO1	enzyme	2,67E-04
CUX1	transcription regulator	2,67E-04
birabresib	chemical drug	2,67E-04
Ifn	group	2,72E-04
POU2F2	transcription regulator	2,82E-04
SERPINE1	other	2,83E-04
tyrphostin AG 1478	chemical drug	2,83E-04
3M-001	chemical drug	2,88E-04
XDH	enzyme	2,88E-04
FOSB	transcription regulator	2,88E-04
lysophosphatidylcholine	chemical - other	2,89E-04
PTTG1	transcription regulator	2,89E-04
galactosylceramide-alpha	chemical reagent	2,89E-04
PIM1	kinase	2,89E-04
brefeldin A	chemical - endogenous non-mammalian	2,89E-04
glucagon	biologic drug	2,89E-04
CpG ODN 1668	chemical reagent	2,89E-04
TCF12	transcription regulator	2,90E-04
mir-10	microRNA	2,91E-04
NCOA2	transcription regulator	2,92E-04
prostaglandin E1	chemical - endogenous mammalian	2,94E-04
OPA1	enzyme	2,94E-04
PREX1	other	2,94E-04
volasertib	chemical drug	2,94E-04
mir-203	microRNA	2,94E-04

SLC29A1	transporter	2,94E-04
N4BP1	enzyme	2,94E-04
PDCD4	other	2,94E-04
pomalidomide	chemical drug	2,94E-04
taxifolin	chemical - endogenous non-mammalian	2,94E-04
peroxynitrite	chemical toxicant	2,94E-04
BSG	transporter	3,04E-04
TEAD2	transcription regulator	3,04E-04
mir-30	microRNA	3,04E-04
USP22	peptidase	3,06E-04
IGHM	transmembrane receptor	3,06E-04
FGFR1	kinase	3,07E-04
phosphate	chemical - endogenous mammalian	3,07E-04
16,16-dimethylprostaglandin E2	chemical - endogenous mammalian	3,10E-04
Ck2 alpha	group	3,10E-04
Ginkgo biloba	chemical drug	3,10E-04
mocetinostat	chemical drug	3,10E-04
N(2)-(gamma-D-glutamyl)-meso-2,2'-diaminopimelic acid	chemical reagent	3,10E-04
Collagen Alpha1	group	3,10E-04
PELI1	enzyme	3,10E-04
NDUFA13	enzyme	3,10E-04
PEMT	enzyme	3,10E-04
MVP	other	3,10E-04
dehydroxymethylepoxyquinomicin	chemical reagent	3,10E-04
selenite	chemical toxicant	3,10E-04
growth factor	group	3,10E-04
RUNX3	transcription regulator	3,16E-04
KLF11	transcription regulator	3,16E-04
1,25-dihydroxyvitamin D	chemical drug	3,16E-04
PDK1	kinase	3,16E-04
caspase	group	3,21E-04
Ubiquitin	group	3,21E-04
SLC22A5	transporter	3,21E-04
AKT3	kinase	3,21E-04
USP18	peptidase	3,21E-04
VAV2	transcription regulator	3,21E-04
NPPA	other	3,21E-04
dithiothreitol	chemical reagent	3,21E-04
suramin	chemical drug	3,21E-04
linoleic acid	chemical - endogenous mammalian	3,30E-04
miR-145-5p (and other miRNAs w/seed UCCAGUU)	mature microRNA	3,30E-04
isotretinoin	biologic drug	3,30E-04
BCL2L11	other	3,31E-04
ITGA1	other	3,31E-04

USP19	peptidase	3,31E-04
IL10RA	transmembrane receptor	3,34E-04
IFN alpha/beta	group	3,35E-04
MACROH2A1	other	3,35E-04
PTHLH	other	3,35E-04
azoxymethane	chemical toxicant	3,35E-04
fingolimod	chemical drug	3,35E-04
ARNT2	transcription regulator	3,45E-04
mir-204	microRNA	3,46E-04
STIM1	ion channel	3,46E-04
TNFSF12	cytokine	3,48E-04
CYP19A1	enzyme	3,48E-04
NVP-TAE684	chemical drug	3,65E-04
eprenetapopt	chemical drug	3,65E-04
formaldehyde	chemical - endogenous mammalian	3,65E-04
GLI3	transcription regulator	3,66E-04
PLA2G10	enzyme	3,66E-04
SQSTM1	transcription regulator	3,69E-04
Collagen(s)	complex	3,78E-04
MAZ	transcription regulator	3,78E-04
TNFRSF9	transmembrane receptor	3,78E-04
MYB	transcription regulator	3,79E-04
corticosteroid	chemical drug	3,85E-04
SPDEF	transcription regulator	3,85E-04
cadmium	chemical toxicant	3,85E-04
PKM	kinase	3,86E-04
zinc	chemical drug	3,86E-04
NCOA1	transcription regulator	3,97E-04
IDH1	enzyme	4,12E-04
IL12B	cytokine	4,12E-04
NFATC1	transcription regulator	4,14E-04
8-hydroxyguanine	chemical - endogenous mammalian	4,15E-04
etretinate	chemical drug	4,15E-04
CERS5	transcription regulator	4,15E-04
RNF152	enzyme	4,15E-04
AQP11	transporter	4,15E-04
NEU4	enzyme	4,15E-04
obinutuzumab	biologic drug	4,15E-04
ENT	group	4,15E-04
MAT2A	enzyme	4,15E-04
F2RL3	G-protein coupled receptor	4,15E-04
RAB7A	enzyme	4,15E-04
POLRMT	enzyme	4,15E-04
SERPIND1	other	4,15E-04

MTUS1	other	4,15E-04
VTX-2337	chemical drug	4,15E-04
taselisib	chemical drug	4,15E-04
LGH447	chemical drug	4,15E-04
azetidine	chemical reagent	4,15E-04
6-cyano-7-nitroquinoxaline-2,3-dione	chemical reagent	4,15E-04
PK11007	chemical reagent	4,15E-04
UDP-D-glucose	chemical - endogenous mammalian	4,15E-04
rituximab	biologic drug	4,18E-04
CCNE1	transcription regulator	4,18E-04
tripterine	chemical - endogenous non-mammalian	4,18E-04
MLN8054	chemical drug	4,19E-04
2,5-dimethylcelecoxib	chemical reagent	4,19E-04
INTS11	enzyme	4,19E-04
USP12	peptidase	4,19E-04
ELL	transcription regulator	4,19E-04
miR-424-3p (miRNAs w/seed AAAACGU)	mature microRNA	4,19E-04
mir-920	microRNA	4,19E-04
MAPK15	kinase	4,19E-04
apilimod	chemical drug	4,19E-04
DLST	enzyme	4,19E-04
GFRA2	transmembrane receptor	4,19E-04
mir-297	microRNA	4,19E-04
proTAME	chemical reagent	4,19E-04
LFM-A13	chemical drug	4,19E-04
AZD4573	chemical drug	4,19E-04
2-amino-3-phosphonopropionic acid	chemical - endogenous mammalian	4,19E-04
PLCG1	enzyme	4,23E-04
DUSP5	phosphatase	4,23E-04
HRG	other	4,23E-04
HNRNPAB	enzyme	4,23E-04
WWTR1-CAMTA1	fusion gene/product	4,23E-04
NCD-38	chemical reagent	4,23E-04
(1S,2R)-NCL-1	chemical reagent	4,23E-04
bisphenol A	chemical - endogenous mammalian	4,24E-04
TEAD	group	4,28E-04
CLEC14A	other	4,28E-04
TRPM8	ion channel	4,28E-04
ITGA4	transmembrane receptor	4,28E-04
mir-506	microRNA	4,28E-04
NCK1	kinase	4,28E-04
alpha-santalol	chemical reagent	4,28E-04
ATF5	transcription regulator	4,28E-04
S100P	other	4,28E-04

cyclic guanosine monophosphate-adenosine monophosphate	chemical - endogenous non-mammalian	4,28E-04
2-hydroxy-1-naphthylaldehyde isonicotinoyl hydrazone	chemical reagent	4,28E-04
gossypol	chemical drug	4,28E-04
3,3'-diindolylmethane	chemical drug	4,37E-04
mir-17	microRNA	4,44E-04
CCN5	growth factor	4,45E-04
artesunic acid	chemical drug	4,47E-04
ZNF710	transcription regulator	4,47E-04
STK4	kinase	4,47E-04
PLK2	kinase	4,47E-04
KN-62	chemical drug	4,47E-04
aristolochic acid I	chemical toxicant	4,47E-04
tetrandrine	chemical drug	4,47E-04
SGPP2	phosphatase	4,47E-04
POR	enzyme	4,54E-04
SKP2	other	4,58E-04
TGS1	enzyme	4,58E-04
NGFR	transmembrane receptor	4,58E-04
RAD21	transcription regulator	4,58E-04
thalidomide	chemical drug	4,58E-04
S100A4	other	4,63E-04
hemin	chemical - endogenous mammalian	4,71E-04
1'-acetoxychavicol acetate	chemical reagent	4,84E-04
CLCN5	ion channel	4,84E-04
PACS1	other	4,84E-04
AIMP1	cytokine	4,84E-04
PNN	other	4,84E-04
N'-((4-oxo-4H-chromen-3-yl)methylene)nicotinohydrazide	chemical reagent	4,84E-04
CD209	other	4,84E-04
PIKFYVE	kinase	4,84E-04
OGG1	enzyme	4,84E-04
OCLN	other	4,84E-04
ST6GAL1	enzyme	4,84E-04
GTF2I	transcription regulator	4,84E-04
RFX5	transcription regulator	4,84E-04
DEK	transcription regulator	4,84E-04
lauric acid	chemical - endogenous mammalian	4,84E-04
prostaglandin D2	chemical - endogenous mammalian	4,88E-04
Hbb-b1	transporter	4,94E-04
Bay 11-7082	chemical - kinase inhibitor	4,94E-04
aldosterone	chemical - endogenous mammalian	4,96E-04
KAT5	transcription regulator	4,99E-04
MYCL	transcription regulator	5,10E-04
MAP2K6	kinase	5,17E-04

CDKN1B	kinase	5,31E-04
NFKBIB	transcription regulator	5,33E-04
MMP1	peptidase	5,33E-04
ANXA1	enzyme	5,33E-04
2,3-bis(4-hydroxyphenyl)-propionitrile	chemical reagent	5,33E-04
IRF2	transcription regulator	5,37E-04
SSRP1	transcription regulator	5,37E-04
clodronic acid	chemical drug	5,37E-04
diallyl disulfide	chemical - endogenous non-mammalian	5,37E-04
CCN2	growth factor	5,60E-04
BMP10	growth factor	5,61E-04
nocodazole	chemical reagent	5,61E-04
SERPINA1	other	5,64E-04
4-hydroxynonenal	chemical toxicant	5,64E-04
MK2206	chemical drug	5,70E-04
fluvastatin	chemical drug	5,70E-04
RNY3	other	5,94E-04
JUN/JUNB/JUND	group	5,94E-04
DCAF1	kinase	5,94E-04
CTSS	peptidase	5,94E-04
PLK4	kinase	5,94E-04
geranylgeranyl pyrophosphate	chemical - endogenous mammalian	5,94E-04
11,12-epoxyeicosatrienoic acid	chemical - endogenous mammalian	5,94E-04
Fibrinogen	complex	5,94E-04
Iga	complex	5,94E-04
CpG ODN 2395	chemical reagent	5,94E-04
NLRC5	transcription regulator	5,94E-04
Usp17la (includes others)	peptidase	5,94E-04
ST8SIA1	enzyme	5,94E-04
butaprost	chemical drug	5,94E-04
SN-011	chemical reagent	5,95E-04
HDL	complex	6,36E-04
FGF10	growth factor	6,36E-04
iron	chemical - endogenous mammalian	6,36E-04
FGF8	growth factor	6,37E-04
THRB	ligand-dependent nuclear receptor	6,58E-04
BMP6	growth factor	6,76E-04
ascorbic acid	chemical - endogenous mammalian	6,76E-04
RETN	other	6,76E-04
carbon monoxide	chemical - endogenous mammalian	6,76E-04
PROC	peptidase	6,83E-04
FYN	kinase	6,83E-04
TOB1	transcription regulator	6,83E-04
nifedipine	chemical drug	6,86E-04

RIPK2	kinase	6,91E-04
TCOF1	transporter	6,91E-04
SASH1	other	6,91E-04
TRIM24	transcription regulator	7,03E-04
CpG ODN 2006	chemical reagent	7,03E-04
HSPA9	other	7,04E-04
IL27RA	transmembrane receptor	7,04E-04
RORA	ligand-dependent nuclear receptor	7,13E-04
sphingosine-1-phosphate	chemical - endogenous mammalian	7,17E-04
NCOA3	transcription regulator	7,21E-04
RARB	ligand-dependent nuclear receptor	7,26E-04
IL1RL2	transmembrane receptor	7,26E-04
CSK	kinase	7,26E-04
VIPR1	G-protein coupled receptor	7,26E-04
MEL T1	chemical - kinase inhibitor	7,26E-04
MS4A1	other	7,26E-04
cyclopiazonic acid	chemical - endogenous non-mammalian	7,26E-04
hydrogen sulfide	chemical - endogenous mammalian	7,26E-04
N1,N11-diethylnorspermine	chemical drug	7,26E-04
MIF	cytokine	7,26E-04
8,9-epoxyeicosatrienoic acid	chemical - endogenous mammalian	7,37E-04
EPHB1	kinase	7,37E-04
Fcor	enzyme	7,37E-04
mir-224	microRNA	7,37E-04
APOH	transporter	7,37E-04
LGALS7/LGALS7B	other	7,37E-04
NPAT	transcription regulator	7,37E-04
LRPPRC	other	7,37E-04
2-[[9-isopropyl-6-[[4-(2-pyridyl)phenyl]methylamino]purin-2-yl]amino]butan-1-ol	chemical drug	7,37E-04
orlistat	chemical drug	7,37E-04
2,2-bis(4-hydroxyphenyl)-1,1,1-trichloroethane	chemical - endogenous mammalian	7,37E-04
FSHR	G-protein coupled receptor	7,55E-04
carbonyl cyanide m-chlorophenyl hydrazone	chemical toxicant	7,55E-04
2-(4-acetoxyphenyl)-2-chloro-N-methylethylamine	chemical reagent	7,77E-04
dibutyl phthalate	chemical toxicant	7,77E-04
miR-203a-3p (and other miRNAs w/seed UGAAAUG)	mature microRNA	7,77E-04
TRADD	other	7,77E-04
PRKAR2B	kinase	7,77E-04
CCR1	G-protein coupled receptor	7,77E-04
SL 327	chemical - protease inhibitor	7,77E-04
1-methyl-4-phenylpyridinium	chemical toxicant	7,77E-04
MALP-2s	chemical reagent	7,77E-04
hydrochloric acid	chemical - endogenous mammalian	7,77E-04
phorbol 12,13-didecanoate	chemical toxicant	7,77E-04

YBX1	transcription regulator	7,86E-04
CD247	transmembrane receptor	7,92E-04
ATP5IF1	other	7,92E-04
miR-9-5p (and other miRNAs w/seed CUUUGGU)	mature microRNA	8,00E-04
NR5A2	ligand-dependent nuclear receptor	8,04E-04
bezafibrate	chemical drug	8,14E-04
IL17R	complex	8,15E-04
MAT1A	enzyme	8,15E-04
LCP2	other	8,15E-04
GADD45A	other	8,15E-04
NOX1	enzyme	8,15E-04
lapatinib	chemical drug	8,15E-04
wogonin	chemical - endogenous non-mammalian	8,15E-04
IL23	complex	8,22E-04
SP4	transcription regulator	8,22E-04
INHA	growth factor	8,53E-04
RORC	ligand-dependent nuclear receptor	8,54E-04
PRKCZ	kinase	8,57E-04
SOST	other	8,57E-04
eplerenone	chemical drug	8,57E-04
Hsp90	group	8,62E-04
CpG ODN 1826	chemical reagent	8,62E-04
CYLD	transcription regulator	8,63E-04
AGTR1	G-protein coupled receptor	8,63E-04
STAG2	other	8,63E-04
SAHM1	chemical reagent	8,63E-04
ribavirin	chemical drug	8,63E-04
S-adenosylmethionine	chemical - endogenous mammalian	8,63E-04
alitretinoin	chemical drug	8,94E-04
(+)-MK-801	chemical drug	9,00E-04
flavokawain B	chemical - endogenous non-mammalian	9,12E-04
nicotinic acetylcholine receptor	complex	9,12E-04
Sphk	group	9,12E-04
AKIRIN2	other	9,12E-04
MT-TE	other	9,12E-04
Org 48762-0	chemical drug	9,12E-04
ASCL2	transcription regulator	9,12E-04
AZD4547	chemical drug	9,12E-04
PDE6B	enzyme	9,12E-04
HMGCR	enzyme	9,12E-04
CCNA2	other	9,12E-04
miR-511-5p (miRNAs w/seed UGUCUUU)	mature microRNA	9,12E-04
PDE3A	enzyme	9,12E-04
MYCBP	transcription regulator	9,12E-04

CTSE	peptidase	9,12E-04
TNFRSF14	transmembrane receptor	9,12E-04
FUBP1	transcription regulator	9,12E-04
CD226	other	9,12E-04
15-E2-isoketal modified phosphatidylethanolamine	chemical reagent	9,12E-04
PRN694	chemical drug	9,12E-04
Immunoglobulin Lambda Light Chain	group	9,12E-04
dacarbazine	chemical drug	9,12E-04
gliotoxin	chemical toxicant	9,12E-04
manidipine	chemical drug	9,12E-04
sanguinarine	chemical - endogenous non-mammalian	9,12E-04
Muscarinic cholinergic receptor	group	9,12E-04
Calcineurin protein(s)	complex	9,22E-04
KDM8	enzyme	9,22E-04
LCK	kinase	9,22E-04
TRAF3IP2	enzyme	9,22E-04
IL25	cytokine	9,23E-04
ITGB1	transmembrane receptor	9,69E-04
RNASEH2B	other	9,76E-04
cholecalciferol	chemical - endogenous mammalian	9,81E-04
GRP	growth factor	1,00E-03
LUCAT1	other	1,00E-03
PLD1	enzyme	1,00E-03
HOXA7	transcription regulator	1,00E-03
methylnitronitrosoguanidine	chemical toxicant	1,00E-03
dicarbethoxydihydrocollidine	chemical toxicant	1,00E-03
epoxomicin	chemical - protease inhibitor	1,00E-03
deoxynivalenol	chemical toxicant	1,00E-03
PKD1	ion channel	1,02E-03
chloroquine	chemical drug	1,03E-03
IL-1R	group	1,05E-03
LRP6	transmembrane receptor	1,05E-03
CD14	transmembrane receptor	1,05E-03
SERPINF1	other	1,05E-03
JAK	group	1,05E-03
SPRY2	other	1,05E-03
SRSF2	transcription regulator	1,05E-03
14,15-epoxyeicosatrienoic acid	chemical - endogenous mammalian	1,05E-03
lonafarnib	chemical drug	1,05E-03
Integrin	complex	1,05E-03
PPP1R1B	phosphatase	1,05E-03
SERPINC1	enzyme	1,05E-03
CSDE1	enzyme	1,05E-03
CXCL2	cytokine	1,05E-03

ABCC8	transporter	1,05E-03
S1PR3	G-protein coupled receptor	1,05E-03
phenyl-N-tert-butylnitrone	chemical reagent	1,05E-03
amlodipine	chemical drug	1,05E-03
oxysterol	chemical - endogenous mammalian	1,05E-03
asoprisnil	chemical drug	1,07E-03
SYK/ZAP	group	1,07E-03
PHF6	transcription regulator	1,07E-03
MIR124	group	1,07E-03
ACVRL1	kinase	1,07E-03
ACKR3	G-protein coupled receptor	1,07E-03
mercuric chloride	chemical toxicant	1,07E-03
PML-RARA	fusion gene/product	1,07E-03
MBD2	transcription regulator	1,07E-03
trastuzumab	biologic drug	1,10E-03
IL31	other	1,10E-03
CTR9	other	1,10E-03
CCK	other	1,10E-03
RARRES2	transmembrane receptor	1,10E-03
desipramine	chemical drug	1,10E-03
1-chloro-2,4-dinitrobenzene	chemical drug	1,10E-03
verteporfin	chemical drug	1,10E-03
lipoarabinomannan	chemical - endogenous non-mammalian	1,10E-03
tauroursodeoxycholic acid	chemical - endogenous mammalian	1,10E-03
CFTR	ion channel	1,10E-03
CYP1A1	enzyme	1,16E-03
GNAS	enzyme	1,17E-03
NQO1	enzyme	1,17E-03
icilin	chemical reagent	1,19E-03
ALKBH7	other	1,19E-03
PBRM1	other	1,19E-03
4-methylene-2-octyl-5-oxotetrahydrofuran-3-carboxylic acid	chemical reagent	1,19E-03
SH3KBP1	other	1,19E-03
PIAS2	transcription regulator	1,19E-03
DRD1	G-protein coupled receptor	1,19E-03
ELN	other	1,19E-03
CACNA1A	ion channel	1,19E-03
azetidyl-2-carboxylic acid	chemical reagent	1,19E-03
quinacrine	chemical drug	1,19E-03
corticosterone	chemical - endogenous mammalian	1,20E-03
CLPP	peptidase	1,22E-03
NOD2	other	1,22E-03
ETS2	transcription regulator	1,22E-03
IKBKE	kinase	1,25E-03

SUMO2	enzyme	1,27E-03
TRIM37	enzyme	1,28E-03
CLEC7A	transmembrane receptor	1,28E-03
POLG	enzyme	1,28E-03
gamma-secretase inhibitor compound E	chemical reagent	1,28E-03
SENP1	peptidase	1,28E-03
2'3'-cyclic guanosine monophosphate-adenosine monophosphate	chemical - endogenous mammalian	1,28E-03
fucoidin	chemical reagent	1,28E-03
POMC	other	1,30E-03
IKZF2	transcription regulator	1,32E-03
PRKCQ	kinase	1,32E-03
ELF4	transcription regulator	1,32E-03
histone deacetylase inhibitor	chemical drug	1,32E-03
PD173074	chemical reagent	1,32E-03
TEAD3	transcription regulator	1,39E-03
HIPK2	kinase	1,39E-03
MEF2D	transcription regulator	1,39E-03
Hif1	complex	1,39E-03
PPP3CA	phosphatase	1,40E-03
elaidic acid	chemical - endogenous mammalian	1,40E-03
FLI1	transcription regulator	1,43E-03
GPS2	transcription regulator	1,43E-03
IL6ST	transmembrane receptor	1,43E-03
PARP1	enzyme	1,44E-03
pregna-4,17-diene-3,16-dione	chemical - endogenous non-mammalian	1,45E-03
Cbp/p300	group	1,45E-03
TOX	transcription regulator	1,45E-03
HOXA13	transcription regulator	1,45E-03
CDH2	other	1,45E-03
resolvin D2	chemical - endogenous mammalian	1,45E-03
MAP3K3	kinase	1,45E-03
SAFB2	other	1,45E-03
TGFB1I1	transcription regulator	1,45E-03
LCAT	enzyme	1,45E-03
MMP11	peptidase	1,45E-03
MLN120B	chemical drug	1,45E-03
P2RX7	ion channel	1,45E-03
LILRB1	transmembrane receptor	1,45E-03
THZ1	chemical drug	1,45E-03
bisindolylmaleimide iv	chemical drug	1,45E-03
nickel	chemical toxicant	1,45E-03
GW9662	chemical reagent	1,45E-03
AIRE	transcription regulator	1,48E-03
glycine	chemical - endogenous mammalian	1,48E-03

BMS-345541	chemical drug	1,48E-03
FZD8	G-protein coupled receptor	1,48E-03
tofacitinib	chemical drug	1,48E-03
elastase	group	1,48E-03
Endothelin	group	1,48E-03
TAF1	transcription regulator	1,48E-03
mir-451	microRNA	1,48E-03
BARX2	transcription regulator	1,48E-03
ITGAL	transmembrane receptor	1,48E-03
RPS6KA3	kinase	1,48E-03
CXCL3	cytokine	1,48E-03
MAML1	transcription regulator	1,48E-03
BHLHA15	transcription regulator	1,48E-03
GAB1	other	1,48E-03
NCR2	transmembrane receptor	1,48E-03
magnolol	chemical - endogenous non-mammalian	1,48E-03
thymoquinone	chemical drug	1,52E-03
RBM5	other	1,52E-03
ATP2B2	transporter	1,52E-03
DPP4	peptidase	1,52E-03
sildenafil	chemical drug	1,52E-03
2-amino-1-methyl-6-phenylimidazo-4-5-b-pyridine	chemical toxicant	1,52E-03
FOXA1	transcription regulator	1,54E-03
PD 180970	chemical drug	1,58E-03
colfosceril palmitate	chemical - endogenous mammalian	1,58E-03
adalimumab	biologic drug	1,58E-03
1-methyl-2-pyrrolidinone	chemical drug	1,58E-03
CINP	other	1,58E-03
CHIA	enzyme	1,58E-03
XRN2	enzyme	1,58E-03
P-TEFb	complex	1,58E-03
sepantronium	chemical drug	1,58E-03
danusertib	chemical drug	1,58E-03
FLII	other	1,58E-03
PF-4691502	chemical drug	1,58E-03
G0S2	other	1,58E-03
miR-181a-2-3p (and other miRNAs w/seed CCACUGA)	mature microRNA	1,58E-03
miR-331-3p (miRNAs w/seed CCCCUGG)	mature microRNA	1,58E-03
mir-105	microRNA	1,58E-03
TIAM1	other	1,58E-03
GCLC	enzyme	1,58E-03
CD151	other	1,58E-03
AHNAK	other	1,58E-03
BAG2	other	1,58E-03

ATP2A1	transporter	1,58E-03
PAPOLA	enzyme	1,58E-03
LPAR3	G-protein coupled receptor	1,58E-03
ACP1	phosphatase	1,58E-03
Mt3	other	1,58E-03
TOP2A	enzyme	1,58E-03
ZC3H13	other	1,58E-03
MAD1L1	other	1,58E-03
CKS2	kinase	1,58E-03
VR23	chemical reagent	1,58E-03
1-(2-hydroxy-5-methylphenyl)-3-phenyl-1,3-propanedione	chemical reagent	1,58E-03
abrocitinib	chemical drug	1,58E-03
phenylacetate	chemical - endogenous mammalian	1,58E-03
verlukast	chemical drug	1,58E-03
atipamezole	chemical drug	1,58E-03
hoechst 33342	chemical reagent	1,58E-03
2-mercaptoethylguanidine	chemical reagent	1,58E-03
chlorophyllin	chemical toxicant	1,58E-03
MM218	chemical reagent	1,58E-03
rubitecan	chemical drug	1,58E-03
ursolic acid	chemical drug	1,58E-03
VAV1	transcription regulator	1,61E-03
CTSB	peptidase	1,61E-03
VTN	other	1,61E-03
urethane	chemical toxicant	1,61E-03
crocidolite asbestos	chemical toxicant	1,61E-03
proteasome inhibitor PSI	chemical - protease inhibitor	1,61E-03
glycyrrhizic acid	chemical drug	1,61E-03
bufalin	chemical reagent	1,61E-03
rasagiline	chemical drug	1,62E-03
GNRH	group	1,62E-03
SUMO3	other	1,62E-03
TLR5	transmembrane receptor	1,62E-03
SIX1	transcription regulator	1,63E-03
Collagen type I (complex)	complex	1,64E-03
FKBP10	enzyme	1,66E-03
morphine	chemical drug	1,67E-03
ATG5	other	1,69E-03
lithium	chemical drug	1,69E-03
saikosaponin A	chemical reagent	1,72E-03
CENPN	other	1,72E-03
motexafin gadolinium	chemical drug	1,72E-03
2,5-bis(5-hydroxymethyl-2-thienyl)furan	chemical reagent	1,72E-03
Cyp4a14	enzyme	1,72E-03

BI 2536	chemical drug	1,72E-03
Cyclin A	group	1,72E-03
R 406	chemical drug	1,72E-03
CYTOR	other	1,72E-03
PADI4	enzyme	1,72E-03
GSTK1	enzyme	1,72E-03
CACNA1C	ion channel	1,72E-03
TRPC4AP	transporter	1,72E-03
PHF10	other	1,72E-03
ADGRF5	G-protein coupled receptor	1,72E-03
NELFCD	other	1,72E-03
LTBP4	growth factor	1,72E-03
BANP	other	1,72E-03
miR-2392 (miRNAs w/seed AGGAUGG)	mature microRNA	1,72E-03
mir-2392	microRNA	1,72E-03
SMARCA1	transcription regulator	1,72E-03
EGR4	transcription regulator	1,72E-03
FALEC	other	1,72E-03
CLTC	other	1,72E-03
RASSF10	other	1,72E-03
BLACAT1	other	1,72E-03
teasaponin	chemical - endogenous non-mammalian	1,72E-03
perifosine	chemical drug	1,72E-03
R59949	chemical drug	1,72E-03
SCH79797	chemical reagent	1,72E-03
mechlorethamine	chemical drug	1,72E-03
domoic acid	chemical toxicant	1,72E-03
AGI-1067	chemical drug	1,72E-03
epoxyeicosatrienoic acid analog B	chemical reagent	1,72E-03
epoxyeicosatrienoic acid analog A	chemical reagent	1,72E-03
buserelin	biologic drug	1,72E-03
dihematoporphyrin ether	chemical drug	1,72E-03
theaflavin	chemical - endogenous non-mammalian	1,72E-03
4-(1-D-ribitylamino)-5-amino-2,6-dihydroxypyrimidine	chemical - endogenous non-mammalian	1,72E-03
ROR2	kinase	1,80E-03
TRAF3	enzyme	1,80E-03
cucurbitacin I	chemical reagent	1,81E-03
FOXD2-AS1	other	1,81E-03
PRR11	other	1,81E-03
IgG2a	complex	1,81E-03
Ppp2c	group	1,81E-03
SRT1720	chemical drug	1,81E-03
L1CAM	other	1,81E-03
FZD7	G-protein coupled receptor	1,81E-03

PGLYRP2	transmembrane receptor	1,81E-03
RPS6KA4	kinase	1,81E-03
B4GALNT1	enzyme	1,81E-03
CD163	transmembrane receptor	1,81E-03
VLDLR	transporter	1,81E-03
LETMD1	other	1,81E-03
PPP5C	phosphatase	1,81E-03
CCL3L3	cytokine	1,81E-03
HPSE	enzyme	1,81E-03
sevoflurane	chemical drug	1,81E-03
[D-Ala2,N-Me-Phe4,Gly5-ol]-Enkephalin	chemical reagent	1,81E-03
cyclomaltodextrin	chemical drug	1,81E-03
IL12A	cytokine	1,82E-03
ADORA2B	G-protein coupled receptor	1,82E-03
RUVBL1	transcription regulator	1,82E-03
Hbb-b2	other	1,82E-03
IFNLR1	transmembrane receptor	1,82E-03
Pam3-Cys	chemical toxicant	1,82E-03
4-methylnitrosoamino-1-(3-pyridinyl)-1-butanone	chemical toxicant	1,82E-03
IFNGR1	transmembrane receptor	1,82E-03
TLR7	transmembrane receptor	1,83E-03
3-deazaneplanocin	chemical drug	1,88E-03
selumetinib	chemical drug	1,88E-03
2-aminopurine	chemical reagent	1,88E-03
BIRC3	enzyme	1,88E-03
Smad	complex	1,88E-03
IREB2	translation regulator	1,88E-03
ketamine	chemical drug	1,88E-03
aphidicolin	chemical toxicant	1,88E-03
ammonium chloride	chemical drug	1,88E-03
tosylphenylalanyl chloromethyl ketone	chemical - protease inhibitor	1,88E-03
ciglitazone	chemical drug	1,89E-03
ROCK	group	1,91E-03
3-nitropropionic acid	chemical toxicant	1,91E-03
salirasib	chemical drug	1,91E-03
NONO	transcription regulator	1,94E-03
TNFSF13	cytokine	1,96E-03
ALOX15	enzyme	1,96E-03
IGFBP3	other	1,96E-03
SERPINE2	other	1,96E-03
BTG2	transcription regulator	1,96E-03
CAMP	other	2,00E-03
TRPM2	ion channel	2,01E-03
ITGAV	transmembrane receptor	2,01E-03

HEIH	other	2,01E-03
LTB4R	G-protein coupled receptor	2,01E-03
EIF4EBP2	translation regulator	2,01E-03
PARK7	enzyme	2,01E-03
pitavastatin	chemical drug	2,01E-03
linsidomine	chemical drug	2,01E-03
ceramide	chemical - endogenous mammalian	2,01E-03
Ctbp	group	2,02E-03
RHOJ	enzyme	2,02E-03
CBX7	other	2,02E-03
CREBZF	transcription regulator	2,02E-03
S100B	other	2,02E-03
CDK5R1	kinase	2,02E-03
ITGA6	transmembrane receptor	2,02E-03
MAPKAPK3	kinase	2,02E-03
thiazolidinedione	chemical drug	2,02E-03
leuprolide	biologic drug	2,02E-03
vanadate	chemical - other	2,02E-03
miR-34a-5p (and other miRNAs w/seed GGCAGUG)	mature microRNA	2,13E-03
NRF1	transcription regulator	2,15E-03
RHO	G-protein coupled receptor	2,15E-03
IKZF3	transcription regulator	2,15E-03
HDAC6	transcription regulator	2,16E-03
isoprenaline	chemical drug	2,16E-03
zoledronic acid	chemical drug	2,16E-03
LIN28B	other	2,22E-03
PIK3CG	kinase	2,22E-03
LPL	enzyme	2,23E-03
EIF2S1	translation regulator	2,23E-03
EGR2	transcription regulator	2,25E-03
heparin	chemical - endogenous mammalian	2,30E-03
SAA	group	2,37E-03
CERK	kinase	2,37E-03
ATP7B	transporter	2,37E-03
FCGR2B	transmembrane receptor	2,37E-03
TNFSF15	cytokine	2,37E-03
PDGF-AA	complex	2,41E-03
shikonin	chemical drug	2,41E-03
RNF20	enzyme	2,41E-03
TICAM2	other	2,41E-03
BTRC	enzyme	2,41E-03
ERF	transcription regulator	2,41E-03
AZGP1	transporter	2,41E-03
H-[1,2,4]oxadiazolo[4,3-alpha]quinoxalin-1-one	chemical reagent	2,41E-03

DKK1	growth factor	2,45E-03
3M-011	chemical reagent	2,48E-03
eltanexor	chemical drug	2,48E-03
rosuvastatin	chemical drug	2,48E-03
SFLLRN (PAR1-activator)	chemical reagent	2,48E-03
DLL4	other	2,55E-03
PPRC1	transcription regulator	2,55E-03
dimethylnitrosamine	chemical toxicant	2,55E-03
FGF21	growth factor	2,55E-03
miR-204-5p (and other miRNAs w/seed UCCCUUU)	mature microRNA	2,55E-03
CEBPE	transcription regulator	2,59E-03
CBL	transcription regulator	2,59E-03
RETNLB	other	2,60E-03
U46619	chemical reagent	2,64E-03
triciribine	chemical drug	2,64E-03
xanthine	chemical - endogenous mammalian	2,64E-03
glycochenodeoxycholate	chemical - endogenous mammalian	2,64E-03
GON4L	transcription regulator	2,64E-03
Rac	group	2,64E-03
SNHG11	other	2,64E-03
TFAP2E	transcription regulator	2,64E-03
LILRA2	other	2,64E-03
SAMHD1	enzyme	2,64E-03
miR-150-5p (and other miRNAs w/seed CUCCCAA)	mature microRNA	2,64E-03
mir-375	microRNA	2,64E-03
PLCE1	enzyme	2,64E-03
WWP2	enzyme	2,64E-03
NPFF	other	2,64E-03
NEU1	enzyme	2,64E-03
ferric ammonium citrate	chemical drug	2,64E-03
salmeterol	chemical drug	2,64E-03
pentobarbital	chemical drug	2,64E-03
zeranol	chemical - endogenous mammalian	2,64E-03
ethylenediaminetetraacetic acid	chemical drug	2,64E-03
anti-benzo(a)pyrene-diol-epoxide	chemical toxicant	2,64E-03
noscapine	chemical drug	2,64E-03
D-tubocurarine	chemical drug	2,64E-03
Cd2+	chemical toxicant	2,64E-03
ZFHX3	transcription regulator	2,69E-03
phenylephrine	chemical drug	2,69E-03
puromycin	chemical - endogenous non-mammalian	2,71E-03
8-pCPT-2-O-Me-cAMP	chemical reagent	2,71E-03
MSR1	transmembrane receptor	2,71E-03
ZBP1	other	2,71E-03

RMRP	other	2,71E-03
PLA2G4A	enzyme	2,71E-03
DHX9	enzyme	2,71E-03
GFI1B	transcription regulator	2,71E-03
prazosin	chemical drug	2,71E-03
sulfasalazine	chemical drug	2,71E-03
zymosan A	chemical - endogenous non-mammalian	2,71E-03
elovanoid N32	chemical - endogenous mammalian	2,71E-03
RXRB	ligand-dependent nuclear receptor	2,78E-03
TARDBP	transcription regulator	2,78E-03
SOX1	transcription regulator	2,78E-03
ZBTB7B	transcription regulator	2,78E-03
PAX7	transcription regulator	2,78E-03
GDF11	growth factor	2,78E-03
H-7	chemical - kinase inhibitor	2,83E-03
geneticin	chemical toxicant	2,91E-03
RUNX1T1	transcription regulator	2,91E-03
Ck2	complex	2,91E-03
SLC16A3	transporter	2,91E-03
Hoxa11os	other	2,91E-03
MEMO1	other	2,91E-03
IL26	cytokine	2,91E-03
nicotiflorin	chemical reagent	2,91E-03
pictilisib	chemical drug	2,91E-03
bicyclol	chemical drug	2,91E-03
PRTN3	peptidase	2,91E-03
Ccl6	cytokine	2,91E-03
RFXAP	transcription regulator	2,91E-03
SHOX	transcription regulator	2,91E-03
PTGES3	enzyme	2,91E-03
RPL11	other	2,91E-03
CD160	transmembrane receptor	2,91E-03
MCAM	other	2,91E-03
niflumic acid	chemical drug	2,91E-03
prodigiosin	chemical toxicant	2,91E-03
SR1078	chemical reagent	2,91E-03
3-beta,17-beta-androstanediol	chemical - endogenous mammalian	2,91E-03
IL2RG	transmembrane receptor	2,92E-03
SIM1	transcription regulator	2,96E-03
LGR4	transmembrane receptor	2,99E-03
Srgn	other	2,99E-03
TXNIP	other	3,00E-03
luteolin	chemical drug	3,00E-03
miR-29b-3p (and other miRNAs w/seed AGCACCA)	mature microRNA	3,02E-03

SPIB	transcription regulator	3,02E-03
Rar	group	3,04E-03
ZBED6	transcription regulator	3,04E-03
GAB2	other	3,04E-03
IGF2BP1	translation regulator	3,04E-03
LOC105372576	other	3,04E-03
CD300LF	other	3,05E-03
IL17C	cytokine	3,05E-03
CHI3L1	enzyme	3,05E-03
PROCR	other	3,05E-03
DTX1	transcription regulator	3,05E-03
NFIX	transcription regulator	3,05E-03
calyculin A	chemical toxicant	3,05E-03
rifaximin	chemical drug	3,05E-03
TBP	transcription regulator	3,14E-03
Nr1h	group	3,25E-03
GMNN	transcription regulator	3,28E-03
zymosan	chemical - endogenous non-mammalian	3,28E-03
POLR2A	enzyme	3,33E-03
CD3E	transmembrane receptor	3,33E-03
C5AR1	G-protein coupled receptor	3,33E-03
SOCS3	phosphatase	3,34E-03
mir-22	microRNA	3,37E-03
DLK1	other	3,37E-03
CGS 21680	chemical reagent	3,37E-03
fisetin	chemical drug	3,37E-03
fish oils	chemical drug	3,49E-03
ALB	transporter	3,49E-03
CRP	other	3,49E-03
K+	chemical - endogenous mammalian	3,49E-03
tert-butyl hydroperoxide	chemical toxicant	3,56E-03
Ras homolog	group	3,56E-03
TCF/LEF	group	3,56E-03
Lymphotoxin	complex	3,56E-03
GNAI3	enzyme	3,56E-03
SOCS6	other	3,56E-03
RNASE1	enzyme	3,56E-03
A-Fos	chemical reagent	3,56E-03
PPP1R15A	other	3,56E-03
TUG1	other	3,56E-03
PD 169316	chemical drug	3,56E-03
tranylcypromine	chemical drug	3,56E-03
acetaldehyde	chemical - endogenous mammalian	3,56E-03
NUP98-NSD1	fusion gene/product	3,56E-03

elovanoid N34	chemical - endogenous mammalian	3,56E-03
2,4,5,2',4',5'-hexachlorobiphenyl	chemical toxicant	3,65E-03
C3	peptidase	3,67E-03
interferon alfacon-1	biologic drug	3,68E-03
resatorvid	chemical drug	3,68E-03
IKK (complex)	complex	3,68E-03
indican	chemical - endogenous mammalian	3,68E-03
TERC	other	3,68E-03
CA9	enzyme	3,68E-03
ING1	transcription regulator	3,68E-03
NF1	other	3,68E-03
KN 93	chemical drug	3,68E-03
NAMPT	cytokine	3,70E-03
monophosphoryl lipid A	chemical drug	3,72E-03
RUBCN	other	3,72E-03
SELENOS	other	3,72E-03
48s	complex	3,72E-03
IGFBP7	transporter	3,72E-03
ZBTB33	transcription regulator	3,72E-03
PDGF (family)	group	3,72E-03
PCK1	kinase	3,72E-03
NUP62	transporter	3,72E-03
APBB1	transcription regulator	3,72E-03
mir-196	microRNA	3,72E-03
RBM5-AS1	other	3,72E-03
CAPN3	peptidase	3,72E-03
POLDIP2	other	3,72E-03
SIRPA	phosphatase	3,72E-03
SCH772984	chemical drug	3,72E-03
Z-551	chemical reagent	3,72E-03
oligomycin	chemical - endogenous non-mammalian	3,72E-03
monorden	chemical - endogenous non-mammalian	3,72E-03
chlorogenic acid	chemical drug	3,72E-03
NUP98-KDM5A	fusion gene/product	3,72E-03
asiatic acid	chemical reagent	3,72E-03
NSC 172285	chemical reagent	3,73E-03
delta-12-prostaglandin J2	chemical - endogenous mammalian	3,73E-03
eritoran	chemical drug	3,73E-03
L-alpha-lysophosphatidylcholine, palmitoyl	chemical - endogenous mammalian	3,73E-03
1-palmitoyl-2-oleoylglycero-3-phosphoglycerol	chemical reagent	3,73E-03
lunasin	biologic drug	3,73E-03
[Lys15,Arg16,Leu27]VIP(1-7)GRF(8-27)	chemical reagent	3,73E-03
ADRA1	group	3,73E-03
(-)-gossypol	chemical drug	3,73E-03

edratide	biologic drug	3,73E-03
ZBED1	enzyme	3,73E-03
FICD	enzyme	3,73E-03
MARCHF3	other	3,73E-03
FENDRR	other	3,73E-03
Gpcr	group	3,73E-03
JADE2	enzyme	3,73E-03
ZMIZ2	transcription regulator	3,73E-03
SNHG3	other	3,73E-03
SIRT4	enzyme	3,73E-03
HYAL1	enzyme	3,73E-03
Tlr12	other	3,73E-03
miR-873-5p (and other miRNAs w/seed CAGGAAC)	mature microRNA	3,73E-03
miR-292b-5p (and other miRNAs w/seed CUCAAAA)	mature microRNA	3,73E-03
rigosertib	chemical drug	3,73E-03
SRCAP	transcription regulator	3,73E-03
FUT4	enzyme	3,73E-03
KIDINS220	transcription regulator	3,73E-03
ASH2L	transcription regulator	3,73E-03
FDPS	enzyme	3,73E-03
TMSB10/TMSB4X	other	3,73E-03
BBC3	other	3,73E-03
PELI2	enzyme	3,73E-03
CDC27	other	3,73E-03
GNL2	enzyme	3,73E-03
HOXD12	transcription regulator	3,73E-03
NEK2	kinase	3,73E-03
GCLM	enzyme	3,73E-03
MI-773	chemical drug	3,73E-03
scoparone	chemical - endogenous non-mammalian	3,73E-03
picolinic acid	chemical toxicant	3,73E-03
SD-282	chemical drug	3,73E-03
G Protein I	complex	3,73E-03
oxytetracycline	chemical drug	3,73E-03
grepafloxacin	chemical drug	3,73E-03
vanadyl sulfate	chemical reagent	3,73E-03
CBFB-MYH11	fusion gene/product	3,73E-03
mini-GAGR	chemical reagent	3,73E-03
allosamidin	chemical reagent	3,73E-03
acetyl-11-keto-beta-boswellic acid	chemical reagent	3,73E-03
Go 6976	chemical drug	3,78E-03
lipofermata	chemical reagent	3,81E-03
GAS6	growth factor	3,81E-03
HMGN1	transcription regulator	3,81E-03

ACE	peptidase	3,81E-03
SFN	other	3,81E-03
titanium dioxide	chemical drug	3,81E-03
1810019D21Rik	other	3,97E-03
GABPA	transcription regulator	3,97E-03
nickel chloride	chemical toxicant	3,97E-03
NFU1	other	3,98E-03
Laminin (complex)	complex	3,98E-03
Hsp70	group	4,05E-03
HSPA5	enzyme	4,05E-03
IRF9	transcription regulator	4,05E-03
pristane	chemical toxicant	4,05E-03
delta-9-tetrahydrocannabinol	chemical drug	4,10E-03
doxycycline	chemical drug	4,12E-03
CRNDE	other	4,43E-03
TFDP1	transcription regulator	4,43E-03
NGLY1	enzyme	4,43E-03
miR-221-3p (and other miRNAs w/seed GCUACAU)	mature microRNA	4,43E-03
NLRP12	other	4,43E-03
NFIL3	transcription regulator	4,43E-03
Klrk1	transmembrane receptor	4,43E-03
honokiol	chemical - endogenous non-mammalian	4,43E-03
SIRT6	enzyme	4,48E-03
ZNF217	transcription regulator	4,48E-03
RANBP3L	other	4,54E-03
GIP	other	4,54E-03
IRF6	transcription regulator	4,54E-03
GSK2816126	chemical drug	4,54E-03
BML-111	chemical reagent	4,56E-03
niclosamide	chemical drug	4,56E-03
GPX8	enzyme	4,56E-03
PARP	group	4,56E-03
CERS6	transcription regulator	4,56E-03
TLR2/3/4/9	group	4,56E-03
Tlr13	other	4,56E-03
FRS3	other	4,56E-03
nirogacestat	chemical drug	4,56E-03
IL10RB	transmembrane receptor	4,56E-03
ENO1	enzyme	4,56E-03
mir-449	microRNA	4,56E-03
ALOX15B	enzyme	4,56E-03
IL9R	transmembrane receptor	4,56E-03
SELE	transmembrane receptor	4,56E-03
LUM	other	4,56E-03

NCR1	transmembrane receptor	4,56E-03
PEA15	transporter	4,56E-03
3-hydroxybutyric acid	chemical - endogenous mammalian	4,56E-03
KU-55933	chemical drug	4,56E-03
polymyxin B	biologic drug	4,56E-03
perilla alcohol	chemical drug	4,56E-03
tridecanoic acid	chemical - endogenous mammalian	4,56E-03
amphetamine	chemical drug	4,56E-03
salinosporamide A	chemical drug	4,58E-03
MAPK13	kinase	4,58E-03
TNFRSF18	transmembrane receptor	4,58E-03
miR-199a-3p (and other miRNAs w/seed CAGUAGU)	mature microRNA	4,58E-03
MAPK12	kinase	4,58E-03
YAP/TAZ	group	4,58E-03
2-mercaptoacetate	chemical drug	4,58E-03
agmatine	chemical - endogenous mammalian	4,58E-03
POU2F1	transcription regulator	4,64E-03
ADIPOQ	other	4,66E-03
IRS2	enzyme	4,68E-03
verapamil	chemical drug	4,68E-03
CTBP1	enzyme	4,69E-03
1-palmitoyl-2-oleoylphosphatidylserine	chemical - endogenous mammalian	4,70E-03
miR-223-3p (miRNAs w/seed GUCAGUU)	mature microRNA	4,70E-03
mir-26	microRNA	4,70E-03
B2M	transmembrane receptor	4,70E-03
NMU	other	4,70E-03
7(R)-maresin 1	chemical - endogenous mammalian	4,70E-03
citarinostat	chemical drug	4,70E-03
acteoside	chemical - endogenous non-mammalian	4,70E-03
curdlan	chemical - endogenous non-mammalian	4,70E-03
THRA	ligand-dependent nuclear receptor	4,70E-03
KMT2D	transcription regulator	4,77E-03
Rxr	group	4,81E-03
CYP1B1	enzyme	4,81E-03
CCR2	G-protein coupled receptor	4,81E-03
Nos	group	5,08E-03
COL1A1	other	5,08E-03
DPH5	enzyme	5,08E-03
olaparib	chemical drug	5,08E-03
AZD8055	chemical drug	5,08E-03
PRF1	transporter	5,08E-03
LY96	transmembrane receptor	5,08E-03
FCGR1A	transmembrane receptor	5,08E-03
H3-3A/H3-3B	other	5,08E-03

IL2RB	transmembrane receptor	5,08E-03
IRAK2	kinase	5,08E-03
DGAT1	enzyme	5,08E-03
Gm12602	other	5,08E-03
costunolide	chemical - endogenous non-mammalian	5,08E-03
1,1-bis(3'-indolyl)-1-(4-hydroxyphenyl)methane	chemical reagent	5,08E-03
di-2-pyridylketone 4-cyclohexyl-4-methyl-3-thiosemicarbazone	chemical drug	5,08E-03
sanglifehrin A	chemical reagent	5,08E-03
mannose	chemical - endogenous mammalian	5,08E-03
mirdametinib	chemical drug	5,29E-03
deferasirox	chemical drug	5,29E-03
xanthohumol	chemical drug	5,29E-03
PLP1	other	5,29E-03
nicotinic acid	chemical - endogenous mammalian	5,29E-03
rolipram	chemical drug	5,29E-03
HDAC4	transcription regulator	5,38E-03
topiramate	chemical drug	5,40E-03
TNFRSF1B	transmembrane receptor	5,44E-03
MMP3	peptidase	5,44E-03
SMARCA2	transcription regulator	5,46E-03
2-methoxyestradiol	chemical - endogenous mammalian	5,47E-03
ZMPSTE24	peptidase	5,47E-03
ANGPT1	growth factor	5,47E-03
CDK8	kinase	5,47E-03
E. coli serotype 0127B8 lipopolysaccharide	chemical - endogenous non-mammalian	5,53E-03
AZ5576	chemical drug	5,60E-03
Npm	group	5,60E-03
diphosphoryl lipid A	chemical toxicant	5,60E-03
1,4-glucan	chemical - endogenous mammalian	5,60E-03
syringin	chemical - endogenous non-mammalian	5,60E-03
7beta-hydroxycholesterol	chemical - endogenous mammalian	5,60E-03
urtica dioica extract	chemical drug	5,60E-03
4-oxo-2-nonenal	chemical toxicant	5,60E-03
4-nitrobenzoic acid	chemical toxicant	5,60E-03
MAML	group	5,60E-03
MSK1/2	group	5,60E-03
PLA2	group	5,60E-03
CP-724,714	chemical drug	5,60E-03
2-methoxycinnamaldehyde	chemical - endogenous non-mammalian	5,60E-03
Calcb	other	5,60E-03
Rbx1	enzyme	5,60E-03
MBNL3	other	5,60E-03
FBXO25	enzyme	5,60E-03
POPDC2	other	5,60E-03

SGF29	other	5,60E-03
ZBTB49	transcription regulator	5,60E-03
SMYD5	other	5,60E-03
BATF2	transcription regulator	5,60E-03
ZNF385B	other	5,60E-03
DSC2	other	5,60E-03
febuxostat	chemical drug	5,60E-03
NCEH1	enzyme	5,60E-03
PARM1	other	5,60E-03
STAT3/5	group	5,60E-03
IKKA/B	group	5,60E-03
diaziquone	chemical drug	5,60E-03
RPL19	other	5,60E-03
OMG	G-protein coupled receptor	5,60E-03
SELENOP	other	5,60E-03
NMB	other	5,60E-03
miR-708-5p (and other miRNAs w/seed AGGAGCU)	mature microRNA	5,60E-03
miR-16-1-3p (miRNAs w/seed CAGUAUU)	mature microRNA	5,60E-03
miR-292-3p (and other miRNAs w/seed AGUGCCG)	mature microRNA	5,60E-03
mir-331	microRNA	5,60E-03
TPPP3	other	5,60E-03
HPS1	other	5,60E-03
ARHGEF28	other	5,60E-03
CCT5	other	5,60E-03
AZU1	peptidase	5,60E-03
MUC16	other	5,60E-03
DST	other	5,60E-03
VANGL1	other	5,60E-03
PLEKHA1	other	5,60E-03
DDX24	enzyme	5,60E-03
WASHC1	other	5,60E-03
CD8B	other	5,60E-03
RAMP3	G-protein coupled receptor	5,60E-03
PLEKHA2	other	5,60E-03
CDC23	enzyme	5,60E-03
GALR2	G-protein coupled receptor	5,60E-03
3-hydroxydodecanoic acid	chemical - endogenous mammalian	5,60E-03
6-n-octylaminouracil	chemical reagent	5,60E-03
CYP24A1	enzyme	5,60E-03
2-methoxy-N-(3-methyl-2-oxo-1,4-dihydroquinazolin-6-yl)benzenesulfonamide	chemical reagent	5,60E-03
troxerutin	chemical drug	5,60E-03
imidazolo-oxindole PKR inhibitor C16	chemical reagent	5,60E-03
HUS1	kinase	5,60E-03
LPCAT1	enzyme	5,60E-03

mir-434	microRNA	5,60E-03
ATB-346	chemical drug	5,60E-03
E-c-HDMAPP	chemical reagent	5,60E-03
BAI	chemical drug	5,60E-03
Mia2	other	5,60E-03
hydroxyl radical	chemical toxicant	5,60E-03
amsacrine	chemical drug	5,60E-03
desethylamiodarone	chemical - endogenous mammalian	5,60E-03
1-o-hexadecyl-2-o-methyl-rac-glycerol	chemical - kinase inhibitor	5,60E-03
gliclazide	chemical drug	5,60E-03
nisoldipine	chemical drug	5,60E-03
kaolin	chemical drug	5,60E-03
cation	chemical - other	5,60E-03
NVP-BHG712	chemical drug	5,60E-03
LZ1 peptide	chemical reagent	5,60E-03
1,1-bis(3'-indolyl)-1-(4-chlorophenyl)methane	chemical reagent	5,60E-03
moringa oleifera aqueous seed extract	chemical reagent	5,60E-03
moringa oleifera diluted seed extract	chemical reagent	5,60E-03
dapansutrile	chemical drug	5,60E-03
Z-YVAD-FMK	chemical reagent	5,60E-03
Z-WEHD-FMK	chemical - protease inhibitor	5,60E-03
teleocidins	chemical toxicant	5,60E-03
trehalose dimycolate	chemical - other	5,60E-03
tetrahydrouridine	chemical drug	5,60E-03
L-canavanine	chemical - endogenous non-mammalian	5,60E-03
GC-GCR dimer	complex	5,74E-03
vemurafenib	chemical drug	5,74E-03
USP7	peptidase	5,74E-03
CD69	transmembrane receptor	5,74E-03
MST1R	kinase	5,74E-03
DDX25	enzyme	5,75E-03
ETS	group	5,81E-03
PDGF-DD	complex	5,81E-03
ORMDL3	other	5,81E-03
IL17a dimer	complex	5,81E-03
KDM4A	transcription regulator	5,81E-03
SEMA7A	transmembrane receptor	5,81E-03
AURKA	kinase	5,81E-03
DEF6	other	5,81E-03
mir-101	microRNA	5,81E-03
BAP1	peptidase	5,81E-03
FSHB	other	5,81E-03
ACTB	other	5,81E-03
CSNK2A1	kinase	5,81E-03

diallyl trisulfide	chemical - endogenous non-mammalian	5,81E-03
RXRA	ligand-dependent nuclear receptor	5,84E-03
maslinic acid	chemical - endogenous non-mammalian	6,13E-03
lipoteichoic acid	chemical - endogenous non-mammalian	6,13E-03
ruxolitinib	chemical drug	6,19E-03
Am 580	chemical reagent	6,19E-03
FGF19	growth factor	6,23E-03
NEDD9	other	6,23E-03
LAMP2	enzyme	6,24E-03
SOX3	transcription regulator	6,24E-03
monobutyl phthalate	chemical toxicant	6,26E-03
KDM2B	enzyme	6,26E-03
MEX3A	other	6,26E-03
arginine	chemical - endogenous mammalian	6,26E-03
NEUROG2	transcription regulator	6,26E-03
mir-130	microRNA	6,26E-03
CYBB	enzyme	6,26E-03
Tlr	group	6,27E-03
Wnt	group	6,36E-03
VitaminD3-VDR-RXR	complex	6,36E-03
PRKACA	kinase	6,36E-03
ILK	kinase	6,36E-03
N-acetylsphingosine	chemical reagent	6,36E-03
MYF6	transcription regulator	6,52E-03
SIX2	transcription regulator	6,64E-03
E2F6	transcription regulator	6,64E-03
L-serine	chemical - endogenous mammalian	6,74E-03
TASP1	peptidase	6,74E-03
APOL1	transporter	6,74E-03
PARP14	enzyme	6,74E-03
YOD1	enzyme	6,74E-03
SMOC2	other	6,74E-03
FFAR1	G-protein coupled receptor	6,74E-03
CYSLTR2	G-protein coupled receptor	6,74E-03
astragalin	chemical - endogenous non-mammalian	6,74E-03
SWAP70	other	6,74E-03
RUVBL2	transcription regulator	6,74E-03
AIMP2	other	6,74E-03
CHRNA3	transmembrane receptor	6,74E-03
CNB-001	chemical reagent	6,74E-03
sesame oil	chemical reagent	6,74E-03
TPCA-1	chemical drug	6,74E-03
clonidine	chemical drug	6,74E-03
Trolox C	chemical drug	6,74E-03

manumycin A	chemical reagent	6,74E-03
lidocaine	chemical drug	6,74E-03
SD6	chemical reagent	6,74E-03
carmustine	chemical drug	6,74E-03
enoxacin	chemical drug	6,74E-03
adenine	chemical - endogenous mammalian	6,76E-03
picropodophyllin	chemical drug	6,76E-03
triclosan	chemical drug	6,76E-03
ZC3H14	other	6,76E-03
CHD1	enzyme	6,76E-03
PTBP1	enzyme	6,76E-03
LBP	transporter	6,76E-03
LAMA5	other	6,76E-03
IL1RAP	transmembrane receptor	6,76E-03
DNAJB6	transcription regulator	6,76E-03
NCAM1	other	6,76E-03
HOXB4	transcription regulator	6,76E-03
DDX17	enzyme	6,76E-03
HELLS	enzyme	6,76E-03
KLK5	peptidase	6,76E-03
IL19	cytokine	6,76E-03
premarin	chemical drug	6,76E-03
ethyl pyruvate	chemical drug	6,76E-03
D609	chemical reagent	6,76E-03
KT5823	chemical - kinase inhibitor	6,76E-03
ethidium	chemical drug	6,76E-03
miR-30c-5p (and other miRNAs w/seed GUAAACA)	mature microRNA	6,82E-03
enterotoxin B	biologic drug	6,94E-03
MAC	complex	6,94E-03
MDM4	transcription regulator	6,94E-03
mir-132	microRNA	6,94E-03
mir-31	microRNA	6,94E-03
miR-26a-5p (and other miRNAs w/seed UCAAGUA)	mature microRNA	6,94E-03
IFRD1	other	6,94E-03
PTPN22	phosphatase	6,94E-03
pilocarpine	chemical drug	6,94E-03
MCB-613	chemical reagent	7,05E-03
CYD0618	chemical reagent	7,05E-03
ganciclovir	chemical drug	7,05E-03
conduritol epoxide	chemical reagent	7,05E-03
GPIIB-IIIA	complex	7,05E-03
GTP	chemical - endogenous mammalian	7,05E-03
5-stearic acid hydroxy stearic acid	chemical - endogenous mammalian	7,05E-03
10-(9Z-hexadecenoyloxy)-octadecanoic acid	chemical - endogenous mammalian	7,05E-03

(R)-limonene	chemical toxicant	7,05E-03
VAV	group	7,05E-03
F Actin	complex	7,05E-03
Dgk	group	7,05E-03
zinc oxide	chemical drug	7,05E-03
obatoclax	chemical drug	7,05E-03
glycitein	chemical drug	7,05E-03
SLAMF9	other	7,05E-03
Snhg20	other	7,05E-03
N-propargyl-1(S)-aminoindan	chemical reagent	7,05E-03
HAND2-AS1	other	7,05E-03
SLC39A9	transporter	7,05E-03
TLR10	transmembrane receptor	7,05E-03
RASSF3	other	7,05E-03
IKBIP	other	7,05E-03
TMEM106A	other	7,05E-03
ZDHHC2	enzyme	7,05E-03
RAB7	group	7,05E-03
vaccenic acid	chemical - endogenous mammalian	7,05E-03
TFPI	other	7,05E-03
PRKD2	kinase	7,05E-03
CSE1L	transporter	7,05E-03
HSPB2	other	7,05E-03
MKI67	other	7,05E-03
CNGB3	ion channel	7,05E-03
NEK10	kinase	7,05E-03
BCL2L12	other	7,05E-03
HAS1	enzyme	7,05E-03
mir-708	microRNA	7,05E-03
mir-548	microRNA	7,05E-03
APAF1	other	7,05E-03
ORM1	other	7,05E-03
MAGED1	transcription regulator	7,05E-03
AATF	transcription regulator	7,05E-03
CHD3	enzyme	7,05E-03
SRPK1	kinase	7,05E-03
LRIG1	other	7,05E-03
DYNLL1	other	7,05E-03
STK38	kinase	7,05E-03
CASP2	peptidase	7,05E-03
STX2	transporter	7,05E-03
URI1	transcription regulator	7,05E-03
RNF17	other	7,05E-03
CDC20	other	7,05E-03

CBLC	enzyme	7,05E-03
HIVEP2	transcription regulator	7,05E-03
RPS20	other	7,05E-03
OMP-52M51	biologic drug	7,05E-03
CXXC4	other	7,05E-03
BCL2A1	other	7,05E-03
fenebrutinib	chemical drug	7,05E-03
EGTA acetoxymethyl ester	chemical reagent	7,05E-03
naphthalene	chemical toxicant	7,05E-03
pyridoxamine	chemical - endogenous mammalian	7,05E-03
risedronic acid	chemical drug	7,05E-03
octyl gallate	chemical reagent	7,05E-03
catechol	chemical - endogenous mammalian	7,05E-03
FC-99	chemical reagent	7,05E-03
PDZ1i	chemical reagent	7,05E-03
monodansylcadaverine	chemical reagent	7,05E-03
Z-IETD-FMK	chemical - protease inhibitor	7,05E-03
propyl-2-(8-(3,4-difluorobenzyl)-2',5'-dioxo-8-azaspiro[bicyclo[3.2.1] octane-3,4'-imidazolidine]-1'-yl)acetate	chemical reagent	7,05E-03
sparfosic acid	chemical drug	7,05E-03
L-asparagine	chemical - endogenous mammalian	7,05E-03
oxamic acid	chemical - endogenous non-mammalian	7,05E-03
quisqualic acid	chemical - endogenous non-mammalian	7,05E-03
9-palmitic acid hydroxy stearic acid	chemical - endogenous mammalian	7,05E-03
SATB2	transcription regulator	7,07E-03
miR-27a-3p (and other miRNAs w/seed UCACAGU)	mature microRNA	7,07E-03
N-[N-(3,5-difluorophenacetyl-L-Ala)]-S-phenylglycine t-butyl ester	chemical - protease inhibitor	7,13E-03
wood smoke particle	chemical reagent	7,27E-03
oleoyl-estrone	chemical drug	7,27E-03
SULF2	enzyme	7,27E-03
RNF138	enzyme	7,27E-03
TF	transporter	7,27E-03
miR-18a-5p (and other miRNAs w/seed AAGGUGC)	mature microRNA	7,27E-03
NT5E	phosphatase	7,27E-03
HOXA5	transcription regulator	7,27E-03
TNFAIP6	other	7,27E-03
DYRK1A	kinase	7,27E-03
CYP2J2	enzyme	7,27E-03
Cdkn1c	other	7,27E-03
FMR1	translation regulator	7,33E-03
CLEC10A	other	7,37E-03
NSD2	enzyme	7,37E-03
OVA-8	chemical reagent	7,37E-03
ARRB2	other	7,37E-03
anandamide	chemical - endogenous mammalian	7,37E-03

N-methyl-D-aspartate	chemical drug	7,49E-03
TRAP1	enzyme	7,59E-03
MSTN	growth factor	7,68E-03
KCNIP3	transcription regulator	7,68E-03
PRKD1	kinase	7,68E-03
SMPD1	enzyme	8,05E-03
TIRAP	other	8,33E-03
ALDH1A2	enzyme	8,33E-03
MAPK11	kinase	8,33E-03
NOSTRIN	transcription regulator	8,33E-03
KNG1	other	8,33E-03
UXT	transcription regulator	8,33E-03
HSPD1	enzyme	8,33E-03
NCOA4	transcription regulator	8,33E-03
irbesartan	chemical drug	8,33E-03
PTK2	kinase	8,42E-03
lysophosphatidylinositol	chemical - endogenous mammalian	8,47E-03
Hsp27	group	8,47E-03
acetovanillone	chemical drug	8,47E-03
NUP98-DDX10	fusion gene/product	8,47E-03
epinephrine	chemical - endogenous mammalian	8,51E-03
FLT3LG	cytokine	8,55E-03
ASAH1	enzyme	8,63E-03
THBS1	other	8,63E-03
FCER1G	transmembrane receptor	8,63E-03
FOXP1	transcription regulator	8,63E-03
histone deacetylase	complex	8,63E-03
Ren2	peptidase	8,79E-03
Fcgr3	group	8,79E-03
HCAR2	G-protein coupled receptor	8,79E-03
LRBA	other	8,79E-03
METTL1	enzyme	8,79E-03
CNOT6L	enzyme	8,79E-03
C-21	chemical drug	8,79E-03
hemoglobin	complex	8,79E-03
Cyp2c23	enzyme	8,79E-03
mir-194	microRNA	8,79E-03
CHEK2	kinase	8,79E-03
PDGFRA	kinase	8,79E-03
GPI	enzyme	8,79E-03
AKR1B1	enzyme	8,79E-03
CCR6	G-protein coupled receptor	8,79E-03
PARP9	enzyme	8,79E-03
clenbuterol	chemical drug	8,79E-03

acadesine	chemical drug	8,79E-03
NfkB-RelA	complex	8,97E-03
PTPN2	phosphatase	8,97E-03
LRPAP1	other	8,97E-03
UBE3A	enzyme	8,97E-03
GAS5	other	8,97E-03
oxazolone	chemical reagent	8,97E-03
GATA4	transcription regulator	9,02E-03
mir-146	microRNA	9,12E-03
LTA	cytokine	9,12E-03
ABCA1	transporter	9,12E-03
ESRRG	ligand-dependent nuclear receptor	9,40E-03
PNPLA2	enzyme	9,40E-03
miR-122-5p (miRNAs w/seed GGAGUGU)	mature microRNA	9,44E-03
BBP-398	chemical drug	9,52E-03
naringin	chemical - endogenous non-mammalian	9,52E-03
gossypin	chemical - endogenous non-mammalian	9,52E-03
CERS2	transcription regulator	9,52E-03
POLR3G	enzyme	9,52E-03
WWC1	transcription regulator	9,52E-03
SLC7A2	transporter	9,52E-03
DEPTOR	other	9,52E-03
AFAP1-AS1	other	9,52E-03
FLZ	chemical drug	9,52E-03
BMS-754807	chemical drug	9,52E-03
PRAME	other	9,52E-03
IL1R2	transmembrane receptor	9,52E-03
RB1CC1	other	9,52E-03
DDB2	other	9,52E-03
CCNT1	transcription regulator	9,52E-03
PAX4	transcription regulator	9,52E-03
PINK1	kinase	9,52E-03
KRIT1	other	9,52E-03
ITGB8	other	9,52E-03
BEX2	other	9,52E-03
NDP	growth factor	9,52E-03
pentylenetetrazol	chemical drug	9,52E-03
indinavir	chemical drug	9,52E-03
rhein	chemical - endogenous non-mammalian	9,52E-03
mesalamine	chemical drug	9,52E-03
pepstatin	chemical - protease inhibitor	9,52E-03
dehydrocostus lactone	chemical - endogenous non-mammalian	9,52E-03
thymidine	chemical - endogenous mammalian	9,52E-03
androstenediol	chemical - endogenous mammalian	9,52E-03

EGLN	group	9,67E-03
SCARB1	transporter	9,70E-03
LTBR	transmembrane receptor	9,70E-03
SMC1A	transporter	9,70E-03
TCF7	transcription regulator	9,70E-03
testosterone propionate	chemical drug	9,70E-03
ARHGAP31	other	9,90E-03
mir-124	microRNA	9,90E-03
HAVCR2	other	9,90E-03
ICMT	enzyme	9,90E-03
NFIC	transcription regulator	9,90E-03
CX-5461	chemical drug	9,90E-03
SPARC	other	1,00E-02
KEAP1	transcription regulator	1,00E-02
TNIP1	other	1,00E-02
memantine	chemical drug	1,00E-02
PDLIM2	other	1,01E-02
PSMB11	peptidase	1,02E-02
ELL2	transcription regulator	1,03E-02
MNT	transcription regulator	1,03E-02
3,4,5,3',4'-pentachlorobiphenyl	chemical toxicant	1,04E-02
MAVS	other	1,04E-02
FGFR2	kinase	1,04E-02
SOD2	enzyme	1,05E-02
vitamin E	chemical drug	1,05E-02
EGLN1	enzyme	1,05E-02
BACH1	transcription regulator	1,05E-02
leukotriene B4	chemical - endogenous mammalian	1,09E-02
lnc-CXCL2-4	other	1,09E-02
SIGIRR	transmembrane receptor	1,09E-02
AHI1	other	1,09E-02
CDC73	other	1,09E-02
trametinib	chemical drug	1,09E-02
Trp53cor1	other	1,09E-02
MTA3	transcription regulator	1,09E-02
HEXIM1	transcription regulator	1,09E-02
Saa3	other	1,09E-02
NOS1	enzyme	1,09E-02
HMGB2	transcription regulator	1,09E-02
alpha-tocopherol	chemical drug	1,09E-02
chrysin	chemical - endogenous non-mammalian	1,09E-02
DNMT1	enzyme	1,10E-02
DSCAM	other	1,10E-02
NR4A2	ligand-dependent nuclear receptor	1,10E-02

mir-145	microRNA	1,11E-02
PNPT1	enzyme	1,11E-02
3M-002	chemical reagent	1,11E-02
ATN1	transcription regulator	1,12E-02
MEF2C	transcription regulator	1,12E-02
neomycin	chemical drug	1,12E-02
NX-13	chemical reagent	1,12E-02
Fgfr	group	1,12E-02
FAT1	other	1,12E-02
SEL1L	other	1,12E-02
miR-503-5p (miRNAs w/seed AGCAGCG)	mature microRNA	1,12E-02
ponesimod	chemical drug	1,12E-02
S1PR2	G-protein coupled receptor	1,12E-02
BIRC2	enzyme	1,12E-02
TEK	kinase	1,12E-02
MUC4	other	1,12E-02
NEDD4	enzyme	1,12E-02
tomatidine	chemical - endogenous non-mammalian	1,12E-02
triflusal	chemical drug	1,12E-02
diethylmaleate	chemical toxicant	1,12E-02
L-carnitine	chemical - endogenous mammalian	1,12E-02
MR-409	chemical reagent	1,12E-02
riboflavin	chemical - endogenous mammalian	1,12E-02
dichlororibofuranosylbenzimidazole	chemical toxicant	1,12E-02
melphalan	chemical drug	1,12E-02
GW501516	chemical drug	1,15E-02
EP4-D	chemical reagent	1,16E-02
PRKCG	kinase	1,16E-02
AKT2	kinase	1,16E-02
Mir122a,b	group	1,16E-02
ibuprofen	chemical drug	1,16E-02
1,2-dimethylhydrazine	chemical toxicant	1,16E-02
lactic acid	chemical - endogenous mammalian	1,16E-02
naringenin	chemical - endogenous non-mammalian	1,16E-02
olomoucine	chemical - kinase inhibitor	1,17E-02
alloxan	chemical reagent	1,17E-02
blinatumomab	biologic drug	1,17E-02
triamcinolone hexacetonide	chemical drug	1,17E-02
rh-endostatin	chemical drug	1,17E-02
4732491K20Rik	other	1,17E-02
patulin	chemical toxicant	1,17E-02
Tlr11	transmembrane receptor	1,17E-02
SCGB3A2	other	1,17E-02
ST6GALNAC1	enzyme	1,17E-02

NSUN2	enzyme	1,17E-02
SH2D5	other	1,17E-02
BCAS2	other	1,17E-02
TP73-AS1	other	1,17E-02
KMT5B	enzyme	1,17E-02
SFRP5	transmembrane receptor	1,17E-02
RDH8	enzyme	1,17E-02
APC (complex)	complex	1,17E-02
catumaxomab	biologic drug	1,17E-02
HLA-DQ	complex	1,17E-02
IER3	other	1,17E-02
WNT2	cytokine	1,17E-02
NUDT16L1	other	1,17E-02
MEFV	other	1,17E-02
KLF12	transcription regulator	1,17E-02
AGR2	other	1,17E-02
CCDC50	other	1,17E-02
SERPINA4	other	1,17E-02
miR-494-3p (miRNAs w/seed GAAACAU)	mature microRNA	1,17E-02
SDC4	other	1,17E-02
ST13	other	1,17E-02
GATAD2B	transcription regulator	1,17E-02
MARCO	transmembrane receptor	1,17E-02
TP53INP1	other	1,17E-02
GNL1	other	1,17E-02
C4A/C4B	other	1,17E-02
MLLT3	transcription regulator	1,17E-02
TDG	enzyme	1,17E-02
GJA8	transporter	1,17E-02
casticin	chemical - endogenous non-mammalian	1,17E-02
ADCYAP1R1	G-protein coupled receptor	1,17E-02
RAPGEF4	other	1,17E-02
ABCB7	transporter	1,17E-02
CTH	enzyme	1,17E-02
KLRB1	transmembrane receptor	1,17E-02
CLEC2A	other	1,17E-02
MARCHF1	enzyme	1,17E-02
JMY	transcription regulator	1,17E-02
enzastaurin	chemical drug	1,17E-02
nicorandil	chemical drug	1,17E-02
ubiquinone 9	chemical - endogenous mammalian	1,17E-02
trimethyltin	chemical reagent	1,17E-02
autologous CD22-targeted CAR-T cells	biologic drug	1,17E-02
fluphenazine	chemical drug	1,17E-02

RWJ 67657	chemical drug	1,17E-02
batatasin I	chemical - endogenous non-mammalian	1,17E-02
vardenafil	chemical drug	1,17E-02
leucovorin	chemical - endogenous mammalian	1,17E-02
murabutide	chemical reagent	1,17E-02
muscarine	chemical toxicant	1,17E-02
borrelia burgdorferi strain B31 peptidoglycan	chemical reagent	1,17E-02
IL23A	cytokine	1,17E-02
MALT1	peptidase	1,17E-02
mir-135	microRNA	1,17E-02
ANXA2	other	1,17E-02
TIMP1	cytokine	1,17E-02
DAXX	transcription regulator	1,17E-02
CBX3	transcription regulator	1,17E-02
MMP12	peptidase	1,18E-02
PRKCB	kinase	1,18E-02
monocrotaline	chemical toxicant	1,18E-02
adavosertib	chemical drug	1,20E-02
RABGEF1	enzyme	1,26E-02
AVP	other	1,26E-02
IFNA4	cytokine	1,26E-02
Esrra	transcription regulator	1,27E-02
ZFPM1	transcription regulator	1,29E-02
CXCL6	cytokine	1,29E-02
ladostigil	chemical drug	1,29E-02
trypsin	group	1,29E-02
KDM4D	enzyme	1,29E-02
TRIM38	enzyme	1,29E-02
MDGA2	other	1,29E-02
DHCR24	enzyme	1,29E-02
LECT2	other	1,29E-02
PLA2G2D	enzyme	1,29E-02
fedratinib	chemical drug	1,29E-02
alisertib	chemical drug	1,29E-02
CSNK2B	kinase	1,29E-02
KLRG1	other	1,29E-02
PAK4	kinase	1,29E-02
LIPA	enzyme	1,29E-02
mir-185	microRNA	1,29E-02
miR-101-3p (and other miRNAs w/seed ACAGUAC)	mature microRNA	1,29E-02
mir-127	microRNA	1,29E-02
HIF3A	transcription regulator	1,29E-02
THBS2	other	1,29E-02
CRY2	enzyme	1,29E-02

BCAR1	enzyme	1,29E-02
PDE4B	enzyme	1,29E-02
CD180	other	1,29E-02
BCR	kinase	1,29E-02
CALCB	other	1,29E-02
GHSR	G-protein coupled receptor	1,29E-02
PIM3	kinase	1,29E-02
AK1	kinase	1,29E-02
PLA2G2E	enzyme	1,29E-02
FZR1	kinase	1,29E-02
CRY1	enzyme	1,29E-02
OPRM1	G-protein coupled receptor	1,29E-02
4-hydroxycinnamyl aldehyde	chemical - endogenous non-mammalian	1,29E-02
spermine nitric oxide complex	chemical toxicant	1,29E-02
KMT2A-AFF1	fusion gene/product	1,29E-02
astressin 2B	biologic drug	1,29E-02
ryanodine	chemical - endogenous non-mammalian	1,29E-02
gamma-linolenic acid	chemical - endogenous mammalian	1,29E-02
gambogic acid	chemical - endogenous non-mammalian	1,29E-02
pectin	chemical drug	1,29E-02
S-equol	chemical drug	1,29E-02
IL1R1	transmembrane receptor	1,30E-02
ACSL4	enzyme	1,30E-02
heme	chemical - endogenous mammalian	1,30E-02
thioctic acid	chemical drug	1,30E-02
miR-205-5p (and other miRNAs w/seed CCUUCAU)	mature microRNA	1,32E-02
mir-144	microRNA	1,32E-02
LTBP1	other	1,32E-02
XIAP	enzyme	1,32E-02
LTF	peptidase	1,32E-02
nilvadipine	chemical drug	1,32E-02
WR 1065	chemical drug	1,32E-02
methyl-beta-cyclodextrin	chemical drug	1,32E-02
RPS6KB1	kinase	1,33E-02
ARHGAP21	other	1,33E-02
GATA2	transcription regulator	1,35E-02
Ap2	group	1,37E-02
mir-221	microRNA	1,37E-02
PAK1	kinase	1,37E-02
carvedilol	chemical drug	1,37E-02
sodium orthovanadate	chemical reagent	1,37E-02
IL7R	transmembrane receptor	1,37E-02
SCAP	other	1,37E-02
HNRNPA2B1	other	1,38E-02

dacinostat	chemical drug	1,40E-02
peoniflorin	chemical drug	1,40E-02
MSI2	other	1,40E-02
IFNW1	cytokine	1,40E-02
CYB5R4	enzyme	1,40E-02
ERVW-1	other	1,40E-02
NOD1	other	1,40E-02
TNFRSF13C	transmembrane receptor	1,40E-02
mir-192	microRNA	1,40E-02
IAPP	other	1,40E-02
KCNJ10	ion channel	1,40E-02
RFX1	transcription regulator	1,40E-02
LY6E	other	1,40E-02
STAU1	transporter	1,40E-02
TMBIM6	other	1,40E-02
GFAP	other	1,40E-02
antimycin A	chemical - endogenous non-mammalian	1,40E-02
DGCR8	enzyme	1,42E-02
SRC (family)	group	1,42E-02
epicatechin	chemical drug	1,43E-02
LATS2	kinase	1,45E-02
EHMT2	transcription regulator	1,45E-02
valsartan	chemical drug	1,45E-02
SMAD2	transcription regulator	1,48E-02
PAX6	transcription regulator	1,52E-02
Collagen type II	complex	1,53E-02
EIF3E	translation regulator	1,53E-02
TXNRD1	enzyme	1,53E-02
PTPRC	phosphatase	1,53E-02
fasudil	chemical drug	1,53E-02
NCOR1	transcription regulator	1,55E-02
olanzapine	chemical drug	1,55E-02
Histone h2a	group	1,57E-02
sodium selenite	chemical drug	1,57E-02
p70 S6k	group	1,57E-02
dimethyl itaconate	chemical reagent	1,57E-02
TLX1	transcription regulator	1,57E-02
NCL	other	1,57E-02
CDKN2B	transcription regulator	1,57E-02
MAP2K2	kinase	1,57E-02
ADA	enzyme	1,57E-02
CARD11	kinase	1,57E-02
TFPI2	other	1,57E-02
CD47	transmembrane receptor	1,57E-02

JAK inhibitor I	chemical drug	1,57E-02
alendronic acid	chemical drug	1,57E-02
UM101	chemical drug	1,57E-02
CpG ODN 2216	chemical reagent	1,59E-02
Stat3-Stat3	complex	1,59E-02
ETV4	transcription regulator	1,59E-02
miR-450a-5p (and other miRNAs w/seed UUUGCGA)	mature microRNA	1,59E-02
COL5A1	other	1,59E-02
AIM2	other	1,59E-02
butylated hydroxyanisol	chemical toxicant	1,59E-02
[(R)-6-(4-(4-benzyl-7-chloronaphthalen-1-yl)-2-methylpiperazin-1-yl)] nicotine nitrile	chemical reagent	1,60E-02
lopinavir	chemical drug	1,60E-02
trapidil	chemical drug	1,60E-02
pentosan polysulfate	chemical drug	1,60E-02
PDK	group	1,60E-02
mepazine	chemical drug	1,60E-02
galactosylceramide	chemical - endogenous mammalian	1,60E-02
GDP	chemical - endogenous mammalian	1,60E-02
enoxaparin	chemical drug	1,60E-02
C1QTNF12	other	1,60E-02
Cyclin B	group	1,60E-02
DUB	group	1,60E-02
lipoxygenase	group	1,60E-02
chlormethiazole	chemical drug	1,60E-02
Esr1-Estrogen-Sp1	complex	1,60E-02
cetylpyridinium	chemical drug	1,60E-02
irisolidone	chemical - endogenous non-mammalian	1,60E-02
GALNT14	enzyme	1,60E-02
TMBIM1	other	1,60E-02
RHOBTB2	enzyme	1,60E-02
USP6	peptidase	1,60E-02
PRMT7	enzyme	1,60E-02
LINC00261	other	1,60E-02
PTPMT1	phosphatase	1,60E-02
CERS4	transcription regulator	1,60E-02
SPON2	other	1,60E-02
SMCR8	other	1,60E-02
PRR7	other	1,60E-02
EDEM1	enzyme	1,60E-02
OTUB2	enzyme	1,60E-02
Fascin	group	1,60E-02
TAFA4	other	1,60E-02
STAB1	transporter	1,60E-02
C1QL1	other	1,60E-02

ZNF300	transcription regulator	1,60E-02
potassium channel	group	1,60E-02
NAA30	enzyme	1,60E-02
DGKH	kinase	1,60E-02
TRPM3	ion channel	1,60E-02
KLHL21	enzyme	1,60E-02
ATP13A2	transporter	1,60E-02
NAAA	enzyme	1,60E-02
GPD1L	enzyme	1,60E-02
EIF4F	complex	1,60E-02
TH17 Cytokine	group	1,60E-02
ATP1A2	transporter	1,60E-02
VX 702	chemical drug	1,60E-02
Gamma tubulin	group	1,60E-02
Inflammasome	complex	1,60E-02
pimasertib	chemical drug	1,60E-02
CASQ1	other	1,60E-02
satratoxin G	chemical toxicant	1,60E-02
Pdi	group	1,60E-02
foretinib	chemical drug	1,60E-02
GPC3	other	1,60E-02
VAMP7	transporter	1,60E-02
ARFGEF2	other	1,60E-02
NAB1	transcription regulator	1,60E-02
PMEPA1	other	1,60E-02
GNLY	other	1,60E-02
PURB	transcription regulator	1,60E-02
GSTA4	enzyme	1,60E-02
Ccdc50	other	1,60E-02
MCRS1	other	1,60E-02
U2AF2	other	1,60E-02
LYZ	enzyme	1,60E-02
MADD	other	1,60E-02
miR-515-3p (and other miRNAs w/seed AGUGCCU)	mature microRNA	1,60E-02
miR-2682-5p (and other miRNAs w/seed AGGCAGU)	mature microRNA	1,60E-02
miR-296-3p (miRNAs w/seed AGGGUUG)	mature microRNA	1,60E-02
miR-376a-5p (miRNAs w/seed UAGAUUC)	mature microRNA	1,60E-02
miR-125b-1-3p (miRNAs w/seed CGGGUUA)	mature microRNA	1,60E-02
miR-149-3p (and other miRNAs w/seed GGGAGGG)	mature microRNA	1,60E-02
P4HB	enzyme	1,60E-02
RPS14	translation regulator	1,60E-02
PBK	kinase	1,60E-02
HSPB3	other	1,60E-02
CARD8	other	1,60E-02

aurora kinase inhibitor III	chemical - kinase inhibitor	1,60E-02
SGI 1776	chemical drug	1,60E-02
FOXO6	transcription regulator	1,60E-02
TPM1	other	1,60E-02
DOK2	other	1,60E-02
USP14	peptidase	1,60E-02
FTMT	enzyme	1,60E-02
WWP1	enzyme	1,60E-02
GZMA	peptidase	1,60E-02
STRAP	other	1,60E-02
CRT-0066101	chemical drug	1,60E-02
TOR2A	other	1,60E-02
ZNF350	transcription regulator	1,60E-02
KPNB1	other	1,60E-02
PDZD2	other	1,60E-02
TXNDC5	enzyme	1,60E-02
LRG1	other	1,60E-02
RBM3	other	1,60E-02
GJC1	ion channel	1,60E-02
MXD4	transcription regulator	1,60E-02
Ank2	other	1,60E-02
GNG2	enzyme	1,60E-02
PAGR1	other	1,60E-02
IDR-1018	chemical reagent	1,60E-02
INHBC	growth factor	1,60E-02
VEZF1	transcription regulator	1,60E-02
CASP8AP2	transcription regulator	1,60E-02
zotiraciclib	chemical drug	1,60E-02
TCP1	other	1,60E-02
8-butyl-10-[(3-methoxy-5-pyrrol-2-ylidenepyrrol-2-ylidene)methyl]-11-azabicyclo[7.2.1]dodeca-1(12),9-diene	chemical reagent	1,60E-02
MXD3	transcription regulator	1,60E-02
RING1	transcription regulator	1,60E-02
EEF1A1	translation regulator	1,60E-02
RPL37	other	1,60E-02
Acp5	phosphatase	1,60E-02
Irs4	other	1,60E-02
RPL23A	other	1,60E-02
Supt20	other	1,60E-02
BUB1B	kinase	1,60E-02
capivasertib	chemical drug	1,60E-02
UIMC1	other	1,60E-02
JPH203	chemical reagent	1,60E-02
AZ-960	chemical reagent	1,60E-02
iso[4]levuglandin E2	chemical - endogenous mammalian	1,60E-02

CYM50358	chemical reagent	1,60E-02
Ro-1138452	chemical reagent	1,60E-02
mercaptosteroid 4	chemical reagent	1,60E-02
mercaptosteroid 6	chemical reagent	1,60E-02
beta2 adrenergic receptor agonist	chemical drug	1,60E-02
14-oxoDHA	chemical - endogenous mammalian	1,60E-02
N6-carboxymethyl-lysine bovine serum albumin	chemical reagent	1,60E-02
LY3214996	chemical drug	1,60E-02
compound 48/80	chemical reagent	1,60E-02
polyhydroxyethyl methacrylate	chemical reagent	1,60E-02
nitrous oxide	chemical drug	1,60E-02
tempo	chemical reagent	1,60E-02
ODN1411	chemical reagent	1,60E-02
RX-821002	chemical reagent	1,60E-02
cibacron blue F 3GA	chemical reagent	1,60E-02
bongkrekic acid	chemical toxicant	1,60E-02
phosphorylcholine	chemical - endogenous mammalian	1,60E-02
glyceollin	chemical - endogenous non-mammalian	1,60E-02
3,4-dihydroxybenzaldehyde	chemical - endogenous non-mammalian	1,60E-02
N-methylsulfonyl-12,12-dibromododec-11-enamide	chemical reagent	1,60E-02
benzamide	chemical reagent	1,60E-02
1-heptanol	chemical reagent	1,60E-02
incyclinide	chemical drug	1,60E-02
10-decarbamoylmitomycin C	chemical toxicant	1,60E-02
idronoxil	chemical drug	1,60E-02
taprostene	chemical reagent	1,60E-02
4-amino-6-hydrazino-7-beta-D-ribofuranosyl-7H-pyrrolo[2,3-d]-pyrimidine-5-carboxamide	chemical reagent	1,60E-02
silver nitrate	chemical drug	1,60E-02
methoxyluteolin	chemical - endogenous non-mammalian	1,60E-02
potassium tetraperoxochromate	chemical reagent	1,60E-02
REC2923	chemical reagent	1,60E-02
3,4-methylenedioxyamphetamine	chemical drug	1,60E-02
Ala-Tyr-Pro-Gly-Lys-Phe-NH2	chemical reagent	1,60E-02
TAPI	chemical - protease inhibitor	1,60E-02
bacitracin	biologic drug	1,60E-02
ODN-BW006	chemical reagent	1,60E-02
ellipticine	chemical reagent	1,60E-02
3,7-dimethyl-1-propargylxanthine	chemical reagent	1,60E-02
strychnine	chemical drug	1,60E-02
MEDICA 16	chemical reagent	1,60E-02
AVI-4126	biologic drug	1,60E-02
phosphorothioate oligodeoxynucleotide	chemical - other	1,60E-02
glyphosate	chemical toxicant	1,60E-02
pegylated leptin antagonist	chemical reagent	1,60E-02

danazol	chemical drug	1,60E-02
epiallopregnanolone	chemical - endogenous mammalian	1,60E-02
1,4-bis[2-(3,5-dichloropyridyloxy)]benzene	chemical toxicant	1,60E-02
JAK1/2	group	1,60E-02
MAP2K3	kinase	1,60E-02
CD86	transmembrane receptor	1,60E-02
bicalutamide	chemical drug	1,60E-02
chondroitin sulfate A	chemical - endogenous mammalian	1,71E-02
Actin	group	1,71E-02
belnacasan	chemical drug	1,71E-02
TRAF3IP3	other	1,71E-02
withaferin A	chemical reagent	1,71E-02
LOXL1	enzyme	1,71E-02
PECAM1	other	1,71E-02
LIMS1	other	1,71E-02
TAC4	other	1,71E-02
CBX2	transcription regulator	1,71E-02
mir-302	microRNA	1,71E-02
miR-196a-5p (and other miRNAs w/seed AGGUAGU)	mature microRNA	1,71E-02
F10	peptidase	1,71E-02
SRSF3	other	1,71E-02
FCER2	transmembrane receptor	1,71E-02
erastin	chemical drug	1,71E-02
CDK6	kinase	1,71E-02
MSI1	other	1,71E-02
BMX	kinase	1,71E-02
LAMC1	other	1,71E-02
HOXA4	transcription regulator	1,71E-02
CELF1	translation regulator	1,71E-02
UBR5	enzyme	1,71E-02
coomassie brilliant blue	chemical drug	1,71E-02
picryl chloride	chemical toxicant	1,71E-02
pyruvic acid	chemical - endogenous mammalian	1,71E-02
nelfinavir	chemical drug	1,71E-02
Aldose Reductase	group	1,73E-02
SRA1	transcription regulator	1,73E-02
SPOP	other	1,73E-02
SPZ1	transcription regulator	1,73E-02
SP100	transcription regulator	1,73E-02
miR-293-5p (and other miRNAs w/seed CUCAAAC)	mature microRNA	1,73E-02
miR-31-5p (and other miRNAs w/seed GGCAAGA)	mature microRNA	1,73E-02
HDAC7	transcription regulator	1,73E-02
TRIM3	enzyme	1,73E-02
ACKR1	G-protein coupled receptor	1,73E-02

LIPG	enzyme	1,73E-02
TNFRSF13B	transmembrane receptor	1,73E-02
PCDH11Y	other	1,73E-02
tranilast	chemical drug	1,73E-02
mangiferin	chemical - endogenous non-mammalian	1,73E-02
epirubicin	chemical drug	1,73E-02
HBEGF	growth factor	1,74E-02
cilostazol	chemical drug	1,74E-02
CAY10585	chemical reagent	1,76E-02
12(S)-hydroxyeicosatetraenoic acid	chemical - endogenous non-mammalian	1,76E-02
Vhl	complex	1,76E-02
N-Cadherin	group	1,76E-02
meldonium	chemical drug	1,76E-02
rhodamine 6G	chemical toxicant	1,76E-02
CTNS	transporter	1,76E-02
ARID3B	transcription regulator	1,76E-02
GPR84	G-protein coupled receptor	1,76E-02
PPT1	enzyme	1,76E-02
Meg3	other	1,76E-02
SLC39A8	transporter	1,76E-02
HSD17B12	enzyme	1,76E-02
EHD2	other	1,76E-02
SULT2B1	enzyme	1,76E-02
ZNF382	transcription regulator	1,76E-02
IL-2R	complex	1,76E-02
saracatinib	chemical drug	1,76E-02
CD8A	other	1,76E-02
SLC25A4	transporter	1,76E-02
CD82	other	1,76E-02
U1 snRNP	complex	1,76E-02
CD33	other	1,76E-02
TTN	kinase	1,76E-02
mir-190	microRNA	1,76E-02
miR-192-5p (and other miRNAs w/seed UGACCUA)	mature microRNA	1,76E-02
miR-140-5p (and other miRNAs w/seed AGUGGUU)	mature microRNA	1,76E-02
SPN	transmembrane receptor	1,76E-02
Ifi202b	other	1,76E-02
FZD5	G-protein coupled receptor	1,76E-02
ATF7	transcription regulator	1,76E-02
DSG2	other	1,76E-02
CUL7	enzyme	1,76E-02
FKBP1A	enzyme	1,76E-02
MAD2L1	other	1,76E-02
MAD2L2	enzyme	1,76E-02

UACA	other	1,76E-02
BLM	enzyme	1,76E-02
NEU3	enzyme	1,76E-02
PROKR1	G-protein coupled receptor	1,76E-02
RBCK1	transcription regulator	1,76E-02
ONECUT2	transcription regulator	1,76E-02
TNRC6A	other	1,76E-02
RFX4	transcription regulator	1,76E-02
NCR3	transmembrane receptor	1,76E-02
GADD45GIP1	other	1,76E-02
PCBP1	translation regulator	1,76E-02
ULBP1	transmembrane receptor	1,76E-02
Gm20703	other	1,76E-02
AP20187	chemical reagent	1,76E-02
raclopride	chemical drug	1,76E-02
temsirolimus	chemical drug	1,76E-02
GW 5074	chemical drug	1,76E-02
benzoic acid	chemical - endogenous mammalian	1,76E-02
ISRIB	chemical reagent	1,76E-02
rabeprazole	chemical drug	1,76E-02
magnesium sulfate	chemical drug	1,76E-02
PCM1-JAK2	fusion gene/product	1,76E-02
cinnamon powder	chemical reagent	1,76E-02
retinaldehyde	chemical - endogenous mammalian	1,76E-02
polaprezinc	biologic drug	1,76E-02
huperzine A	chemical drug	1,76E-02
vinpocetine	chemical drug	1,76E-02
garcinol	chemical - endogenous non-mammalian	1,78E-02
CD3 group	group	1,80E-02
LMNB1	other	1,80E-02
N-ethyl-N-nitrosourea	chemical toxicant	1,80E-02
RNASEL	enzyme	1,84E-02
RNASE2	enzyme	1,84E-02
resolvin D1	chemical - endogenous mammalian	1,84E-02
SUMO1	enzyme	1,84E-02
UBE2I	enzyme	1,84E-02
SH3TC2	other	1,86E-02
miR-133a-3p (and other miRNAs w/seed UUGGUCC)	mature microRNA	1,86E-02
CHADL	other	1,86E-02
CNR2	G-protein coupled receptor	1,86E-02
MSC	transcription regulator	2,00E-02
MTORC1	complex	2,01E-02
SFRP1	transmembrane receptor	2,01E-02
colchicine	chemical drug	2,01E-02

rifampin	chemical drug	2,03E-02
lactosylceramide	chemical - endogenous mammalian	2,11E-02
prostaglandin A1	chemical - endogenous non-mammalian	2,11E-02
CaMKII	complex	2,11E-02
HCAR1	G-protein coupled receptor	2,11E-02
TRIM2	enzyme	2,11E-02
KDM4C	enzyme	2,11E-02
ZNF148	transcription regulator	2,11E-02
MTM1	phosphatase	2,11E-02
ITGA9	other	2,11E-02
NTN1	growth factor	2,11E-02
SFTPD	other	2,11E-02
MAP3K5	kinase	2,11E-02
CCN3	growth factor	2,11E-02
GLP-1-(7-34)-amide	biologic drug	2,11E-02
mezerein	chemical - endogenous non-mammalian	2,11E-02
STAT	group	2,11E-02
MBTD1	other	2,11E-02
ISLR	other	2,11E-02
MTTP	transporter	2,11E-02
HSF2	transcription regulator	2,11E-02
ethylene glycol tetraacetic acid	chemical reagent	2,11E-02
ezetimibe	chemical drug	2,11E-02
taurocholic acid	chemical - endogenous mammalian	2,19E-02
JINK1/2	group	2,19E-02
RPSA	translation regulator	2,19E-02
BGN	other	2,19E-02
YWHAZ	enzyme	2,19E-02
ETV1	transcription regulator	2,19E-02
RACK1	enzyme	2,19E-02
VAV3	cytokine	2,19E-02
CPE	peptidase	2,19E-02
FAAH	enzyme	2,19E-02
ramipril	chemical drug	2,19E-02
R-WIN 55,212	chemical reagent	2,19E-02
cetuximab	biologic drug	2,19E-02
(Z,E)-5-(4-ethylbenzylidine)-2-thioxothiazolidin-4-one	chemical reagent	2,19E-02
FOXG1	transcription regulator	2,19E-02
CHRNA1	transmembrane receptor	2,19E-02
TAP1	transporter	2,19E-02
EHHADH	enzyme	2,19E-02
miR-374b-5p (and other miRNAs w/seed UAUAAUA)	mature microRNA	2,19E-02
GHRHR	G-protein coupled receptor	2,19E-02
PER2	transcription regulator	2,19E-02

GLUL	enzyme	2,19E-02
CBX8	other	2,19E-02
DIRAS3	enzyme	2,19E-02
CPEB1	translation regulator	2,19E-02
IL18R1	transmembrane receptor	2,19E-02
CCL19	cytokine	2,19E-02
SP2	transcription regulator	2,19E-02
HNRNPD	transcription regulator	2,19E-02
TOPBP1	other	2,19E-02
BTC	growth factor	2,19E-02
Ptgs2os2	other	2,19E-02
RGS1	enzyme	2,19E-02
lith-O-Asp	chemical reagent	2,19E-02
FITC	chemical reagent	2,19E-02
ebselen	chemical drug	2,19E-02
ethylene dimethanesulfonate	chemical reagent	2,19E-02
fevipiprant	chemical drug	2,19E-02
N-carbobenzyloxy-leucine-leucine-norvalinal	chemical - protease inhibitor	2,19E-02
deguelin	chemical drug	2,19E-02
U18666A	chemical reagent	2,19E-02
BSCL2	other	2,21E-02
TRIM28	transcription regulator	2,21E-02
NEUROG1	transcription regulator	2,21E-02
TLR8	transmembrane receptor	2,22E-02
ABCG1	transporter	2,22E-02
KDR	kinase	2,22E-02
LMNA	other	2,24E-02
STEAP3	transporter	2,24E-02
AIP	transcription regulator	2,24E-02
RARG	ligand-dependent nuclear receptor	2,28E-02
HDL-cholesterol	complex	2,41E-02
IMMT	other	2,41E-02
TRH	other	2,41E-02
NF2	other	2,41E-02
TFAP2B	transcription regulator	2,41E-02
CIDEC	other	2,41E-02
ceruletide	biologic drug	2,41E-02
parthenolide	chemical drug	2,41E-02
USF1	transcription regulator	2,42E-02
DNMT3B	enzyme	2,42E-02
capsaicin	chemical drug	2,43E-02
SUPT16H	transcription regulator	2,44E-02
ASCL1	transcription regulator	2,46E-02
EHMT1	transcription regulator	2,47E-02

AICAR	chemical - endogenous mammalian	2,47E-02
mir-19	microRNA	2,50E-02
herbimycin	chemical drug	2,50E-02
quinolinic acid	chemical - endogenous mammalian	2,50E-02
omeprazole	chemical drug	2,50E-02
sphingosylphosphocholine	chemical - endogenous mammalian	2,50E-02
R 59022	chemical drug	2,50E-02
L-cysteine	chemical - endogenous mammalian	2,50E-02
dopamine receptor	group	2,50E-02
bazedoxifene	chemical drug	2,50E-02
C8	complex	2,50E-02
L-type Calcium Channel	complex	2,50E-02
S6K1	group	2,50E-02
bafilomycin A	chemical reagent	2,50E-02
BLVRA	enzyme	2,50E-02
HTR7	G-protein coupled receptor	2,50E-02
lentinan	chemical drug	2,50E-02
SENP7	peptidase	2,50E-02
OLFM4	other	2,50E-02
RASSF6	other	2,50E-02
TRIM29	transcription regulator	2,50E-02
FBXO42	other	2,50E-02
NAALADL2	other	2,50E-02
MARCHF2	enzyme	2,50E-02
salmonella typhimurium lipopolysaccharide	chemical - endogenous non-mammalian	2,50E-02
CTDSP1	phosphatase	2,50E-02
NEK7	kinase	2,50E-02
STK17A	kinase	2,50E-02
S100A10	other	2,50E-02
SPTAN1	other	2,50E-02
TIP60	complex	2,50E-02
idelalisib	chemical drug	2,50E-02
CYT003-QbG10	chemical drug	2,50E-02
GPRC5A	G-protein coupled receptor	2,50E-02
PSMB9	peptidase	2,50E-02
KHSRP	enzyme	2,50E-02
FGF3	growth factor	2,50E-02
PI3	other	2,50E-02
IL22RA2	transmembrane receptor	2,50E-02
DNM3OS	other	2,50E-02
NEDD8	enzyme	2,50E-02
SFRP2	transmembrane receptor	2,50E-02
GUSB	enzyme	2,50E-02
genipin	chemical - endogenous non-mammalian	2,50E-02

IL4I1	enzyme	2,50E-02
ING4	transcription regulator	2,50E-02
PRSS8	peptidase	2,50E-02
PANDAR	other	2,50E-02
HTRA1	peptidase	2,50E-02
NEK6	kinase	2,50E-02
CD276	other	2,50E-02
GPR183	G-protein coupled receptor	2,50E-02
SCUBE3	other	2,50E-02
PPP1CA	phosphatase	2,50E-02
CD99	other	2,50E-02
MPL	transmembrane receptor	2,50E-02
CSNK1A1	kinase	2,50E-02
ANPEP	peptidase	2,50E-02
C7	other	2,50E-02
AP3B1	transporter	2,50E-02
EDA	cytokine	2,50E-02
HA900	chemical reagent	2,50E-02
ivermectin	chemical drug	2,50E-02
cilostamide	chemical reagent	2,50E-02
acrylamide	chemical toxicant	2,50E-02
nigericin	chemical drug	2,50E-02
racemic flurbiprofen	chemical drug	2,50E-02
RP 73401	chemical toxicant	2,50E-02
7,8-dihydro-7,8-dihydroxybenzo(a)pyrene 9,10-oxide	chemical toxicant	2,50E-02
adapalene	chemical drug	2,50E-02
setanaxib	chemical drug	2,50E-02
aripiprazole	chemical drug	2,50E-02
tangeretin	chemical - endogenous non-mammalian	2,50E-02
pentazocine	chemical drug	2,50E-02
tyrphostin AG 127	chemical drug	2,50E-02
SLR14	chemical reagent	2,50E-02
miricorilant	chemical reagent	2,50E-02
SETX	enzyme	2,53E-02
Pde4	group	2,53E-02
CD200	other	2,53E-02
MED12	transcription regulator	2,53E-02
IFNK	cytokine	2,53E-02
MPZ	other	2,53E-02
miR-19b-3p (and other miRNAs w/seed GUGCAAA)	mature microRNA	2,53E-02
miR-218-5p (and other miRNAs w/seed UGUGCUU)	mature microRNA	2,53E-02
SLC2A1	transporter	2,53E-02
LOX	enzyme	2,53E-02
MBD1	transcription regulator	2,53E-02

RFX2	transcription regulator	2,53E-02
SCGB1A1	cytokine	2,53E-02
Sch-23390	chemical drug	2,53E-02
dipyridamole	chemical drug	2,53E-02
pifithrin alpha	chemical reagent	2,53E-02
Zn2+	chemical - endogenous mammalian	2,53E-02
CXCL10	cytokine	2,54E-02
IDR-1002	chemical reagent	2,54E-02
fluvoxamine	chemical drug	2,54E-02
mir-122	microRNA	2,66E-02
SOX7	transcription regulator	2,67E-02
RTN4	other	2,67E-02
NfkB1-RelA	complex	2,74E-02
SIGLEC8	transmembrane receptor	2,74E-02
tempol	chemical drug	2,74E-02
bromocriptine	chemical drug	2,74E-02
cyclic GMP	chemical - endogenous mammalian	2,75E-02
LINC00662	other	2,75E-02
CMKLR1	G-protein coupled receptor	2,75E-02
ATXN2	other	2,75E-02
PURA	transcription regulator	2,75E-02
CBLB	enzyme	2,75E-02
miR-143-3p (and other miRNAs w/seed GAGAUGA)	mature microRNA	2,75E-02
ANGPTL2	other	2,75E-02
TNFRSF25	transmembrane receptor	2,75E-02
TNFAIP2	other	2,75E-02
GPR37	G-protein coupled receptor	2,75E-02
NDN	transcription regulator	2,75E-02
101.10 peptide	chemical reagent	2,75E-02
bosutinib	chemical drug	2,75E-02
cystamine	chemical drug	2,75E-02
erythromycin	chemical drug	2,75E-02
glyburide	chemical drug	2,75E-02
WIN 55,212-2	chemical - other	2,75E-02
morin	chemical - endogenous non-mammalian	2,75E-02
caffeic acid	chemical drug	2,75E-02
reserpine	chemical drug	2,75E-02
palmitoylethanolamide	chemical drug	2,75E-02
rebamipide	chemical drug	2,75E-02
RGS10	enzyme	2,76E-02
tributyltin	chemical reagent	2,79E-02
dorsomorphin	chemical - kinase inhibitor	2,79E-02
EGR3	transcription regulator	2,80E-02
bafilomycin A1	chemical drug	2,80E-02

NR1H4	ligand-dependent nuclear receptor	2,82E-02
mir-29	microRNA	2,87E-02
IL4R	transmembrane receptor	2,87E-02
RUNX1-RUNX1T1	fusion gene/product	2,87E-02
streptozocin	chemical drug	2,88E-02
ursodeoxycholic acid	chemical - endogenous mammalian	2,93E-02
KCNE3	ion channel	2,93E-02
BAK1	other	2,94E-02
PTH1R	G-protein coupled receptor	2,94E-02
TRB	transmembrane receptor	2,94E-02
SMAD5	transcription regulator	2,94E-02
selenium	chemical drug	2,94E-02
fludarabine	chemical drug	2,94E-02
SOX9	transcription regulator	2,97E-02
Ciap	group	3,00E-02
EIF2A	translation regulator	3,00E-02
PADI2	enzyme	3,00E-02
KDM4B	enzyme	3,00E-02
PCSK9	peptidase	3,00E-02
EIF4G1	translation regulator	3,00E-02
FSTL1	other	3,00E-02
ACVR1C	kinase	3,00E-02
TRIM21	enzyme	3,00E-02
vanillin	chemical - endogenous non-mammalian	3,00E-02
chelerythrine	chemical drug	3,00E-02
SB 290157	chemical reagent	3,00E-02
KAT6A	enzyme	3,03E-02
ARV771	chemical reagent	3,04E-02
behenic acid	chemical - endogenous mammalian	3,04E-02
treprostinil	chemical drug	3,04E-02
cloprostenol	chemical drug	3,04E-02
hypoxanthine	chemical - endogenous mammalian	3,04E-02
L-alanine	chemical - endogenous mammalian	3,04E-02
QC6352	chemical reagent	3,04E-02
N4	chemical reagent	3,04E-02
repertaxin	chemical drug	3,04E-02
Rsk	group	3,04E-02
tyrosine kinase	group	3,04E-02
chitinase	group	3,04E-02
TEC/BTK/ITK/TXK/BMX	group	3,04E-02
SNHG1	other	3,04E-02
tozasertib	chemical drug	3,04E-02
tylophorine	chemical drug	3,04E-02
MCU	ion channel	3,04E-02

CACTIN	other	3,04E-02
CCDC88B	enzyme	3,04E-02
LRP4	other	3,04E-02
Vacuolar H+ ATPase	complex	3,04E-02
MARCHF5	enzyme	3,04E-02
UBA5	enzyme	3,04E-02
LY75	transmembrane receptor	3,04E-02
ZXDC	transcription regulator	3,04E-02
ATF6B	transcription regulator	3,04E-02
TNIP3	other	3,04E-02
HECTD3	enzyme	3,04E-02
GNL3L	other	3,04E-02
USE1	other	3,04E-02
GLIPR2	other	3,04E-02
HSPBP1	other	3,04E-02
B3GNT2	enzyme	3,04E-02
SPATA2	other	3,04E-02
ASCC1	transcription regulator	3,04E-02
UFC1	enzyme	3,04E-02
SLC13A5	transporter	3,04E-02
NBR2	other	3,04E-02
TEX11	other	3,04E-02
SLC22A3	transporter	3,04E-02
CDK5RAP3	other	3,04E-02
NDUFA4L2	enzyme	3,04E-02
MTSS1	other	3,04E-02
CUEDC2	other	3,04E-02
RPRD1A	other	3,04E-02
TRIL	other	3,04E-02
DACT3	other	3,04E-02
LRRC19	other	3,04E-02
Sapk	group	3,04E-02
tetrahydrocurcumin	chemical - endogenous mammalian	3,04E-02
asparagine	chemical - endogenous mammalian	3,04E-02
proanthocyanidin derivative	chemical - other	3,04E-02
VLDL	complex	3,04E-02
PDLIM1	transcription regulator	3,04E-02
PX 478	chemical drug	3,04E-02
AZD7762	chemical drug	3,04E-02
nintedanib	chemical drug	3,04E-02
SERPING1	other	3,04E-02
TBR1	transcription regulator	3,04E-02
perfluorooctane sulfonic acid	chemical toxicant	3,04E-02
RAB11FIP3	other	3,04E-02

cerotic acid	chemical - endogenous mammalian	3,04E-02
CTSD	peptidase	3,04E-02
MZB1	other	3,04E-02
CDC25B	phosphatase	3,04E-02
CCL17	cytokine	3,04E-02
POU2F3	transcription regulator	3,04E-02
FGD5-AS1	other	3,04E-02
SPTBN1	other	3,04E-02
CLIP1	other	3,04E-02
miR-381-3p (and other miRNAs w/seed AUACAAG)	mature microRNA	3,04E-02
mir-766	microRNA	3,04E-02
mir-361	microRNA	3,04E-02
mir-32	microRNA	3,04E-02
MIR585	microRNA	3,04E-02
crocin	chemical drug	3,04E-02
CALCR	G-protein coupled receptor	3,04E-02
SGCB	other	3,04E-02
ORM2	other	3,04E-02
PHLDA1	other	3,04E-02
HUNK	kinase	3,04E-02
MEP1A	peptidase	3,04E-02
NASP	other	3,04E-02
lanatoside C	chemical - endogenous non-mammalian	3,04E-02
HSPB6	other	3,04E-02
SLC7A11	transporter	3,04E-02
GJB2	transporter	3,04E-02
LSM1	other	3,04E-02
APOD	transporter	3,04E-02
RGD1560225	other	3,04E-02
OTULIN	peptidase	3,04E-02
APLP2	other	3,04E-02
NLRC3	other	3,04E-02
Rps6ka5	kinase	3,04E-02
S100A2	other	3,04E-02
LSINCT5	other	3,04E-02
SNRK	kinase	3,04E-02
BICD2	other	3,04E-02
SKLB023	chemical reagent	3,04E-02
ELOA	transcription regulator	3,04E-02
AKAP13	other	3,04E-02
chelidonine	chemical - endogenous non-mammalian	3,04E-02
S3I-1757	chemical reagent	3,04E-02
PLK3	kinase	3,04E-02
CSTB	peptidase	3,04E-02

PLCD4	enzyme	3,04E-02
RAN	enzyme	3,04E-02
SCIN	other	3,04E-02
PODXL	kinase	3,04E-02
1-[2,3-bis(furan-2-yl)quinoxalin-6-yl]-3-(4-bromophenyl)urea	chemical reagent	3,04E-02
TBPL1	transcription regulator	3,04E-02
MK-8776	chemical drug	3,04E-02
NBN	other	3,04E-02
NOC2L	transcription regulator	3,04E-02
TXN2	enzyme	3,04E-02
CAY10397	chemical reagent	3,04E-02
N-hydroxy-2,2-diphenylacetamide	chemical reagent	3,04E-02
WTAP	other	3,04E-02
PCCA-DT	other	3,04E-02
GR-MD-02	chemical drug	3,04E-02
farnesyltransferase inhibitor	chemical drug	3,04E-02
3-(3-pyridinyl)-1-(4-pyridinyl)-2-propen-1-one	chemical reagent	3,04E-02
(+)-epicatechin	chemical drug	3,04E-02
DEETGE-CAL-Tat	chemical reagent	3,04E-02
p38 MAP kinase inhibitor	chemical drug	3,04E-02
ITF3056	chemical reagent	3,04E-02
belumosudil	chemical drug	3,04E-02
carboxyamido-triazole	chemical drug	3,04E-02
pyrazole	chemical - endogenous non-mammalian	3,04E-02
CV 6209	chemical reagent	3,04E-02
NSC719239	chemical drug	3,04E-02
ascomycin	chemical reagent	3,04E-02
necrostatin-1s	chemical reagent	3,04E-02
alpha-methylhydrocinnamic acid	chemical - endogenous mammalian	3,04E-02
ibotenic acid	chemical toxicant	3,04E-02
centrinone	chemical drug	3,04E-02
protoporphyrin IX	chemical - endogenous mammalian	3,04E-02
4-methylcatechol	chemical - endogenous mammalian	3,04E-02
ferrous sulfate	chemical drug	3,04E-02
senexin B	chemical drug	3,04E-02
NUP98-HOXD13	fusion gene/product	3,04E-02
CCI-007	chemical reagent	3,04E-02
1-eicosapentaenoylglycerol	chemical reagent	3,04E-02
vapreotide	biologic drug	3,04E-02
miR-499a-5p inhibitor	chemical reagent	3,04E-02
miR-208b inhibitor	chemical reagent	3,04E-02
microcystin-LR	chemical toxicant	3,04E-02
perillic acid	chemical - endogenous non-mammalian	3,04E-02
yohimbine	chemical drug	3,04E-02

erucic acid	chemical - endogenous non-mammalian	3,04E-02
15-hydroxyeicosatetraenoic acid	chemical - endogenous mammalian	3,04E-02
titanium	chemical reagent	3,04E-02
vigabatrin	chemical drug	3,04E-02
IgG-opsonized ovalbumin	chemical reagent	3,04E-02
pubchem compound 16020046	chemical reagent	3,04E-02
Vi capsular polysaccharide	chemical drug	3,04E-02
cholesterol ester	chemical - endogenous mammalian	3,04E-02
karenitecin	chemical drug	3,04E-02
sodium bisulfide	chemical reagent	3,05E-02
KL	enzyme	3,05E-02
Ccl2	cytokine	3,05E-02
telmisartan	chemical drug	3,05E-02
SMO	G-protein coupled receptor	3,08E-02
RCE1	peptidase	3,10E-02
NFE2L1	transcription regulator	3,10E-02
PCGF2	transcription regulator	3,10E-02
SAMMSON	other	3,10E-02
KIN001-043	chemical drug	3,10E-02
NOS3	enzyme	3,12E-02
KAT2B	transcription regulator	3,12E-02
FASN	enzyme	3,21E-02
COLQ	other	3,21E-02
CBFB	transcription regulator	3,33E-02
NR0B2	ligand-dependent nuclear receptor	3,37E-02
prostaglandin J2	chemical - endogenous mammalian	3,37E-02
NCF1	enzyme	3,37E-02
NPPB	other	3,37E-02
PRKD	group	3,37E-02
UPF2	other	3,37E-02
sodium tungstate	chemical drug	3,37E-02
6-mercaptopurine	chemical drug	3,37E-02
lnc-HAND2-2	other	3,37E-02
belinostat	chemical drug	3,37E-02
HOXA11-AS	other	3,37E-02
LINC01139	other	3,37E-02
kremezin	chemical drug	3,37E-02
PANX1	transporter	3,37E-02
ELOVL2	enzyme	3,37E-02
Shc	group	3,37E-02
B4GALT6	enzyme	3,37E-02
LRRC32	other	3,37E-02
DUOXA1	other	3,37E-02
secukinumab	biologic drug	3,37E-02

CAVIN1	transcription regulator	3,37E-02
PPM1D	phosphatase	3,37E-02
LGALS8	other	3,37E-02
HLA-DQB1	other	3,37E-02
SEMA4D	transmembrane receptor	3,37E-02
PDK2	kinase	3,37E-02
MIR320	group	3,37E-02
P2RX4	ion channel	3,37E-02
SETD7	enzyme	3,37E-02
RNF41	enzyme	3,37E-02
ATP1B1	transporter	3,37E-02
CDCP1	other	3,37E-02
IGBP1	phosphatase	3,37E-02
LILRB3	transmembrane receptor	3,37E-02
HOXD9	transcription regulator	3,37E-02
TP53BP2	other	3,37E-02
LAT2	other	3,37E-02
CXCL16	cytokine	3,37E-02
KCNJ11	ion channel	3,37E-02
NFKBIE	transcription regulator	3,37E-02
CTTN	other	3,37E-02
SYNCRIP	other	3,37E-02
GPAT4	enzyme	3,37E-02
ZMYND8	transcription regulator	3,37E-02
ulixertinib	chemical drug	3,37E-02
RECK	other	3,37E-02
PRKCH	kinase	3,37E-02
IKK-2 inhibitor VIII	chemical drug	3,37E-02
INCB054329	chemical drug	3,37E-02
diltiazem	chemical drug	3,37E-02
allyl isothiocyanate	chemical toxicant	3,37E-02
calmidazolium	chemical drug	3,37E-02
NF 449	chemical reagent	3,37E-02
PCI-34051	chemical reagent	3,37E-02
L-lactic acid	chemical - endogenous mammalian	3,37E-02
sodium dodecyl sulfate	chemical drug	3,37E-02
levamisole	chemical drug	3,37E-02
calcium chloride	chemical drug	3,37E-02
tin mesoporphyrin	chemical drug	3,37E-02
mastoparan	chemical toxicant	3,37E-02
alpha-naphthoflavone	chemical reagent	3,37E-02
hexamethoxyflavone	chemical toxicant	3,37E-02
oleoylethanolamide	chemical - endogenous mammalian	3,37E-02
poly(U)RNA	chemical reagent	3,37E-02

C-miR146a	chemical reagent	3,37E-02
N-chlorotaurine	chemical - kinase inhibitor	3,37E-02
desoxycorticosterone	chemical - endogenous mammalian	3,39E-02
(+)-catechin	chemical drug	3,39E-02
Cyclin E	group	3,39E-02
UCN2	other	3,39E-02
Hmgb1	transcription regulator	3,39E-02
ZDHHC7	enzyme	3,39E-02
FZD9	G-protein coupled receptor	3,39E-02
IL13RA2	transmembrane receptor	3,39E-02
KLF9	transcription regulator	3,39E-02
IL-17f dimer	complex	3,39E-02
MIR101	group	3,39E-02
HBP1	transcription regulator	3,39E-02
ITGB6	other	3,39E-02
NBEAL2	other	3,39E-02
SDC1	enzyme	3,39E-02
AQP7	transporter	3,39E-02
BCL10	transcription regulator	3,39E-02
USP1	peptidase	3,39E-02
HDAC8	transcription regulator	3,39E-02
ECM1	transporter	3,39E-02
nimesulide	chemical drug	3,39E-02
polyamines	chemical - other	3,39E-02
zearalenone	chemical toxicant	3,39E-02
DIM-C-pPhOH-3-Cl-5-OCH3	chemical reagent	3,39E-02
leupeptin	chemical - protease inhibitor	3,39E-02
canrenoate potassium	chemical drug	3,39E-02
manganese	chemical - endogenous mammalian	3,39E-02
ONECUT1	transcription regulator	3,43E-02
beta-naphthoflavone	chemical toxicant	3,46E-02
PCGEM1	other	3,49E-02
SLC9A3R1	transporter	3,49E-02
HOXC6	transcription regulator	3,49E-02
paricalcitol	chemical drug	3,49E-02
Pro-inflammatory Cytokine	group	3,50E-02
ZEB2	transcription regulator	3,50E-02
TBX21	transcription regulator	3,50E-02
plumbagin	chemical toxicant	3,53E-02
TASL	enzyme	3,53E-02
Relaxin	group	3,53E-02
TRA	transmembrane receptor	3,53E-02
EXOSC3	enzyme	3,53E-02
UCP2	transporter	3,53E-02

SLPI	other	3,53E-02
mir-7	microRNA	3,53E-02
HSD17B4	enzyme	3,53E-02
MSX1	transcription regulator	3,53E-02
ochratoxin A	chemical toxicant	3,53E-02
PRMT1	enzyme	3,57E-02
chenodeoxycholic acid	chemical - endogenous mammalian	3,59E-02
DSCAML1	other	3,59E-02
LAS1L	other	3,69E-02
APOA1	transporter	3,69E-02
clozapine	chemical drug	3,69E-02
mir-34	microRNA	3,82E-02
OSCAR	other	3,84E-02
GLIS2	transcription regulator	3,84E-02
THBS4	other	3,84E-02
ZIC3	transcription regulator	3,84E-02
DMD	other	3,89E-02
CTCF	transcription regulator	3,90E-02
IFNE	cytokine	3,91E-02
IRAK1	kinase	3,91E-02
lipid	chemical - endogenous mammalian	3,91E-02
KDM5A	transcription regulator	4,03E-02
GHR	transmembrane receptor	4,04E-02
NKX2-1	transcription regulator	4,08E-02
DNMT3A	enzyme	4,09E-02
resolvin E1	chemical - endogenous mammalian	4,11E-02
deoxycorticosterone acetate	chemical drug	4,11E-02
thyroid hormone receptor	group	4,11E-02
BPIFB1	other	4,11E-02
SIRT2	transcription regulator	4,11E-02
MST1	growth factor	4,11E-02
mir-9	microRNA	4,11E-02
miR-103-3p (and other miRNAs w/seed GCAGCAU)	mature microRNA	4,11E-02
mir-103	microRNA	4,11E-02
JARID2	transcription regulator	4,11E-02
IL36G	cytokine	4,11E-02
PSMD10	transcription regulator	4,11E-02
SRD5A1	enzyme	4,11E-02
CXCR2	G-protein coupled receptor	4,11E-02
CLEC4E	other	4,11E-02
8-(4-chlorophenylthio)-guanosine-3', 5'-cyclic monophosphate	chemical reagent	4,11E-02
PI-103	chemical drug	4,11E-02
Sox2ot	other	4,11E-02
ghrelin	biologic drug	4,11E-02

CBS/CBSL	enzyme	4,11E-02
PHB2	transcription regulator	4,11E-02
ARHGDIG	other	4,11E-02
XRCC6	enzyme	4,11E-02
TRAF5	transporter	4,11E-02
mir-515	microRNA	4,11E-02
GSN	other	4,11E-02
EPHX2	enzyme	4,11E-02
ADORA1	G-protein coupled receptor	4,11E-02
ATXN7	other	4,11E-02
SOD3	enzyme	4,11E-02
TNFAIP8	other	4,11E-02
IRX5	transcription regulator	4,11E-02
pimozide	chemical drug	4,11E-02
10-(6'-ubiquinonyl)decyltriphenylphosphonium	chemical drug	4,11E-02
sertraline	chemical drug	4,11E-02
isoliquiritigenin	chemical - endogenous non-mammalian	4,11E-02
pterostilbene	chemical drug	4,11E-02
TGAL copolymer	biologic drug	4,11E-02
iloprost	chemical drug	4,11E-02
MBD3	other	4,17E-02
MEF2A	transcription regulator	4,17E-02
FHL2	transcription regulator	4,23E-02
ziritaxestat	chemical drug	4,23E-02
CHRNA7	transmembrane receptor	4,36E-02
mir-126	microRNA	4,36E-02
HOXC8	transcription regulator	4,36E-02
PBX1	transcription regulator	4,36E-02
MEIS1	transcription regulator	4,36E-02
indole-3-carbinol	chemical drug	4,36E-02
POSTN	other	4,36E-02
HBA1/HBA2	transporter	4,36E-02
FUS-DDIT3	fusion gene/product	4,36E-02
Irp	group	4,39E-02
UDP	chemical - endogenous mammalian	4,39E-02
azathioprine	chemical drug	4,39E-02
nitroglycerin	chemical drug	4,39E-02
teriflunomide	chemical drug	4,39E-02
Stat1 dimer	complex	4,39E-02
tocilizumab	biologic drug	4,39E-02
coal tar	chemical drug	4,39E-02
HOXA-AS2	other	4,39E-02
DLX6-AS1	other	4,39E-02
CEMIP	enzyme	4,39E-02

MGAT1	enzyme	4,39E-02
T 0070907	chemical reagent	4,39E-02
DPY30	other	4,39E-02
MTF1	transcription regulator	4,39E-02
LTB4R2	G-protein coupled receptor	4,39E-02
HDAC11	transcription regulator	4,39E-02
PPP1R15B	phosphatase	4,39E-02
DGCR5	other	4,39E-02
SNHG20	other	4,39E-02
CD80/CD86	group	4,39E-02
Traj18	other	4,39E-02
Gcn5l	group	4,39E-02
G2535	chemical reagent	4,39E-02
TXK	kinase	4,39E-02
CD53	other	4,39E-02
HSP90AA1	enzyme	4,39E-02
NRBP2	kinase	4,39E-02
KRT19	other	4,39E-02
miR-193a-3p (and other miRNAs w/seed ACUGGCC)	mature microRNA	4,39E-02
mir-320	microRNA	4,39E-02
miR-142-3p (and other miRNAs w/seed GUAGUGU)	mature microRNA	4,39E-02
miR-186-5p (miRNAs w/seed AAAGAAU)	mature microRNA	4,39E-02
miR-185-5p (and other miRNAs w/seed GGAGAGA)	mature microRNA	4,39E-02
ADAR	enzyme	4,39E-02
PTCSC3	other	4,39E-02
LIMA1	other	4,39E-02
C6	other	4,39E-02
CAMK2G	kinase	4,39E-02
OSTM1	other	4,39E-02
TNFSF8	cytokine	4,39E-02
MANF	other	4,39E-02
KLF7	transcription regulator	4,39E-02
GIPR	G-protein coupled receptor	4,39E-02
ENTPD1	enzyme	4,39E-02
ELK3	transcription regulator	4,39E-02
JMJD1C	enzyme	4,39E-02
MYZAP	other	4,39E-02
H3C14	other	4,39E-02
Cxcl3	cytokine	4,39E-02
MAP3K11	kinase	4,39E-02
TAS1R3	G-protein coupled receptor	4,39E-02
CYBA	enzyme	4,39E-02
BP-1-102	chemical reagent	4,39E-02
pargyline	chemical drug	4,39E-02

clioquinol	chemical drug	4,39E-02
kukoamine A	chemical - endogenous non-mammalian	4,39E-02
enterolactone	chemical - endogenous mammalian	4,39E-02
rosmarinic acid	chemical - endogenous non-mammalian	4,39E-02
tin protoporphyrin IX	chemical drug	4,39E-02
phospholipid	chemical - endogenous mammalian	4,39E-02
CA074-methyl ester	chemical reagent	4,39E-02
Z-DEVD-FMK	chemical - protease inhibitor	4,39E-02
allopregnanolone	chemical - endogenous mammalian	4,39E-02
trestolone	chemical drug	4,39E-02
maneb	chemical toxicant	4,41E-02
GCG	other	4,50E-02
PITX2	transcription regulator	4,53E-02
mir-183	microRNA	4,66E-02
NOG	growth factor	4,66E-02
GATA6	transcription regulator	4,67E-02
ALDH2	enzyme	4,74E-02
CNGA3	ion channel	4,74E-02
HDAC9	transcription regulator	4,74E-02
cyanocobalamin	chemical - endogenous mammalian	4,74E-02
poly dA-dT	chemical reagent	4,74E-02
11-deoxyprostaglandin E1	chemical reagent	4,81E-02
8-epi-prostaglandin F2alpha	chemical - endogenous mammalian	4,81E-02
L-aspartic acid	chemical - endogenous mammalian	4,81E-02
beta-glucan	chemical drug	4,81E-02
teniposide	chemical drug	4,81E-02
H-151	chemical reagent	4,81E-02
Adenosine Receptor	group	4,81E-02
xylooligosaccharide	chemical - endogenous non-mammalian	4,81E-02
Mst/krs	group	4,81E-02
AOPEP	peptidase	4,81E-02
styrene	chemical toxicant	4,81E-02
ATPase	group	4,81E-02
MKNK	group	4,81E-02
Cpla2	group	4,81E-02
Erm	group	4,81E-02
aurapten	chemical - endogenous non-mammalian	4,81E-02
CYP	group	4,81E-02
RNF187	enzyme	4,81E-02
IL12RB2/IL23R	group	4,81E-02
anthralin	chemical drug	4,81E-02
DACT1	other	4,81E-02
MELK	kinase	4,81E-02
ETHE1	enzyme	4,81E-02

GPR65	G-protein coupled receptor	4,81E-02
SPINDOC	other	4,81E-02
BPI	transporter	4,81E-02
CHD5	enzyme	4,81E-02
INSL5	other	4,81E-02
ZNF524	other	4,81E-02
ZC3H10	other	4,81E-02
PYHIN1	other	4,81E-02
Snhg8	other	4,81E-02
G6PC3	phosphatase	4,81E-02
sGC	complex	4,81E-02
SLU7	enzyme	4,81E-02
MPC1	transporter	4,81E-02
TAS2R14	G-protein coupled receptor	4,81E-02
IgD	complex	4,81E-02
harmol	chemical - endogenous non-mammalian	4,81E-02
benzenesulfonic acid	chemical reagent	4,81E-02
PLA2G7	enzyme	4,81E-02
ALDH3A2	enzyme	4,81E-02
TAF9	transcription regulator	4,81E-02
IgG2b	complex	4,81E-02
azilsartan	chemical drug	4,81E-02
Cathepsin	group	4,81E-02
7S NGF	complex	4,81E-02
Mucin	group	4,81E-02
GIT1	kinase	4,81E-02
AP3D1	other	4,81E-02
Atf	group	4,81E-02
DCB 3503	chemical reagent	4,81E-02
RAP1B	enzyme	4,81E-02
DAPK1	kinase	4,81E-02
STX11	transporter	4,81E-02
FGR	kinase	4,81E-02
GRAP2	other	4,81E-02
ERN2	kinase	4,81E-02
CRK	other	4,81E-02
PUF60	other	4,81E-02
CBX4	transcription regulator	4,81E-02
LRP8	transmembrane receptor	4,81E-02
NUP98	transporter	4,81E-02
UBB	enzyme	4,81E-02
ZBTB46	transcription regulator	4,81E-02
HAO1	enzyme	4,81E-02
NCK2	kinase	4,81E-02

miR-570-3p (miRNAs w/seed GAAAACA)	mature microRNA	4,81E-02
MIR4269	microRNA	4,81E-02
miR-379-5p (and other miRNAs w/seed GGUAGAC)	mature microRNA	4,81E-02
miR-1285-3p (and other miRNAs w/seed CUGGGCA)	mature microRNA	4,81E-02
mir-612	microRNA	4,81E-02
mir-1275	microRNA	4,81E-02
PPP1R13B	phosphatase	4,81E-02
LGMN	peptidase	4,81E-02
HADHA	enzyme	4,81E-02
FGA	other	4,81E-02
FGG	other	4,81E-02
RHOH	enzyme	4,81E-02
UTS2	other	4,81E-02
RBM14	transcription regulator	4,81E-02
PILRB	other	4,81E-02
Orm1 (includes others)	other	4,81E-02
XAF1	other	4,81E-02
WFS1	enzyme	4,81E-02
CXADR	transmembrane receptor	4,81E-02
RAP1GDS1	other	4,81E-02
DLGAP1	other	4,81E-02
DCX	other	4,81E-02
ARLNC1	other	4,81E-02
BRCA2	transcription regulator	4,81E-02
RANBP1	other	4,81E-02
P2RY12	G-protein coupled receptor	4,81E-02
RPS3	enzyme	4,81E-02
NME3	kinase	4,81E-02
GTF2H4	transcription regulator	4,81E-02
CCL22	cytokine	4,81E-02
ADAM8	peptidase	4,81E-02
SH2B2	other	4,81E-02
IL36RN	cytokine	4,81E-02
AQP3	transporter	4,81E-02
SPIN1	other	4,81E-02
ARL5B	enzyme	4,81E-02
GTF3A	transcription regulator	4,81E-02
SLC37A4	transporter	4,81E-02
VCAM1	transmembrane receptor	4,81E-02
ribociclib	chemical drug	4,81E-02
RACGAP1	transporter	4,81E-02
N-lauroyl-L-phenylalanine	chemical reagent	4,81E-02
DDT	enzyme	4,81E-02
PLAA	other	4,81E-02

TG6-10-1	chemical reagent	4,81E-02
Mcpt1	peptidase	4,81E-02
Muc1	transmembrane receptor	4,81E-02
SNHG22	other	4,81E-02
RFXANK	transcription regulator	4,81E-02
GSK-2606414	chemical reagent	4,81E-02
NT157	chemical reagent	4,81E-02
CLDN2	other	4,81E-02
polyinosine-polycytidylic acid/polyethylenimine formulation	chemical reagent	4,81E-02
CMP5	chemical reagent	4,81E-02
H3B-8800	chemical drug	4,81E-02
sulfamethoxazole/trimethoprim	chemical drug	4,81E-02
methylamine	chemical - endogenous mammalian	4,81E-02
N-hydroxy-N'-(4-butyl-2-methylphenyl)formamidine	chemical reagent	4,81E-02
phenylmethylsulfonyl fluoride	chemical - protease inhibitor	4,81E-02
SQ 29548	chemical reagent	4,81E-02
CP 96345	chemical drug	4,81E-02
vinyl carbamate	chemical toxicant	4,81E-02
plevitrexed	chemical drug	4,81E-02
dimaprit	chemical drug	4,81E-02
butylated hydroxytoluene	chemical toxicant	4,81E-02
tranexamic acid	chemical drug	4,81E-02
ethacrynic acid	chemical drug	4,81E-02
etidronic acid	chemical drug	4,81E-02
roflumilast	chemical drug	4,81E-02
paxilline	chemical - endogenous non-mammalian	4,81E-02
opioid	chemical drug	4,81E-02
H-8	chemical toxicant	4,81E-02
ethyl protocatechuate	chemical reagent	4,81E-02
sodium azide	chemical toxicant	4,81E-02
sodium chlorate	chemical reagent	4,81E-02
CAY10595	chemical reagent	4,81E-02
1-docosapentaenoylglycerol	chemical reagent	4,81E-02
FT671	chemical - protease inhibitor	4,81E-02
sappanone A	chemical - endogenous non-mammalian	4,81E-02
geraniol	chemical - endogenous non-mammalian	4,81E-02
ampelopsin	chemical drug	4,81E-02
omacetaxine mepesuccinate	chemical drug	4,81E-02
chlorine	chemical toxicant	4,81E-02
5-hydroxydecanoic acid	chemical - endogenous mammalian	4,81E-02
vidarabine	chemical drug	4,81E-02
pemetrexed	chemical drug	4,81E-02
L-buthionine (SR)-sulfoximine	chemical drug	4,81E-02
L-homocysteine	chemical - endogenous mammalian	4,81E-02

9-(9Z-octadecenoyloxy)-octadecanoic acid	chemical - endogenous mammalian	4,81E-02
marinobufagenin	chemical - endogenous mammalian	4,81E-02
kb-NB 142-70	chemical drug	4,81E-02
L-alpha-hydroxyglutarate	chemical - endogenous mammalian	4,81E-02
SETD2	enzyme	4,84E-02
PTGS1	enzyme	4,84E-02
HBB	transporter	4,84E-02
beta-carotene	chemical - endogenous mammalian	4,84E-02
CHD4	enzyme	4,86E-02
DIO2	enzyme	4,90E-02
Srebp	group	4,91E-02
TMSB4	group	4,91E-02
EGOT	other	4,91E-02
MFN2	enzyme	4,91E-02
GPR132	G-protein coupled receptor	4,91E-02
miR-486-5p (and other miRNAs w/seed CCUGUAC)	mature microRNA	4,91E-02
CSF3R	transmembrane receptor	4,91E-02
ATR	kinase	4,91E-02
GNA11	enzyme	4,91E-02
ANGPTL4	other	4,91E-02
EMD	other	4,91E-02
NRTN	growth factor	4,91E-02
SCP2	transporter	4,91E-02
acetyl-L-carnitine	chemical - endogenous mammalian	4,91E-02
PS-1145	chemical drug	4,91E-02
helenalin	chemical - endogenous non-mammalian	4,91E-02
27-hydroxycholesterol	chemical - endogenous mammalian	4,91E-02
FTO	enzyme	4,91E-02
RCAN1	other	4,91E-02
GSR	enzyme	4,91E-02
SOX10	transcription regulator	4,91E-02
FKBP5	enzyme	4,91E-02
CORT	other	4,91E-02
pyruvaldehyde	chemical - endogenous mammalian	4,91E-02
4-tert-octylphenol	chemical toxicant	4,91E-02
2,4-dinitrofluorobenzene	chemical toxicant	4,91E-02

Table S7. Common canonical pathways expressed by eDCs (Cheng et al) and BMP7-DCs identified by VENNY.
Only significant pathways were used for analysis ($p < 0.05$)

**207 elements included exclusively in "eDC Cheng et al":**	**84 common elements in "eDC Cheng et al" and "BMP7-DC":**	**28 elements included exclusively in "BMP7-DC":**
EIF2 Signaling	Glucocorticoid Receptor Signaling	Phagosome Formation
Kinetochore Metaphase Signaling Pathway	Integrin Signaling	Role of Pattern Recognition Receptors in Recognition of Bacteria and Viruses
Antigen Presentation Pathway	ILK Signaling	Coagulation System
MSP-RON Signaling In Macrophages Pathway	Hepatic Fibrosis Signaling Pathway	FAK Signaling
Signaling by Rho Family GTPases	RAC Signaling	Inhibition of Matrix Metalloproteases
Phagosome Maturation	Ephrin Receptor Signaling	LPS/IL-1 Mediated Inhibition of RXR Function
Molecular Mechanisms of Cancer	Production of Nitric Oxide and Reactive Oxygen Species in Macrophages	Airway Pathology in Chronic Obstructive Pulmonary Disease
Regulation of Actin-based Motility by Rho	HER-2 Signaling in Breast Cancer	G-Protein Coupled Receptor Signaling
Germ Cell-Sertoli Cell Junction Signaling	Neuroinflammation Signaling Pathway	Fatty Acid Activation
Actin Nucleation by ARP-WASP Complex	HMGB1 Signaling	Hepatic Cholestasis
Aryl Hydrocarbon Receptor Signaling	Actin Cytoskeleton Signaling	Extrinsic Prothrombin Activation Pathway
Glioma Invasiveness Signaling	Fcy Receptor-mediated Phagocytosis in Macrophages and Monocytes	Melatonin Degradation III
Sirtuin Signaling Pathway	Coronavirus Pathogenesis Pathway	Chondroitin Sulfate Biosynthesis (Late Stages)
Virus Entry via Endocytic Pathways	Role of Tissue Factor in Cancer	Gustation Pathway
ID1 Signaling Pathway	IL-8 Signaling	γ-linolenate Biosynthesis II (Animals)
Cell Cycle: G2/M DNA Damage Checkpoint Regulation	Tumor Microenvironment Pathway	Mitochondrial L-carnitine Shuttle Pathway
mTOR Signaling	IL-10 Signaling	Type II Diabetes Mellitus Signaling
Th1 and Th2 Activation Pathway	Atherosclerosis Signaling	MSP-RON Signaling Pathway
Th2 Pathway	LXR/RXR Activation	Chondroitin Sulfate Biosynthesis
fMLP Signaling in Neutrophils	MSP-RON Signaling In Cancer Cells Pathway	Dermatan Sulfate Biosynthesis
RHOGDI Signaling	HGF Signaling	Pyrimidine Deoxyribonucleotides De Novo Biosynthesis I
Apoptosis Signaling	Acute Phase Response Signaling	Xenobiotic Metabolism CAR Signaling Pathway
p53 Signaling	Pulmonary Fibrosis Idiopathic Signaling Pathway	Fatty Acid β-oxidation I
NRF2-mediated Oxidative Stress Response	IL-6 Signaling	Cell Cycle Regulation by BTG Family Proteins
Role of PKR in Interferon Induction and Antiviral Response	Xenobiotic Metabolism Signaling	Tetrapyrrole Biosynthesis II
Th1 Pathway	Axonal Guidance Signaling	Breast Cancer Regulation by Stathmin1
Epithelial Adherens Junction Signaling	IL-17A Signaling in Fibroblasts	Phospholipase C Signaling
Hereditary Breast Cancer Signaling	IL-17A Signaling in Gastric Cells	Cellular Effects of Sildenafil (Viagra)
Sumoylation Pathway	Sertoli Cell-Sertoli Cell Junction Signaling	
Clathrin-mediated Endocytosis Signaling	Caveolar-mediated Endocytosis Signaling	
Estrogen Receptor Signaling	ERK/MAPK Signaling	
MYC Mediated Apoptosis Signaling	Granulocyte Adhesion and Diapedesis	
B Cell Development	Agrin Interactions at Neuromuscular Junction	
Natural Killer Cell Signaling	Colorectal Cancer Metastasis Signaling	
Ferroptosis Signaling Pathway	Agranulocyte Adhesion and Diapedesis	
TNFR1 Signaling	Regulation of Cellular Mechanics by Calpain Protease	
Mitotic Roles of Polo-Like Kinase	PI3K/AKT Signaling	
Cholecystokinin/Gastrin-mediated Signaling	IL-12 Signaling and Production in Macrophages	
Remodeling of Epithelial Adherens Junctions	Wound Healing Signaling Pathway	
Semaphorin Signaling in Neurons	Leukocyte Extravasation Signaling	
PPAR Signaling	Hepatic Fibrosis / Hepatic Stellate Cell Activation	
Protein Ubiquitination Pathway	PTEN Signaling	
IL-4 Signaling	Neuregulin Signaling	
Thrombin Signaling	Paxillin Signaling	
Renal Cell Carcinoma Signaling	Osteoarthritis Pathway	
HIF1α Signaling	Coronavirus Replication Pathway	
GADD45 Signaling	VDR/RXR Activation	
PD-1, PD-L1 cancer immunotherapy pathway	Docosahexaenoic Acid (DHA) Signaling	
Senescence Pathway	GM-CSF Signaling	
CXCR4 Signaling	Erythropoietin Signaling Pathway	
LPS-stimulated MAPK Signaling	Role of Macrophages, Fibroblasts and Endothelial Cells in Rheumatoid Arthritis	
Regulation of eIF4 and p70S6K Signaling	Role of IL-17A in Psoriasis	
PAK Signaling	Inhibition of Angiogenesis by TSP1	
Oxidative Phosphorylation	TREM1 Signaling	
Xenobiotic Metabolism AHR Signaling Pathway	PPARα/RXRα Activation	
Synaptogenesis Signaling Pathway	Interferon Signaling	
Reelin Signaling in Neurons	Toll-like Receptor Signaling	
Unfolded protein response	STAT3 Pathway	
RHOA Signaling	Pulmonary Healing Signaling Pathway	
Granzyme B Signaling	Chemokine Signaling	
Acute Myeloid Leukemia Signaling	Semaphorin Neuronal Repulsive Signaling Pathway	

Gαq Signaling	Role of IL-17F in Allergic Inflammatory Airway Diseases	
PI3K Signaling in B Lymphocytes	Pyroptosis Signaling Pathway	
Hypoxia Signaling in the Cardiovascular System	Role of IL-17A in Arthritis	
CSDE1 Signaling Pathway	Differential Regulation of Cytokine Production in Macrophages and T Helper Cells by IL-17A and IL-17F	
Renin-Angiotensin Signaling	Adipogenesis pathway	
Ephrin B Signaling	Airway Inflammation in Asthma	
CD27 Signaling in Lymphocytes	Role of MAPK Signaling in Inhibiting the Pathogenesis of Influenza	
Induction of Apoptosis by HIV1	Iron homeostasis signaling pathway	
Tumoricidal Function of Hepatic Natural Killer Cells	Oxytocin Signaling Pathway	
JAK/STAT Signaling	BEX2 Signaling Pathway	
TWEAK Signaling	Oxytocin In Brain Signaling Pathway	
Crosstalk between Dendritic Cells and Natural Killer Cells	Phenylalanine Degradation IV (Mammalian, via Side Chain)	
Granzyme A Signaling	Cardiac Hypertrophy Signaling (Enhanced)	
GNRH Signaling	Role of Hypercytokinemia/hyperchemokinemia in the Pathogenesis of Influenza	
Regulation Of The Epithelial Mesenchymal Transition By Growth Factors Pathway	Differential Regulation of Cytokine Production in Intestinal Epithelial Cells by IL-17A and IL-17F	
14-3-3-mediated Signaling	Salvage Pathways of Pyrimidine Deoxyribonucleotides	
Huntington's Disease Signaling	Thyroid Cancer Signaling	
PEDF Signaling	IL-17 Signaling	
BAG2 Signaling Pathway	Opioid Signaling Pathway	
IGF-1 Signaling	Heme Degradation	
Death Receptor Signaling	GP6 Signaling Pathway	
Prostate Cancer Signaling	IL-7 Signaling Pathway	
Cardiac Hypertrophy Signaling	Inflammasome pathway	
Cell Cycle Control of Chromosomal Replication		
Ceramide Signaling		
CLEAR Signaling Pathway		
Pancreatic Adenocarcinoma Signaling		
ATM Signaling		
IL-9 Signaling		
Putrescine Degradation III		
RAR Activation		
FAT10 Cancer Signaling Pathway		
Polyamine Regulation in Colon Cancer		
Adrenomedullin signaling pathway		
Angiopoietin Signaling		
SPINK1 General Cancer Pathway		
Mitochondrial Dysfunction		
Glioblastoma Multiforme Signaling		
BMP signaling pathway		
IL-23 Signaling Pathway		
Autophagy		
Telomerase Signaling		
Thrombopoietin Signaling		
TNFR2 Signaling		
iNOS Signaling		
Ephrin A Signaling		
Role of JAK1, JAK2 and TYK2 in Interferon Signaling		
RANK Signaling in Osteoclasts		
ERK5 Signaling		
Antioxidant Action of Vitamin C		
Pathogenesis of Multiple Sclerosis		
p70S6K Signaling		
ERBB Signaling		
GDNF Family Ligand-Receptor Interactions		
Macropinocytosis Signaling		
Role of MAPK Signaling in Promoting the Pathogenesis of Influenza		
P2Y Purigenic Receptor Signaling Pathway		
Prolactin Signaling		
TGF-β Signaling		
NF-κB Activation by Viruses		
UVB-Induced MAPK Signaling		
BER (Base Excision Repair) Pathway		
IL-13 Signaling Pathway		
Amyotrophic Lateral Sclerosis Signaling		
Role of p14/p19ARF in Tumor Suppression		

IL-3 Signaling		
UVA-Induced MAPK Signaling		
Estrogen-Dependent Breast Cancer Signaling		
Sphingosine-1-phosphate Signaling		
NGF Signaling		
Spermidine Biosynthesis I		
ERB2-ERBB3 Signaling		
Xenobiotic Metabolism General Signaling Pathway		
Cyclins and Cell Cycle Regulation		
Gα12/13 Signaling		
Aldosterone Signaling in Epithelial Cells		
Role of CHK Proteins in Cell Cycle Checkpoint Control		
Glioma Signaling		
Relaxin Signaling		
CCR3 Signaling in Eosinophils		
CD40 Signaling		
Gap Junction Signaling		
PDGF Signaling		
Tight Junction Signaling		
Melanoma Signaling		
April Mediated Signaling		
Chronic Myeloid Leukemia Signaling		
UVC-Induced MAPK Signaling		
B Cell Activating Factor Signaling		
Tryptophan Degradation X (Mammalian, via Tryptamine)		
Protein Kinase A Signaling		
Role of BRCA1 in DNA Damage Response		
3-phosphoinositide Degradation		
Apelin Endothelial Signaling Pathway		
HOTAIR Regulatory Pathway		
3-phosphoinositide Biosynthesis		
Histamine Degradation		
Fatty Acid α-oxidation		
Endoplasmic Reticulum Stress Pathway		
Noradrenaline and Adrenaline Degradation		
Serine Biosynthesis		
Mouse Embryonic Stem Cell Pluripotency		
Endocannabinoid Developing Neuron Pathway		
Small Cell Lung Cancer Signaling		
Superpathway of Inositol Phosphate Compounds		
D-myo-inositol (1,4,5,6)-Tetrakisphosphate Biosynthesis		
D-myo-inositol (3,4,5,6)-tetrakisphosphate Biosynthesis		
Antiproliferative Role of Somatostatin Receptor 2		
Vitamin-C Transport		
G Beta Gamma Signaling		
NAD Signaling Pathway		
Neurotrophin/TRK Signaling		
Mismatch Repair in Eukaryotes		
Role of JAK1 and JAK3 in γc Cytokine Signaling		
Dopamine Degradation		
Ethanol Degradation IV		
D-myo-inositol-5-phosphate Metabolism		
Endometrial Cancer Signaling		
Phenylethylamine Degradation I		
Role of Osteoblasts, Osteoclasts and Chondrocytes in Rheumatoid Arthritis		
FLT3 Signaling in Hematopoietic Progenitor Cells		
Role of MAPK Signaling in the Pathogenesis of Influenza		
Oncostatin M Signaling		
4-1BB Signaling in T Lymphocytes		
Ethanol Degradation II		
Androgen Signaling		
Endothelin-1 Signaling		
Insulin Secretion Signaling Pathway		
Estrogen-mediated S-phase Entry		
MIF Regulation of Innate Immunity		

Regulation of the Epithelial-Mesenchymal Transition Pathway		
HIPPO signaling		
Purine Nucleotides De Novo Biosynthesis II		
IL-1 Signaling		
EGF Signaling		
Activation of IRF by Cytosolic Pattern Recognition Receptors		
Fc Epsilon RI Signaling		
DNA damage-induced 14-3-3σ Signaling		
Apelin Pancreas Signaling Pathway		
Superpathway of Serine and Glycine Biosynthesis I		
Tetrahydrofolate Salvage from 5,10-methenyltetrahydrofolate		
dTMP De Novo Biosynthesis		
Circadian Rhythm Signaling		
Glutathione Redox Reactions I		
p38 MAPK Signaling		
IL-17A Signaling in Airway Cells		

Table S8. Common Upstream Regulators expressed by eDCs (Cheng et al) and BMP7-DCs identified by VENNY.
Only significant Upstream Regulators were used for analysis ($p < 0.05$)

2084 elements included exclusively in "eDC Cheng et al":	2215 common elements in "eDC Cheng et al" and "BMP7-DC":	1186 elements included exclusively in "BMP7-DC":
5-fluorouracil	TP53	(S,R)-3-(4-hydroxyphenyl)-4,5-dihydro-5-isoxazole acetic acid, methyl ester
E2F1	TGFB1	lipoxin A4
E2F4	beta-estradiol	FKHR
COPS5	dexamethasone	bis(4-hydroxyphenyl)sulfone
CD 437	lipopolysaccharide	GBP5
methylselenic acid	MYC	UFD1
	TNF	NPLOC4
ST1926	KRAS	brusatol
l-asparaginase	IFNG	RUNX2
Eldr	PD98059	PARP2
LARP1	IgG	SLC4A1
	sirolimus	thermozymocidin
PSEN1	NFKBIA	GPBAR1
torin1	CDKN1A	BET
GPER1	IL2	palmitoleic acid
CCND1	HGF	NR1H2
CDK19	CD3	CD36
CKAP2L	ERBB2	CF-02
TBX2	IL1B	GNAI2
PC-SPES	CDKN2A	bilirubin
diethylstilbestrol	aflatoxin B1	TIFA
AR	ESR1	MAS1
RABL6	HRAS	laquinimod
AREG	APP	prostaglandin A2
26s Proteasome	camptothecin	TO-901317
HNF4A	Immunoglobulin	NKRF
RASSF1	IL4	3-hydroxyanthranilic acid
topotecan	tretinoin	chitin
5-azacytidine	CEBPB	salvin
RICTOR	PDGF BB	IL17F
black raspberry extract	CD40LG	TENM1
GnRH analog	CTNNB1	MHC CLASS I (family)
RRP1B	medroxyprogesterone acetate	CTSK
n-nitrosomethylbenzylamine	doxorubicin	MCC950
BMS-690514	CSF2	I-BOP
acyline	hydrogen peroxide	RLN2
GnRH-A	2-(4-amino-1-isopropyl-1H-pyrazolo[3,4-d]pyrimidin-3-yl)-1H-indol-5-ol	MAFK
trans-hydroxytamoxifen	TCR	PRC2
panobinostat	TP73	STK40
L-methionine	LY294002	N-omega-propyl-L-arginine
miR-124-3p (and other miRNAs w/seed AAGGCAC)	ESR2	Rab11
MAX	calcitriol	SR1555
GABA	FOXO3	SR2211
IPMK	Interferon alpha	polyinosinic acid
CST5	dihydrotestosterone	dabrafenib
FBXW7	GLI1	CD5L
FUS	EGF	bupivacaine
uranyl nitrate	MAPT	SU1498
glutamine	tetradecanoylphorbol acetate	minocycline
estrogen	fulvestrant	P2RY6
NFYA	IL3	CCNC
discodermolide	MYCN	dehydroisoandrosterone
COL18A1	butyric acid	FOXA2
RBL2	OSM	docosapentaenoic acid
diclofenac	filgrastim	GRB10
calcimycin	TP63	theophylline
PPP1R13L	8-bromo-cAMP	DHX36
LONP1	PRL	buthionine sulfoximine
ibrutinib	PGR	CCR3
bicuculline	Vegf	PTGIR
PLK1	STAT3	DGKZ

ADRA1D	RB1	ISG15
S100A6	JUN	OXTR
Rb	YAP1	3-methylcholanthrene
NFYB	YY1	Focal adhesion kinase
ARVib-7	methylprednisolone	CBX5
ARVib-31	forskolin	FOXC2
telapristone acetate	FOS	CARD9
raltitrexed	progesterone	DEFA1 (includes others)
PTPRJ	U0126	ABRAXAS2
ASPSCR1-TFE3	EGFR	TMEM208
NORAD	imatinib	RRAD
oxaliplatin	IL13	MAT2B
HNF1A-AS1	IL33	PES1
CD24	trichostatin A	ONC-201
methyl methanesulfonate	arsenic trioxide	fomepizole
ATF6	KLF6	triphenyltin
EIF2AK3	IL10	fosfomycin
ammonium	cisplatin	minodronate
BCOR	NR3C1	aluminum
RBL1	cyclosporin A	PPIF
CDK1	PTEN	octanoic acid
O6-benzylguanine	CG	VASP
dalfampridine	IL6	TRAF4
glutamyl-Se-methylselenocysteine	NFkB (complex)	cicaprost
LIN9	cycloheximide	IHH
desmopressin	AGN194204	WBP2
ethionine	IL15	BV6
TIMP3	TNFSF11	Betacatenin/TCF
seocalcitol	APOE	gamma-tocotrienol
CDK4	AKT1	albuterol
stearic acid	TCF3	miR-182-5p (and other miRNAs w/seed UUGGCAA)
ST3-Hel2A-2	SB203580	ACE2
MXI1	CREB1	C1q
ZFTA-RELA	poly rI:rC-RNA	TNFRSF12A
clopidogrel	CASR	BAY 61-3606
MFAP5	Ige	mevalonic acid
SMC3	TCF4	EFNB2
Cdc42	IKBKB	sofalcone
1,2-dithiol-3-thione	SMARCA4	firtecan pegol
KDM3B	paclitaxel	ARHGEF2
TAF6	RAF1	LRRC8C
HLX	CD44	FLLL-32
sulindac sulfide	D-glucose	AC083837.1
MKNK1	4-hydroxytamoxifen	TET2
TSC1	fluticasone propionate	BAR501
E2f	prostaglandin E2	HRH3
EFNA1	dextran sulfate	pentoxifylline
MAP3K8	BCR (complex)	N-(1-carbamoyl-2-phenyl-ethyl) butyramide
epothilone B	HIF1A	oridonin
anacardic acid	SPI1	metyrapone
spermidine	ERK1/2	mir-96
miR-16-5p (and other miRNAs w/seed AGCAGCA)	LDL	kawain
bromobenzene	Lh	Ferritin
pembrolizumab	CD28	STAP2
CDK2	E2F3	S1PR1
CHEK1	thapsigargin	Integrinα
PCLAF	FOXM1	PORCN
PIK3CA	decitabine	CACNA2D1
PXDN	F2	RBBP8
T-5224	mir-21	Wfdc17
BAG1	SLC15A4	pubchem compound 11483544
MLXIPL	nitrofurantoin	halofuginol
TFEB	IGF1	DMP1
cadmium chloride	etoposide	mir-182
cis-urocanic acid	P38 MAPK	IFNAR2

farnesol	FOXO1	ADORA3
FADD	VEGFA	HMGA2
everolimus	DYSF	RAG1
APEX1	ERK	NTS
Z36	mifepristone	SPHK2
trovafloxacin	FAS	ODC1
KLF3	HSF1	AXIN1
BTNL2	EPO	GABPB1
E2F2	IL27	TLR6
KLK4	BCL6	arecoline
CYB561A3	MAPK1	ACKR2
APOC1	PI3K (complex)	mir-33
L2HGDH	NFKB1	Tnfsf9
PRKAA	TFRC	DL-fructose
BIRC5	tetrachlorodibenzodioxin	Iberiotoxin
2-amino-5-phosphonovaleric acid	gefitinib	thioinosine
imipramine blue	Z-LLL-CHO	KCNK2
amino acids	RELA	PLA2G4E
caffeine	NUPR1	Fendrr
CD5	genistein	LDL-cholesterol
EFNA2	KAT2A	2-chlorohexadecanoic acid
CARM1	lactacystin	NEFL
EFNA5	CSF1	TPR
miR-483-3p (miRNAs w/seed CACUCCU)	CHUK	TRPC3
CHRM3	LDLR	SH2B3
TRAF7	FSH	PSMD4
4-nitroquinoline-1-oxide	GRN	Hrg
miR-125b-5p (and other miRNAs w/seed CCCUGAG)	CD40	U 50488H
AZ-1	VDR	deferiprone
miR-24-3p (and other miRNAs w/seed GGCUCAG)	troglitazone	lestaurtinib
ZBTB7A	EIF4E	18-alpha-glycyrrhetinic acid
FHIT	metribolone	13-hydroxyoctadecadienoic acid
benzene	BRCA1	KLF1
roscovitine	AHR	FFAR3
DRAP1	INSR	vasoactive intestinal peptide
eflornithine	TAZ	RBP4
NSUN3	TAS4464	LGALS9
EZR	Salmonella enterica serotype abortus equi lipopolysaccharide	paroxetine
mir-214	IGF1R	Ni2+
BCYRN1	MTOR	DUX4
mitoxantrone	NRG1	MAFG
PKNOX2	bee venom	COMMD1
BMI1	resiquimod	creatine
RBM20	let-7	NR4A3
LEPR	tanespimycin	cAMP-Gef
FOLR1	vorinostat	FIRRE
EWSR1	NR1H3	QRFP
MASTL	MAP2K1/2	TCIM
H2AX	Insulin	SELL
PIK3CD	leukotriene D4	ULK1
EFNA4	bexarotene	TSC22D1
ABL1	NRAS	fangchinoline
GAS2L3	IL1A	CLIC4
mevastatin	palbociclib	CAPNS1
ALKBH5	ANGPT2	cepharanthine
nilotinib	IL5	MFAP2
GH1	gentamicin	diphenyliodonium
SIN3B	ionomycin	cyclosporin
idarubicin	inosine	nicotinamide-beta-riboside
MDL 73811	mitomycin C	MOG
arotinoid acid	actinomycin D	APLN
EFNA3	curcumin	CTBP2
CLOCK	ID2	P2RY2
promegestone	cardiotoxin	FGF9
RPS15	romidepsin	ZIC1

FBXO32	KDM5B	NODAL
bendamustine	CALCA	garcinia yunnanensis extract YTE-17
SMAD1/5	nitric oxide	cyanidin 3-O-glucoside
miR-183-5p (miRNAs w/seed AUGGCAC)	epigallocatechin-gallate	ANKS6
MED15	FGF2	HERC2
SP3	EGR1	ARHGEF1
pCPT-cAMP	HOXA9	CXCR1
EIF2AK4	CCL5	LY2109761
5-fluoro-2-hydroxycinnamaldehyde	vancomycin	APCS
5-fluoro-2-benzoyloxycinnamaldehyde	MITF	LPA
SERCA	bortezomib	WNT7B
vanillyl-N-nonylamide	ELAVL1	CCX771
EEF2K	PRKCD	CCDC80
6,7-dinitroquinoxaline-2,3-dione	CEBPA	filgotinib
Pln	STAT1	amoxicillin
SIN3A	KDM1A	vandetanib
PLN	SP600125	palmitoyl-Cys((RS)-2,3-di(palmitoyloxy)-propyl)-Ala-Gly-OH
CDK4/6	AGT	sulfo-N-succinimidyl oleate
DOT1L	PPARA	ROCK1
E2F8	GNAQ	pexidartinib
CHRM1	triamcinolone acetonide	pravastatin
TLE1	wortmannin	DDIT4
DAP3	VHL	FABP4
RPL22	IRF4	IL1RL1
F2RL1	Igm	ML-193
GPX1	WT1	Mir218
FLT3	OGA	MEDAG
NME1	MAP2K1	ZBED2
ouabain	budesonide	TMEM79
trichloroethylene	ETV6-RUNX1	ABCD1
EBI3	MET	HOXA2
NSUN6	H2AZ1	S100a7a
CAMK4	TERT	BRD1
12-(3-adamantan-1-yl-ureido) dodecanoic acid	Akt	mir-338
NANOG	NGF	SIX3
miR-1-3p (and other miRNAs w/seed GGAAUGU)	MYOD1	poloxamer
ABCB4	IRAK4	ammonium trichloro(dioxoethylene-O,O'-)tellurate
EIF4EBP1	salmonella minnesota R595 lipopolysaccharides	5-iodotubercidin
ERN1	PAX3-FOXO1	alpha-methylparatyrosine
halofuginone	TFAP2A	BMPR2
THPO	HTT	CXCR3
CRBN	deferoxamine	STS
benzyl isothiocyanate	rosiglitazone	PRKG1
Pkg	tacrolimus	Fgf
4-O-carboxymethylascochlorin	concanavalin a	doramapimod
CETP	PTGER2	Glucocorticoid-GCR
mir-148	SP1	NAP1L1
TAF4	SIRT1	NQO2
MXD1	PPARG	rhesus theta-defensin 1
GSKJ4	NFE2L2	FFAR2
SIRT3	MHC II	THBD
cytochalasin B	BDNF	mir-217
Igkv1-117	DUSP1	thiostrepton
acetic acid	CSF3	NCF4
MAP3K12	GNB1	MLH1
pevonedistat	HAVCR1	pubchem compound 11368987
GABARAPL2	IFNA2	IC87114
MED1	RNA polymerase II	(5-(4-N-methyl-N(2-pyridyl)amino)ethoxy)benzyl thiazolidine-2,4-dione
RNASEH2A	IFI16	TAPI-1
NPSR1	EPHA2	AI-1
CHFR	TLR4	22(S)-hydroxycholesterol
NOTCH3	Jnk	NRIP1
STUB1	Ap1	ALOX5
TINCR	Tgf beta	APOB
NTRK1	ADRB	PHB

SOCS2	HDAC1	PRLR
cannabidiol	IL17A	SARM1
NUMB	Mek	IL17RD
DPP-23	tributyrin	PLA2G1B
phorbol esters	TSC2	miR-208a-3p (and other miRNAs w/seed UAAGACG)
miR-291a-3p (and other miRNAs w/seed AAGUGCU)	EIF2AK2	salinomycin
CIITA	TNFSF10	WNT5B
ATF1	raloxifene	COPA
chloropromazine	pirinixic acid	TNNI3
GABARAPL1	ATF3	SLC9A1
EIF2B5	PTP4A1	Flg
MYL2	BTK	WNT10B
PCM1	GNA15	Ncoa6
RBX1	SMARCB1	emactuzumab
CKS1B	TGFA	montelukast
4-coumaric acid	FOXP3	SS18-SSX2
2-mercaptoethanol	CREM	lamivudine
miR-199a-5p (and other miRNAs w/seed CCAGUGU)	mibolerone	Cu2+
TWIST2	IL1	BCL11B
miR-21-5p (and other miRNAs w/seed AGCUUAU)	cuprizone	oleanolic acid
mir-24	SNAI1	mir-181
TRRAP	valproic acid	ELF3
geranylgeranylacetone	REL	IL20
carfilzomib	NPM1	Brd4
E2F5	BHLHE40	ANTXR2
levodopa	tamoxifen	highly active antiretroviral therapy
D-galactosamine	PRKCE	STAR
COP1	mycophenolic acid	TYRO3
AURKB	TNFSF13B	BCL11A
GABARAP	IGF2	MED14
TEAD1	ID3	HLA-G
LIN28A	5-N-ethylcarboxamido adenosine	CEBPG
HUWE1	ATM	HSPB8
ganetespib	EDN1	ARF6
DAB2IP	carbon tetrachloride	C9
AFP	quercetin	CHGA
SFTPA1	nicotine	WRN
UQCC3	kainic acid	telaglenastat
SHC1	2-bromoethylamine	KU-60019
gemfibrozil	indomethacin	clotrimazole
tert-butyl-hydroquinone	N-acetyl-L-cysteine	roflumilast N-oxide
TRPS1	INS	JMF3086
H2AB3 (includes others)	bisindolylmaleimide I	cholic acid
LRP1	KITLG	I-BET-151
CAB39L	TWIST1	pimagedine
RASSF5	tunicamycin	azithromycin
SN-38	ERG	particulate matter
ROR1	okadaic acid	ERFE
SRSF1	FLCN	CTSG
SBDS	ZBTB16	HPGDS
Ngf	JUNB	CCL21
ALKBH1	SMAD7	IFNL4
KLF17	PGF	VEGFD
H1-2	TGFBR2	PTGER1
MAPK10	IL21	HOXA1
asciminib	ERBB3	KPNA2
RHOB	GNB2	trans-stilbene oxide
L-685,458	tyrphostin AG490	abscisic acid
DT-061	palmitic acid	Complement
SMARCE1	E. coli B5 lipopolysaccharide	nordihydroguaiaretic acid
A2M	simvastatin	poly-L-lysine
UCP1	ILF3	FGF7
ZAP70	SNCA	ARNTL
ITCH	MAPK7	CL 316243
FGFR3	H89	plerixafor

NFAT (complex)	vinorelbine	MSX2
C1QA	RARA	CEACAM1
SREBF2	RC3H1	4-nonylphenol
GCS-100	miR-17-5p (and other miRNAs w/seed AAAGUGC)	SCAVENGER receptor CLASS A
FANCA	Tcf7	TYR
NFE2L3	GAST	RPL13A
MTBP	BCL3	MAFF
FEM1A	IRF8	IQGAP2
EIF2AK1	ACOX1	BGLAP
PPM1B	POU5F1	NR1D2
DIABLO	TLR3	PLAGL2
RHOC	allopurinol	ST14
COTI-2	RGS2	FPR2
aplidine	CDH1	WNT3
L-histidine	EML4-ALK	N-(3-oxododecanoyl)-homoserine lactone
NELFB	NKX2-3	JTE-013
CCAT1	NR3C2	gallic acid
ERRFI1	STAT6	4-octyl itaconate
NUMBL	tosedostat	diacylglycerol
cucurbitacin B	SP110	5,8,11,14-eicosatetraynoic acid
phenethyl isothiocyanate	sorafenib	loxoribine
EIF4G2	MYBL2	auranofin
IND S1	NOTCH1	CYP27B1
RPS6KA5	ADCYAP1	clarithromycin
edaravone	NFATC2	CDKN2B-AS1
BAPTA	prexasertib	FFAR4
SAMSN1	PTGS2	IFNL2
tetraethylammonium	Creb	PTGDR2
NFATC3	Histone h3	FNDC5
doxifluridine	potassium chloride	PIAS4
Foxo	cerivastatin	VEGFC
E2F7	Irgm1	KIF1B
FANCD2	puromycin aminonucleoside	PDE5A
actinonin	ARNT	zinc protoporphyrin IX
TFE3	ATF4	INPP5D
vincristine	5-O-mycolyl-beta-araf-(1->2)-5-O-mycolyl-alpha-araf-(1->1')-glycerol	ZNF281
carbamazepine	interferon beta-1a	ricinoleic acid
PLA2R1	peptidoglycan	acetoacetyl-coenzyme A
IND S7	F7	(5Z,13E)-11alpha-hydroxy-9,15-dioxoprost-13-enoic acid
CDX2	QKI	Keratin 16
DOCK8	PPARGC1A	GSK583
PDGFB	PELP1	PxIL2P
ANXA7	semaxinib	S. pneumoniae glucosyl-diacylglycerol
TYMS	triamterene	LM-1685
ARHGEF25	mir-15	GPSM3
floxuridine	hyaluronic acid	cyanuric acid
mir-25	testosterone	hydroxamic acid
DACH1	ACTL6A	LTB4R/LTB4R2
UCHL1	CNTF	NDPK
HDAC2	FN1	MKK3/6
DCN	hydrocortisone	IRAK1/4
Fus	KLF4	olesoxime
HMGXB4	bleomycin	acridine half-mustard
FAM3B	fenamic acid	Spag11a
IL2RA	estrogen receptor	NF279
TWNK	phytohemagglutinin	USP20
mir-133	cyclophosphamide	NCK
CDC42	TAL1	3-(1-(3-imidazol-1-ylpropyl)-1H-indol-3-yl)-4-anilino-1H-pyrrole-2,5-dione
CNR1	PIK3R1	CPTP
MEL S3	FOSL1	CARD17
TLE3	AMPK	SSC5D
OSMR	SRC	EMC2
CSF2RA	eicosapentenoic acid	Il1bos
EP400	NTRK2	FBXL17
carbamylcholine	resveratrol	UROS

CSF2RB	TCL1A	ZNF496
LYL1	niacinamide	PELI3
CD70	lomustine	FOXN2
indirubin	fluoride	BRIP1
nimodipine	FOXC1	CMPK2
IL24	PLG	KCNJ12
TFAP4	PML	CALCOCO1
MTA1	MDM2	PPIP5K1
PSEN2	arsenite	URGCP
DDX5	staurosporine	CYBC1
miR-92a-3p (and other miRNAs w/seed AUUGCAC)	PAX3	PRG3
cytidylyl-3'-5'-guanosine	Growth hormone	FNDC3B
tryptase	TGM2	FCN1
HINFP	Pkc(s)	DAGLA
NELFA	docosahexaenoic acid	canakinumab
PTMA	PAF1	PSTPIP2
RTKN	Hdac	CLDN9
TKT	BNIP3L	ZNF580
NELFE	bucladesine	GSDMD
GNRH2	MMP9	CCDC62
asbestos	NLRP3	RNF207
1,1-bis(3'-indolyl)-1-(4-trifluoromethyl-phenyl)methane	KLF5	NLRP7
BRAF	entinostat	CCDC47
UBA1	STAT5A	MAPKBP1
HFE	lovastatin	ZG16B
LLGL2	SREBF1	CLIC1
miR-7a-5p (and other miRNAs w/seed GGAAGAC)	TREM1	CAMSAP2
arsenic	MAPK9	EVX2
IDH2	IRF1	EMC6
NFYC	PTH	PTGR2
NLRX1	cytokine	PLAC1
BCL2L1	mir-8	SMG6
GSTO1	IL12 (complex)	rimantadine
CUX1	dopamine	fostamatinib
POU2F2	RUNX1	Integrin alpha 5 beta 1
SERPINE1	IKBKG	Mapk kinase
PTTG1	15-deoxy-delta-12,14 -PGJ 2	pidilizumab
PIM1	XBP1	Tenascin
glucagon	1-methyl-4-phenyl-1,2,3,6-tetrahydropyridine	KSR2
prostaglandin E1	SOCS1	ADCY2
PREX1	CCN1	PRPF4
volasertib	NS-398	mir-129
mir-203	fluocinolone acetonide	miR-331-5p (and other miRNAs w/seed UAGGUAU)
SLC29A1	DDIT3	miR-125a-3p (miRNAs w/seed CAGGUGA)
N4BP1	RET	miR-105-5p (and other miRNAs w/seed CAAAUGC)
peroxynitrite	ECSIT	AQUAPORIN
FGFR1	STK11	ANXA4
phosphate	Pdgf (complex)	TOB2
Ck2 alpha	diethylnitrosamine	CUBN
mocetinostat	TMPRSS2-ERG	TNPO2
Collagen Alpha1	ADRA1A	ETF1
PEMT	miR-155-5p (miRNAs w/seed UAAUGCU)	TYMP
selenite	thioacetamide	GTPBP4
growth factor	MYD88	TONSL
PDK1	MAP2K4	BANF1
caspase	SPP1	PTPRU
Ubiquitin	VCAN	ZBTB1
SLC22A5	gentamicin C	Uox
VAV2	caffeic acid phenethyl ester	CLC
miR-145-5p (and other miRNAs w/seed UCCAGUU)	CLDN7	Cbr2
ARNT2	NPC1	HK1
STIM1	SELP	PMS2
eprenetapopt	ADGRE2	ZNF184
formaldehyde	Tnf (family)	WDR83OS
GLI3	TPH1	CARD16

Collagen(s)	SMAD3	CARD18
MAZ	TFAP2C	16:0 ceramide-1-phosphate
8-hydroxyguanine	CXCL12	CDK20
etretinate	IL9	CD164
AQP11	anisomycin	ETS1-AS1
NEU4	prednisolone	F12
obinutuzumab	entolimod	PTOV1
ENT	sulindac	quercetin-3-methyl ether
MAT2A	TREX1	quercetin-3,4'-dimethyl ether
F2RL3	EP300	tetramethylpyrazine
RAB7A	Pam3-Cys-Ser-Lys4	EVL
POLRMT	Ifn gamma	Nlrp1a
SERPIND1	RAS	minozac free base
MTUS1	Ca2+	ZNF140
taselisib	JQ1	Rhodobacter sphaeroides lipopolysaccharide
LGH447	mir-1	tat-psi-delta-RACK
azetidine	deoxycholate	RPLP0
6-cyano-7-nitroquinoxaline-2,3-dione	BCR-ABL1	nardostachys chinensis extract
PK11007	TCF	CWP232228
UDP-D-glucose	S-nitroso-N-acetyl-DL-penicillamine	3,4-dideoxyglucosone-3-ene
CCNE1	dactolisib	erteberel
MLN8054	IL7	lipophosphonoglycan
2,5-dimethylcelecoxib	PLX5622	beractant
INTS11	homocysteine	dexamethasone/tobramycin
USP12	PP2/AG1879 tyrosine kinase inhibitor	mitoguazone
ELL	Histone h4	pustulan
MAPK15	MRTFB	sulfaphenazole
apilimod	SOD1	L-threo-safingol
DLST	MAPK3	cicletanine
mir-297	diphenyleneiodonium	Ro 20-1724
proTAME	LYN	TAK-779
2-amino-3-phosphonopropionic acid	glucocorticoid	PD173955
DUSP5	SHH	GSK805
WWTR1-CAMTA1	lysophosphatidic acid	aspergillus beta-glucan
NCD-38	IL11RA	pneumocystis beta-glucan
(1S,2R)-NCL-1	ZFP36	flunitrazepam
TEAD	STAT5B	pantoprazole
TRPM8	VCP	tolrestat
mir-506	benzo(a)pyrene	anthraquinone
NCK1	cigarette smoke	MUC2
alpha-santalol	G protein alphai	benzo(a)pyrene-3,6-quinone
ATF5	IL22	phorone
2-hydroxy-1-naphthylaldehyde isonicotinoyl hydrazone	trabectedin	repaglinide
gossypol	methylnitrosourea	anti-miR-302d
aristolochic acid I	E. coli B4 lipopolysaccharide	anti-miR-302c inhibitor
SGPP2	SRF	beta-penta-O-galloyl-glucose
SKP2	WNT3A	potassium iodide
TGS1	PRDM1	THZ531
RAD21	methotrexate	(E)-2-(2-quinolin-2-yl-vinyl)-benzene-1,4-diol
CLCN5	ciprofloxacin	leucyl-leucine-methyl ester
PACS1	IL32	polyphosphate 65
PNN	CD38	luteolin 7-O-beta-D-glucoside
N'-((4-oxo-4H-chromen-3-yl)methylene)nicotinohydrazide	IRF3	isoflavonoid
CD209	aspirin	ferricyanide
PIKFYVE	spermine	cardiolipin
OGG1	temozolomide	17S-hydroperoxy-docosahexaenoic acid
ST6GAL1	ELK1	UMP
GTF2I	GATA1	cladribine
RFX5	ELOVL3	poractant alfa
lauric acid	SNAI2	13-hydroxydocosahexaenoic acid
MYCL	IRS1	lactulose
MMP1	RAC2	V2+
4-hydroxynonenal	napabucasin	NSC 228155
MK2206	PTPN6	glycerol
JUN/JUNB/JUND	PTX3	THRAP3

DCAF1	2-deoxyglucose	SHARPIN
NLRC5	KLF2	ATG16L1
butaprost	EPAS1	IGHE
FGF10	aldesleukin	PRDX2
iron	TRAF2	IQGAP1
BMP6	bromodeoxyuridine	2-acetylaminofluorene
ascorbic acid	nitroprusside	sodium chloride
TOB1	MAPKAPK2	ACVR1
SASH1	ethanol	cyclopamine
HSPA9	6-hydroxydopamine	OLR1
NCOA3	CpG oligonucleotide	NR1I2
VIPR1	acetaminophen	3-deoxy-2-octulosonic acid(2)-lipid A
MEL T1	IFN Beta	sitagliptin
MS4A1	GNA14	ALDH1A1
cyclopiazonic acid	PLAU	TNFRSF6B
N1,N11-diethylnorspermine	SOX2	mir-205
8,9-epoxyeicosatrienoic acid	Fc gamma receptor	IFIT2
EPHB1	PRKAG3	allyl sulfide
Fcor	PRKAA1	indole
mir-224	S100A8	HOXA3
APOH	IFNB1	LRP5
LGALS7/LGALS7B	Pka	alpha-amanitin
NPAT	infliximab	Pdgf Ab
LRPPRC	imiquimod	CCN4
2-[[9-isopropyl-6-[[4-(2-pyridyl)phenyl]methylamino]purin-2-yl]amino]butan-1-ol	BAX	PFKFB3
2,2-bis(4-hydroxyphenyl)-1,1,1-trichloroethane	RIPK1	miR-100-5p (and other miRNAs w/seed ACCCGUA)
FSHR	phenylbutazone	RAB2A
carbonyl cyanide m-chlorophenyl hydrazone	rottlerin	DNM1L
dibutyl phthalate	BRD4	FABP5
miR-203a-3p (and other miRNAs w/seed UGAAAUG)	HOXA10	PLA2G2A
PRKAR2B	NAE1	C5AR2
SL 327	IKZF1	INT-767
phorbol 12,13-didecanoate	triptolide	DMH1
ATP5IF1	GFI1	cerulenin
miR-9-5p (and other miRNAs w/seed CUUUGGU)	STAT4	propionic acid
MAT1A	fenofibrate	IFN type 1
LCP2	SATB1	pregnenolone carbonitrile
GADD45A	EWSR1-FLI1	Gli
lapatinib	CP-55940	UNC93B1
wogonin	KRT14	TFEC
SP4	PAK2	ITGB4
INHA	TNFRSF8	PAEP
Hsp90	PPARD	KLF13
STAG2	oblimersen	NSC23766
SAHM1	fluoromethyl 2,2-difluoro-1-(trifluoromethyl)vinyl ether	monensin
S-adenosylmethionine	TAB1	ethosuximide
flavokawain B	mono-(2-ethylhexyl)phthalate	spironolactone
nicotinic acetylcholine receptor	Map3k7	cyclooxygenase
AKIRIN2	Sb202190	ssRNA40
MT-TE	TCF7L2	SLC2A11
ASCL2	PRNP	SLC2A10
AZD4547	ELANE	TFAM
PDE6B	MAPK14	CCND3
HMGCR	FASLG	TLR1
CCNA2	zVAD-FMK	batimastat
miR-511-5p (miRNAs w/seed UGUCUUU)	PDX1	pirfenidone
MYCBP	mir-223	ADM
CTSE	PD184352	FOSL2
TNFRSF14	HMGA1	KLF15
FUBP1	E. coli lipopolysaccharide	DUSP11
CD226	GAPDH	Stat5 dimer
PRN694	lenalidomide	WLS
Immunoglobulin Lambda Light Chain	PIN1	LGR5
gliotoxin	F2R	FGD3
Muscarinic cholinergic receptor	PRKCA	stattic

KDM8	Raf	Collagen type IV
LCK	trans-cinnamaldehyde	PARG
methylnitronitrosoguanidine	androgen	G6PD
epoxomicin	IL1RN	COL2A1
PKD1	baicalein	PER1
SPRY2	Fcer1	MGLL
SRSF2	TLR2	tamibarotene
14,15-epoxyeicosatrienoic acid	ZFP91	L-tryptophan
Integrin	CX3CL1	PARPBP
PPP1R1B	hexachlorobenzene	FUT8
CSDE1	ITGB2	PRRX1
ABCC8	SB 216763	RIPK3
amlodipine	NFKB2	GNA13
asoprisnil	atorvastatin	TNFSF9
SYK/ZAP	sodium arsenite	ASIP
PHF6	LEP	TERF2IP
MIR124	laminaran	dimethyl fumarate
mercuric chloride	NOS2	coenzyme Q10
MBD2	CCL2	probucol
IL31	INSIG1	zinc sulfate
CTR9	RBPJ	5,6-epoxyeicosatrienoic acid
CCK	GDF2	guanosine
verteporfin	RHOA	tenofovir
CYP1A1	CLEC11A	SB 220025
GNAS	carrageenan	miglitol
icilin	APC	isopropyl thiogalactoside
ALKBH7	IL18	androgen receptor antagonist (S)-11
PBRM1	PI3K (family)	androgen receptor antagonist (R)-9
SH3KBP1	SB-431542	staphylococcus aureus peptidoglycan G
PIAS2	arachidonic acid	NONG009656
DRD1	ETV5	BMS-214662
CACNA1A	clofibrate	CERT1
azetidyl-2-carboxylic acid	10E,12Z-octadecadienoic acid	lonaprisan
quinacrine	isobutylmethylxanthine	[D-Ala7]-angiotensin I/II (1-7)
corticosterone	ADAP1	glemanserin
CLPP	cyclic AMP	PYDC2
ETS2	TEAD4	enkephalin, methionine
IKBKE	Sos	nitrosomethylurethane
SUMO2	STING1	T-type Calcium Channel
TRIM37	Y 27632	alum compound
POLG	captopril	MIB2
gamma-secretase inhibitor compound E	fenretinide	FBXO31
SENP1	MDK	FBXO3
IKZF2	DDX3X	NTN4
histone deacetylase inhibitor	PPP2R5C	NLRP2
PD173074	NRP1	NMRAL1
TEAD3	LGALS1	VSIG4
PPP3CA	POU2AF1	ARL16
elaidic acid	mir-154	RETSAT
TOX	tazemetostat	PYDC1
HOXA13	ARID1A	CLPX
SAFB2	HNF1B	REM2
TGFB1I1	ROCK2	RPRD1B
LCAT	HSP90B1	CCDC88C
MMP11	MRTFA	OSI-027
THZ1	KDM3A	GPR4
nickel	SAA1	USP50
Endothelin	phenacetin	LBH
mir-451	DETA-NONOate	SPHKAP
BARX2	ADRA1B	ADH5
RPS6KA3	MUC1	VENTX
BHLHA15	CD244	ZNF322
NCR2	JAK2	BEST4
magnolol	melatonin	SENP5
RBM5	BMP4	AGO3

ATP2B2	CEBPD	ETS-ELK1
2-amino-1-methyl-6-phenylimidazo-4-5-b-pyridine	ZC3H12A	CTNNβ-LEF1
FOXA1	alvocidib	AFAP1L2
PD 180970	NFAT5	BCL6 peptide inhibitor
1-methyl-2-pyrrolidinone	SCD	EIF4H
sepantronium	CSF	Inflammasome (Nalp3, Asc, Casp1)
danusertib	HIC1	RNMT
PF-4691502	CREBBP	MCF2L
G0S2	SELPLG	PTPRF
mir-105	alefacept	HLTF
GCLC	CDK9	DAPK3
CD151	NOX4	RAC3
ATP2A1	carboplatin	GSTM1
PAPOLA	ritonavir	SYMPK
ACP1	hydroxyurea	KLHL40
Mt3	MAPK8	TRIM16
TOP2A	cholesterol	LAMA3
ZC3H13	25-hydroxycholesterol	ARIH2
MAD1L1	celecoxib	mir-622
CKS2	isoproterenol	miR-675-3p (miRNAs w/seed UGUAUGC)
1-(2-hydroxy-5-methylphenyl)-3-phenyl-1,3-propanedione	MAP3K14	miR-301a-5p (and other miRNAs w/seed CUCUGAC)
abrocitinib	PLC	mir-186
phenylacetate	superoxide	mir-663
atipamezole	silicon dioxide	Zingiber officiale ginger extract
hoechst 33342	Ptprd	IGK
MM218	N-(3-(aminomethyl)benzyl)acetamidine	N-acetylcytidine
rubitecan	LMO2	RPL15
glycyrrhizic acid	uric acid	CCNDBP1
rasagiline	RAC1	EHD3
GNRH	STAT5a/b	DCTN4
SUMO3	ZNF106	SIGLEC11
lithium	ETS1	OSTF1
saikosaponin A	SUPT20H	gedatolisib
CENPN	reactive oxygen species	poly-L-glutamic acid-peptoid 1 conjugate QM56
motexafin gadolinium	cobalt chloride	FBXO4
2,5-bis(5-hydroxymethyl-2-thienyl)furan	CASP8	BCLAF1
BI 2536	MAP2K7	ILKAP
Cyclin A	NKX3-1	NAGLU
R 406	dinoprost	CRADD
CYTOR	TGFBR1	ADAMTS13
PADI4	piceatannol	COMP
GSTK1	GSK3B	AGO4
CACNA1C	HDAC3	ARNTL2
TRPC4AP	Sn50 peptide	GPR32
PHF10	SP2509	TMEM9B
ADGRF5	metronidazole	DRP2
NELFCD	RELB	DPH1
LTBP4	oleic acid	STAMBP
BANP	SND1	PDHA1
miR-2392 (miRNAs w/seed AGGAUGG)	GNRH1	ADAMTS5
mir-2392	S100A9	RHCE/RHD
SMARCA1	SYK	FXYD5
FALEC	PRKDC	Gm12250
CLTC	chrysotile asbestos	Sf1
RASSF10	LIF	P2rx7
BLACAT1	zerumbone	NLRP1
teasaponin	PTAFR	CLEC2D
perifosine	L-glutamic acid	miR-101b-3p (miRNAs w/seed UACAGUA)
R59949	stallimycin	N-acetyl-lysyltyrosylcysteine-amide
SCH79797	hydroxypropyl-beta-cyclodextrin	CYP4A22-AS1
mechlorethamine	LDB1	fidarestat
domoic acid	ATF2	sorafenib derivative SC-1
epoxyeicosatrienoic acid analog B	colistin	P144
epoxyeicosatrienoic acid analog A	CITED2	bimekizumab
buserelin	DICER1	trimellitic anhydride

dihematoporphyrin ether	ITGAM	amprenavir
4-(1-D-ribitylamino)-5-amino-2,6-dihydroxypyrimidine	NDRG1	bunazosin
ROR2	methylmercury	Ro 32-0432
FOXD2-AS1	enalapril	phosphorylethanolamine
PRR11	histamine	inostamycin
Ppp2c	SPHK1	amlexanox
L1CAM	17-alpha-ethinylestradiol	thiamin pyrophosphate
RPS6KA4	PP1	milrinone
VLDLR	LGALS3	E-4031
RUVBL1	BID	zafirlukast
4-methylnitrosoamino-1-(3-pyridinyl)-1-butanone	TP53COR1	dofetilide
Smad	S-(2,3-bispalmitoyloxypropyl)-cysteine-GDPKHPKSF	dioctyl adipate
IREB2	METTL3	BAY-u9773
aphidicolin	ATP-gamma-S	KRP-297
ammonium chloride	SDCBP	BIBX1522
3-nitropropionic acid	Nfat (family)	hexaarginine-neomycin B conjugate
salirasib	MAP3K1	(S)-flurbiprofen
TNFSF13	kanamycin A	KR 62436
HEIH	BMP2	octanal
EIF4EBP2	TLR9	NSC 651016
linsidomine	EHF	wax ester
Ctbp	daidzein	pca 4230
RHOJ	IL12 (family)	urushiol
CBX7	PLAT	SF 6847
CREBZF	SOX11	phenol
S100B	mir-155	heme arginate
ITGA6	NFkB (family)	echium oil
MAPKAPK3	hexamethylene bisacetamide	NAB2-STAT6
thiazolidinedione	N-formyl-Met-Leu-Phe	GSK2656157
leuprolide	PDPK1	brimapitide
RHO	beraprost	ingenol-dibenzoate
IKZF3	clorgyline	tetrahydropalmatine
HDAC6	SR 144528	KT 5926
isoprenaline	1-((2-chlorophenyl)diphenylmethyl)-1H-pyrazole	(13S)-hydroperoxyoctadecadienoic acid
LIN28B	cytarabine	oleylamide
EGR2	INHBA	trilostane
ATP7B	gemcitabine	17-epiestriol
FCGR2B	GATA3	fluorocitric acid
TNFSF15	SAFB	arylsulfonamide 64B
PDGF-AA	TRPV4	1-phenazinecarboxylic acid
RNF20	CFLAR	alvespimycin
eltanexor	SYVN1	NFE2
rosuvastatin	PTGES	PLA2G6
DLL4	EZH2	epicatechin gallate
PPRC1	PALMD	Ro41-5253
FGF21	ozone	retinol
miR-204-5p (and other miRNAs w/seed UCCCUUU)	HOXD10	9Z,11E-octadecadienoic acid
triciribine	calphostin C	RXRG
glycochenodeoxycholate	TICAM1	OGT
GON4L	CAT	ML290
SNHG11	9,10-dimethyl-1,2-benzanthracene	BMPER
TFAP2E	Alpha catenin	UCN
miR-150-5p (and other miRNAs w/seed CUCCCAA)	berberine	WNT7A
mir-375	FGF1	FOXF1
NPFF	dimethyl sulfoxide	BRD7
ferric ammonium citrate	PLCG2	NR2F6
salmeterol	PDCD1	FABP1
pentobarbital	CTLA4	7-ketocholesterol
zeranol	TREM2	diosgenin
noscapine	SCH 58261	calpain
D-tubocurarine	collagenase	NCOR-LXR-Oxysterol-RXR-9 cis RA
ZFHX3	AS1842856	TNFRSF10A
phenylephrine	methapyrilene	SOX6
puromycin	LCN2	EGLN3
8-pCPT-2-O-Me-cAMP	TNC	TGIF1

ZBP1	leukotriene C4	IGFBP5
RMRP	AKT inhibitor VIII	EXT1
GFI1B	lisinopril	Gm21596/Hmgb1
prazosin	mir-488	immethridine
zymosan A	Notch	TCF20
SOX1	sulforafan	lithocholic acid
ZBTB7B	BMP7	BMP
PAX7	CXCL8	Pka catalytic subunit
H-7	HSPB1	MTA2
geneticin	EPCAM	TRPC1
RUNX1T1	silibinin	SLC9A3
Ck2	GW3965	ellagic acid
SLC16A3	PTGER4	GW7647
MEMO1	CAV1	NR2F2
nicotiflorin	V-PYRRO/NO	4-CMTB
pictilisib	selenomethylselenocysteine	GY1-22
bicyclol	HSPA1A/HSPA1B	Convulxin
Ccl6	FOXL2	thioguanine
RFXAP	apigenin	minoxidil
SHOX	docetaxel	ergosterol-5,8-peroxide
PTGES3	Cdk	HS-243
RPL11	vitamin K3	R2HBJJ
CD160	hymecromone	NONMMUG036351
MCAM	PLAUR	DPTIP
niflumic acid	folic acid	Osteocalcin
prodigiosin	di(2-ethylhexyl) phthalate	narciclasine
SR1078	metformin	1-palmitoyl-2-arachidonoyl-phosphatidylcholine
3-beta,17-beta-androstanediol	etanercept	myosin-light-chain kinase
IL2RG	JAG2	methysticin
SIM1	MAFB	Stat1-Stat3
miR-29b-3p (and other miRNAs w/seed AGCACCA)	diphtheria toxin	Egfr-Erbb2
Rar	MAP2K5	Stat5b dimer
ZBED6	IFIH1	NIK
GAB2	Gsk3	gliquidone
LOC105372576	JAG1	LRRC8E
DTX1	C5	NIBAN2
rifaximin	Ro31-8220	NOP58
GMNN	plicamycin	Il3r
SOCS3	IRF5	GPC6
mir-22	zidovudine	NDUFAF3
DLK1	evodiamine	MSRA
CGS 21680	ilomastat	PSMD14
fisetin	MAOA	RPS6KA
fish oils	bryostatin 1	LINC01006
K+	vismodegib	KLHL2
tert-butyl hydroperoxide	JUND	FERMT3
A-Fos	BRD2	OLA1
PPP1R15A	CGA	INAVA
TUG1	GLI2	bis(4-hydroxycinnamoyl)methane
PD 169316	paraquat	UBE2D3
tranylcypromine	RNF31	gelatinase
acetaldehyde	JAK1	Fetal Hemoglobin
NUP98-NSD1	CRH	OXSI-2
2,4,5,2',4',5'-hexachlorobiphenyl	CIP2A	pycnogenols
CA9	TSH	Hnp alpha
NF1	RPTOR	SRGAP1
monophosphoryl lipid A	IL6R	vistusertib
RUBCN	ZEB1	kahweol
SELENOS	tiron	SERTAD1
48s	EPOR	MR1
IGFBP7	hemozoin	PAG1
ZBTB33	erlotinib	PLEC
PDGF (family)	let-7a-5p (and other miRNAs w/seed GAGGUAG)	EMP2
PCK1	losartan potassium	BAG4
NUP62	TSC22D3	SEMA3D

APBB1	1-palmitoyl-2-(5-oxovaleroyl)-sn-glycero-3-phosphorylcholine	DNASE1
RBM5-AS1	IL36A	AGAP2
SCH772984	ITGAX	ABCC2
oligomycin	adenosine	TRD
monorden	vitamin D	PKC delta V1-1 peptide 8-17
NUP98-KDM5A	L-triiodothyronine	CLCN3
asiatic acid	pyrrolidine dithiocarbamate	mir-370
NSC 172285	IL36B	mir-673
delta-12-prostaglandin J2	Mt2	miR-224-5p (miRNAs w/seed AAGUCAC)
ADRA1	ACTN4	ATOX1
(-)-gossypol	SOX4	NPHS1
ZBED1	PRKN	EEF1D
FICD	IL11	HYAL2
SNHG3	SMAD4	CEACAM6
HYAL1	Ifnar	CEACAM21
miR-873-5p (and other miRNAs w/seed CAGGAAC)	ICAM1	RALGDS
miR-292b-5p (and other miRNAs w/seed CUCAAAA)	FCGR2A	MGAT4A
SRCAP	F3	EIF2B1
KIDINS220	FOXO4	MKNK2
ASH2L	KIT	PTTG1IP
FDPS	NCOR2	SRPK2
BBC3	CCR5	RPS6KA2
GNL2	WNT5A	TRPC6
HOXD12	C1QBP	CTAG1A/CTAG1B
NEK2	ferulic acid	ZFY
GCLM	bardoxolone methyl	ANK1
MI-773	N(G)-monomethyl-D-arginine	DDX4
picolinic acid	NR4A1	ACS84
oxytetracycline	TNFSF14	KIRREL1
CBFB-MYH11	TRIB3	RNH1
acetyl-11-keto-beta-boswellic acid	andrographolide	CEACAM7
lipofermata	vinblastine	CD72
GAS6	NEIL2	LAG3
HMGN1	cephaloridine	RASSF7
titanium dioxide	baicalin	RECQL
NFU1	ITGA5	YWHAE
HSPA5	ITGB3	NPHS2
delta-9-tetrahydrocannabinol	platelet activating factor	GLUD1
CRNDE	VEGFB	ERCC6
TFDP1	CLU	ERC1
NGLY1	amphotericin B	FAM177A1
miR-221-3p (and other miRNAs w/seed GCUACAU)	nystatin	Cacnb1
Klrk1	dexmedetomidine	CRIP2
honokiol	CALC	CYP2C9
RANBP3L	POLR2M	Naip1 (includes others)
GSK2816126	CCL3	SCT
niclosamide	kaempferol	ADAMDEC1
GPX8	UCN-01	TIGAR
PARP	TGFB2	RDH16
CERS6	CD2	RPL10
TLR2/3/4/9	CSF1R	ATAD2
nirogacestat	VIP	CNTFR
ENO1	BCL2	HERC5
mir-449	LEF1	(E)-1-(2-nitrovinyl)naphthalene
ALOX15B	cocaine	(E)-9-(2-nitrovinyl)anthracene
IL9R	anakinra	N-butyryl arginine
SELE	glutathione	3-hydroxybutyrate arginate
LUM	BAPTA-AM	anastrozole
NCR1	norepinephrine	prinaberel
PEA15	N-nitro-L-arginine methyl ester	filipin
KU-55933	GNA12	astemizole
tridecanoic acid	IFNAR1	cilomilast
amphetamine	DDX58	neratinib
salinosporamide A	IFNL1	epalrestat
MAPK13	ITK	ethylisopropylamiloride

YAP/TAZ	GDNF	albendazole
2-mercaptoacetate	CD9	rocuronium
agmatine	ADAMTS12	tarenflurbil
verapamil	CXCR4	KIRA6
1-palmitoyl-2-oleoylphosphatidylserine	rotenone	AX15839
mir-26	8-chlorophenylthio-adenosine 3',5'-cyclic monophosphate	etodolac
B2M	MEN1	ethyl gallate
citarinostat	IRGM	phloretin
Rxr	CCL11	mycophenolate mofetil
CYP1B1	IFNA1/IFNA13	nickel subsulfide
DPH5	daporinad	G141
olaparib	GJA1	CDD450
AZD8055	KRT17	mitochondria-targeted paraquat
PRF1	PTPN11	C66
FCGR1A	grape seed extract	voriconazole
H3-3A/H3-3B	5-hydroxytryptamine	Ac-YVAD-CMK
Gm12602	cinnamaldehyde	myristoylated PKC-zeta pseudosubstrate peptide inhibitor
costunolide	ShK-223	L-JNK inhibitor I
1,1-bis(3'-indolyl)-1-(4-hydroxyphenyl)methane	ABT-737	TFLLR
di-2-pyridylketone 4-cyclohexyl-4-methyl-3-thiosemicarbazone	PD 153035	glutathione diethyl ester
mannose	SMARCA5	indolepropionic acid
PLP1	lipid A	(indol-3-yl)pyruvic acid
topiramate	estriol	R-etodolac
TNFRSF1B	conjugated linoleic acid	quinine
MMP3	SKIV2L	dobutamine
SMARCA2	WWTR1	mizoribine
2-methoxyestradiol	N-Ac-Leu-Leu-norleucinal	3-hydroxykynurenine
CDK8	WNT1	senicapoc
Npm	advanced glycation end-products	arginine butyrate
7beta-hydroxycholesterol	3-methyladenine	2R,4R-4-aminopyrrolidine-2,4-dicarboxylic acid
4-nitrobenzoic acid	TAC1	ponasterone A
MSK1/2	lithium chloride	Gd3+
PLA2	IRF2BP1	MYRF
CP-724,714	MEOX2	UTP
Calcb	TRAF6	ZNF503
Rbx1	PRKAA2	BMPR1B
MBNL3	Mt1	SMARCC1
FBXO25	irinotecan	PAX5
SGF29	CNOT7	Smad2/3-Smad4
ZBTB49	BACH2	IFNL3
SMYD5	ERBB4	BATF
BATF2	methamphetamine	isoflurane
ZNF385B	STAT2	dichlorovinylcysteine
NCEH1	IRF7	PPARGC1B
PARM1	dinaciclib	LPIN1
STAT3/5	DGKA	NFATC4
OMG	putrescine	PLAGL1
SELENOP	midostaurin	IRAK3
NMB	ADAM10	MECP2
miR-708-5p (and other miRNAs w/seed AGGAGCU)	molybdenum disulfide	Bvht
miR-16-1-3p (miRNAs w/seed CAGUAUU)	fatty acid	ADAM17
miR-292-3p (and other miRNAs w/seed AGUGCCG)	ZBTB10	FECH
TPPP3	HMOX1	DNASE2
ARHGEF28	PF4	TG
CCT5	TNFRSF1A	ID1
MUC16	TBK1	AGTR2
DST	IgG1	ADIPOR1
VANGL1	ADRB3	CGP 74514A
PLEKHA1	3-aminobenzamide	inecalcitol
DDX24	W7	dienogest
WASHC1	biochanin A	rivaroxaban
RAMP3	propylthiouracil	ITPR
PLEKHA2	CGAS	Bvr
CDC23	TLR7/8	15-LOX
GALR2	ADP	yangonin

CYP24A1	Sod	CC8490
2-methoxy-N-(3-methyl-2-oxo-1,4-dihydroquinazolin-6-yl)benzenesulfonamide	MMP8	ticrynafen
troxerutin	tanshinone II	smectite
HUS1	C3AR1	sucralose
LPCAT1	Il3	NR2F1-AS1
mir-434	CISH	Snhg6
BAI	bardoxolone	ZFP42
Mia2	calcipotriene	IL17RC
hydroxyl radical	ESRRA	BRD3
amsacrine	Mapk	DNAJB4
1-o-hexadecyl-2-o-methyl-rac-glycerol	FLT1	Carlr
gliclazide	FANCC	WAPL
nisoldipine	N-acetylmuramyl-L-alanyl-D-isoglutamine	SP6
kaolin	BUD23	CYP2C8
cation	ARRB1	HOXC5
NVP-BHG712	HNRNPU	USP35
1,1-bis(3'-indolyl)-1-(4-chlorophenyl)methane	ATG7	DUOX1
teleocidins	AGER	Slfn1
trehalose dimycolate	TYROBP	NLRP6
tetrahydrouridine	ATP	C1QL3
L-canavanine	TNFAIP3	ORAI3
GC-GCR dimer	salicylic acid	IGSF11
USP7	n-3 fatty acids	TMEM119
DDX25	NADPH oxidase	MUCL1
ETS	ELK4	SLC39A4
PDGF-DD	TSLP	KDM7A
KDM4A	leucine	NXF1
SEMA7A	IL17RA	pertuzumab
AURKA	green tea polyphenol	Casein
DEF6	SU6656	NCF2
mir-101	4-phenylbutyric acid	ZFYVE9
BAP1	CDH11	TLCD3A
FSHB	BCAP31	MOAP1
CSNK2A1	PYCARD	ADRA2B
diallyl trisulfide	NFKBIZ	HABP2
ruxolitinib	nitroarginine	TJP1
NEDD9	N-cor	miR-27a-5p (miRNAs w/seed GGGCUUA)
SOX3	HMGB1	mir-431
monobutyl phthalate	HDAC5	miR-330-5p (and other miRNAs w/seed CUCUGGG)
KDM2B	KCNN4	SNX17
arginine	hydroquinone	SKAP2
NEUROG2	bempedoic acid	BMS 182874
N-acetylsphingosine	propofol	K-604
MYF6	S-nitrosoglutathione	CEACAM5
SIX2	Angiotensin II receptor type 1	GALR1
E2F6	neuroprotectin D1	VSX2
L-serine	IGFBP2	ACSL3
TASP1	TXN	anatabine
YOD1	ciprofibrate	PER3
SMOC2	trinitrobenzenesulfonic acid	UBTF
CYSLTR2	TGFB3	SIGMAR1
astragalin	pioglitazone	CD58
SWAP70	PPP2CA	SIGLEC10
RUVBL2	emodin	CTNNAL1
AIMP2	8-bromoguanosine 3',5'-cyclic monophosphate	WNT6
CHRNA3	NCSTN	LDN-193189
CNB-001	amiodarone	SERPINB7
sesame oil	phorbol 12,13-dibutyrate	SCTR
clonidine	Pde	RPS18
Trolox C	TRG	ALX4
lidocaine	MC1R	GNPAT
carmustine	YBX3	IL20RA
enoxacin	PD 168393	SLC9A3R2
picropodophyllin	LIPE	TGIF2LX
triclosan	dexamethasone phosphate	RPL35A

ZC3H14	ADCY	PARP10
PTBP1	Ikb	CES1
LAMA5	GDF15	AMELX
IL1RAP	IL37	ARF1
DNAJB6	THZ2	RPL12
NCAM1	MMP2	TRIM6-TRIM34
HOXB4	CASP3	RPL5
DDX17	MAP3K7	ANXA6
HELLS	MYOC	HS6ST2
KT5823	ADORA2A	PF-8380
ethidium	sunitinib	LY5
mir-132	NRG2	griseofulvin
miR-26a-5p (and other miRNAs w/seed UCAAGUA)	birabresib	diethylcarbamazine
IFRD1	Ifn	acetoacetic acid
pilocarpine	tyrphostin AG 1478	KP-SD-1
MCB-613	3M-001	clobenpropit
CYD0618	XDH	trimetazidine
ganciclovir	FOSB	LY117018
conduritol epoxide	lysophosphatidylcholine	moxifloxacin
GPIIB-IIIA	galactosylceramide-alpha	lomefloxacin
GTP	brefeldin A	nabumetone
(R)-limonene	CpG ODN 1668	flavonoid
VAV	TCF12	betamethasone valerate
F Actin	mir-10	cadmium sulfate
zinc oxide	NCOA2	sodium alum
obatoclax	OPA1	WZB117
glycitein	PDCD4	Phe-Pro-Arg-chloromethyl ketone
SLAMF9	pomalidomide	myristoylated protein kinase C peptide inhibitor
Snhg20	taxifolin	limonene
N-propargyl-1(S)-aminoindan	BSG	succinylacetone
HAND2-AS1	TEAD2	procysteine
SLC39A9	mir-30	isoacteoside
RASSF3	USP22	D-mannose
IKBIP	IGHM	N-butyldeoxynojirimycin
TMEM106A	16,16-dimethylprostaglandin E2	ecdysterone
ZDHHC2	Ginkgo biloba	steroid hormone
RAB7	N(2)-(gamma-D-glutamyl)-meso-2,2'-diaminopimelic acid	pyocyanin
vaccenic acid	PELI1	Pb2+
TFPI	NDUFA13	FST
PRKD2	MVP	PIAS1
CSE1L	dehydroxymethylepoxyquinomicin	KDM6B
MKI67	RUNX3	CFB
CNGB3	KLF11	TBX5
NEK10	1,25-dihydroxyvitamin D	Calcineurin A
BCL2L12	AKT3	IFT88
HAS1	USP18	FGFR4
mir-708	NPPA	deoxycorticosterone acetate/potassium chloride/sodium chloride
mir-548	dithiothreitol	TYK2
APAF1	suramin	4-(2-aminoethyl)benzenesulfonylfluoride
ORM1	linoleic acid	RYR1
MAGED1	isotretinoin	TET1
AATF	BCL2L11	miR-338-3p (miRNAs w/seed CCAGCAU)
CHD3	ITGA1	UPF1
SRPK1	USP19	BECN1
LRIG1	IL10RA	advanced glycation end product 3
DYNLL1	IFN alpha/beta	(+)-fluprostenol
STK38	MACROH2A1	ginsenoside Re
CASP2	PTHLH	p38 Sapk
STX2	azoxymethane	ILX-23-7553
URI1	fingolimod	DAPK
RNF17	mir-204	DIPQUO
CDC20	TNFSF12	asialo GM1 ganglioside
CBLC	CYP19A1	dibutyryl cGMP
HIVEP2	NVP-TAE684	fludrocortisone
RPS20	PLA2G10	Hbb

OMP-52M51	SQSTM1	arginase
CXXC4	TNFRSF9	Ctnna
fenebrutinib	MYB	IFN alpha receptor
EGTA acetoxymethyl ester	corticosteroid	antioxidant
naphthalene	SPDEF	beta-sitosterol
pyridoxamine	cadmium	SMPD3
risedronic acid	PKM	Jun-ATF2
octyl gallate	zinc	NLR
catechol	NCOA1	IRF3-IRF7
FC-99	IDH1	MLYCD
PDZ1i	IL12B	GPR17
monodansylcadaverine	NFATC1	SSBP2
Z-IETD-FMK	CERS5	propolis
propyl-2-(8-(3,4-difluorobenzyl)-2',5'-dioxo-8-azaspiro[bicyclo[3.2.1] octane-3,4'-imidazolidine]-1'-yl)acetate	RNF152	TRIM58
sparfosic acid	VTX-2337	HLA-DR
L-asparagine	rituximab	GALNT4
oxamic acid	tripterine	MCOLN2
quisqualic acid	miR-424-3p (miRNAs w/seed AAAACGU)	CDH16
miR-27a-3p (and other miRNAs w/seed UCACAGU)	mir-920	CREB3L4
SULF2	GFRA2	RREB1
RNF138	LFM-A13	GATAD2A
miR-18a-5p (and other miRNAs w/seed AAGGUGC)	AZD4573	RECQL4
NT5E	PLCG1	1810058I24Rik
DYRK1A	HRG	ETV7
Cdkn1c	HNRNPAB	EIF3I
FMR1	bisphenol A	PF-562271
NSD2	CLEC14A	MIR3936HG
OVA-8	ITGA4	RPS11
ARRB2	S100P	ACSL6
anandamide	cyclic guanosine monophosphate-adenosine monophosphate	MRS 2578
N-methyl-D-aspartate	3,3'-diindolylmethane	toosendanin
TRAP1	mir-17	MCF2
MSTN	CCN5	TLN1
ALDH1A2	artesunic acid	miR-466m-3p (and other miRNAs w/seed ACAUACA)
MAPK11	ZNF710	MIR3662
NOSTRIN	STK4	neflamapimod
UXT	PLK2	GAS7
Hsp27	KN-62	TIAL1
THBS1	tetrandrine	LSP1
FOXP1	POR	PROS1
histone deacetylase	NGFR	CDR2
Ren2	thalidomide	SEPTIN9
HCAR2	S100A4	FLOT1
METTL1	hemin	PSAP
CNOT6L	1'-acetoxychavicol acetate	PFN1
mir-194	AIMP1	VGLL4
CHEK2	OCLN	BRD8
PDGFRA	DEK	BCAR3
CCR6	prostaglandin D2	CEL
PARP9	Hbb-b1	RAD51C
clenbuterol	Bay 11-7082	CCL25
acadesine	aldosterone	PRMT2
UBE3A	KAT5	EDIL3
GATA4	MAP2K6	MPO
LTA	CDKN1B	SLC25A12
ABCA1	NFKBIB	ORC2
ESRRG	ANXA1	astaxanthin
BBP-398	2,3-bis(4-hydroxyphenyl)-propionitrile	KRT10
gossypin	IRF2	RAB4A
CERS2	SSRP1	RPS12
POLR3G	clodronic acid	fluticasone
WWC1	diallyl disulfide	2-cyclohexen-1-one
SLC7A2	CCN2	LY379196
AFAP1-AS1	BMP10	silicon phthalocyanine
FLZ	nocodazole	cinnamyl-3,4-dihydroxy-alpha-cyanocinnamate

BMS-754807	SERPINA1	lansoprazole
PRAME	fluvastatin	pentamidine
DDB2	RNY3	phenol derivative
PAX4	CTSS	AD-5057
KRIT1	PLK4	aurothiomalate
ITGB8	geranylgeranyl pyrophosphate	orantinib
BEX2	11,12-epoxyeicosatrienoic acid	cathelicidin-WA
NDP	Fibrinogen	dexamethasone/INS/isobutylmethylxanthine
pentylenetetrazol	Iga	DASA-58
mesalamine	CpG ODN 2395	cannabinoid
pepstatin	Usp17la (includes others)	9-hydroxyoctadecadienoic acid
dehydrocostus lactone	ST8SIA1	dideoxyadenosine
thymidine	SN-011	dapsone
androstenediol	HDL	Hg2+
SMC1A	FGF8	crizotinib
TCF7	THRB	TANK
testosterone propionate	RETN	XAV939
ARHGAP31	carbon monoxide	PRKAR1A
ICMT	PROC	exenatide
CX-5461	FYN	SH42
SPARC	nifedipine	
memantine	RIPK2	
PDLIM2	TCOF1	
ELL2	TRIM24	
3,4,5,3',4'-pentachlorobiphenyl	CpG ODN 2006	
SOD2	IL27RA	
lnc-CXCL2-4	RORA	
AHI1	sphingosine-1-phosphate	
Trp53cor1	RARB	
MTA3	IL1RL2	
HEXIM1	CSK	
NOS1	hydrogen sulfide	
DNMT1	MIF	
DSCAM	orlistat	
mir-145	2-(4-acetoxyphenyl)-2-chloro-N-methylethylamine	
ATN1	TRADD	
Fgfr	CCR1	
FAT1	1-methyl-4-phenylpyridinium	
SEL1L	MALP-2s	
miR-503-5p (miRNAs w/seed AGCAGCG)	hydrochloric acid	
ponesimod	YBX1	
NEDD4	CD247	
tomatidine	NR5A2	
triflusal	bezafibrate	
L-carnitine	IL17R	
MR-409	NOX1	
riboflavin	IL23	
dichlororibofuranosylbenzimidazole	RORC	
melphalan	PRKCZ	
PRKCG	SOST	
AKT2	eplerenone	
Mir122a,b	CpG ODN 1826	
1,2-dimethylhydrazine	CYLD	
olomoucine	AGTR1	
triamcinolone hexacetonide	ribavirin	
rh-endostatin	alitretinoin	
4732491K20Rik	(+)-MK-801	
patulin	Sphk	
SCGB3A2	Org 48762-0	
ST6GALNAC1	PDE3A	
NSUN2	15-E2-isoketal modified phosphatidylethanolamine	
SH2D5	dacarbazine	
BCAS2	manidipine	
TP73-AS1	sanguinarine	
KMT5B	Calcineurin protein(s)	

RDH8	TRAF3IP2	
APC (complex)	IL25	
catumaxomab	ITGB1	
HLA-DQ	RNASEH2B	
IER3	cholecalciferol	
WNT2	GRP	
NUDT16L1	LUCAT1	
KLF12	PLD1	
AGR2	HOXA7	
miR-494-3p (miRNAs w/seed GAAACAU)	dicarbethoxydihydrocollidine	
SDC4	deoxynivalenol	
GATAD2B	chloroquine	
MARCO	IL-1R	
TP53INP1	LRP6	
GNL1	CD14	
C4A/C4B	SERPINF1	
MLLT3	JAK	
TDG	lonafarnib	
GJA8	SERPINC1	
casticin	CXCL2	
ADCYAP1R1	S1PR3	
RAPGEF4	phenyl-N-tert-butylnitrone	
ABCB7	oxysterol	
CTH	ACVRL1	
KLRB1	ACKR3	
CLEC2A	PML-RARA	
MARCHF1	trastuzumab	
JMY	RARRES2	
nicorandil	desipramine	
ubiquinone 9	1-chloro-2,4-dinitrobenzene	
trimethyltin	lipoarabinomannan	
fluphenazine	tauroursodeoxycholic acid	
batatasin I	CFTR	
vardenafil	NQO1	
leucovorin	4-methylene-2-octyl-5-oxotetrahydrofuran-3-carboxylic acid	
murabutide	ELN	
muscarine	NOD2	
ANXA2	CLEC7A	
TIMP1	2'3'-cyclic guanosine monophosphate-adenosine monophosphate	
CBX3	fucoidin	
MMP12	POMC	
PRKCB	PRKCQ	
RABGEF1	ELF4	
IFNA4	HIPK2	
Esrra	MEF2D	
CXCL6	Hif1	
ladostigil	FLI1	
trypsin	GPS2	
MDGA2	IL6ST	
DHCR24	PARP1	
LECT2	pregna-4,17-diene-3,16-dione	
alisertib	Cbp/p300	
CSNK2B	CDH2	
PAK4	resolvin D2	
LIPA	MAP3K3	
mir-185	MLN120B	
miR-101-3p (and other miRNAs w/seed ACAGUAC)	P2RX7	
HIF3A	LILRB1	
THBS2	bisindolylmaleimide iv	
CRY2	GW9662	
BCAR1	AIRE	
PDE4B	glycine	
CD180	BMS-345541	
BCR	FZD8	
CALCB	tofacitinib	

GHSR	elastase	
PIM3	TAF1	
AK1	ITGAL	
FZR1	CXCL3	
CRY1	MAML1	
OPRM1	GAB1	
4-hydroxycinnamyl aldehyde	thymoquinone	
KMT2A-AFF1	DPP4	
astressin 2B	sildenafil	
ryanodine	colfosceril palmitate	
gambogic acid	adalimumab	
S-equol	CINP	
miR-205-5p (and other miRNAs w/seed CCUUCAU)	CHIA	
LTBP1	XRN2	
LTF	P-TEFb	
nilvadipine	FLII	
WR 1065	miR-181a-2-3p (and other miRNAs w/seed CCACUGA)	
RPS6KB1	miR-331-3p (miRNAs w/seed CCCCUGG)	
ARHGAP21	TIAM1	
Ap2	AHNAK	
mir-221	BAG2	
PAK1	LPAR3	
IL7R	VR23	
SCAP	verlukast	
dacinostat	2-mercaptoethylguanidine	
peoniflorin	chlorophyllin	
MSI2	ursolic acid	
IFNW1	VAV1	
mir-192	CTSB	
IAPP	VTN	
KCNJ10	urethane	
RFX1	crocidolite asbestos	
STAU1	proteasome inhibitor PSI	
TMBIM6	bufalin	
GFAP	TLR5	
antimycin A	SIX1	
LATS2	Collagen type I (complex)	
EHMT2	FKBP10	
SMAD2	morphine	
PAX6	ATG5	
TLX1	Cyp4a14	
NCL	EGR4	
CDKN2B	AGI-1067	
MAP2K2	theaflavin	
CARD11	TRAF3	
TFPI2	cucurbitacin I	
CD47	IgG2a	
JAK inhibitor I	SRT1720	
CpG ODN 2216	FZD7	
miR-450a-5p (and other miRNAs w/seed UUUGCGA)	PGLYRP2	
COL5A1	B4GALNT1	
butylated hydroxyanisol	CD163	
[(R)-6-(4-(4-benzyl-7-chloronaphthalen-1-yl)-2-methylpiperazin-1-yl)] nicotine nitrile	LETMD1	
trapidil	PPP5C	
pentosan polysulfate	CCL3L3	
mepazine	HPSE	
galactosylceramide	sevoflurane	
GDP	[D-Ala2,N-Me-Phe4,Gly5-ol]-Enkephalin	
Cyclin B	cyclomaltodextrin	
DUB	IL12A	
Esr1-Estrogen-Sp1	ADORA2B	
cetylpyridinium	Hbb-b2	
GALNT14	IFNLR1	
TMBIM1	Pam3-Cys	
RHOBTB2	IFNGR1	

USP6	TLR7	
PRMT7	3-deazaneplanocin	
LINC00261	selumetinib	
PTPMT1	2-aminopurine	
CERS4	BIRC3	
SPON2	ketamine	
SMCR8	tosylphenylalanyl chloromethyl ketone	
PRR7	ciglitazone	
EDEM1	ROCK	
OTUB2	NONO	
Fascin	ALOX15	
STAB1	IGFBP3	
C1QL1	SERPINE2	
potassium channel	BTG2	
NAA30	CAMP	
TRPM3	TRPM2	
KLHL21	ITGAV	
ATP13A2	LTB4R	
GPD1L	PARK7	
EIF4F	pitavastatin	
ATP1A2	ceramide	
Gamma tubulin	CDK5R1	
pimasertib	vanadate	
CASQ1	miR-34a-5p (and other miRNAs w/seed GGCAGUG)	
Pdi	NRF1	
foretinib	zoledronic acid	
VAMP7	PIK3CG	
ARFGEF2	LPL	
NAB1	EIF2S1	
PMEPA1	heparin	
PURB	SAA	
GSTA4	CERK	
MCRS1	shikonin	
MADD	TICAM2	
miR-515-3p (and other miRNAs w/seed AGUGCCU)	BTRC	
miR-2682-5p (and other miRNAs w/seed AGGCAGU)	ERF	
miR-296-3p (miRNAs w/seed AGGGUUG)	AZGP1	
miR-376a-5p (miRNAs w/seed UAGAUUC)	H-[1,2,4]oxadiazolo[4,3-alpha]quinoxalin-1-one	
miR-149-3p (and other miRNAs w/seed GGGAGGG)	DKK1	
P4HB	3M-011	
PBK	SFLLRN (PAR1-activator)	
TPM1	dimethylnitrosamine	
DOK2	CEBPE	
USP14	CBL	
FTMT	RETNLB	
WWP1	U46619	
STRAP	xanthine	
TOR2A	Rac	
KPNB1	LILRA2	
PDZD2	SAMHD1	
TXNDC5	PLCE1	
RBM3	WWP2	
MXD4	NEU1	
Ank2	ethylenediaminetetraacetic acid	
GNG2	anti-benzo(a)pyrene-diol-epoxide	
PAGR1	Cd2+	
INHBC	MSR1	
VEZF1	PLA2G4A	
CASP8AP2	DHX9	
zotiraciclib	sulfasalazine	
TCP1	elovanoid N32	
8-butyl-10-[(3-methoxy-5-pyrrol-2-ylidenepyrrol-2-ylidene)methyl]-11-azabicyclo[7.2.1]dodeca-1(12),9-diene	RXRB	
MXD3	TARDBP	
RING1	GDF11	
EEF1A1	Hoxa11os	

RPL37	IL26	
Irs4	PRTN3	
RPL23A	LGR4	
BUB1B	Srgn	
capivasertib	TXNIP	
UIMC1	luteolin	
JPH203	SPIB	
AZ-960	IGF2BP1	
beta2 adrenergic receptor agonist	CD300LF	
N6-carboxymethyl-lysine bovine serum albumin	IL17C	
compound 48/80	CHI3L1	
polyhydroxyethyl methacrylate	PROCR	
nitrous oxide	NFIX	
ODN1411	calyculin A	
RX-821002	TBP	
glyceollin	Nr1h	
3,4-dihydroxybenzaldehyde	zymosan	
benzamide	POLR2A	
1-heptanol	CD3E	
incyclinide	C5AR1	
10-decarbamoylmitomycin C	ALB	
idronoxil	CRP	
taprostene	Ras homolog	
4-amino-6-hydrazino-7-beta-D-ribofuranosyl-7H-pyrrolo[2,3-d]-pyrimidine-5-carboxamide	TCF/LEF	
REC2923	Lymphotoxin	
3,4-methylenedioxyamphetamine	GNAI3	
Ala-Tyr-Pro-Gly-Lys-Phe-NH2	SOCS6	
bacitracin	RNASE1	
3,7-dimethyl-1-propargylxanthine	elovanoid N34	
MEDICA 16	C3	
AVI-4126	interferon alfacon-1	
phosphorothioate oligodeoxynucleotide	resatorvid	
1,4-bis[2-(3,5-dichloropyridyloxy)]benzene	IKK (complex)	
JAK1/2	indican	
MAP2K3	TERC	
CD86	ING1	
bicalutamide	KN 93	
chondroitin sulfate A	NAMPT	
Actin	mir-196	
withaferin A	CAPN3	
LOXL1	POLDIP2	
LIMS1	SIRPA	
TAC4	Z-551	
CBX2	chlorogenic acid	
FCER2	eritoran	
MSI1	L-alpha-lysophosphatidylcholine, palmitoyl	
BMX	1-palmitoyl-2-oleoylglycero-3-phosphoglycerol	
LAMC1	lunasin	
HOXA4	[Lys15,Arg16,Leu27]VIP(1-7)GRF(8-27)	
CELF1	edratide	
UBR5	MARCHF3	
picryl chloride	FENDRR	
pyruvic acid	Gpcr	
SRA1	JADE2	
SPOP	ZMIZ2	
SPZ1	SIRT4	
SP100	Tlr12	
miR-293-5p (and other miRNAs w/seed CUCAAAC)	rigosertib	
HDAC7	FUT4	
TNFRSF13B	TMSB10/TMSB4X	
PCDH11Y	PELI2	
HBEGF	CDC27	
12(S)-hydroxyeicosatetraenoic acid	scoparone	
Vhl	SD-282	
meldonium	G Protein I	

rhodamine 6G	grepafloxacin	
CTNS	vanadyl sulfate	
ARID3B	mini-GAGR	
GPR84	allosamidin	
PPT1	Go 6976	
Meg3	ACE	
SLC39A8	SFN	
HSD17B12	1810019D21Rik	
EHD2	GABPA	
SULT2B1	nickel chloride	
ZNF382	Laminin (complex)	
IL-2R	Hsp70	
CD8A	IRF9	
SLC25A4	pristane	
CD82	doxycycline	
U1 snRNP	NLRP12	
TTN	NFIL3	
mir-190	SIRT6	
miR-192-5p (and other miRNAs w/seed UGACCUA)	ZNF217	
SPN	GIP	
Ifi202b	IRF6	
ATF7	BML-111	
DSG2	Tlr13	
CUL7	FRS3	
FKBP1A	IL10RB	
MAD2L1	3-hydroxybutyric acid	
MAD2L2	polymyxin B	
UACA	perilla alcohol	
BLM	TNFRSF18	
NEU3	miR-199a-3p (and other miRNAs w/seed CAGUAGU)	
PROKR1	MAPK12	
ONECUT2	POU2F1	
TNRC6A	ADIPOQ	
RFX4	IRS2	
NCR3	CTBP1	
GADD45GIP1	miR-223-3p (miRNAs w/seed GUCAGUU)	
PCBP1	NMU	
ULBP1	7(R)-maresin 1	
Gm20703	acteoside	
AP20187	curdlan	
raclopride	THRA	
temsirolimus	KMT2D	
GW 5074	CCR2	
benzoic acid	Nos	
ISRIB	COL1A1	
rabeprazole	LY96	
magnesium sulfate	IL2RB	
PCM1-JAK2	IRAK2	
cinnamon powder	DGAT1	
retinaldehyde	sanglifehrin A	
polaprezinc	mirdametinib	
garcinol	deferasirox	
LMNB1	xanthohumol	
SH3TC2	nicotinic acid	
MTORC1	rolipram	
colchicine	HDAC4	
rifampin	ZMPSTE24	
CaMKII	ANGPT1	
TRIM2	E. coli serotype 0127B8 lipopolysaccharide	
KDM4C	AZ5576	
ZNF148	diphosphoryl lipid A	
MTM1	1,4-glucan	
NTN1	syringin	
STAT	urtica dioica extract	
MBTD1	4-oxo-2-nonenal	

ISLR	MAML	
HSF2	2-methoxycinnamaldehyde	
ethylene glycol tetraacetic acid	POPDC2	
ezetimibe	DSC2	
YWHAZ	febuxostat	
RACK1	IKKA/B	
VAV3	diaziquone	
CPE	RPL19	
FAAH	mir-331	
ramipril	HPS1	
cetuximab	AZU1	
(Z,E)-5-(4-ethylbenzylidine)-2-thioxothiazolidin-4-one	CD8B	
FOXG1	3-hydroxydodecanoic acid	
CHRNA1	6-n-octylaminouracil	
EHHADH	imidazolo-oxindole PKR inhibitor C16	
miR-374b-5p (and other miRNAs w/seed UAUAAUA)	ATB-346	
CBX8	E-c-HDMAPP	
DIRAS3	desethylamiodarone	
CPEB1	LZ1 peptide	
IL18R1	moringa oleifera aqueous seed extract	
CCL19	moringa oleifera diluted seed extract	
SP2	dapansutrile	
HNRNPD	Z-YVAD-FMK	
TOPBP1	Z-WEHD-FMK	
BTC	vemurafenib	
Ptgs2os2	CD69	
RGS1	MST1R	
lith-O-Asp	ORMDL3	
FITC	IL17a dimer	
ethylene dimethanesulfonate	ACTB	
fevipiprant	RXRA	
N-carbobenzyloxy-leucine-leucine-norvalinal	maslinic acid	
deguelin	lipoteichoic acid	
U18666A	Am 580	
NEUROG1	FGF19	
LMNA	LAMP2	
STEAP3	MEX3A	
AIP	mir-130	
RARG	CYBB	
HDL-cholesterol	Tlr	
TRH	Wnt	
TFAP2B	VitaminD3-VDR-RXR	
CIDEC	PRKACA	
ceruletide	ILK	
USF1	APOL1	
ASCL1	PARP14	
EHMT1	FFAR1	
AICAR	TPCA-1	
mir-19	manumycin A	
quinolinic acid	SD6	
sphingosylphosphocholine	adenine	
L-cysteine	CHD1	
dopamine receptor	LBP	
bazedoxifene	KLK5	
L-type Calcium Channel	IL19	
S6K1	premarin	
bafilomycin A	ethyl pyruvate	
HTR7	D609	
SENP7	miR-30c-5p (and other miRNAs w/seed GUAAACA)	
OLFM4	enterotoxin B	
RASSF6	MAC	
TRIM29	MDM4	
NAALADL2	mir-31	
CTDSP1	PTPN22	
NEK7	5-stearic acid hydroxy stearic acid	

STK17A	10-(9Z-hexadecenoyloxy)-octadecanoic acid	
S100A10	Dgk	
SPTAN1	TLR10	
TIP60	HSPB2	
idelalisib	BCL2A1	
CYT003-QbG10	9-palmitic acid hydroxy stearic acid	
GPRC5A	SATB2	
PSMB9	N-[N-(3,5-difluorophenacetyl-L-Ala)]-S-phenylglycine t-butyl ester	
KHSRP	wood smoke particle	
FGF3	oleoyl-estrone	
PI3	TF	
IL22RA2	HOXA5	
DNM3OS	TNFAIP6	
NEDD8	CYP2J2	
SFRP2	CLEC10A	
GUSB	KCNIP3	
ING4	PRKD1	
PANDAR	SMPD1	
HTRA1	TIRAP	
NEK6	KNG1	
CD276	HSPD1	
GPR183	NCOA4	
SCUBE3	irbesartan	
PPP1CA	PTK2	
CD99	lysophosphatidylinositol	
MPL	acetovanillone	
ANPEP	NUP98-DDX10	
AP3B1	epinephrine	
EDA	FLT3LG	
cilostamide	ASAH1	
acrylamide	FCER1G	
racemic flurbiprofen	Fcgr3	
RP 73401	LRBA	
7,8-dihydro-7,8-dihydroxybenzo(a)pyrene 9,10-oxide	C-21	
setanaxib	hemoglobin	
aripiprazole	Cyp2c23	
tangeretin	GPI	
pentazocine	AKR1B1	
tyrphostin AG 127	NfkB-RelA	
SLR14	PTPN2	
miricorilant	LRPAP1	
SETX	GAS5	
IFNK	oxazolone	
MPZ	mir-146	
miR-19b-3p (and other miRNAs w/seed GUGCAAA)	PNPLA2	
miR-218-5p (and other miRNAs w/seed UGUGCUU)	miR-122-5p (miRNAs w/seed GGAGUGU)	
LOX	naringin	
MBD1	DEPTOR	
RFX2	IL1R2	
Sch-23390	RB1CC1	
Zn2+	CCNT1	
fluvoxamine	PINK1	
mir-122	indinavir	
SOX7	rhein	
RTN4	EGLN	
bromocriptine	SCARB1	
cyclic GMP	LTBR	
CMKLR1	mir-124	
ATXN2	HAVCR2	
PURA	NFIC	
CBLB	KEAP1	
ANGPTL2	TNIP1	
TNFAIP2	PSMB11	
GPR37	MNT	
NDN	MAVS	

101.10 peptide	FGFR2	
bosutinib	vitamin E	
cystamine	EGLN1	
WIN 55,212-2	BACH1	
morin	leukotriene B4	
reserpine	SIGIRR	
palmitoylethanolamide	CDC73	
rebamipide	trametinib	
tributyltin	Saa3	
dorsomorphin	HMGB2	
EGR3	alpha-tocopherol	
bafilomycin A1	chrysin	
NR1H4	NR4A2	
ursodeoxycholic acid	PNPT1	
KCNE3	3M-002	
PTH1R	MEF2C	
TRB	neomycin	
selenium	NX-13	
fludarabine	S1PR2	
SOX9	BIRC2	
EIF2A	TEK	
KDM4B	MUC4	
EIF4G1	diethylmaleate	
ACVR1C	GW501516	
chelerythrine	EP4-D	
KAT6A	ibuprofen	
ARV771	lactic acid	
cloprostenol	naringenin	
QC6352	alloxan	
TEC/BTK/ITK/TXK/BMX	blinatumomab	
SNHG1	Tlr11	
tozasertib	SFRP5	
tylophorine	MEFV	
MCU	CCDC50	
CCDC88B	SERPINA4	
LRP4	ST13	
Vacuolar H+ ATPase	enzastaurin	
MARCHF5	autologous CD22-targeted CAR-T cells	
UBA5	RWJ 67657	
LY75	borrelia burgdorferi strain B31 peptidoglycan	
ATF6B	IL23A	
GNL3L	MALT1	
USE1	mir-135	
GLIPR2	DAXX	
HSPBP1	monocrotaline	
UFC1	adavosertib	
SLC13A5	AVP	
NBR2	ZFPM1	
CDK5RAP3	KDM4D	
NDUFA4L2	TRIM38	
MTSS1	PLA2G2D	
CUEDC2	fedratinib	
RPRD1A	KLRG1	
Sapk	mir-127	
asparagine	PLA2G2E	
proanthocyanidin derivative	spermine nitric oxide complex	
PDLIM1	gamma-linolenic acid	
PX 478	pectin	
AZD7762	IL1R1	
nintedanib	ACSL4	
SERPING1	heme	
TBR1	thioctic acid	
RAB11FIP3	mir-144	
CTSD	XIAP	
CCL17	methyl-beta-cyclodextrin	

POU2F3	GATA2	
FGD5-AS1	carvedilol	
SPTBN1	sodium orthovanadate	
CLIP1	HNRNPA2B1	
miR-381-3p (and other miRNAs w/seed AUACAAG)	CYB5R4	
mir-32	ERVW-1	
MIR585	NOD1	
crocin	TNFRSF13C	
CALCR	LY6E	
SGCB	DGCR8	
HUNK	SRC (family)	
lanatoside C	epicatechin	
LSM1	valsartan	
RGD1560225	Collagen type II	
OTULIN	EIF3E	
APLP2	TXNRD1	
BICD2	PTPRC	
ELOA	fasudil	
AKAP13	NCOR1	
chelidonine	olanzapine	
PLK3	Histone h2a	
PLCD4	sodium selenite	
RAN	p70 S6k	
SCIN	dimethyl itaconate	
PODXL	ADA	
1-[2,3-bis(furan-2-yl)quinoxalin-6-yl]-3-(4-bromophenyl)urea	alendronic acid	
TBPL1	UM101	
MK-8776	Stat3-Stat3	
NBN	ETV4	
NOC2L	AIM2	
TXN2	lopinavir	
N-hydroxy-2,2-diphenylacetamide	PDK	
WTAP	enoxaparin	
PCCA-DT	C1QTNF12	
farnesyltransferase inhibitor	lipoxygenase	
3-(3-pyridinyl)-1-(4-pyridinyl)-2-propen-1-one	chlormethiazole	
p38 MAP kinase inhibitor	irisolidone	
belumosudil	TAFA4	
NSC719239	ZNF300	
alpha-methylhydrocinnamic acid	DGKH	
ibotenic acid	NAAA	
centrinone	TH17 Cytokine	
ferrous sulfate	VX 702	
NUP98-HOXD13	Inflammasome	
CCI-007	satratoxin G	
1-eicosapentaenoylglycerol	GPC3	
microcystin-LR	GNLY	
yohimbine	Ccdc50	
karenitecin	U2AF2	
sodium bisulfide	LYZ	
KL	miR-125b-1-3p (miRNAs w/seed CGGGUUA)	
RCE1	RPS14	
NFE2L1	HSPB3	
PCGF2	CARD8	
SAMMSON	aurora kinase inhibitor III	
KAT2B	SGI 1776	
COLQ	FOXO6	
sodium tungstate	GZMA	
lnc-HAND2-2	CRT-0066101	
belinostat	ZNF350	
HOXA11-AS	LRG1	
LINC01139	GJC1	
kremezin	IDR-1018	
LRRC32	Acp5	
DUOXA1	Supt20	

CAVIN1	iso[4]levuglandin E2	
PPM1D	CYM50358	
HLA-DQB1	Ro-1138452	
SEMA4D	mercaptosteroid 4	
PDK2	mercaptosteroid 6	
MIR320	14-oxoDHA	
P2RX4	LY3214996	
SETD7	tempo	
CDCP1	cibacron blue F 3GA	
LILRB3	bongkrekic acid	
HOXD9	phosphorylcholine	
TP53BP2	N-methylsulfonyl-12,12-dibromododec-11-enamide	
LAT2	silver nitrate	
KCNJ11	methoxyluteolin	
SYNCRIP	potassium tetraperoxochromate	
GPAT4	TAPI	
ZMYND8	ODN-BW006	
ulixertinib	ellipticine	
RECK	strychnine	
PRKCH	glyphosate	
INCB054329	pegylated leptin antagonist	
diltiazem	danazol	
allyl isothiocyanate	epiallopregnanolone	
calmidazolium	belnacasan	
NF 449	TRAF3IP3	
PCI-34051	PECAM1	
L-lactic acid	mir-302	
sodium dodecyl sulfate	miR-196a-5p (and other miRNAs w/seed AGGUAGU)	
levamisole	F10	
calcium chloride	SRSF3	
tin mesoporphyrin	erastin	
mastoparan	CDK6	
alpha-naphthoflavone	coomassie brilliant blue	
oleoylethanolamide	nelfinavir	
C-miR146a	Aldose Reductase	
N-chlorotaurine	miR-31-5p (and other miRNAs w/seed GGCAAGA)	
desoxycorticosterone	TRIM3	
(+)-catechin	ACKR1	
Cyclin E	LIPG	
KLF9	tranilast	
MIR101	mangiferin	
HBP1	epirubicin	
ITGB6	cilostazol	
NBEAL2	CAY10585	
AQP7	N-Cadherin	
HDAC8	saracatinib	
ECM1	CD33	
nimesulide	miR-140-5p (and other miRNAs w/seed AGUGGUU)	
polyamines	FZD5	
DIM-C-pPhOH-3-Cl-5-OCH3	RBCK1	
leupeptin	huperzine A	
canrenoate potassium	vinpocetine	
manganese	CD3 group	
ONECUT1	N-ethyl-N-nitrosourea	
beta-naphthoflavone	RNASEL	
PCGEM1	RNASE2	
SLC9A3R1	resolvin D1	
TBX21	SUMO1	
Relaxin	UBE2I	
TRA	miR-133a-3p (and other miRNAs w/seed UUGGUCC)	
EXOSC3	CHADL	
mir-7	CNR2	
HSD17B4	MSC	
ochratoxin A	SFRP1	
chenodeoxycholic acid	lactosylceramide	

DSCAML1	prostaglandin A1	
LAS1L	HCAR1	
clozapine	ITGA9	
THBS4	SFTPD	
IFNE	MAP3K5	
lipid	CCN3	
KDM5A	GLP-1-(7-34)-amide	
deoxycorticosterone acetate	mezerein	
thyroid hormone receptor	MTTP	
BPIFB1	taurocholic acid	
SIRT2	JINK1/2	
MST1	RPSA	
miR-103-3p (and other miRNAs w/seed GCAGCAU)	BGN	
mir-103	ETV1	
JARID2	R-WIN 55,212	
SRD5A1	TAP1	
8-(4-chlorophenylthio)-guanosine-3', 5'-cyclic monophosphate	GHRHR	
PI-103	PER2	
Sox2ot	GLUL	
ghrelin	ebselen	
CBS/CBSL	BSCL2	
PHB2	TRIM28	
XRCC6	TLR8	
TRAF5	ABCG1	
mir-515	KDR	
GSN	IMMT	
ATXN7	NF2	
SOD3	parthenolide	
TNFAIP8	DNMT3B	
sertraline	capsaicin	
isoliquiritigenin	SUPT16H	
pterostilbene	herbimycin	
MEF2A	omeprazole	
CHRNA7	R 59022	
mir-126	C8	
PBX1	BLVRA	
indole-3-carbinol	lentinan	
POSTN	FBXO42	
HBA1/HBA2	MARCHF2	
Irp	salmonella typhimurium lipopolysaccharide	
UDP	genipin	
Stat1 dimer	IL4I1	
coal tar	PRSS8	
HOXA-AS2	CSNK1A1	
DLX6-AS1	C7	
CEMIP	HA900	
MGAT1	ivermectin	
T 0070907	nigericin	
DPY30	adapalene	
MTF1	Pde4	
LTB4R2	CD200	
HDAC11	MED12	
PPP1R15B	SLC2A1	
DGCR5	SCGB1A1	
SNHG20	dipyridamole	
CD80/CD86	pifithrin alpha	
Traj18	CXCL10	
Gcn5l	IDR-1002	
G2535	NfkB1-RelA	
TXK	SIGLEC8	
CD53	tempol	
HSP90AA1	LINC00662	
NRBP2	miR-143-3p (and other miRNAs w/seed GAGAUGA)	
KRT19	TNFRSF25	
mir-320	erythromycin	

miR-142-3p (and other miRNAs w/seed GUAGUGU)	glyburide	
miR-185-5p (and other miRNAs w/seed GGAGAGA)	caffeic acid	
ADAR	RGS10	
PTCSC3	mir-29	
C6	IL4R	
CAMK2G	RUNX1-RUNX1T1	
OSTM1	streptozocin	
TNFSF8	BAK1	
MANF	SMAD5	
GIPR	Ciap	
ENTPD1	PADI2	
ELK3	PCSK9	
Cxcl3	FSTL1	
MAP3K11	TRIM21	
TAS1R3	vanillin	
CYBA	SB 290157	
BP-1-102	behenic acid	
pargyline	treprostinil	
clioquinol	hypoxanthine	
kukoamine A	L-alanine	
enterolactone	N4	
rosmarinic acid	repertaxin	
tin protoporphyrin IX	Rsk	
phospholipid	tyrosine kinase	
Z-DEVD-FMK	chitinase	
allopregnanolone	CACTIN	
trestolone	ZXDC	
maneb	TNIP3	
GCG	HECTD3	
GATA6	B3GNT2	
ALDH2	SPATA2	
CNGA3	ASCC1	
HDAC9	TEX11	
cyanocobalamin	SLC22A3	
11-deoxyprostaglandin E1	TRIL	
Mst/krs	DACT3	
styrene	LRRC19	
MKNK	tetrahydrocurcumin	
Cpla2	VLDL	
Erm	perfluorooctane sulfonic acid	
anthralin	cerotic acid	
MELK	MZB1	
ETHE1	CDC25B	
GPR65	mir-766	
CHD5	mir-361	
INSL5	ORM2	
ZNF524	PHLDA1	
ZC3H10	MEP1A	
PYHIN1	NASP	
G6PC3	HSPB6	
sGC	SLC7A11	
SLU7	GJB2	
MPC1	APOD	
IgD	NLRC3	
harmol	Rps6ka5	
benzenesulfonic acid	S100A2	
7S NGF	LSINCT5	
Atf	SNRK	
FGR	SKLB023	
GRAP2	S3I-1757	
ERN2	CSTB	
CRK	CAY10397	
PUF60	GR-MD-02	
CBX4	(+)-epicatechin	
LRP8	DEETGE-CAL-Tat	

NUP98	ITF3056	
ZBTB46	carboxyamido-triazole	
NCK2	pyrazole	
miR-570-3p (miRNAs w/seed GAAAACA)	CV 6209	
MIR4269	ascomycin	
miR-379-5p (and other miRNAs w/seed GGUAGAC)	necrostatin-1s	
miR-1285-3p (and other miRNAs w/seed CUGGGCA)	protoporphyrin IX	
mir-1275	4-methylcatechol	
PPP1R13B	senexin B	
LGMN	vapreotide	
RHOH	miR-499a-5p inhibitor	
UTS2	miR-208b inhibitor	
XAF1	perillic acid	
WFS1	erucic acid	
RAP1GDS1	15-hydroxyeicosatetraenoic acid	
DLGAP1	titanium	
ARLNC1	vigabatrin	
BRCA2	IgG-opsonized ovalbumin	
RANBP1	pubchem compound 16020046	
NME3	Vi capsular polysaccharide	
GTF2H4	cholesterol ester	
CCL22	Ccl2	
SH2B2	telmisartan	
ARL5B	SMO	
GTF3A	KIN001-043	
SLC37A4	NOS3	
ribociclib	FASN	
RACGAP1	CBFB	
N-lauroyl-L-phenylalanine	NR0B2	
DDT	prostaglandin J2	
PLAA	NCF1	
Mcpt1	NPPB	
SNHG22	PRKD	
RFXANK	UPF2	
GSK-2606414	6-mercaptopurine	
polyinosine-polycytidylic acid/polyethylenimine formulation	PANX1	
phenylmethylsulfonyl fluoride	ELOVL2	
SQ 29548	Shc	
vinyl carbamate	B4GALT6	
plevitrexed	secukinumab	
dimaprit	LGALS8	
butylated hydroxytoluene	RNF41	
etidronic acid	ATP1B1	
paxilline	IGBP1	
H-8	CXCL16	
ethyl protocatechuate	NFKBIE	
sodium azide	CTTN	
sodium chlorate	IKK-2 inhibitor VIII	
1-docosapentaenoylglycerol	hexamethoxyflavone	
FT671	poly(U)RNA	
geraniol	UCN2	
omacetaxine mepesuccinate	Hmgb1	
chlorine	ZDHHC7	
5-hydroxydecanoic acid	FZD9	
vidarabine	IL13RA2	
pemetrexed	IL-17f dimer	
marinobufagenin	SDC1	
kb-NB 142-70	BCL10	
SETD2	USP1	
PTGS1	zearalenone	
DIO2	HOXC6	
Srebp	paricalcitol	
TMSB4	Pro-inflammatory Cytokine	
miR-486-5p (and other miRNAs w/seed CCUGUAC)	ZEB2	
ATR	plumbagin	

GNA11	TASL	
EMD	UCP2	
NRTN	SLPI	
SCP2	MSX1	
acetyl-L-carnitine	PRMT1	
helenalin	APOA1	
FTO	mir-34	
4-tert-octylphenol	OSCAR	
	GLIS2	
	ZIC3	
	DMD	
	CTCF	
	IRAK1	
	GHR	
	NKX2-1	
	DNMT3A	
	resolvin E1	
	mir-9	
	IL36G	
	PSMD10	
	CXCR2	
	CLEC4E	
	ARHGDIG	
	EPHX2	
	ADORA1	
	IRX5	
	pimozide	
	10-(6'-ubiquinonyl)decyltriphenylphosphonium	
	TGAL copolymer	
	iloprost	
	MBD3	
	FHL2	
	ziritaxestat	
	HOXC8	
	MEIS1	
	FUS-DDIT3	
	azathioprine	
	nitroglycerin	
	teriflunomide	
	tocilizumab	
	miR-193a-3p (and other miRNAs w/seed ACUGGCC)	
	miR-186-5p (miRNAs w/seed AAAGAAU)	
	LIMA1	
	KLF7	
	JMJD1C	
	MYZAP	
	H3C14	
	CA074-methyl ester	
	PITX2	
	mir-183	
	NOG	
	poly dA-dT	
	8-epi-prostaglandin F2alpha	
	L-aspartic acid	
	beta-glucan	
	teniposide	
	H-151	
	Adenosine Receptor	
	xylooligosaccharide	
	AOPEP	
	ATPase	
	aurapten	
	CYP	
	RNF187	
	IL12RB2/IL23R	

	DACT1	
	SPINDOC	
	BPI	
	Snhg8	
	TAS2R14	
	PLA2G7	
	ALDH3A2	
	TAF9	
	IgG2b	
	azilsartan	
	Cathepsin	
	Mucin	
	GIT1	
	AP3D1	
	DCB 3503	
	RAP1B	
	DAPK1	
	STX11	
	UBB	
	HAO1	
	mir-612	
	HADHA	
	FGA	
	FGG	
	RBM14	
	PILRB	
	Orm1 (includes others)	
	CXADR	
	DCX	
	P2RY12	
	RPS3	
	ADAM8	
	IL36RN	
	AQP3	
	SPIN1	
	VCAM1	
	TG6-10-1	
	Muc1	
	NT157	
	CLDN2	
	CMP5	
	H3B-8800	
	sulfamethoxazole/trimethoprim	
	methylamine	
	N-hydroxy-N'-(4-butyl-2-methylphenyl)formamidine	
	CP 96345	
	tranexamic acid	
	ethacrynic acid	
	roflumilast	
	opioid	
	CAY10595	
	sappanone A	
	ampelopsin	
	L-buthionine (SR)-sulfoximine	
	L-homocysteine	
	9-(9Z-octadecenoyloxy)-octadecanoic acid	
	L-alpha-hydroxyglutarate	
	HBB	
	beta-carotene	
	CHD4	
	EGOT	
	MFN2	
	GPR132	
	CSF3R	
	ANGPTL4	

	PS-1145	
	27-hydroxycholesterol	
	RCAN1	
	GSR	
	SOX10	
	FKBP5	
	CORT	
	pyruvaldehyde	
	2,4-dinitrofluorobenzene	
